# Four-Winged Propeller-Shaped Indole-Modified and Indole-Substituted Tetraphenylethylenes: Greenish-Blue Emitters with Aggregation-Induced Emission Features for Conventional Organic Light-Emitting Diodes

**DOI:** 10.1021/acsomega.2c05914

**Published:** 2022-11-17

**Authors:** Ferruh Lafzi, Yunus Taskesenligil, Betül Canımkurbey, Selin Pıravadılı, Haydar Kilic, Nurullah Saracoglu

**Affiliations:** † Department of Chemistry, Faculty of Sciences, Atatürk University, Erzurum25240, Türkiye; ‡ Sabuncuoglu Serefeddin Health Services Vocational School, Amasya University, Amasya05100, Türkiye; § Materials Technologies, Marmara Research Center (MAM), The Scientific and Technological Research Council of Turkey (TUBITAK), Gebze, Kocaeli 41470, Türkiye

## Abstract

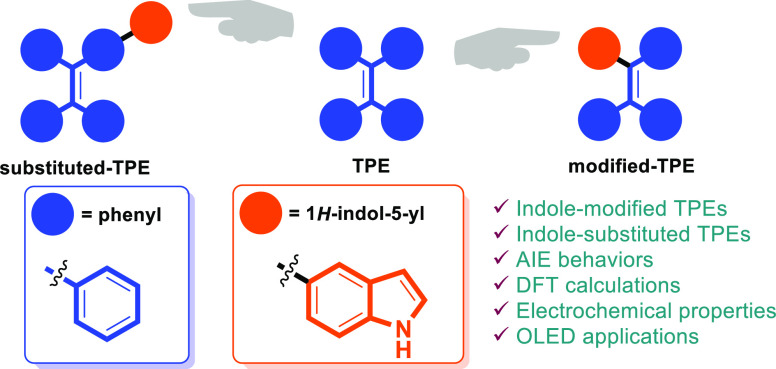

Aggregation-induced emission (AIE) is an extraordinary photochemical phenomenon described by Tang’s group in 2001, where the aggregation of some organic molecules enhances their light emission by limiting intramolecular activity in the aggregate state. This phenomenon offers new opportunities for researchers due to its potential applications in optoelectronics, energy, and biophysics. Tetraphenylethylenes (TPEs) are reliable AIE luminogens with a wide range of successful applications in material chemistry. To expand the library of AIE-active TPEs, both a series of TPE analogues, in which the phenyl rotor has been replaced by the indole ring, and indole-substituted TPE derivatives were designed and synthesized through vinyl–aryl and aryl–aryl bond formations using the Suzuki coupling reaction. Efficient synthetic routes that delivered indole-modified and indole-substituted TPEs have been developed, and almost all heterocyclic TPE analogues have demonstrated AIE behavior. Furthermore, to test whether the indole ring can be diversified, two title compounds were converted to a series of bis(indolyl)methane (BIM), and these BIM–TPE materials showed typical AIE properties. Interestingly, two compounds indicated a solvent vapor fuming reversible switch between bright blue emission and greenish-yellow emission. Upon fuming with dichloromethane, their fluorescence spectra showed 8 and 32 nm red-shift and could return to the original state after fuming with hexane. Furthermore, we have explored the effects of replacing the phenyl ring in TPE with indole together with the substitution of TPE with indole ring(s) on the performance of organic light-emitting diode (OLED) device applications. In addition, density functional theory calculations; the optical, electrochemical, light emission, electroluminescence characteristics; and admittance spectroscopic analysis of OLED devices of four representative TPEs have been investigated in detail. As a result, the indole–TPEs are potential blue emitters with AIE features for conventional OLEDs, which is a significant color in displays and lighting.

## Introduction

**Figure 1:**
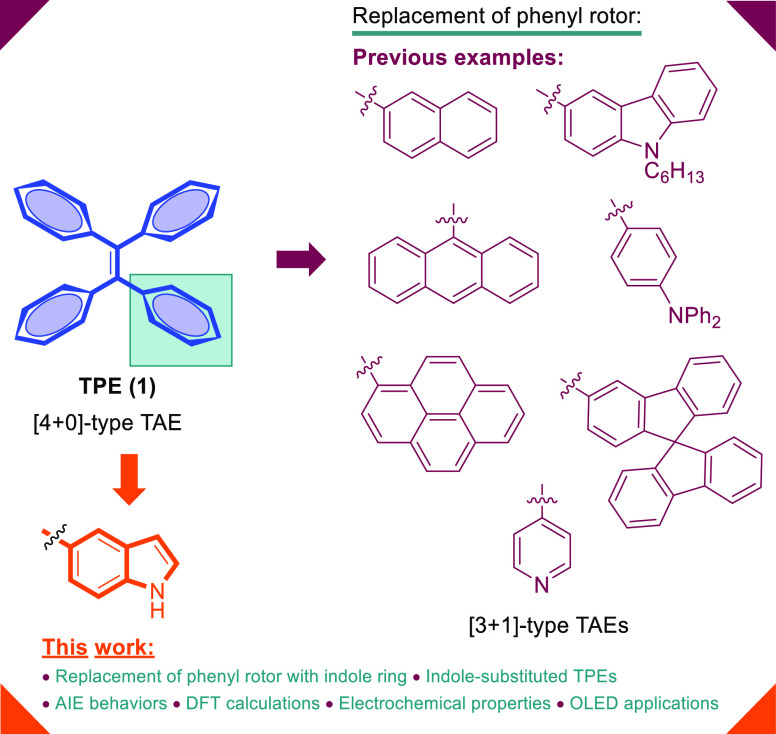
Previous examples for modified TPEs.

Luminescent materials have attracted increasing attention, in the field of science and technology, due to their wide range of applications such as fluorescent probes, sensors, bioimaging agents, and organic light-emitting diode (OLED) devices, in recent years.^1−4^ In 2001, the discovery of the concept of aggregation-induced emission (AIE) opened new avenues for the development of advanced luminescent materials in the aggregate or solid state.^5^ AIE, an opposite phenomenon of the conventional aggregation-caused quenching (ACQ) effect, has raised great attention from scientists.^6^ In most luminogens, the ACQ and AIE effect competes depending on the molecular structures and the intermolecular packing. Since the report of the AIE phenomenon, tetraphenylethylene (TPE, **1**) is the most studied and the most popular AIE luminogen to construct highly efficient solid-state emitting materials because of its simple molecular structure and facile modification (Figure 1).^1b^ The typical AIEgens exhibit poor fluorescence when fairly dissolved in a solvent such as tetrahydrofuran (THF), toluene, and chloroform, whereas the emission strongly increases as a consequence of the aggregate formation by the addition of a poor solvent for AIEgens which reduces their solubility such as water. During the past decade, both experimental and theoretical studies have proposed that the main cause of the AIE effect is the restriction of intramolecular motion, including rotation, vibration, and so forth in the aggregates.^7,8^ Since then, many studies have reported on the design and synthesis of AIE luminogens possessing advantages such as efficient solid-state emission, facile synthesis, and ease of functionalization.^9,10^ Till date, a large number of TPE-based AIEgens have been developed, and their potential applications have been evaluated.^11^ In the solution state, the rotation of phenyl rotors in TPE leads to annihilating the excitons in a nonradiative transition mode. However, intramolecular rotation (IMR) of the rotor is restricted in the aggregated state, resulting in the predominant radiative transition of the excited state, so that the luminogens render emissive.^12^ Therefore, diversification of aromatic rotors is important to create AIE luminogens.^13^ Replacement of phenyl rotors in TPE by various aromatic groups such as naphthalene,^12^ anthracene,^14^ pyrene,^15^ triphenylamine,^9a,16^ carbazole,^9a^ spirobifluorene,^17^ and pyridine^18^ have allowed a generation of new AIE luminogens (Figure 1). The substitution effect of TPE core was studied in terms of synthesis, photophysical properties, and applications.^19^ These materials have emerged as promising candidates for OLEDs as an example of optoelectronic devices. Until now, TPE-based OLEDs have been reported in the literature, and most of them possess a better hole-transporting ability than an electron-transporting one.^20^ Tang’s group reported the LEDs fabricated from nonemissive tetraphenylethylene (TPE, **1**), and its diphenylated derivative emitted blue light with a maximum luminance of ∼1800 and 11000 cd/m^2^, respectively.^21^ TPE (**1**) has no good emission, however, its electroluminescence (EL) emission is shown in the deep-blue region. Modifications of TPE have provided novel AIE emitters with superior light output efficiency.^22^ Considering applications in chemistry and materials science in the last two decades, the tetraarylethylenes (TAEs) have been classified into five groups based on the aryl groups, such as [4 + 0]-, [3 + 1]-, [2 + 2]-, [2 + 1 + 1]-, and [1 + 1 + 1 + 1]-TAEs.^23^

Among heterocycles, indole and its derivatives are the parent substance of a large number of important compounds that occur in nature and exhibit a broad pharmacological profile.^24^ Indole analogues are considered to be one of the most important classes of heterocyclic compounds due to their several applications in the medical fields. Also, indoles are capable of having potential physical properties for many different applications due to their unsaturated and electron-rich structures. Indole-derived compounds may represent photochromism and EL; thus, some of these compounds have been commonly used in the designs associated with nonlinear optics, two-photon fluorescent probes, cell imaging, photothermal ablation of cancer cells, solar cells, and optoelectronic materials.^25^ Especially, indole-based triazatruxene materials remain attractive to chemists due to their various organic electronics and optoelectronic applications.^26^ Remarkable properties of both indole and TPE encouraged us to the synthesis of TPE-based indole derivatives. Till date, most studies have been restricted with more carbocyclic-based aryl groups as the rotor. We hereby introduced a new heteroaryl-based molecular rotor where the phenyl ring was replaced with an indole unit. We wondered whether this replacement caused changes in the photophysical and EL properties of TPE, a splendid AIE-luminogen with a twisted, propeller-like conformation. Herein, we reported a series of indole-modified triphenylethenes ([3 + 1]-type TAEs), including their photophysical properties in the aggregated state. Also, we have synthesized TPEs ([4 + 0]-type TAEs) with indol substituent(s) on the TPE core and investigated in detail their AIE behavior. Furthermore, to investigate their AIE characterization, some indole derivatives were converted to bis(indolyl)methanes (BIM). In addition, the OLED performance measurements and admittance spectroscopy of four propeller-shaped indole-modified and indole-substituted TPEs were also investigated as an emissive layer (EML) to achieve blue emission in OLEDs, which has been the important color in display and lighting areas.

## Results and Discussion

### Synthesis and Functionalization

**Scheme 1:**
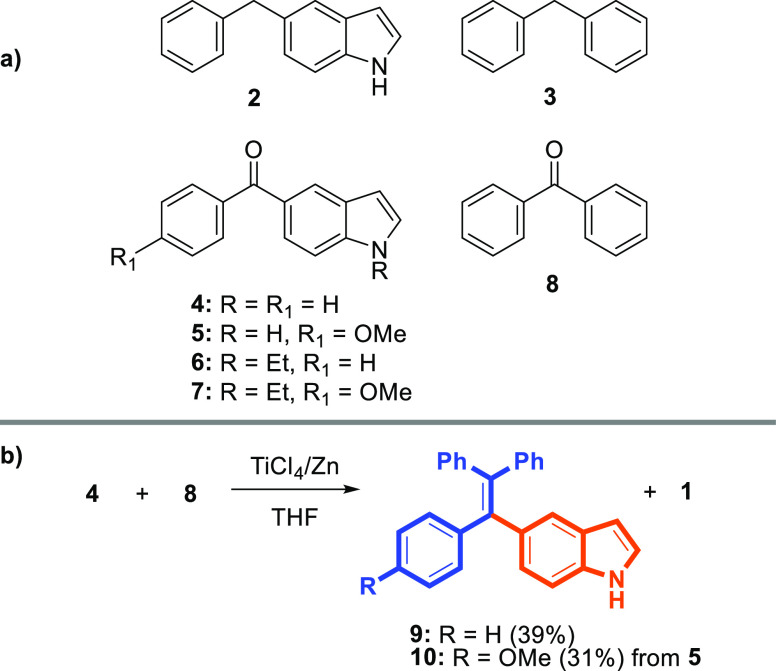
Used Diarylmethanes and Diaryl Ketones; Synthesis of TPE **9** and **10** via McMurry Coupling

We applied the Knoevenagel condensation and McMurry coupling as methods to access our desired indole-modified TPEs. As condensation or coupling partners, 5-benzyl-1*H*-indole (**2**)^27^ and diaryl ketones (**4–7**)^28^ were synthesized via a palladium-catalyzed cross-coupling reaction, as reported in the literature (Scheme 1a). First of all, the reactions between diphenylmethane (**3**) and (1*H*-indol-5-yl) (phenyl)methanones (**4–7**) in the presence of *n*-butyl lithium followed by dehydration, which failed to provide the desired Knoevenagel condensation product. The cross-couplings of indol-5-yl-phenylmethanone (**4**) and benzophenone (**8**) in the presence of TiCl_4_ and zinc dust afforded the desired indole-modified TPE **9** in a low yield (39%) together with TPE (**1**) (Scheme 1b). However, the reaction between **5** and **8** led to the formation of a mixture of indole–TPE **10** and **1** in a low yield.

**Scheme 2:**
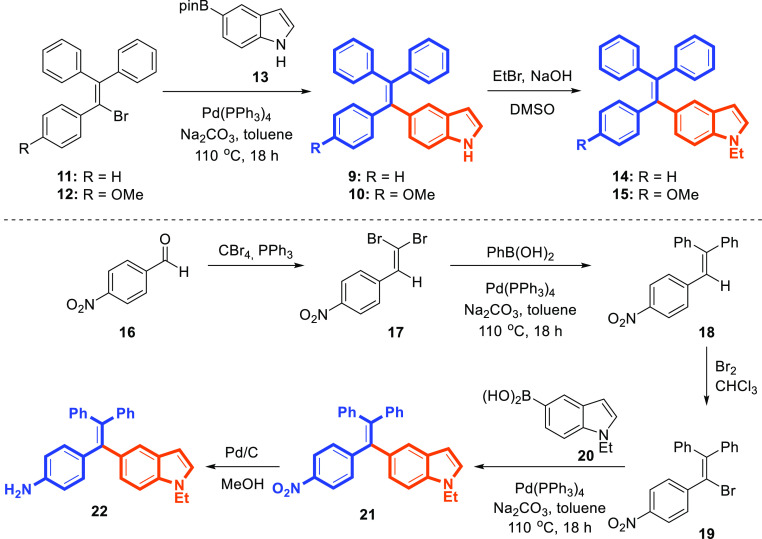
Synthesis of Indole-Modified TPEs **9**, **10**, **14**, **15**, **21**, and **22**

We then investigated the feasibility of the Suzuki coupling reaction as our initial efforts to indole-modified ethenes **9** and **10**, via Knoevenagel condensation and McMurry coupling were not efficient and fruitful. Initially, 1,1,2-triphenyl-2-bromoethenes **11** and **12** as starting materials were synthesized following literature protocols.^29^ We selected the Suzuki coupling reaction between 1,1,2-triphenyl-2-bromoethene (**11**) and indolyl-5-pinacol boronate (**13**) as model substrates that would establish indole-modified ethene **9** (Scheme 2). We were pleased to verify that coupling in the presence of Pd(PPh_3_)_4_ as a catalyst with aqueous Na_2_CO_3_ as a base in toluene at 110 °C afforded the desired product **9** in a 70% yield. We examined the scope of the substrates in this catalytic system (Scheme 2). Aryl pinacol boronate **13** was coupled with **12** to produce the corresponding indole-modified alkene **10** in good yield, whereas indole–TPE compound **21** was synthesized in good yield via the reaction of bromoethene **19** prepared from **18**(30) with *N*-ethylindole-5-boronic acid (**20**)^31^ (Scheme 2). However, *N*-alkylated-TPEs **14** and **15** were also prepared from free (NH)–indole–TPEs **9** and **10** and alkyl bromides in the presence of NaOH in dimethyl sulfoxide (DMSO) at room temperature. Additionally, nitro derivative **21** was reduced to the corresponding amine **22** in high yield (95%) via catalytic hydrogenation (Scheme 2).

**Scheme 3:**
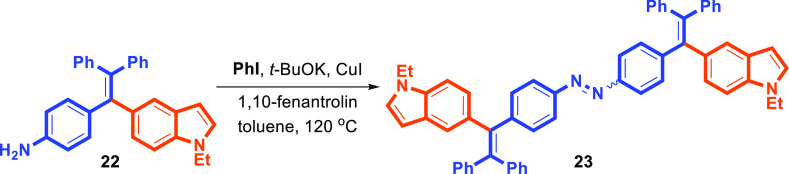
Unusual Formation of **23**

**Scheme 4:**
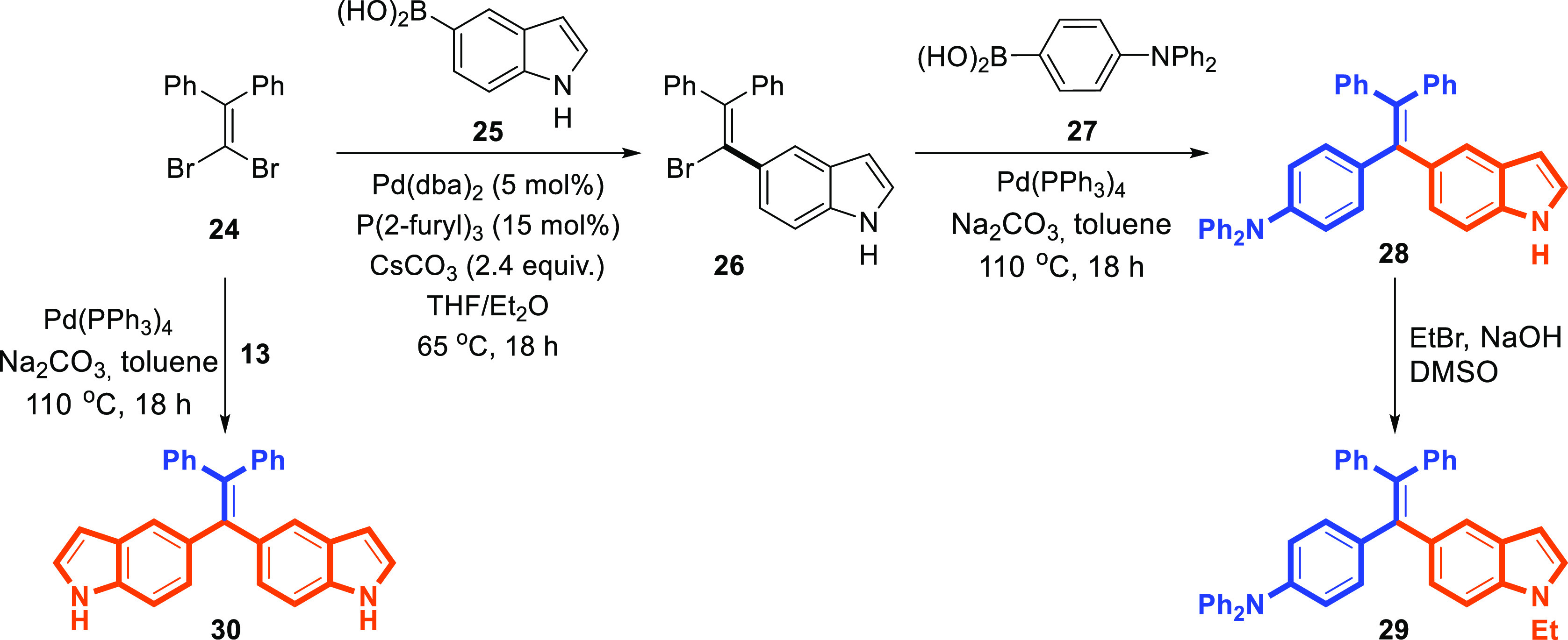
Synthesis of Indole-Modified TPEs **28–30**

Later, we attempted the synthesis of the *N*,*N*-diphenylamine-substituted indole–TPE **29** (see Scheme 4). For this, treatment of amine-substituted TPE **22** with iodobenzene in the presence of CuI/1,10-phenanthroline as a ligand and *t*-BuOK as a base in toluene at 120 °C for 12 h led to unexpected product **23** in 70% yield instead of the desired product **29** (Scheme 3).^32^ We assume that unexpected N=N bond formation proceeded via the CuI-triggered oxidative dehydrogenative coupling of amine **22** to azobenzene **23**.

Furthermore, the selective synthesis of **29** was achieved via the successive Suzuki-coupling reactions with two types of boronic acids starting from geminal dibromo-alkene (Scheme 4). The reaction between geminal-dibromoethene^33^**24** and indole-5-boronic acid (**25**) was carried out with tri(2-furyl)phosphine as the ligand in the presence of Pd(dba)_2_ as a catalyst. This reaction condition revealed that the choice of phosphine ligand was crucial.^34^ The sole product in the reaction was the desired cross-coupled product **26** in good yield. No bis-cross-coupling product **30** was observed in any case. In the presence of palladium/Na_2_CO_3_ catalysts, the cross-coupling of **26** and **27** gave the desired indole–TPE **28**. Furthermore, an *N*-alkylated product of **29** was obtained (Scheme 4). Next, we turned our focus toward the bis-cross-coupling reaction of geminal-dibromide **24** (Scheme 4). The synthesis of 5,5′-(2,2-diphenylethene-1,1-diyl)bis(1*H*-indole) (**30**) is illustrated in Scheme 4 and was similar to the straightforward procedures that were reported in the previous synthesis route. Briefly, double Suzuki coupling between **24** and indoleboronic acid pinacol ester **13** then afforded **30** in good yields (Scheme 4).

**Scheme 5:**
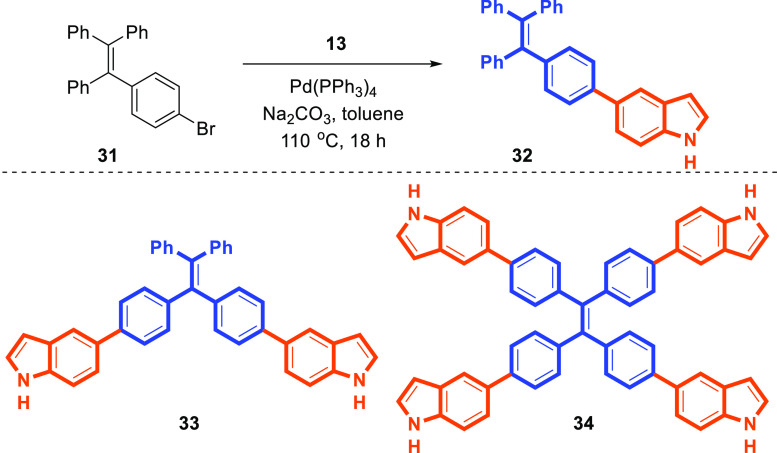
Synthesis of Indole-Substituted TPEs **32–34**

Besides, the indole-substituted TPE derivative **32** was synthesized by the Pd-catalyzed Suzuki cross-coupling reaction of 1-bromo-4-(1,2,2-triphenylethenyl)benzene (**31**)^35^ with the 5-indoleboronic acid pinacol ester (**13**) in 85% yield (Scheme 5). Similarly, multiple indolyl units (as **33** and **34**) were installed onto the TPE scaffold through Suzuki cross-coupling reactions (Scheme 5). The precursor bromo-TPE derivatives of **32–34** were synthesized by reported procedures.^36^

**Scheme 6:**
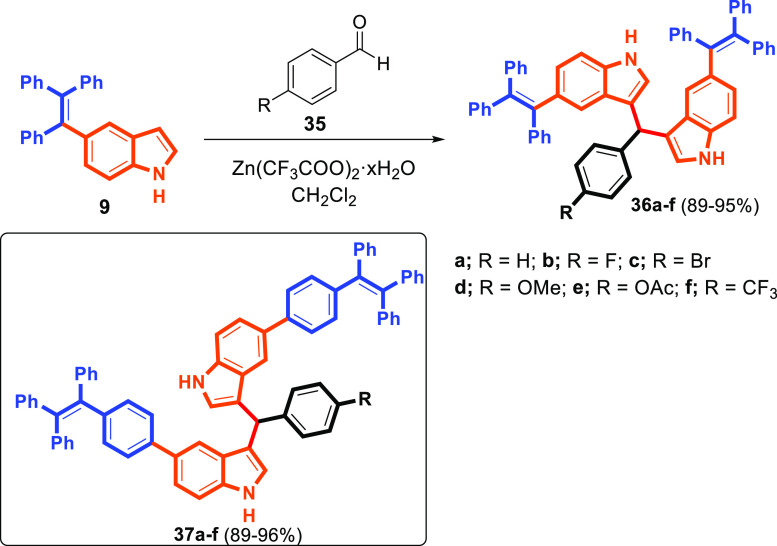
Synthesis of BIM–TPEs **36a–f** and **37a–f**

Indoles can be easily diversified with high functional group tolerance and are one of the most prevalent structures in functional materials. However, BIMs and their derivatives are also present in a variety of natural products, synthetic compounds, and colorimetric sensors. Therefore, we aimed to test whether both indole-modified and indole-substituted TPEs were diversifiable. The reaction of indole **9** with benzaldehyde (**35a**) in the presence of zinc triflate afforded BIMs **36a** including TPE-core via a Friedel–Crafts-type alkylation (Scheme 6). The BIMs **37a** were synthesized from the reaction of **35a** and TPE–indole **32** under the same reaction conditions (Scheme 6). A wider scope of substituents on the phenyl ring was very well tolerated, leading to the depicted BIMs **36a–f** and **37a–f** in excellent yields (89–96%) (Scheme 6).

### AIE Characteristics

**Figure 2:**
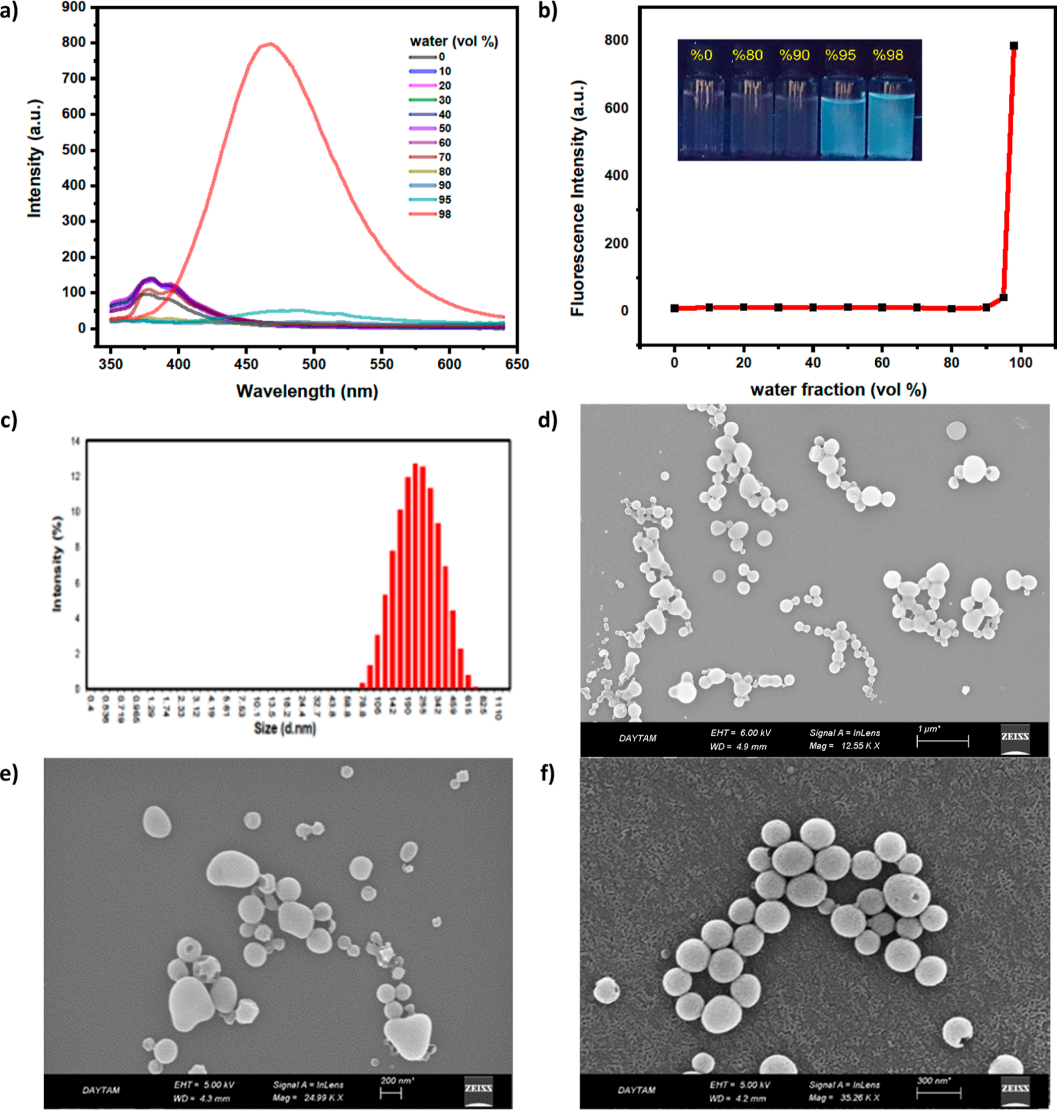
(a) Fluorescence spectra of **9** in THF and THF–water mixtures with different water fractions; (b) plot of peak fluorescence intensities of **9** in THF–water with different water fractions. Luminogen concentration: 20 μM; λ_ex_ = 330 nm; intensity calculated at λ_max_. Photograph of **9** in THF–water mixtures (*f*_w_ = 0–98%) with different water fractions (20 μM) under 365 nm UV illumination. Photograph of **9** in water–THF mixed solution (*f*_w_ = 0–98%) under an UV lamp; (c) DLS particle size distribution profile of **9** in THF–water mixtures (2:98, v/v); and (d–f) SEM images.

To test whether indole-modified and indole-substituted TPEs (**9**, **10**, **14**, **15**, **21**, **22**, **28–30**, **32–34**, **36a–f**, and **37a–f**) were AIE active, the AIE properties of compounds were evaluated in THF/water solvent mixtures through gradual increments of water (*f*_w_). These compounds were well dissolved in THF but completely insoluble in water. The AIE characteristics were explored using emission and absorption spectroscopies. As a model compound, the UV–vis absorption spectra of **9** exhibited the main absorption band peaked at ∼279 nm (Figure 2a), whereas the solution of indole-modified TPE **9** in a pure THF was almost non-emissive (Figure 2b). Also, the photoluminescence (PL) of **9** in the THF–water mixture remained very weak up to the 90% water fraction (*f*_w_), while the PL intensity started to rise swiftly for *f*_w_ > 90% (Figure 2b). At 98% water fraction, the PL intensity of **9** was increased by 750-fold at 468 nm (Figure 2b). The significant enhancement of the emission intensity was attributed to the formation of aggregates, which resulted in the constraint of the IMR process. The photograph of **9** in different THF–water mixtures under 365 nm UV light showed the AIE behavior (Figure 2b). To prove the formation of molecular aggregates, the nanoaggregates at 98% water fraction (*f*_w_) were also studied with the cooperation of dynamic light scattering (DLS) and scanning electron microscopy (SEM) measurements. The DLS study showed the formation of nanosized particles with average sizes of 260 nm (Figure 2c). On the other hand, the SEM image revealed the presence of micrometer-sized aggregates pieced together by nanosized particles (Figure 2d–f).

Almost all compounds showed similar emission behavior in the water–THF mixture (see the Supporting Information), supporting that these luminogens were also AIE-active.

### Mechanofluorochromic Behaviors

**Figure 3:**
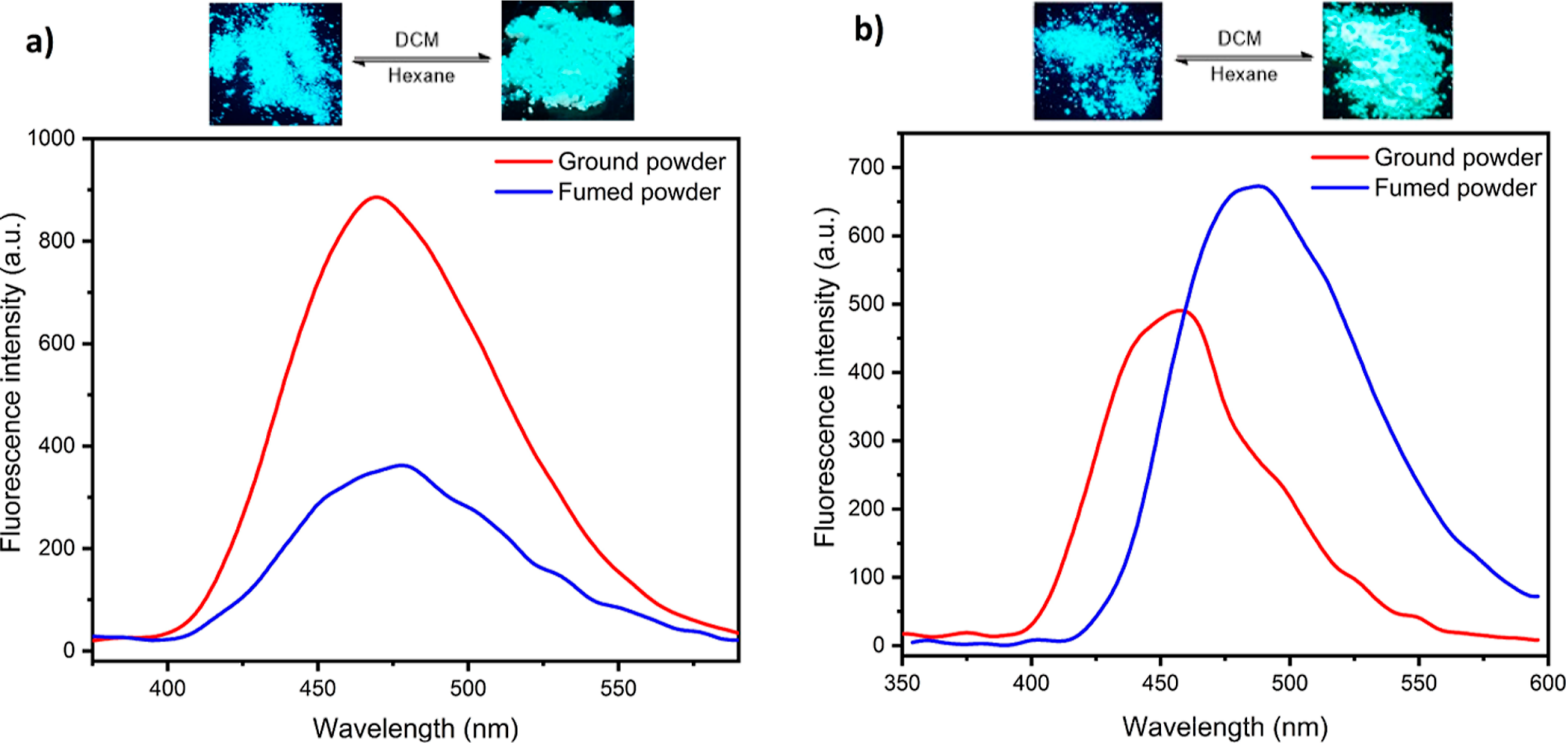
Images of **9** and **32** in the ground (left) and solvent-fumed (right) samples under an UV lamp (365 nm). PL spectra of the ground and fumed powders of (a) **9** and (b) **32**.

Subsequently, the mechanochromic characteristics of indole–TPEs in hand were researched by PL spectroscopy. Among the indole–TPP–luminogens, indole-modified TPE **9** and indole-substituted TPE **32** displayed fluorescent solvent fuming behavior, as shown in Figure 3. The solid-state emission spectra were measured to compare with that of **9** and **32** (before fuming), respectively. The luminophores exhibited bright blue emission and greenish-yellow emission before and after fuming under UV excitation at 365 nm, respectively. Even, the color change of solids upon fuming operation was visible to the naked eye under UV excitation and daylight. The luminophores exhibited the emission maximum at 478 and 488 nm in the ground form before fuming. Compound **9** upon fumigation exhibited a slight bathochromic shift by 8 nm in comparison with the basic form. On the other hand, **32** was remarkably red-shifted by 32 nm (from 456 to 488 nm) after fuming. We attributed that the extent of planarization (and hence conjugation) after fuming was not the same in both compounds. The fumed samples could be completely switched into the ground state by hexane fumigation.

### OLED Device Application

In the next stage, we focused on whether four indole-modified and indole-substituted TPEs (**9** and **32–34**) could be used as an EML for conventional OLED device applications. For this purpose, the optical, theoretical, and electrical properties of these TPE-based OLEDs were investigated in detail.

### Photophysical Properties

**Figure 4:**
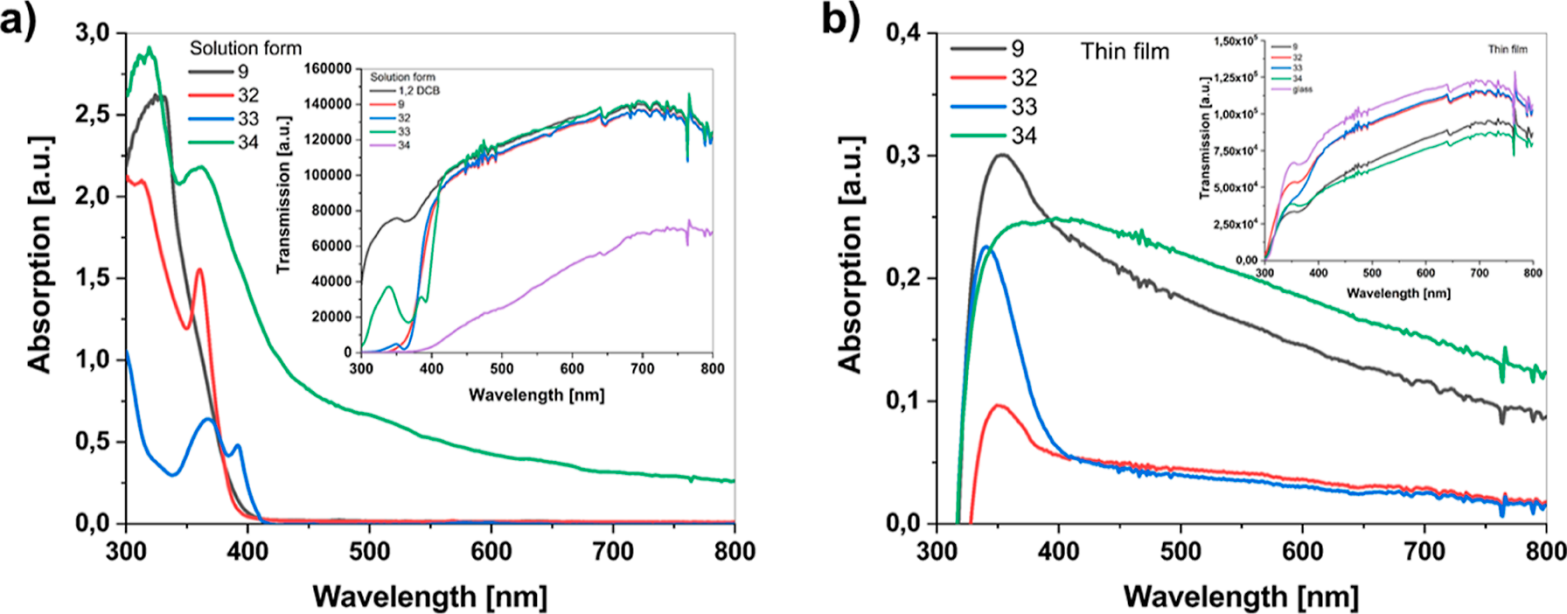
(a) UV–vis absorption spectra of solution form of TPEs **9**, **32**, **33**, and **34** in 1,2-DCB (inset: transmission spectra of solution form of TPEs **9**, **32**, **33**, and **34** in 1,2-DCB) and (b) UV–vis absorption spectra of spin-coated thin films of TPEs **9**, **32**, **33**, and **34** (inset: transmission spectra of spin-coated thin films of TPEs **9**, **32**, **33**, and **34**).

**Figure 5:**
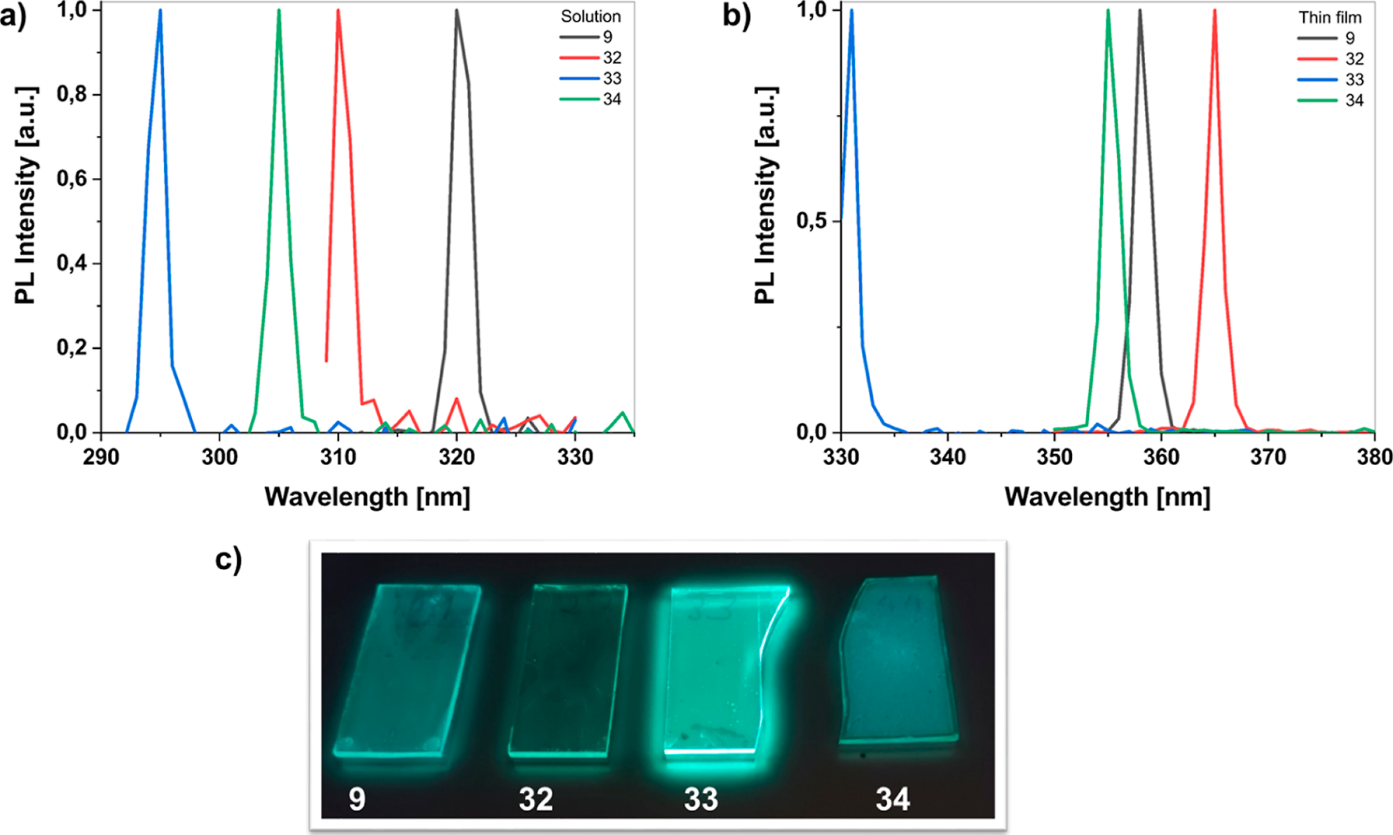
(a) PL spectra of solutions of TPEs **9**, **32**, **33**, and **34** in 1,2-DCB; (b) PL spectra of spin-coated thin films of TPEs **9**, **32**, **33**, and **34** under daylight; and (c) images of the spin-coated thin films of TPEs **9**, **32**, **33**, and **34** under UV light at 366 nm.

To understand the effects on electronic and optical properties of the incorporation with indole of the TPE unit, the UV–visible absorption spectrum of TPEs **9**, **32**, **33**, and **34** was recorded both in solution and in spin-coated thin-film forms (Figure 4). The thin films of TPEs **9**, **32**, **33**, and **34** were prepared by the spin casting technique on pre-cleaned glass substrates with 1000 rpm spin rate from solution in 20 mg/mL in 1,2-dichlorobenzene (1,2-DCB) at 50 °C annealing temperature. As seen in Figure 4a, the absorption peaks of TPE in 1,2-DCB nearly covered the whole visible region, the absorption band maxima were at 327, 310, 300, and 309 nm for the solution of TPEs **32**, **33**, and **34**, respectively. The absorption shoulders of TPEs **32**, **33**, and **34** were at 360, 367, and 362 nm, respectively. The absorption spectrum peaks of TPEs **9**, **32**, **33**, and **34** in thin-film forms were at 352, 352, 340, and 394 nm, respectively (Figure 4b). The compounds showed nearly a bathochromic shift of 25 nm (TPE **9**), 42 nm (TPE **32**), 40 nm (TPE **33**), and 80 nm (TPE **34**), which might be attributed to the presence of intermolecular interactions in the 2D solid state. Comparing the spectra of a spin-coated thin film of TPEs in Figure 5b, TPE **9** has the maximum absorption characteristics, while TPE **32** has the lowest. This difference might be ascribed to the intramolecular charge transfer (ICT) between the indole substituent and TPE unit of TPE **32**. In addition, large differences were observed in the absorption curves of the compounds. The TPEs **9**, **32**, and **34** have a broader absorption curve, while the TPE **33** showed a Gaussian-like shape with a more specific peak wavelength. The absorption values in the thin-film state of the TPEs **9**, **32**, **33**, and **34** were lower than the solution forms. These low photon absorptions could be attributed to the restricted rotations of the aryl rings in 2D thin-film states. Finally, the optical band gaps were calculated from the UV–vis absorption edge to be 3.11, 3.11, 2.91, and 2.61 eV for TPEs **9**, **32**, **33**, and **34**, respectively (Figure 4).

To further investigate the origin of the absorption properties, we characterized the PL emission spectra of TPEs **9**, **32**, **33**, and **34** in solution and as thin films. Figure 5 shows the PL spectra of TPEs **9**, **32**, **33**, and **34** both solutions form in 1,2-DCB and as a spin-coated thin film on a glass substrate. In solution, the PL emissions of TPEs **9**, **32**, **33**, and **34** were 320, 310, 295, and 305 nm, respectively (Figure 5a). In the spin-casted thin films, the PL emissions of TPEs **9**, **32**, **33**, and **34** were 357, 365, 330, and 355 nm, respectively (Figure 5b). Upon transitioning from solution to thin films, red-shifts (30–50 nm) in the PL spectra were observed. The results showed that the fluorescence emission red-shifts from solution to thin films were presumably on account of the aggregation formation and the occurrence of the intermolecular interaction in the solid state. We furthermore analyzed the Stokes shifts of these compounds. In fact, in comparison between solution and thin-film emission spectra, there have been found differences in their maximum emission wavelength because the emission process was dependent on the environment of the molecules. 1,2-DCB has a solvent polarity index of 2.7. For this reason, the observed Stokes shifts for TPEs **9**, **32**, **33**, and **34** were not attributed to the solvent polarity, but these shifts could be related to ICT. The TPE **33** has emitted intense purple-blue fluorescence with the emission peak at 330 nm in the solid state. The emission maximum of TPE **33** was at 295 nm in 1,2-DCB with a 35 nm Stokes shift. Among the TPE derivatives, compounds **9**, **32**, and **34** showed the largest Stokes shifts in the solid state, while compound **34** showed the smallest Stokes shift in the solid state. However, TPEs **9**, **32**, **33**, and **34** displayed higher fluorescence emissions in the thin-film states than that in their solution. Therefore, lower fluorescence quantum yields in solution were observed, whereas compounds exhibited higher fluorescence quantum yields in the solid-state form due to the AIE phenomenon. In Figure 5c, the images of the spin-coated thin films of TPEs **9**, **32**, **33**, and **34** under UV light at 366 nm show that TPE **33** had a maximum light output than the others.

The solid-state absolute PL quantum yields (PLQYs) of TPEs **9**, **32**, **33**, and **34** varied over a much wider range (35–0.30%) (Table 2). The fluorescence quantum yields of **9**, **32**, **33**, and **34** were 35, 28.1, 25.8, and 0.30%, respectively. In particular, TPE **9** exhibited the largest PLQY (35%), whereas the value of TPE **34** was the lowest (0.30%). The order of decrease in the solid-state quantum efficiencies (**9** > **32** > **33** > **34**) was most likely correlated with the increase in indole substituents on the TPE backbone. These decreases could be mainly due to the ICT effect of the electron-rich indole rings. Chromaticity coordinates for TPEs **9**, **32**, **33**, and **34** were found to be (0.169, 0.259), (0.170, 0.164), (0.160, 0.164), and (0.220, 0.241), respectively, corresponding to the blueish region in CIE gamut, as shown in Table 2.

### Electrochemical Behaviors

**Figure 6:**
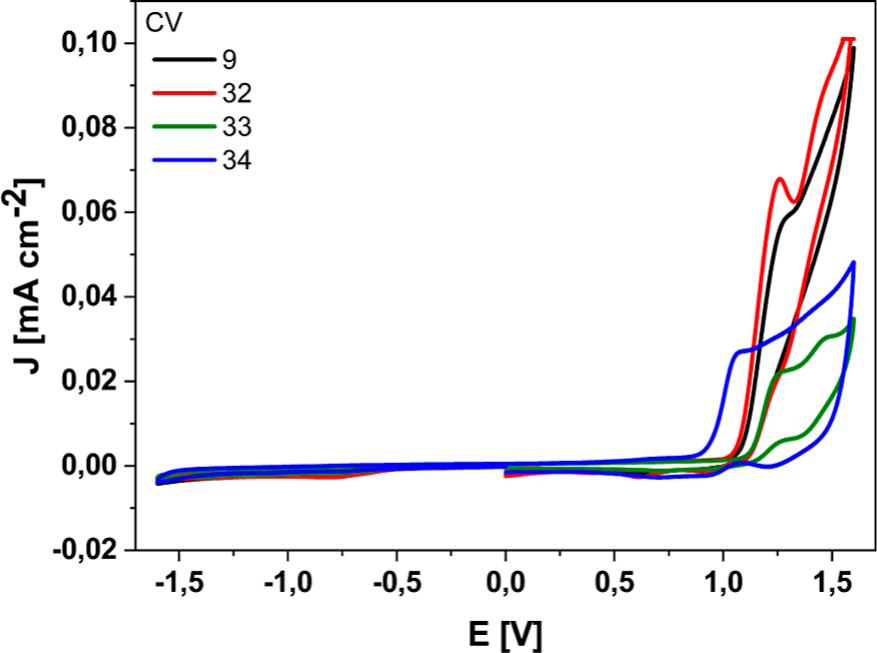
CV curves of TPEs **9**, **32–34** on a glassy carbon as a working electrode in acetonitrile solution with 0.1 M TBAPF_6_ electrolyte. The scanning speed is 100 mV/s.

**Figure 7:**
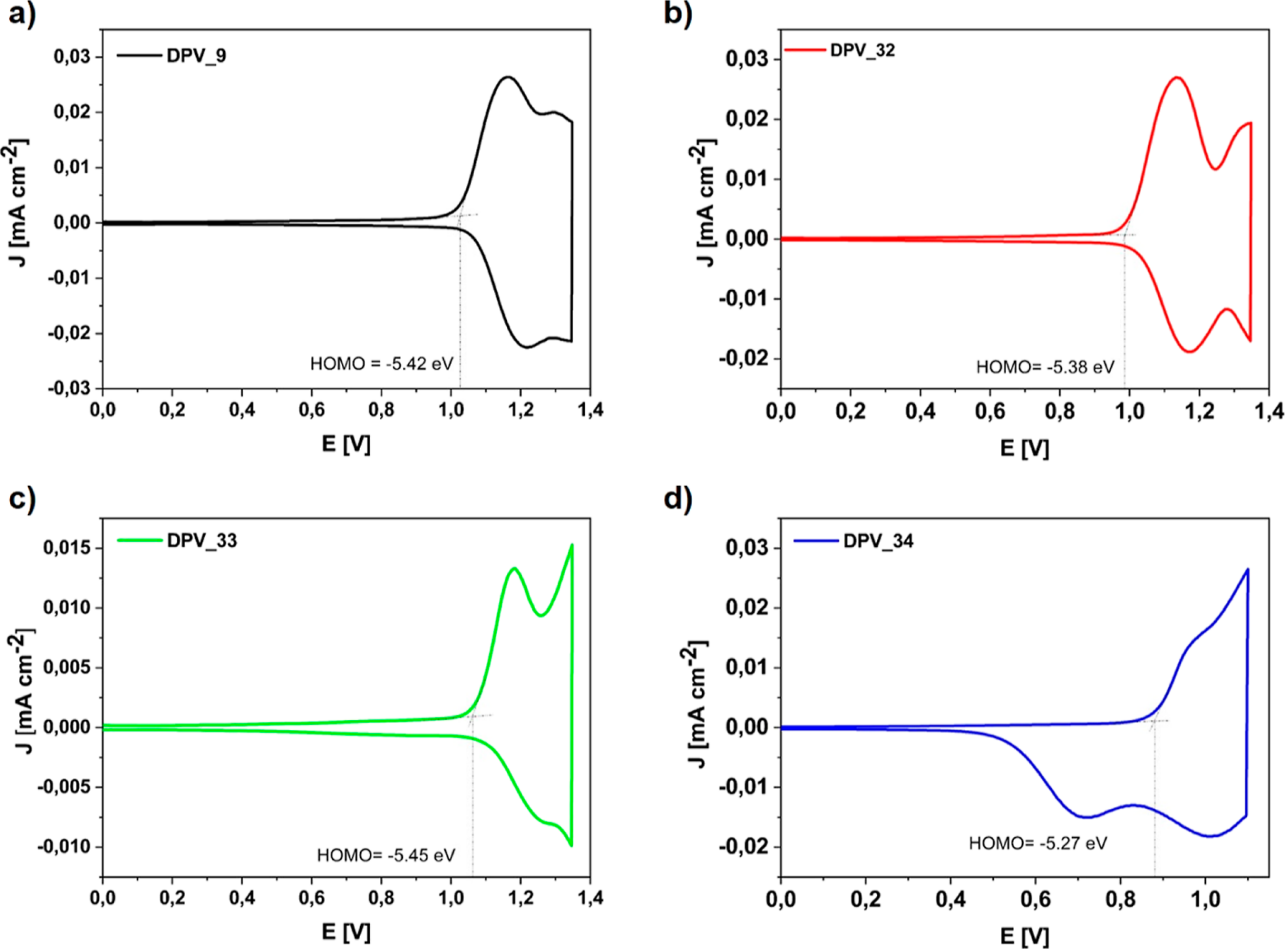
DPV measurements of TPEs (a) **9**, (b) **32**, (c) **33**, and (d) **34**.

To investigate the electrochemical properties of the prepared TPEs **9**, **32**, **33**, and **34** materials, cyclic voltammetry (CV) and differential pulse voltammetry (DPV) were used (Figures 6 and 7). The anodic scan at the CV of TPEs **9**, **32**, **33**, and **34** showed reversible oxidation waves with half-wave potential at about *E*^ox/red^ = 1.09, 1.08, 1.13, and 0.95 V, respectively, which were attributed to oxidation. The oxidation of the TPE **34** was observed in a lower potential than that of the others, which could be attributed to the added extra electron-rich-indole donor moiety on the TPE backbone.

The corresponding highest occupied molecular orbital (HOMO) energy levels were determined from the values of the first onset oxidation potential concerning ferrocene as an external reference. According to the formula of *E*_HOMO_ = −[*E*_onset_^ox^ + 4.8], their HOMO energy levels for TPEs **9**, **32**, **33**, and **34** were determined to be −5.42, −5.38, −5.45, and −5.27 V, respectively. These HOMO energy levels were comparatively lower than the work function (ca. −4.8 eV) of indium tin oxide (ITO) anode, which can significantly decrease the energy barrier between the emission and the hole injection layers (HILs). The lowest unoccupied molecular orbital (LUMO) energy levels were determined from band gaps via the onset of UV–vis absorption spectra. Finally, the LUMO levels of TPEs **9**, **32**, **33**, and **34** were found to be −2.31, −2.27, −2,54, and −2.66 eV, respectively.

### Theoretical Calculations

To probe the molecular and electronic structures, both Frontier molecular orbitals and molecular configuration calculations of TPEs **9**, **32**, **33**, and **34** were conducted via density functional theory (DFT) at the B3LYP with the basis set of 6-311G (d,p).^37^ The four-winged propeller-like molecular structures were linked with different torsion angles between the ethylene core and the adjacent aryl rings (Table 1). The four-winged propeller-like molecular structure was responsible for the separation of the HOMO and LUMO. As seen in Table 1, for TPE **9** and **32**, HOMO-1 electrons were predominantly localized on the indole moieties, while the HOMO and LUMO + 1 electrons were almost delocalized on the entire conjugated backbone. The LUMO of both the compounds was predominantly located on the skeleton outside the indole rings (Table 1). The HOMO – 1 in TPE **33** was located mainly on the indole units, whereas HOMO and LUMO were distributed throughout the TPE skeleton, and LUMO + 1 was dispersed over indole-substituted phenyl rings. As shown in Table 1, the HOMO – 1 of TPE **34** was localized on the two indoles angled by 180° concerning each other, while the HOMO was localized on the central double bond. For TPE **34**, LUMO was located mainly on the central TPE core, while LUMO + 1 was dispersed over the TPE core, with minor distributions on the two indole rings (Table 1). The theoretically calculated HOMO energy levels were well-matched with experimental CV measurements, while theoretically calculated LUMO energy levels are less negative than those experimentally calculated (∼1.0 eV). The distinct HOMO–LUMO charge separations in the four-winged propeller-shaped molecules confirmed the better ICT characteristics. Clearly, the LUMO energy levels calculated by DFT are not in good agreement with the experimental value (∼1.0 eV). We consider that these results are associated with B3LYP functional attributed to basis set convergence.^38^

**Table 1:**
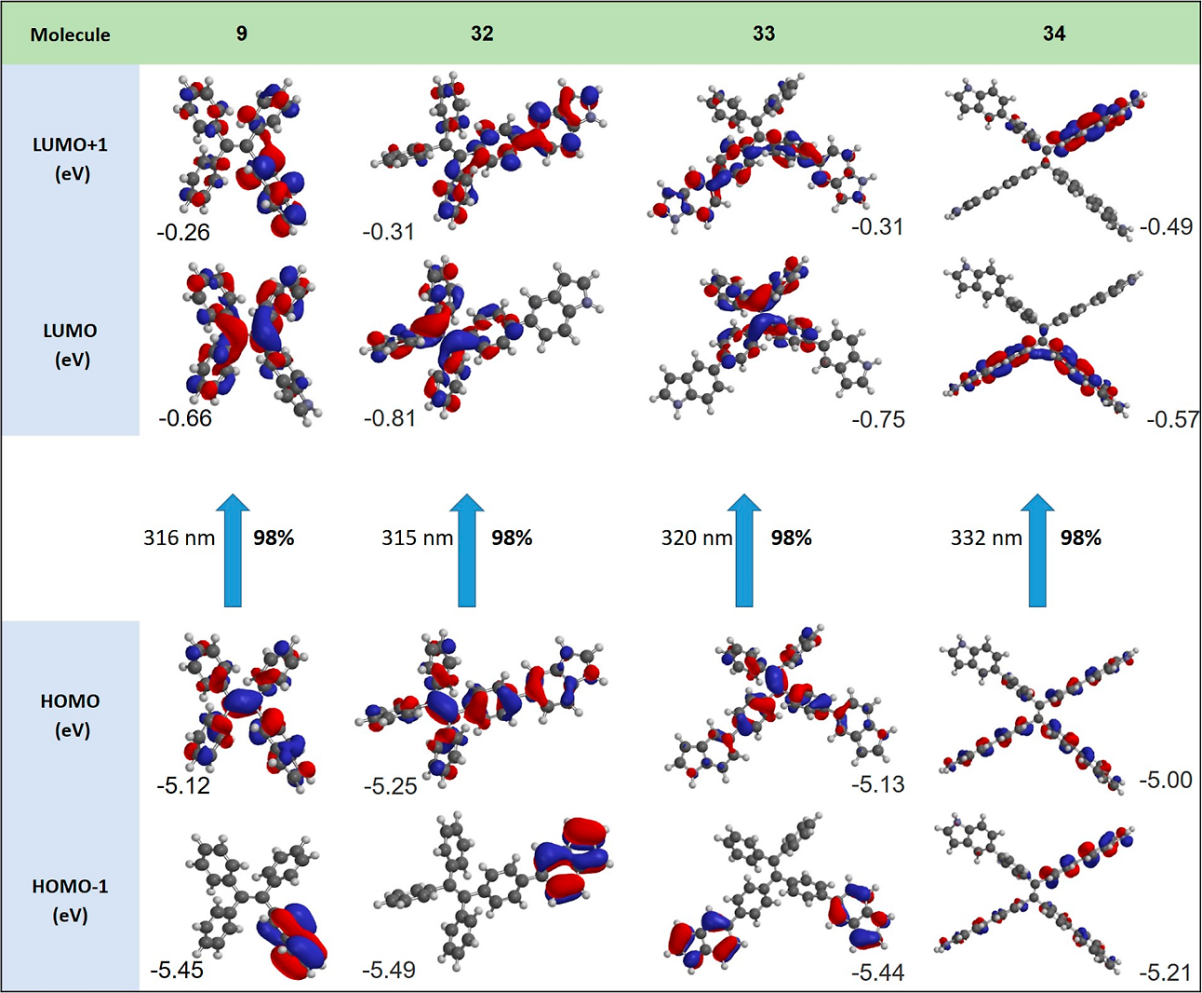
HOMO and LUMO Energy Levels Based on DFT Calculations at the B3LYP/6-31G(d,p) Level of TPEs **9**, **32**, **33**, and **34**

### OLED Device Characterization

**Figure 8:**
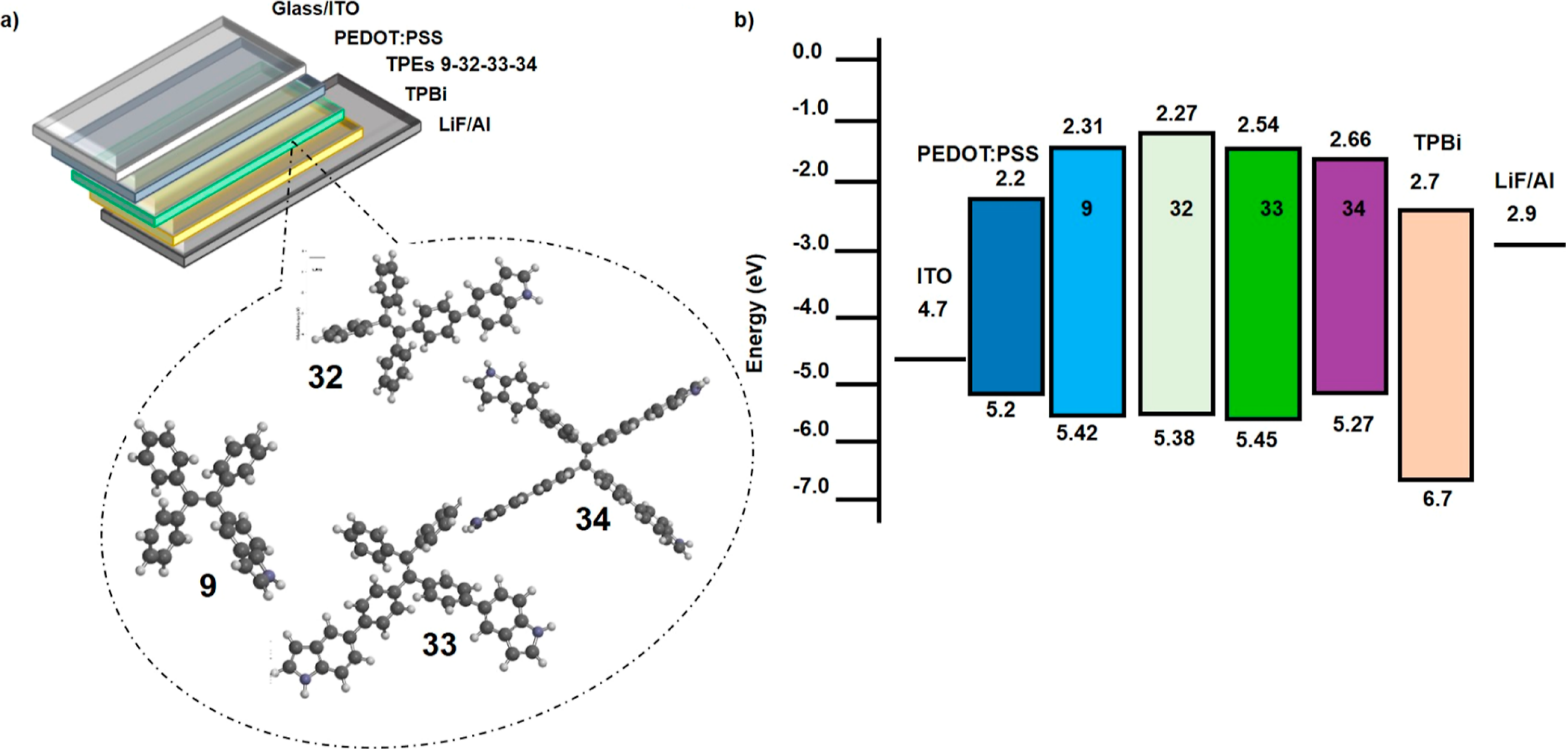
(a) 3D schematic structure of the devices and optimized structures of TPEs **9**, **32**, **33**, and **34** and (b) schematic energy-level diagram for the component materials.

To assess the EL performance, materials **9**, **32**, **33**, and **34** were used as the EML in the fabrication of conventional OLEDs. Compounds **9**, **32**, and **33** had higher PLQY than **34**. The schematic of device structures and energy-level diagrams of OLEDs using TPEs **9**, **32**, **33**, and **34** as the EML are depicted in Figure 8a,b, respectively. The HOMO–LUMO levels in compounds matched reasonably well with those of poly(3,4-ethylenedioxythiophene)/poly(styrene sulfonate) (PEDOT/PSS) (−5.2 eV) as the HIL to transport holes from anode contact to the EML (Table 1 and Figure 8b). The LUMO energy level (∼−3.0 eV) of compounds was coherent with 1,3,5-tris(1-phenyl-1*H*-benzimidazol-2-yl)benzene (TPBi) as electron injection layer (EIL) to inject electrons from the EIL to the EML due to its outstanding electron transporting and hole blocking capabilities with very deep HOMO energy level. The film-forming ability of compound **34** was not good due to the inhomogeneity and roughness of its thin film. This was apparent evidence that the high tendency for crystallization to take a role as the solution-processed EML in the device. For compound **34**, the worst film-forming ability could be attributed to the following two reasons: (1) not having strong π–π stacking interactions between compound **34** and the thiophene rings on PEDOT/PSS and (2) formation of aggregates or crystallines.

**Figure 9:**
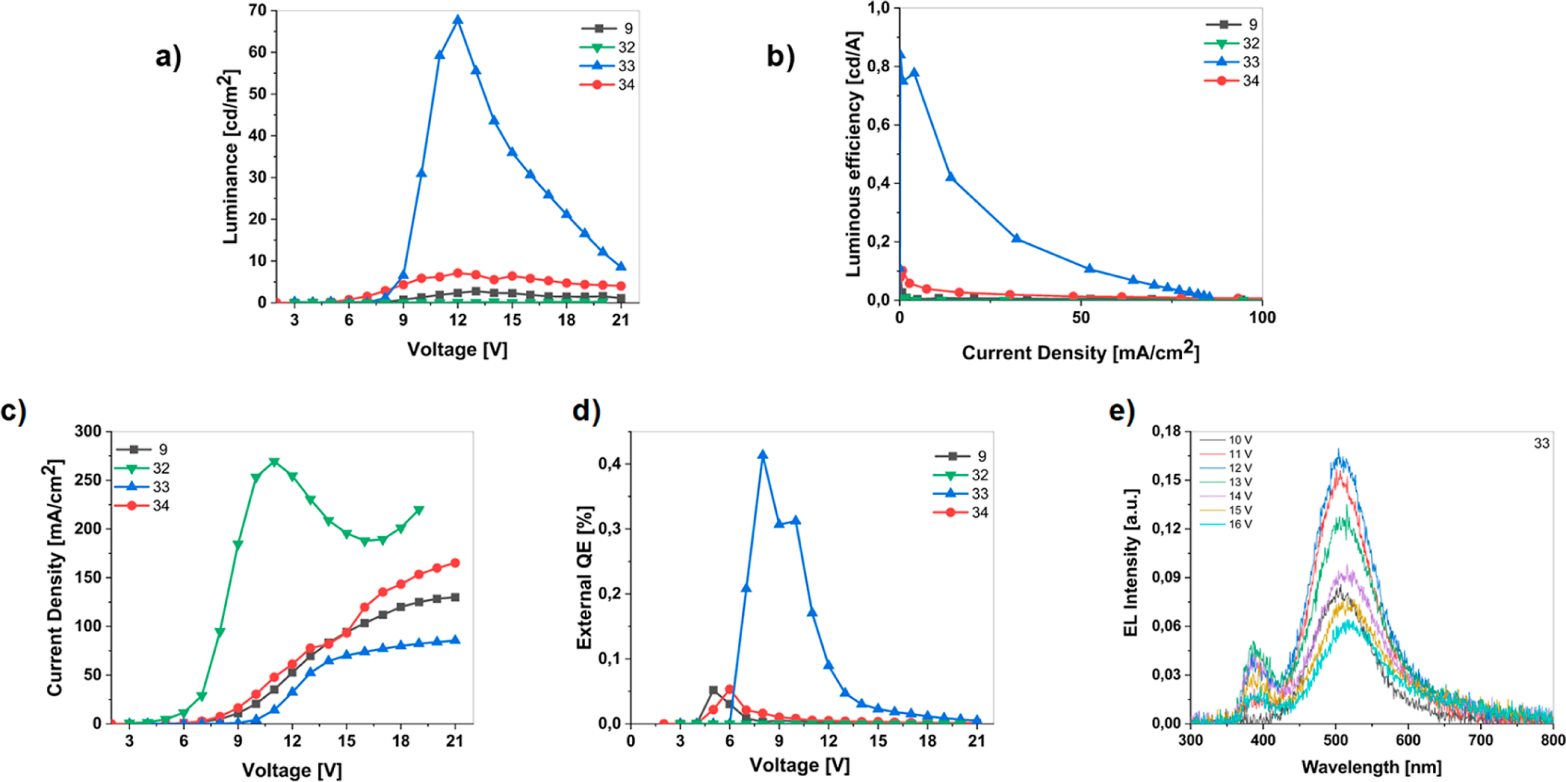
(a) Luminance–voltage; (b) luminous (current) efficiency–current density; (c) current density–voltage; (d) EQE–voltage, and (e) EL spectra of a **33**-based device (10–16 V).

The fabricated OLEDs were of the architecture of ITO/PEDOT: PSS (70 nm)/TPEs **9**, **32**, **33**, and **34** (50 nm)/TPBi (40 nm)/LiF (0.5 nm)/Al (110 nm) (Figure 8a). The luminance–voltage–current density, external quantum efficiency, and EL intensity plot of the conventional OLEDs fabricated using these compounds are illustrated in Figure 9a–e, and the light output performances of devices are summarized in Table 2. Figure 9a shows the luminance–voltage (*L*–*V*) characteristics measured for each conventional OLED device. OLED with TPE **32** had the highest luminance with 67.6 cd/m^2^ at 12 V than others **9**, **32**, **33**, and **34** (Figure 9a). While OLED devices with TPEs **9**, **32**, and **34** showed poor performances, the OLED with TPE **33** exhibited a bright blue light emission with 0.84 cd/A current efficiencies (Figure 9b) and higher external quantum efficiency (0.41%) (Figure 9d). Figure 9c displays the measured current density–voltage (*J*–*V*) characteristics of OLED devices with TPEs **9**, **32**, **33**, and **34**. While the OLED device with **32** has no turn-on voltage (voltage when the luminance of 1 cd/m^2^) due to poor luminance, turn-on voltages of OLED devices with **9**, **33**, and **34** are 9.45, 7.80, and 6.30 V, respectively (Figure 9c). Although the HOMO–LUMO levels in compounds and adjacent PEDOT/PSS match reasonably well, the turn-on voltage and operating voltage of OLEDs with TPEs **9**, **33**, and **34** were found high. The turn-on voltage of **9** was higher than that of devices **33** and **34**. It could be attributed to the differences in thin-film morphologies in the devices. More importantly, the TPE **33**-based OLED device emitted bluish-purple fluorescence with maximum EL emission at 503 nm (Figure 9e), associated with color coordinates of (0.26 and 0.43) in green color (Table 2). The ratio of the peak/shoulder intensity was four; therefore, the blue color was dominant as a light output emission of the OLED device. Among the TPEs studied, the performance of the OLED of TPE **33** is significantly higher than that of other AIE materials. This may be attributed to its better thin-film-forming ability and fine solubility than the others.

**Table 2:**
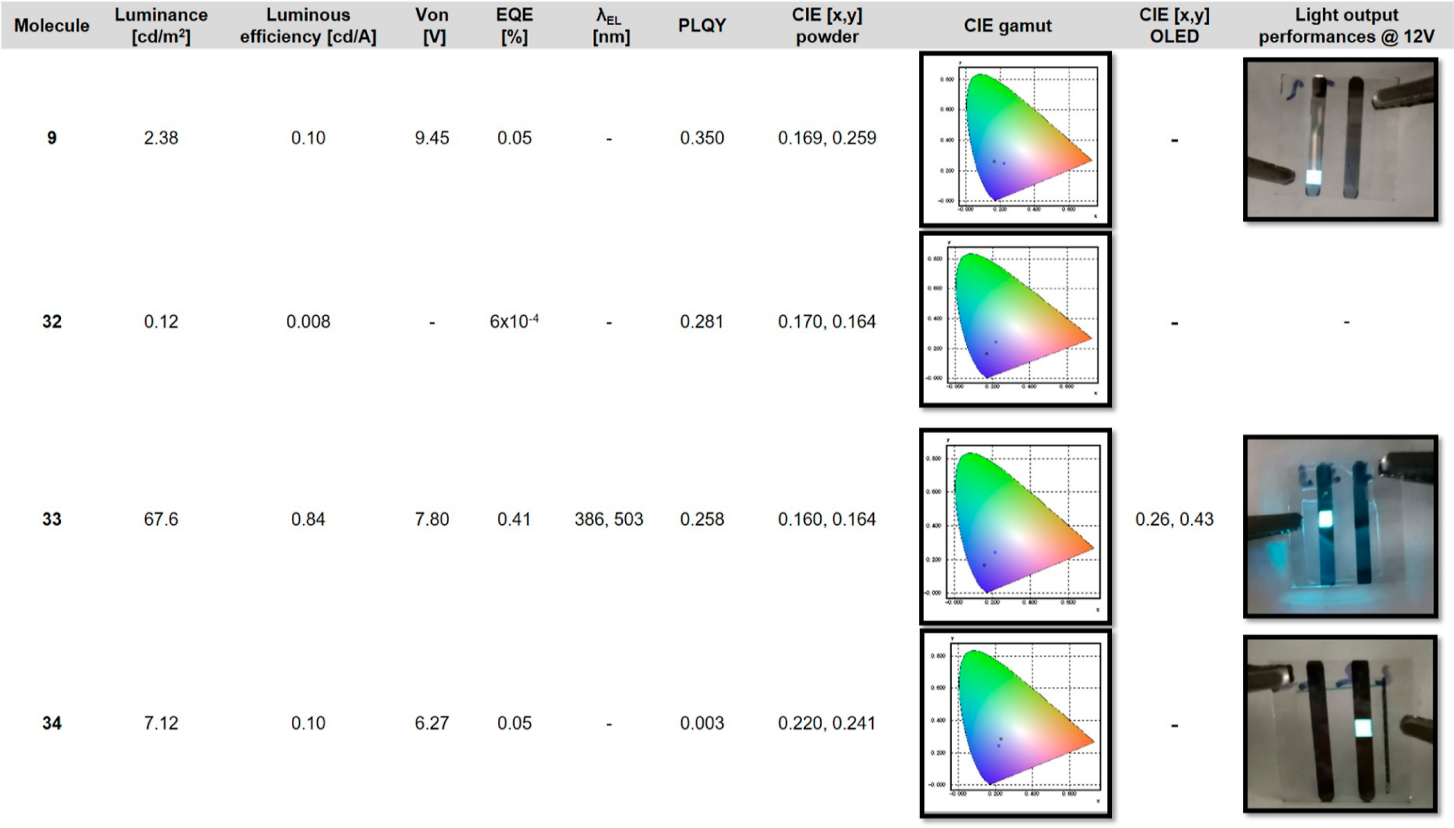
Summary of the Electroluminescent Performance of the Conventional OLED Devices Utilizing TPEs **9**, **32**, **33**, and **34**

### Electrical Properties and Impedance Spectroscopy Characteristics

**Figure 10:**
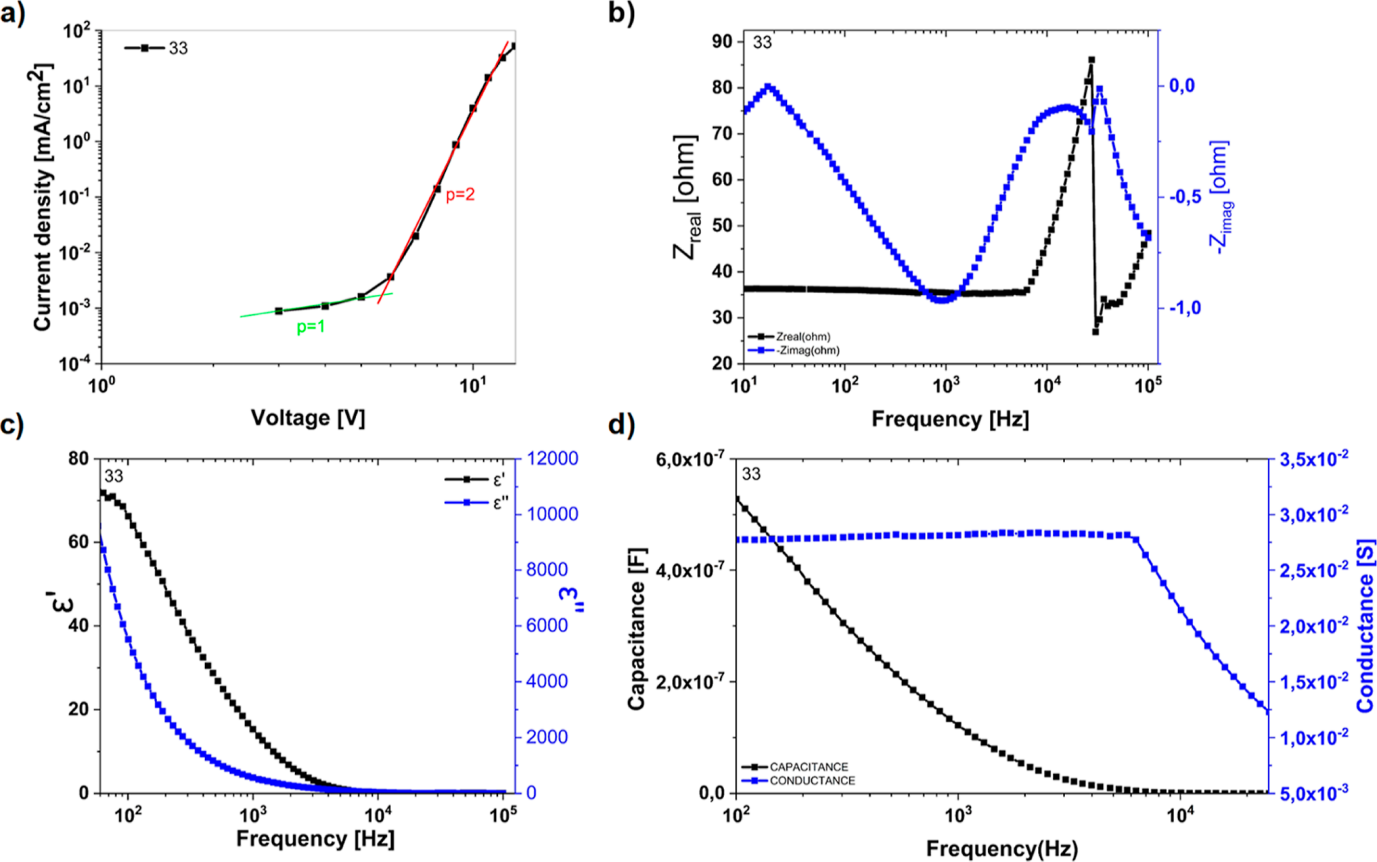
(a) Logarithmic current density–voltage; (b) *Z*_real_ and *Z*_imaginary_ vs frequency; (c) dielectric constant–frequency; and (d) capacitance and conductance vs frequency.

Since the OLED device of **33** had the best performance, its thin film employed electrochemical impedance spectroscopy (EIS) to investigate charge transport properties (Figure 10). Devices have shown typical diode behavior. The current–density characteristics in log–log plots of the [ITO/TPE **33**/Al] device are presented in Figure 10a. The characteristics showed two different regions according to the power law, . At low voltages (*p* = 1), the ohmic relation meant that the current density depended linearly on the voltage.^39^ In eq 1, μ is the charge carrier mobility, *q* is the charge of the electron, *p*_O_ is the free carrier density, and *d* is the organic layer thickness
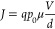
1

The space charge limited current (SCLC) region was observed at a higher applied voltage, where the current density *J* depends quadratically on the applied voltage (*p* = 2).^40^ In eq 2,  is the permittivity of vacuum and  is the relative permittivity of the organic layer
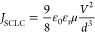
2

The charge mobility and carrier density in the device were calculated from the intersection of logarithmic *J* (V) and *J*_SCLC_ (V) characteristics. From the *J*–*V* characteristics of the [ITO/TPE **33**/Al] device, charge carrier mobility, , carrier density, , and dielectric constant, , could be found. Real permittivity, also called dielectric constant, is usually variable with frequency, although it is always specified at an AC frequency of 100 Hz and temperature conditions of 25 °C.

Impedance spectroscopy is a powerful method to obtain the charge transport and understand the conduction mechanism of the optoelectronic devices.^41^ Therefore, this method was used to determine the dielectric features of the device [ITO/TPE **33**/Al]. The behavior of the reel (*Z*_real_) and imaginary (*Z*_imag_) part with the frequency was investigated for a device with TPE **33** (Figure 10b). These *Z*_real_–*f* measurements showed the frequency-independent behavior of the *Z*_real_ up to 10^4^ Hz. The plateau regime can be seen in the *Z*_real_–*f* graph; this plateau was corresponding to the resistance of charge migration decreasing with increasing frequency. The maximum value of *Z*_real_ was related to the resistances of the ITO/TPE **33** contact and organic layer. In the *Z*_imag_–*f* graph, *Z*_imag_ had two maximum peaks that corresponded to the relaxation frequency (*f*_0_) and time (τ_0_) showing the (τ_0_ = 1/2π *f*_0_) presence of two relaxation processes in the system.^42^ τ_0_ values 9.1 ms and 4.65 μs indicated a dipolar relaxation type.^43^ The real ε′ and the imaginary ε″ parts of the dielectric constants were dependent on the frequency [ε*(ω) = ε′(ω) – ε″(ω)]. The frequency-dependent dielectric constants are given in Figure 10c.
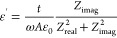
3a
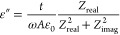
3b

The dielectric constants for a device with TPE **33** were calculated by using eq 3a, where *A* is the active area of the device, *t* is the thickness of the thin film, and ε_0_ is the permittivity of free space. As seen in Figure 10b, the dielectric constant decreased with the increased frequency due to a decrease in the electrical polarization of the space charge carriers, and generated dipoles were insufficient to comply with a variation of the applied AC electric field.

As seen in Figure 10c, the dielectric constant decreased with the increased frequency which was a fundamental characteristic of dielectric materials. At low frequencies, the space charge polarization was dominant and hence the dielectric constant was high. This type of behavior can be easily understood by the hopping phenomenon of electrons and space charge polarization.^44^ The dielectric constant of any material is due to the dipolar, electronic, ionic, and interfacial polarization. At low frequencies, dipolar and interfacial polarizations are responsible for the dielectric behavior of the material. However, at higher frequencies, electronic polarization is responsible for the dielectric, and the contribution of dipolar polarization becomes insignificant. The decrease in dielectric constant with increased frequency could be explained based on the dipole relaxation phenomenon.^45^

Figure 10d shows the dependence of the capacitance and conductivity on frequency for the [ITO/TPE **33**/Al] devices. At low frequencies, the σ was DC conductance and remained constant (ω → 0); however, a decline of the conductivity was detected at a critical frequency (*f* = 34 KHz) which was the second relaxation zone in Figure 10b. A plateau region at low frequencies corresponds to σ_DC_ However, this behavior was opposite of the Jonscher universal dynamic response,^46^ in which the frequency–conductivity relation can be expressed using eq 3b
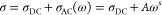
4where σ_DC_ is the dc, σ_AC_ is the ac conductivity, ω is the angular frequency, *s* is the exponent (0 ≤ *s* <≤1), and *A* is the dispersion parameter that determines the strength of polarizability. At the *f* = 34 kHz, the charge carriers transport from site to site, after this frequency, it could be the heat that caused the decrease in the conductivity, dependent on the capacitive reactance of TPE **33**. Also, in eq 4, ε_0_ = constant permittivity of free; ε′ = real part of dielectric constant, and tan δ = loss tangent or dielectric loss.
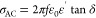
5

In this eq 4, there was a possibility that the product of these quantities might decrease with frequency. Then, σ_AC_ might decrease with frequency.

## Conclusions

In summary, a series of indole-modified and indole-substituted TPE derivatives have been designed and readily synthesized. The photophysical properties of novel TPEs have been studied in detail. Results showed that the molecules are all typical AIE luminogens (AIEgen). Apart from this, two of these AIEgens have been converted to BIM derivatives as a series of new AIEgens via the indole ring. We have utilized AIEgen molecules to fabricate conventional OLED devices, investigate the optoelectronic properties of the indole-modified and indole-substituted TPE derivatives in OLEDs, and obtain efficient blue color, which is still a great important display and lighting area. TPE **33** served as a blue emitter in a solution-processed OLED device. TPEs **9**, **33**, and **34** as an EML in OLED devices had a good film-forming ability. In addition, the energy levels of molecules matched with the HIL and EIL. Moreover, a detailed impedance analysis and calculation of impedance, capacitance, and dielectric constants change with the frequency of TPEs **9**, **32**, **33**, and **34** were performed. A detailed mathematical computation procedure was associated with voltage, current, capacitance, impedance, charge carrier mobility, and charge density. The TPE **33**-based conventional OLED device has the highest luminance with 67.6 cd/m^2^ with ITO/PEDOT: PSS/TPEs/TPBi/LiF/Al. While OLED devices with TPEs **9**, **32**, and **34** showed poor performances, the OLED with TPE **33** exhibited a bright blue light emission with 0.84 cd/A current efficiencies and higher external quantum efficiency (0.41%). Therefore, to understand the charge carrier properties of TPE **33**, the ITO/TPE **33**/Al device was also fabricated for impedance analysis.

## Supporting Information Available

Experimental procedures, NMR and HRMS spectra, and other characterization details (PDF)
