# Supplementary material for: Four-Winged Propeller-Shaped Indole-Modified and Indole-Substituted Tetraphenylethylenes: Greenish-Blue Emitters with Aggregation-Induced Emission Features for Conventional Organic Light-Emitting Diodes
Source: ACS Omega. 2022 Nov 17;7(48):44322–37. doi: 10.1021/acsomega.2c05914 (PMC9730769; doi:10.1021/acsomega.2c05914)
Supplement: Supplementary file 1 — ao2c05914_si_001.pdf [file ao2c05914_si_001.pdf]

Supporting Information

Four-Winged Propeller-Shaped Indole-Modified and Indole Substituted Tetraphenylethylenes: Greenish-Blue Emitters with Aggregation-Induced Emission Features for Conventional Organic Light-Emitting Diodes

Ferruh Lafzi,[†] Yunus Taskesenligil,[†] Betül Canımkurbey,[‡] Selin Pıravadılı,[*,§] Haydar Kilic,[†] Nurullah Saracoglu[*,†]

[†]Department of Chemistry, Faculty of Sciences, Atatürk University, Erzurum, 25240, Türkiye

[‡]Sabuncuoglu Serefeddin Health Services Vocational School, Amasya University, Amasya, 05100, Türkiye

[§]Materials Technologies, Marmara Research Center (MAM), The Scientific and Technological Research Council of Turkey (TUBITAK), Gebze, Kocaeli, 41470, Türkiye

Table of Contents

1.	**Synthesis**	**S3-S10**
2.	**NMR Spectra of Compounds**	**S11-S48**
3.	**HRMS Spectra**	**S49-S57**
4.	**AIE Characteristics**	**S58-S80**
5.	**Theoretical Calculations**	**S81-S99**
6.	**Electrochemical Measurements**	**S99-S100**
7.	**OLED Application**	**S100**

1. Synthesis

General Information. ^1H NMR and ^{13}C NMR experiments were performed on either 400 MHz Varian and 400 MHz Bruker Avance II instruments using $CDCl_3$, DMSO-d_6, MeOD, and Acetone-d_6 as the solvent with tetramethylsilane (TMS) as an internal standard at room temperature, and the coupling constants *J* are given in hertz. The multiplicity is designated as s = singlet, bs = broad singlet, d = doublet, t = triplet, q = quartet, dd = doublet of doublets, m = multiplet. High-resolution mass spectrometry (HRMS) of all compounds was recorded on a QTOF (Quadrupole time-of-flight) spectrometry device. Column chromatography was performed using silica gel pore size 60 Å, 70-230 mesh (sigma).

General procedure for Suzuki coupling = GP1

To a stirred solution of bromo derivative (1 equiv.) and boronic acid or ester derivative (1.1 equiv.) in toluene (40 mL) and EtOH/H_2O (4:4 mL) was added Na_2CO_3 (10 equiv.) and then the system was purged with nitrogen several times. Then $Pd(PPh_3)_4$ (0.02 equiv.) was added and the reaction mixture was stirred at 85 °C for 24 h. After cooling to room temperature, the reaction mixture was mixed with water and extracted with dichloromethane (3 × 40 mL) and the organic layers were combined and dried over Na_2SO_4. After the solvent was removed under reduced pressure, the residue was purified by column chromatography (EtOAc/hexane) to give the desired compound.

General procedure for alkylation = GP2

To a solution of *N*-H or substituted indoles (1 equiv.) in DMSO (5 mL) sodium hydroxide (1.2 equiv.) was added and stirred for 20-30 minutes at room temperature. Then the flask was placed in an ice bath and alkyl halide (1.2 equiv.) was added slowly for 10 minutes. Removed the ice bath and stirred the reaction mixture for 5 h at room temperature. After completion, the reaction mixture was poured into ice water. Extracted the organic product with ethyl acetate (2 × 30 mL) and dried over anhydrous Na_2SO_4. Removed the solvent under vacuum and purified the product with column chromatography (EtOAc/hexane) to give the desired compound.

5-Benzyl-1*H*-indole (2). A reaction tube was charged with ether-imidazolium chloride (8 mg, 0.16 mmol), $Pd_2(dba)_3$ (3.8 mg, 0.04 mmol), and Cs_2CO_3 (540 mg, 1.6 mmol) under nitrogen atmosphere. Then, dioxane (2.0 mL) and H_2O (0.2 mL) were added and the mixture was stirred for 15 min at 80 °C. Benzyl chloride (105 mg, 0.8 mmol) and (1*H*-indol-5-yl)boronic acid (200 mg, 1.24 mmol) were added at room temperature. The reaction mixture was stirred at 90 °C for 15 h. After the reaction mixture was cooled to room temperature water and saturated Na_2CO_3 were added, and then the resulting mixture was extracted with CH_2Cl_2. The combined organic layers were dried over Na_2SO_4. Concentration and purification through silica gel column chromatography (hexane/CH_2Cl_2= 9:1) gave the **2** (160 mg, 93%) as a yellow oil. **^1H NMR (400 MHz, $CDCl_3$):** δ 7.99 (bs, 1H), 7.45 (s, 1H), 7.35 – 7.10 (m, 7H), 7.02 (dd, *J* = 8.3, 1.2 Hz, 1H), 6.47 (d, *J* = 1.9 Hz, 1H), 4.07 (s, CH_2, 2H). **^{13}C NMR (100 MHz, $CDCl_3$):** δ 142.4, 134.5, 132.6, 129.0, 128.4, 128.1, 125.8, 124.4, 123.5, 120.6, 111.0, 102.4, 42.1.

(1*H*-Indol-5-yl)(phenyl)methanone (4). An oven-dried pressure tube equipped with a stir bar was charged with 1-benzoylpiperidine-2,6-dione (200 mg, 0.92 mmol), potassium carbonate (318 mg, 2.3 mmol), boric acid (114 mg, 1.84 mmol), (1*H*-indol-5-yl)boronic acid (178 mg, 1.1 mmol), $Pd(OAc)_2$ (6.2 mg, 0.03 mmol), and $PCyHBF_4$ (41 mg, 0.11 mmol). Tetrahydrofuran (5 mL) was added the reaction mixture was placed in a preheated oil bath at 70 °C for 12h. The reaction mixture was cooled down to room temperature, diluted with CH_2Cl_2 (10 mL), filtered, and concentrated. The residue was purified by column chromatography (EtOAc/hexane = 1:9) to give compound **4** as a white solid (145 mg, 71%, mp 154.5−155.5 °C). **^1H NMR (400 MHz, $CDCl_3$):** δ 8.85 (bs,

1H), 8.14 (s, 1H), 7.91 – 7.73 (m, 3H), 7.64 – 7.53 (m, 1H), 7.54 – 7.38 (m, 3H), 7.29 – 7.24 (m, 1H), 6.63 (s, 1H). **^{13}C NMR (100 MHz, $CDCl_3$):** δ 197.7, 139.0, 138.4, 131.7, 130.0, 129.6, 128.2, 127.2, 125.9, 125.4, 124.2, 111.2, 104.2.

(1*H*-Indol-5-yl)(4-methoxyphenyl)methanone (5). The same procedure described for the synthesis of **4** was used. Column chromatography gave the product **5** as a white-off solid (380 mg, 74% yield; mp 145.5–146.5 °C). **^{1}H NMR (400 MHz, $CDCl_3$):** δ 8.89 (bs, 1H), 8.11 (s, 1H), 7.88 – 7.84 (m, AA′ part of AA′BB′ system, 2H), 7.73 (d, *J* = 8.4 Hz, A part of AB system, 1H), 7.42 (d, *J* = 8.4 Hz, B part of AB system, 1H), 7.33 – 7.24 (m, 1H), 6.99 – 6.94 (m, BB′ part of AA′BB′ system, 2H), 6.63 (s, 1H), 3.88 (s, 3H). **^{13}C NMR (100 MHz, $CDCl_3$):** δ 196.6, 162.7, 138.2, 132.5, 131.4, 130.2, 127.1, 125.8, 124.7, 124.0, 113.4, 111.1, 104.0, 55.5.

(1-Ethyl-1*H*-indol-5-yl)(phenyl)methanone (6). The same procedure described for the synthesis of **4** was used. Column chromatography gave the product **6** as a brown oil (420 mg, 73% yield). **^{1}H NMR (400 MHz, $CDCl_3$):** δ 8.14 (s, 1H), 7.88 – 7.78 (m, 3H), 7.62 – 7.57 (m, 1H), 7.54 – 7.48 (m, 2H), 7.44 (d, *J* = 8.6 Hz, 1H), 7.23 (d, *J* = 3.1 Hz, 1H), 6.62 (d, *J* = 3.1 Hz, 1H), 4.25 (q, *J* = 7.3 Hz, 2H), 1.53 (t, *J* = 7.3 Hz, 3H). **^{13}C NMR (100 MHz, $CDCl_3$):** δ 197.2, 139.2, 138.1, 131.5, 129.9, 129.1, 128.7, 128.1, 127.8, 125.6, 123.7, 109.2, 103.1, 41.3, 15.5.

(1-Ethyl-1*H*-indol-5-yl)(4-methoxyphenyl)methanone (7). The same procedure described for the synthesis of **4** was used. Column chromatography gave product **7** as white-off solid (355 mg, 78% yield; mp 82–83 °C). **^{1}H NMR (400 MHz, $CDCl_3$):** δ 8.11 (s, 1H), 7.88 – 7.85 (m, AA′ part of AA′BB′ system, 2H), 7.78 (d, *J* = 8.6 Hz, A part of AB system, 1H), 7.43 (d, *J* = 8.6 Hz, B part of AB system, 1H), 7.23 (d, *J* = 3.1 Hz, 1H), 7.01 – 6.98 (m, BB′ part of AA′BB′ system, 2H), 6.61 (d, *J* = 3.1 Hz, 1H), 4.25 (q, *J* = 7.3 Hz, 2H), 3.92 (s, 3H), 1.53 (t, *J* = 7.3 Hz, 3H). **^{13}C NMR (100 MHz, $CDCl_3$):** δ 196.1, 162.6, 137.8, 132.4, 131.6, 129.7, 128.5, 127.8, 125.0, 123.6, 113.3, 109.0, 102.9, 55.5, 41.2, 15.5.

5-(1,2,2-Triphenylvinyl)-1*H*-indole (9). A 100 mL two-necked round-bottom flask with a reflux condenser was charged with zinc dust (121 mg, 1.84 mmol) and 15 mL of dry THF. The flask was evacuated under vacuum and pumped in dry nitrogen three times. The mixture was cooled to -78 °C, and $TiCl_4$ (101 µL, 0.9 mmol) was slowly added by a syringe. The mixture was slowly warmed to room temperature, stirred for 0.5 h, and then refluxed for 2 h. Pyridine (37 µl, 0.46 mmol) was added to the mixture and refluxed for an additional 10 min. A solution of benzophenone (109 mg, 0.6 mmol) and (1*H*-indol-5-yl)(phenyl)methanone (102 mg, 0.46 mmol) in THF (10 mL) was added dropwise and refluxed for 18 h. The reaction was warmed to room temperature and quenched with 10% aqueous K_2CO_3 solution and filtrated. The filtration was extracted with dichloromethane three times, and the organic layers were combined and washed with brine twice and dried over Na_2SO_4. The solvent was evaporated under reduced pressure, and the crude product was purified on a silica gel column chromatography (EtOAc/hexane = 1:9) to give compound **9** as a white solid (67 mg, 39%, mp 175.0–176.0 °C). **^{1}H NMR (400 MHz, $CDCl_3$):** δ 7.88 (bs, 1H), 7.22 (s, 1H), 7.16 (s, 1H), 7.06 – 6.91 (m, 16H), 6.82 (dd, *J* = 8.4, 1.4 Hz, 1H), 6.27 (s, 1H). **^{13}C NMR (100 MHz, $CDCl_3$):** δ 144.7 (2C), 144.4 (2C), 142.1, 139.8, 135.6, 134.6, 131.53, 131.50, 131.46, 127.6 (2C), 127.5, 126.2 (2C), 126.1, 126.0, 124.2, 123.8, 110.1, 102.9. **HRMS (APCI-TOF) m/z:** $[M + H]^+$ calcd for $C_{28}H_{22}N$, 372.1752; found 372.1746.

Also, 5-(1,2,2-triphenylvinyl)-1*H*-indole (**9**) was obtained in 70% yield (540 mg) from Suzuki coupling reaction (**GP1**).

5-(1-(4-Methoxyphenyl)-2,2-diphenylvinyl)-1*H*-indole (10). The same procedure described for the synthesis of **9** was used. Column chromatography gave the product **10** as white-off solid (25 mg, 31% yield; mp

181.0–182.0 °C). **^{1}H NMR (400 MHz, $CDCl_3$):** δ 7.98 (bs, 1H), 7.30 (s, 1H), 7.14 – 6.99 (m, 12H), 6.98 – 6.95 (m, AA′ part of AA′BB′ system, 2H), 6.89 (dd, *J* = 8.5, 1.6 Hz, 1H), 6.66 – 6.56 (m, BB′ part of AA′BB′ system, 2H), 6.36 (d, *J* = 2.1 Hz, 1H), 3.72 (s, CH_3, 3H). **^{13}C NMR (100 MHz, $CDCl_3$):** δ 157.9, 144.7, 144.6, 141.6, 138.9, 137.1, 135.8, 134.6, 132.7, 131.5, 131.48, 127.7, 127.5, 127.48, 126.3, 125.9, 125.8, 124.1, 123.9, 112.9, 110.0, 102.9, 55.1. **HRMS (APCI-TOF) m/z:** $[M + H]^+$ calcd for $C_{29}H_{24}NO$, 402.1858; found 402.1843.

Also, 5-(1-(4-methoxyphenyl)-2,2-diphenylvinyl)-1*H*-indole (**10**) was obtained in 84% yield (690 mg) from Suzuki coupling reaction (**GP1**).

(2-Bromoethene-1,1,2-triyl)tribenzene (11). To a solution of ethene-1,1,2-triyltribenzene (1.0 g, 3.49 mmol) in chloroform (15 mL) was added a solution of bromine (0.49 g, 3.06 mmol) in chloroform (10 mL) slowly. After decolouration of the solution, the solvent was evaporated under reduced pressure, and the residue crystallized two times from ethanol to give **11** (0.8 mg, 62%, mp 115.0–116.0 °C) as a white solid. **^{1}H NMR (400 MHz, $CDCl_3$):** δ 7.32 – 7.27 (m, 4H), 7.27 – 7.18 (m, 3H), 7.12 – 7.04 (m, 3H), 7.00 – 6.96 (m, 3H), 6.94 – 6.79 (m, 2H). **^{13}C NMR (100 MHz, $CDCl_3$):** δ 143.8, 143.6, 141.12, 141.08, 130.4, 130.3, 129.5, 128.2, 128.0, 127.96, 127.9, 127.6, 127.0, 122.2.

(2-Bromo-2-(4-methoxyphenyl)ethene-1,1-diyl)dibenzene (12). The same procedure described for the synthesis of **11** was used. Recrystallization from ethanol gave the product as a light-brown solid (1.1 g, 90% yield; mp 124.5–125.5 °C). **^{1}H NMR (400 MHz, $CDCl_3$):** δ 7.41 – 7.15 (m, 8H), 7.15 – 7.04 (m, 2H), 7.01 – 6.90 (m, 2H), 6.70 (d, *J* = 8.8 Hz, 2H), 3.76 (s, 3H). **^{13}C NMR (100 MHz, $CDCl_3$):** δ 159.1, 144.1, 142.7, 141.4, 133.4, 131.8, 130.4, 129.6, 128.2, 127.9, 127.5, 126.9, 122.5, 113.4, 55.2.

5-(4,4,5,5-Tetramethyl-1,3,2-dioxaborolan-2-yl)-1*H*-indole (13). To a solution of 5-bromo-1*H*-indole (1 g, 5.1 mmol) in dioxane were added $Pd_2(dba)_3$ (93 mg, 0.1 mmol), XPhos (2-dicyclohexylphosphino-2′,4′,6′-triisopropylbiphenyl) (97 mg, 0.2 mmol), KOAc (1.5 g, 15.3 mmol) and bis(pinacolato)diboron (1.42 g, 5.61 mmol). The reaction mixture was stirred on a pressure tube at 110 °C for 20 hours, then was cooled at room temperature, filtrate over Celite, and washed with ethyl acetate. To the reaction mixture was added water. The layers were separated and the aqueous phase was extracted with ethyl acetate (3 x 10). The combined organic phases were washed with brine, dried over sodium sulfate and the solvent evaporated under reduced pressure. The residue was purified by column chromatography (hexane/acetone = 0.3:9.7) to give the product as a white-off solid (1.0 g, 80% yield; mp 93.0–94.0 °C). **^{1}H NMR (400 MHz, $CDCl_3$):** δ 8.18 (bs, 1H), 7.64 (d, *J* = 8.1 Hz, 1H), 7.39 (d, *J* = 8.1 Hz, 1H), 7.26 (s, 1H), 7.21 – 7.18 (m, 1H), 6.58 – 6.55 (m, 1H), 1.37 (s, 12H). **^{13}C NMR (100 MHz, $CDCl_3$)**: δ 137.9, 128.7, 128.1, 127.6, 124.2, 110.5, 103.1, 83.5, 24.9 (carbon adjacent to boron was not observed).

4-(4,4,5,5-1-Ethyl-5-(1,2,2-triphenylvinyl)-1*H*-indole (14). Compound **14** was obtained according to **GP2**. The product was obtained over column chromatography as a white-off solid (250 mg, 69% yield; mp 159.5–160.5 °C). **^{1}H NMR (400 MHz, $CDCl_3$):** δ 7.20 – 7.17 (m, 2H), 7.07 – 6.93 (m, 16H), 6.82 (dd, *J* = 8.6, 1.6 Hz, 1H), 6.22 (d, *J* = 3.0 Hz, 1H), 4.01 (q, *J* = 7.3 Hz, 2H), 1.36 (t, *J* = 7.3 Hz, 3H). **^{13}C NMR (100 MHz, $CDCl_3$):** δ 144.9, 144.5, 144.4, 142.1, 139.5, 134.9, 134.6, 131.6, 131.52, 131.50, 128.2, 127.61, 127.60, 127.5, 126.9, 126.1, 126.04, 126.0, 125.6, 124.1, 108.3, 101.3, 40.9, 15.4. **HRMS (APCI-TOF) m/z:** $[M + H]^+$ calcd for $C_{30}H_{26}N$, 400.2065; found 400.2067.

1-Ethyl-5-(1-(4-methoxyphenyl)-2,2-diphenylvinyl)-1*H*-indole (15). Compound **15** was obtained according to **GP2**. The product was obtained over column chromatography as a yellow solid (485 mg, 82% yield; mp 1151.3–

152.3 °C). **^{1}H NMR (400 MHz, $CDCl_3$):** δ 7.31 – 7.27 (m, 2H), 7.19 – 7.03 (m, 11H), 7.01 – 6.97 (m, AA′ part of AA′BB′ system, 2H), 6.93 (d, *J* = 8.4 Hz, 1H), 6.66 – 6.62 (m, BB′ part of AA′BB′ system, 2H), 6.32 (s, 1H), 4.11 (q, *J* = 7.3 Hz, 2H), 3.76 (s, 3H), 1.46 (t, *J* = 7.3 Hz, 3H). **^{13}C NMR (100 MHz, $CDCl_3$):** δ 157.9, 144.8, 144.7, 141.7, 138.7, 137.3, 135.2, 134.6, 132.8, 131.53, 131.50, 128.1, 127.7, 127.6, 126.9, 125.9, 125.8, 125.7, 124.2, 112.9, 108.3, 101.3, 55.1, 40.9, 15.4. **HRMS (APCI-TOF) m/z:** $[M + H]^+$ calcd for $C_{31}H_{28}NO$, 430.2171; found 430.2158.

1-(2,2-Dibromovinyl)-4-nitrobenzene (17). To a solution of 4-nitrobenzaldehyde (1.0 g, 6.62 mmol) in dichloromethane (20 mL) was added CBr_4 (3.29 g, 9.93 mmol) and cooled to 0 ºC. Then a solution of PPh_3 (5.21 g, 19.85 mmol) in dichloromethane (10 mL) was added dropwise to the reaction mixture and stirred for 2 h at room temperature. After completion, the reaction mixture was insoluble material was removed by filtration. The solvent evaporated under reduced pressure. The residue was purified by column chromatography (hexane/ EtOAc = 9.5:0.5) to give the product as a yellow solid (1.75 g, 86% yield; mp 104.0–105.0 °C). **^{1}H NMR (400 MHz, $CDCl_3$):** δ 8.29 – 8.23 (m, AA′ part of AA′BB′ system, 2H), 7.76 – 7.69 (m, BB′ part of AA′BB′ system, 2H), 7.58 (s, 1H). **^{13}C NMR (100 MHz, $CDCl_3$):** δ 141.5, 134.9, 129.2, 123.7, 94.1 (2C).

(2-(4-Nitrophenyl)ethene-1,1-diyl)dibenzene (18). Compound **18** was obtained according to **GP1**. Column chromatography gave the product as yellow solid (400 mg, 81% yield; mp 154.3–155.3 °C). **^{1}H NMR (400 MHz, $CDCl_3$):** δ 8.00 (d, *J* = 8.8 Hz, 2H), 7.41 – 7.34 (m, 8H), 7.23 – 7.12 (m, 4H), 7.02 (s, 1H). **^{13}C NMR (100 MHz, $CDCl_3$):** δ 147.0, 145.9, 144.3, 142.4, 139.3, 130.2, 130.0, 129.0, 128.5, 128.4, 128.3, 127.9, 125.8, 123.3.

(2-Bromo-2-(4-nitrophenyl)ethene-1,1-diyl)dibenzene (19). The same procedure described for the synthesis of **11** was used. Recrystallization from ethanol gave the product as a light yellow solid (228 mg, 90% yield; mp 178.0–179.5 °C). **^{1}H NMR (400 MHz, $CDCl_3$):** δ 8.06 (dd, *J* = 8.6, 1.5 Hz, 2H), 7.52 – 7.47 (m, 2H), 7.46 – 7.35 (m, 5H), 7.22 – 7.09 (m, 3H), 7.01 – 6.94 (m, 2H). **^{13}C NMR (100 MHz, $CDCl_3$):** δ 147.8, 146.8, 146.5, 142.8, 140.3, 131.3, 130.3, 129.4, 128.3 (2C), 128.1, 127.9, 123.3, 118.5. **HRMS (ESI-TOF) m/z:** $[M + H]^+$ calcd for $C_{20}H_{15}BrNO_2$, 380.0281; found 380.0283.

1-Ethyl-5-(1-(4-nitrophenyl)-2,2-diphenylvinyl)-1*H*-indole (21). Compound **21** was obtained according to **GP1**. Column chromatography gave the product as yellow solid (300 mg, 85% yield; mp 169.0–170.0 °C). **^{1}H NMR (400 MHz, $CDCl_3$):** δ 7.96 (d, *J* = 8.5 Hz, 2H), 7.31 – 7.22 (m, 3H), 7.19 – 7.14 (m, 2H), 7.15 – 7.05 (m, 10H), 6.89 (d, *J* = 8.5 Hz, 1H), 6.35 (d, *J* = 3.0 Hz, 1H), 4.13 (q, *J* = 7.2 Hz, 2H), 1.48 (t, *J* = 7.2 Hz, 3H). **^{13}C NMR (100 MHz, $CDCl_3$):** δ 152.3, 145.8, 143.4 (2C), 142.4, 140.1, 134.8, 133.7, 132.2, 131.4, 131.3, 128.4, 128.0, 127.7, 127.4, 127.0, 126.7, 125.2, 124.1, 122.9, 108.8, 101.4, 41.0, 15.4. **HRMS (APCI-TOF) m/z:** $[M + H]^+$ calcd for $C_{30}H_{25}N_2O_2$, 445.1946; found 445.1954.

4-(1-(1-Ethyl-1*H*-indol-5-yl)-2,2-diphenylvinyl)aniline (22). 1-Ethyl-5-(1-(4-nitrophenyl)-2,2-diphenylvinyl)-1*H*-indole (**21**) (200 mg, 0.4 mmol) was dissolved in MeOH (10 mL), and 10% Pd/C (15 mg) was added. The mixture was stirred in an H_2 atmosphere for 12 h. The Pd/C was removed by filtration and the solvent was evaporated to obtain a pure compound **22** as a yellow solid (178 mg, 95%; mp 191.0–192.0 °C). **^{1}H NMR (400 MHz, $CDCl_3$):** δ 7.34 (s, 1H), 7.19 – 7.03 (m, 12H), 6.95 (dd, *J* = 8.6, 1.6 Hz, 1H), 6.91 – 6.86 (m, AA′ part of AA′BB′ system, 2H), 6.50 – 6.43 (m, BB′ part of AA′BB′ system, 2H), 6.33 (d, *J* = 3.1 Hz, 1H), 4.11 (q, *J* = 7.3 Hz, 2H), 3.68 (bs, 2H), 1.47 (t, *J* = 7.3 Hz, 3H). **^{13}C NMR (100 MHz, $CDCl_3$):** δ 145.1, 144.9, 144.0, 142.1, 138.1, 135.6, 135.3, 134.6, 132.7, 131.6, 131.5, 128.1, 127.6, 127.5, 126.8, 125.8, 125.7, 125.6, 124.2, 114.5, 108.2, 101.3, 40.9, 15.4. **HRMS (APCI-TOF) m/z:** $[M + H]^+$ calcd for $C_{30}H_{27}N_2$, 415.2169; found 415.2143.

1,2-Bis(4-(1-(1-ethyl-1*H*-indol-5-yl)-2,2-diphenylvinyl)phenyl)diazene (23). Solution of 4-(1-(1-Ethyl-1*H*-indol-5-yl)-2,2-diphenylvinyl)aniline (**22**) (510 mg, 0.12 mmol) in toluene (4 mL) was charged to a sealed tube and purged with nitrogen several times. Then *t*-BuOK (40.6 mg, 0.36 mmol), iodobenzene (26 µL, 0.24 mmol), CuI (0.7 mg, 0.03 mmol) and 1,10-Phenanthroline (0.7 mg, 0.03 mmol) were added to mixture and heated to 110 ^{o}C for 20h. After cooling to room temperature, the reaction solution was filtered to remove the precipitated base and washed with toluene. The solution was concentrated to obtain the crude product. Column chromatography (hexane/ EtOAc = 9.8:0.2) gave the product as red solid (70 mg, 70% yield; mp 260.0–261.0 °C). **^{1}H NMR (400 MHz, $CDCl_3$):** δ 7.55 – 7.47 (m, AA′ part of AA′BB′ system, 4H), 7.21 (s, 2H), 7.13 – 7.06 (m, BB′ part of AA′BB′ system, 4H), 7.03 – 6.93 (m, 24H), 6.83 (dd, *J* = 8.6, 1.8 Hz, 2H), 6.22 (d, *J* = 3.1 Hz, 2H), 4.00 (q, *J* = 7.3 Hz, 4H), 1.35 (t, *J* = 7.3 Hz, 6H). **^{13}C NMR (100 MHz, $CDCl_3$):** δ 150.9, 148.0, 144.2 (2C), 141.5, 140.7, 134.8, 134.5, 132.4, 131.6, 131.5, 128.3, 127.8, 127.7, 127.0, 126.4, 126.2, 125.6, 124.3, 122.0, 108.5, 101.4, 40.9, 15.4. **HRMS (APCI-TOF) m/z:** $[M + H]^+$ calcd for $C_{60}H_{49}N_4$, 825.3952; found 825.3970.

(2,2-Dibromoethene-1,1-diyl)dibenzene (24). To a solution of benzophenone (1. 0 g, 5.49 mmol) and CBr4 (3.64 g, 10.98 mmol) in toluene (70 mL) was added PPh_3 (5.76 g, 21.95 mmol). The mixture was heated at 130 ^{o}C in an oil bath for 4 days. After cooling to room temperature, the solvent was removed by rotary evaporator and the residue was extracted with n-hexane. The hexane phases were combined and dried to give a residue, which was separated by column chromatography using n-hexane as the eluent to give the product. Recrystallization from ethanol gave the product as a white solid (1.6 g, 86% yield; mp 87.4–88.4 °C). **^{1}H NMR (400 MHz, $CDCl_3$):** δ 7.31 – 7.19 (m, 10H). **^{13}C NMR (100 MHz, $CDCl_3$):** δ 147.9, 141.4, 128.8, 128.4, 128.0, 90.3.

5-(1-Bromo-2,2-diphenylvinyl)-1*H*-indole (26). Under an argon atmosphere, (2,2-Dibromoethene-1,1-diyl)dibenzene (338 mg, 1.0 mmol), (1*H*-indol-5-yl)boronic acid (169 mg, 1.05 mmol), tri(2-furyl)-phosphine (34.8 mg, 0.15 mmol), and bis(dibenzylideneacetone)palladium (46 mg, 0.05 mmol) were placed in a 50 mL Schlenk tube. THF (5 mL), ether (2.1 mL), and a solution of cesium carbonate (652 mg, 2.0 mmol) in water (2 mL) were then added. The reaction mixture was stirred at reflux for 18 h. The product was extracted with ethyl acetate (3 x 30 mL). The combined organic layers were washed with brine (10 mL) and dried over anhydrous sodium sulfate. Evaporation followed by silica gel column chromatography (hexane:/ethyl acetate = 9.5:0.5) afforded 1d as a white solid (185 mg, 49%; mp 198.7–199.7 °C). **^{1}H NMR (400 MHz, $CDCl_3$):** δ 8.05 (bs, 1H), 7.68 (s, 1H), 7.43 – 7.35 (m, 4H), 7.35 – 7.28 (m, 1H), 7.16 – 7.12 (m, 3H), 7.05 – 6.95(m, 5H), 6.50 – 6.32 (m, 1H). **^{13}C NMR (100 MHz, $CDCl_3$):** δ 144.5, 142.3, 141.6, 135.2, 132.9, 130.4, 129.7, 128.2, 127.8, 127.5, 127.3, 126.6, 124.8, 124.7, 124.4, 123.2, 110.4, 103.3. **HRMS (ESI-TOF) m/z:** $[M + H]^+$ calcd for $C_{22}H_{17}BrN$, 374.0539; found 374.0540.

4-(1-(1*H*-Indol-5-yl)-2,2-diphenylvinyl)-*N,N*-diphenylaniline (28). Compound **28** was obtained according to **GP1**. Column chromatography gave the product as a light brown solid (79 mg, 84% yield; mp 180.4–181.4 °C). **^{1}H NMR (400 MHz, $CDCl_3$):** δ 7.83 (bs, 1H), 7.29 (s, 1H), 7.14 – 7.08 (m, 5H), 7.06 – 6.91 (m, 15H), 6.90 – 6.79 (m, 5H), 6.69 (d, *J* = 8.6 Hz, 2H), 6.31 – 6.28 (m, 1H). **^{13}C NMR (100 MHz, $CDCl_3$):** δ 147.7 (2C), 145.8, 144.7, 144.4, 141.8, 139.4, 139.1, 135.4, 134.6, 132.4, 131.6, 131.5, 129.1, 127.6 (2C), 126.2, 126.1, 125.9, 124.2, 124.2, 123.9, 123.0, 122.6, 110.1, 102.9. **HRMS (ESI-TOF) m/z:** $[M + H]^+$ calcd for $C_{40}H_{31}N_2$, 539.2482; found 539.2480.

4-(1-(1-Ethyl-1*H*-indol-5-yl)-2,2-diphenylvinyl)-*N,N*-diphenylaniline (29). Compound **29** was obtained according to **GP2**. Column chromatography gave the product as yellow solid (60 mg, 95% yield; mp 82.3–83.3 °C). **^{1}H NMR (400 MHz, $CDCl_3$)** δ 7.28 (d, *J* = 1.4 Hz, 1H), 7.17 – 7.11 (m, 5H), 7.08 – 6.94 (m, 15H), 6.91 – 6.80

(m, 5H), 6.71 – 6.69 (m, 2H), 6.26 (d, *J* = 3.1 Hz, 1H), 4.01 (q, *J* = 7.2 Hz, 2H), 1.36 (t, *J* = 7.2 Hz, 3H). **^{13}C NMR (100 MHz, CDCl$_3$)** δ 147.7, 145.7, 144.8, 144.5, 141.9, 139.3, 139.2, 134.7, 134.7, 132.4, 131.6, 131.5, 129.1, 128.2, 127.6, 127.5, 126.9, 126.0, 125.9, 125.6, 124.14, 124.08, 123.0, 122.5, 108.3, 101.3, 40.9, 15.4. **HRMS (ESI-TOF) m/z:** $[M + H]^+$ calcd for $C_{42}H_{35}N_2$, 567.2795; found 567.2775.

5,5'-(2,2-Diphenylethene-1,1-diyl)bis(1*H*-indole) (30). Compound **30** was obtained according to **GP1**. Column chromatography gave the product as a white-off solid (180 mg, 85% yield; mp 295.4–296.4 °C). **^{1}H NMR (400 MHz, Aseton-*d*$_6$)** δ 9.96 (s, 2H), 7.14 (s, 2H), 7.08 (s, 2H), 7.00 (d, *J* = 8.4 Hz, A part of AB system, 2H), 6.97 – 6.81 (m, 10H), 6.73 (d, *J* = 8.4 Hz, B part of AB system, 2H), 6.13 (s, 2H). **^{13}C NMR (100 MHz, Aseton-*d*$_6$)** δ 146.2, 144.9, 138.9, 136.8, 136.0, 132.2, 128.7, 128.4, 126.5, 126.3, 125.7, 124.2, 111.1, 102.7. **HRMS (ESI-TOF) m/z:** $[M + H]^+$ calcd for $C_{30}H_{23}N_2$, 411.1856; found 411.1882.

5-(4-(1,2,2-Triphenylvinyl)phenyl)-1*H*-indole (32). Compound **32** was obtained according to **GP1**. Column chromatography gave the product as a white-off solid (170 mg, 85% yield; mp 211.6–212.6 °C). **^{1}H NMR (400 MHz, CDCl$_3$).** δ 8.11 (bs, 1H), 7.87 (s, 1H), 7.53 – 7.37 (m, 4H), 7.37 – 6.95 (m, 18H), 6.61 (s, 1H). **^{13}C NMR (100 MHz, CDCl$_3$):** δ 144.0, 143.93, 143.91, 141.7, 140.8, 140.3, 135.3, 132.9 (2C), 131.7, 131.5, 131.4, 131.4, 128.4, 127.8, 127.68, 127.65, 126.4, 126.4 (2C), 124.8, 121.7, 119.0, 111.2, 103.0. **HRMS (ESI-TOF) m/z:** $[M + H]^+$ calcd for $C_{34}H_{26}N$, 448.2060; found 448.2065.

5,5'-((2,2-Diphenylethene-1,1-diyl)bis(4,1-phenylene))bis(1*H*-indole) (33). Compound **33** was obtained according to **GP1**. Column chromatography gave the product as a light brown solid (95 mg, 82% yield; mp >300 °C). **^{1}H NMR (400 MHz, DMSO-*d*$_6$):** δ 11.13 (s, 2H), 7.78 (s, 2H), 7.48 (d, *J* = 7.2 Hz, 4H), 7.43 (d, *J* = 8.5 Hz, 2H), 7.40 – 7.33 (m, 4H), 7.25 – 7.01 (m, 14H), 6.45 (s, 2H). **^{13}C NMR (100 MHz, DMSO-*d*$_6$):** δ 143.5, 141.1, 140.2, 140.1, 139.7, 135.5, 131.3, 130.7, 130.5, 128.2, 127.9, 126.5, 126.0, 125.8, 120.1, 117.9, 111.7, 101.5. **HRMS (ESI-TOF) m/z:** $[M + H]^+$ calcd for $C_{42}H_{31}N_2$, 563.2482; found 563.2476.

1,1,2,2-Tetrakis(4-(1*H*-indol-5-yl)phenyl)ethane (34). Compound **34** was obtained according to **GP1**. Column chromatography gave the product as yellow solid (100 mg, 81% yield; mp >300 °C). **^{1}H NMR (400 MHz, DMSO-*d*$_6$):** δ 11.10 (bs, 4H), 7.79 (s, 4H), 7.51 (d, *J* = 7.1 Hz, 8H), 7.46 – 7.30 (m, 12H), 7.15 (d, *J* = 7.2 Hz, 8H), 6.44 (s, 4H). **^{13}C NMR (100 MHz, DMSO-*d*$_6$):** δ 141.5, 139.7, 139.7, 135.5, 131.4, 130.6, 128.2, 125.9, 125.8, 120.1, 117.9, 111.7, 101.6. **HRMS (ESI-TOF) m/z:** $[M + H]^+$ calcd for $C_{58}H_{41}N_4$, 793.3326; found 793.3314.

General procedure for BIM-TPE = GP3

To a stirred solution of indole-modified (**9**) or indole-substituted (**31**) TPEs (2 equiv.) in CH_2Cl_2 (15 mL) aldehyde (1 equiv.) and a catalytic amount of $Zn(CF_3COO)_2{\cdot}xH_2O$ (0.01 equiv.) was added and the mixture was stirred at room temperature for 12 h. After completion of the reaction (monitored by TLC), the solvent was evaporated. The residue was purified by silica gel column chromatography (EtOAc/hexane) to give the desired compound.

3,3'-(Phenylmethylene)bis(5-(1,2,2-triphenylvinyl)-1*H*-indole) (36a). Compound **36a** was obtained according to **GP3**. Column chromatography gave the product as yellow solid (52 mg, 93% yield; mp 162.4–163.4 °C). **^{1}H NMR (400 MHz, CDCl$_3$):** δ 7.50 (bs, 2H), 7.11 – 7.02 (m, 5H), 7.02 – 6.84 (m, 34H), 6.76 – 6.70 (m, 2H), 6.27 (d, *J* = 1.8 Hz, 2H), 5.20 (s, 1H). **^{13}C NMR (100 MHz, CDCl$_3$):** δ 144.5, 144.4, 144.3, 144.0, 142.2, 139.5, 135.3, 134.7, 131.44, 131.40, 131.36, 128.4, 128.0, 127.6, 127.5, 127.4, 126.6, 126.1, 125.9, 125.7, 125.6, 123.4, 123.2, 120.0, 110.1, 39.7. **HRMS (ESI-TOF) m/z:** $[M + H]^+$ calcd for $C_{63}H_{47}N_2$, 831.3734; found 831.3720.

3,3'-((4-Fluorophenyl)methylene)bis(5-(1,2,2-triphenylvinyl)-1*H*-indole) (36b). Compound **36b** was obtained according to **GP3**. Column chromatography gave the product as yellow solid (51 mg, 89% yield; mp 148.9–149.9 °C). **^{1}H NMR (400 MHz, Acetone-*d*$_6$):** δ 9.86 (bs, NH, 2H), 7.15 (d, *J* = 8.4 Hz, 2H), 7.12 – 6.84 (m, 36H), 6.78 (dd, *J* = 8.4, 1.5 Hz, 2H), 6.65 (d, *J* = 2.0 Hz, 2H), 5.35 (s, 1H). **^{13}C NMR (100 MHz, Acetone-*d*$_6$):** δ 161.9 (d, *J* = 241.8 Hz), 145.5, 145.4, 145.3, 143.5, 141.8 (d, *J* = 2.4 Hz), 140.3, 136.8, 134.9, 132.1, 132.0, 131.9, 130.7 (d, *J* = 7.7 Hz), 128.4, 128.4, 128.3, 127.5, 127.0, 126.9, 126.8, 125.8, 124.7, 123.5, 119.9, 115.3 (d, *J* = 21.2 Hz), 111.4, 39.9. **HRMS (ESI-TOF) m/z:** [M + H]$^+$ calcd for $C_{63}H_{46}FN_2$, 849.3640; found 849.3649.

3,3'-((4-Bromophenyl)methylene)bis(5-(1,2,2-triphenylvinyl)-1*H*-indole) (36c). Compound **36c** was obtained according to **GP3**. Column chromatography gave the product as yellow solid (57 mg, 93% yield; mp 213.8–214.8 °C). **^{1}H NMR (400 MHz, CDCl$_3$):** δ 7.60 (bs, 2H), 7.18 (s, 2H), 7.14 (d, *J* = 8.4 Hz, 2H), 7.09 – 6.71 (m, 36H), 6.28 (s, 2H), 5.21 (s, 1H). **^{13}C NMR (100 MHz, CDCl$_3$):** δ 144.4, 144.2, 143.1, 142.1, 139.6, 135.3, 134.9, 131.4, 131.37, 131.35, 131.1, 130.1, 127.6, 127.5, 127.4, 126.4 126.13, 126.1, 125.84, 125.77, 123.4, 123.0, 119.6, 119.3, 110.2, 104.3, 39.2. **HRMS (ESI-TOF) m/z:** [M + H]$^+$ calcd for $C_{63}H_{46}BrN_2$, 909.2839; found 909.2835.

3,3'-((4-Methoxyphenyl)methylene)bis(5-(1,2,2-triphenylvinyl)-1*H*-indole) (36d). Compound **36d** was obtained according to **GP3**. Column chromatography gave the product as yellow solid (55 mg, 89% yield; mp 158.0–159.0 °C). **^{1}H NMR (400 MHz, CDCl$_3$):** δ 7.51 (s, 2H), 7.04 – 6.83 (m, 38H), 6.73 (d, *J* = 8.5 Hz, 2H), 6.60 (d, *J* = 8.5 Hz, 2H), 6.25 (d, *J* = 1.5 Hz, 2H), 5.16 (s, 1H), 3.71 (s, 3H). **^{13}C NMR (100 MHz, CDCl$_3$):** δ 157.6, 144.5, 144.5, 144.3, 142.2, 139.5, 136.3, 135.3, 134.7, 131.5, 131.4, 131.4, 129.3, 127.6, 127.5, 127.4, 126.6, 126.1, 125.7, 125.6, 123.3, 123.2, 120.3, 113.4, 110.1, 55.2, 38.9 (1C signal overlaps). **HRMS (ESI-TOF) m/z:** [M + H]$^+$ calcd for $C_{64}H_{49}N_2O$, 861.3839; found 861.3824.

4-(Bis(5-(1,2,2-triphenylvinyl)-1*H*-indol-3-yl)methyl)phenyl acetate (36e). Compound **36e** was obtained according to **GP3**. Column chromatography gave the product as yellow solid (55 mg, 95% yield; mp 189.1–190.1 °C). **^{1}H NMR (400 MHz, DMSO-*d*$_6$):** δ 10.71 (d, *J* = 1.7 Hz, 2H), 7.16 – 6.82 (m, 38H), 6.77 – 6.54 (m, 4H), 5.14 (s, 1H), 2.26 (s, 3H). **^{13}C NMR (100 MHz, DMSO-*d*$_6$):** δ 169.1, 148.2, 144.0, 143.9, 143.7, 142.2, 142.0, 138.8, 135.0, 133.0, 130.7, 130.5, 128.5, 127.7, 127.6, 127.5, 126.2, 126.1, 126.0, 125.9, 124.2, 123.6, 121.68, 121.65, 121.0, 118.1, 110.6, 38.2, 20.9. **HRMS (ESI-TOF) m/z:** [M + H]$^+$ calcd for $C_{65}H_{49}N_2O_2$, 889.3789; found 889.3780.

3,3'-((4-(Trifluoromethyl)phenyl)methylene)bis(5-(1,2,2-triphenylvinyl)-1*H*-indole) (36f). Compound **36f** was obtained according to **GP3**. Column chromatography gave the product as yellow solid (51 mg, 92% yield; mp 207.2–208.2 °C). **^{1}H NMR (400 MHz, DMSO-*d*$_6$):** δ 10.76 (bs, 2H), 7.43 (d, *J* = 7.6 Hz, 2H), 7.16 – 6.82 (m, 36H), 6.71 (s, 2H), 6.67 (d, *J* = 8.4 Hz, 2H), 5.31 (s, 1H). **^{13}C NMR (100 MHz, DMSO-*d*$_6$):** δ 149.5, 144.0, 143.9, 143.7, 142.0, 138.9, 135.1, 133.2, 130.7, 130.6, 130.5, 128.3, 127.7, 127.62, 127.56, 127.4, 126.1 (q, *J* = 31.9 Hz), 126.1, 126.1, 125.9, 124.8 – 124.6 (m), 124.5 (q, *J* = 273.3 Hz), 124.3, 123.8, 121.6, 119.3, 117.2, 110.7, 38.9 (3C signal overlaps). **HRMS (ESI-TOF) m/z:** [M + H]$^+$ calcd for $C_{64}H_{46}F_3N_2$, 899.3608; found 899.3617.

3,3'-(Phenylmethylene)bis(5-(4-(1,2,2-triphenylvinyl)phenyl)-1*H*-indole) (37a). Compound **37a** was obtained according to **GP3**. Column chromatography gave the product as yellow solid (54 mg, 96% yield; mp 198.6–199.6 °C). **^{1}H NMR (400 MHz, CDCl$_3$):** δ 7.79 (d, *J* = 1.8 Hz, 2H), 7.53 (s, 2H), 7.40 – 7.17 (m, 14H), 7.15 – 6.96 (m, 34H), 5.89 (s, 1H). **^{13}C NMR (100 MHz, CDCl$_3$)**: δ 144.0, 143.9, 143.9, 141.6, 140.8, 140.7, 140.2, 136.2, 132.3, 131.6, 131.5, 131.40, 131.36, 128.7, 128.3, 127.7, 127.63, 127.61, 127.60, 126.4, 126.3 (2C), 126.2, 124.3, 121.6, 120.1, 118.0, 111.2, 40.1 (2C signal overlaps). **HRMS (ESI-TOF) m/z:** [M + H]$^+$ calcd for $C_{75}H_{55}N_2$, 983.4360; found 983.4352.

3,3'-((4-Fluorophenyl)methylene)bis(5-(4-(1,2,2-triphenylvinyl)phenyl)-1*H*-indole) (37b). Compound **37b** was obtained according to **GP3**. Column chromatography gave the product as yellow solid (49 mg, 93% yield; mp 175.9–176.9 °C). ^{1}**H NMR (400 MHz, CDCl$_3$):** δ 7.69 (bs, 2H), 7.42 (s, 2H), 7.28 (dd, *J* = 8.5, 1.6 Hz, 2H), 7.22 (d, *J* = 8.5 Hz, 2H), 7.21 – 7.12 (m, 8H), 7.07 – 6.90 (m, 32H), 6.85 (t, *J* = 8.7 Hz, 2H), 6.49 (d, *J* = 1.6 Hz, 2H), 5.78 (s, 1H). ^{13}**C NMR (100 MHz, CDCl$_3$):** δ 161.5 (d, *J* = 243.9 Hz), 144.0, 143.90, 143.87, 141.7, 140.8, 140.1, 139.6, 139.6, 136.2, 132.4, 131.7, 131.5, 131.4, 131.4, 130.1 (d, *J* = 7.7 Hz), 127.8, 127.7, 127.6, 127.4, 126.4, 126.3, 124.3, 121.8, 119.9, 117.9, 115.0 (d, *J* = 21.2 Hz), 111.3, 39.4. **HRMS (ESI-TOF) m/z:** [M + H]$^+$ calcd for $C_{75}H_{54}FN_2$, 1001.4266; found 1001.4261

3,3'-((4-Bromophenyl)methylene)bis(5-(4-(1,2,2-triphenylvinyl)phenyl)-1*H*-indole) (37c). Compound **37c** was obtained according to **GP3**. Column chromatography gave the product as white-off solid (52 mg, 89% yield; mp 194.1–195.1 °C). ^{1}**H NMR (400 MHz, CDCl$_3$):** δ 7.75 (bs, 2H), 7.49 (s, 2H), 7.38 – 7.32 (m, 4H), 7.28 (d, *J* = 8.4 Hz, 4H), 7.24 (d, *J* = 8.4 Hz, 4H), 7.16 (d, *J* = 8.4 Hz, 2H), 7.12 – 6.97 (m, 32H), 6.54 (d, *J* = 1.3 Hz, 2H), 5.82 (s, 1H). ^{13}**C NMR (100 MHz, CDCl$_3$):** δ 144.0, 143.91, 143.90, 143.1, 141.7, 140.8, 140.8, 140.1, 136.2, 132.5, 131.7, 131.5, 131.4, 131.4, 130.5, 127.8, 127.7, 127.7, 127.3, 126.4, 126.4, 124.3, 121.8, 120.0, 119.4, 117.8, 111.4, 39.6 (3C signal overlaps). **HRMS (ESI-TOF) m/z:** [M + H]$^+$ calcd for $C_{75}H_{54}BrN_2$, 1061.3465; found 1061.3466

3,3'-((4-Methoxyphenyl)methylene)bis(5-(4-(1,2,2-triphenylvinyl)phenyl)-1*H*-indole) (37d). Compound **37d** was obtained according to **GP3**. Column chromatography gave the product as yellow solid (52 mg, 92% yield; mp 180.0–181.0 °C). ^{1}**H NMR (400 MHz, CDCl$_3$):** δ 7.74 (bs, 2H), 7.46 (s, 2H), 7.31 – 7.22 (m, 4H), 7.21 – 7.13 (m, 6H), 7.06 – 6.88 (m, 34H), 6.72 (d, *J* = 8.6 Hz, 2H), 6.54 (s, 2H), 5.78 (s, 1H), 3.68 (s, 3H). ^{13}**C NMR (100 MHz, CDCl$_3$)**: δ 158.0, 144.0, 143.91, 143.90, 141.6, 140.8, 140.7, 140.2, 136.3, 136.2, 132.2, 131.6, 131.5, 131.39, 131.36, 129.6, 127.7, 127.6, 127.6, 127.5, 126.4, 126.3 (2C), 124.2, 121.6, 120.4, 118.1, 113.6, 111.2, 55.3, 39.2 (1C signal overlaps). **HRMS (ESI-TOF) m/z:** [M + H]$^+$ calcd for $C_{76}H_{57}N_2O$, 1013.4465; found 1013.4474.

4-(Bis(5-(4-(1,2,2-triphenylvinyl)phenyl)-1*H*-indol-3-yl)methyl)phenyl acetate (37e). Compound **37e** was obtained according to **GP3**. Column chromatography gave the product as yellow solid (52 mg, 95% yield; mp 201.3–202.3 °C). ^{1}**H NMR (400 MHz, CDCl$_3$):** δ 7.68 (bs, 2H), 7.43 (s, 2H), 7.27 (dd, *J* = 8.5, 1.4 Hz, 2H), 7.21 – 7.14 (m, 8H), 7.07 – 6.90 (m, 36H), 6.87 (d, *J* = 8.5 Hz, 2H), 5.78 (s, 1H), 2.18 (s, 3H). ^{13}**C NMR (100 MHz, CDCl$_3$):** δ 169.7, 149.0, 144.0, 143.91, 143.90, 141.6, 141.5, 140.8, 140.7, 140.1, 136.2, 132.3, 131.7, 131.5, 131.41, 131.40, 129.6, 127.8, 127.7, 127.6, 127.4, 126.39, 126.37, 126.3, 124.5, 121.7, 121.2, 119.7, 117.9, 111.3, 39.4, 21.2 (1C signal overlaps). **HRMS (ESI-TOF) m/z:** [M + H]$^+$ calcd for $C_{77}H_{57}N_2O_2$, 1041.4415; found 1041.4403.

3,3'-((4-(Trifluoromethyl)phenyl)methylene)bis(5-(4-(1,2,2-triphenylvinyl)phenyl)-1*H*-indole) (37f). Compound **37f** was obtained according to **GP3**. Column chromatography gave the product as yellow solid (50 mg, 89% yield; mp 185.3–186.3 °C). ^{1}**H NMR (400 MHz, CDCl$_3$):** δ 7.85 (d, *J* = 1.8 Hz, 2H), 7.51 (d, *J* = 8.2 Hz, 2H), 7.49 (s, 2H), 7.43 (d, *J* = 8.2 Hz, 2H), 7.38 (dd, *J* = 8.5, 1.4 Hz, 2H), 7.33 (d, *J* = 8.5 Hz, 2H), 7.27 – 7.21 (m, 4H), 7.14 – 6.98 (m, 34H), 6.61 (d, *J* = 1.7 Hz, 2H), 5.95 (s, 1H). ^{13}**C NMR (100 MHz, CDCl$_3$):** δ 148.2, 148.0, 144.0, 143.9, 141.8, 140.9, 140.8, 140.0, 136.2, 132.6, 131.7, 131.5, 131.43, 131.41, 129.1, 128.6 (q, *J* = 32.3 Hz), 127.8, 127.7, 127.69, 127.3, 126.5, 126.4, 126.38, 125.3 (q, *J* = 4.1 Hz), 124.5 (q, *J* = 272.2 Hz), 124.4, 121.9, 119.1, 117.8, 111.5, 40.0 (1C signal overlaps). **HRMS (ESI-TOF) m/z:** [M + H]$^+$ calcd for $C_{76}H_{54}F_3N_2$, 1051.4234; found 1051.4212.

2. NMR Spectra of Compounds

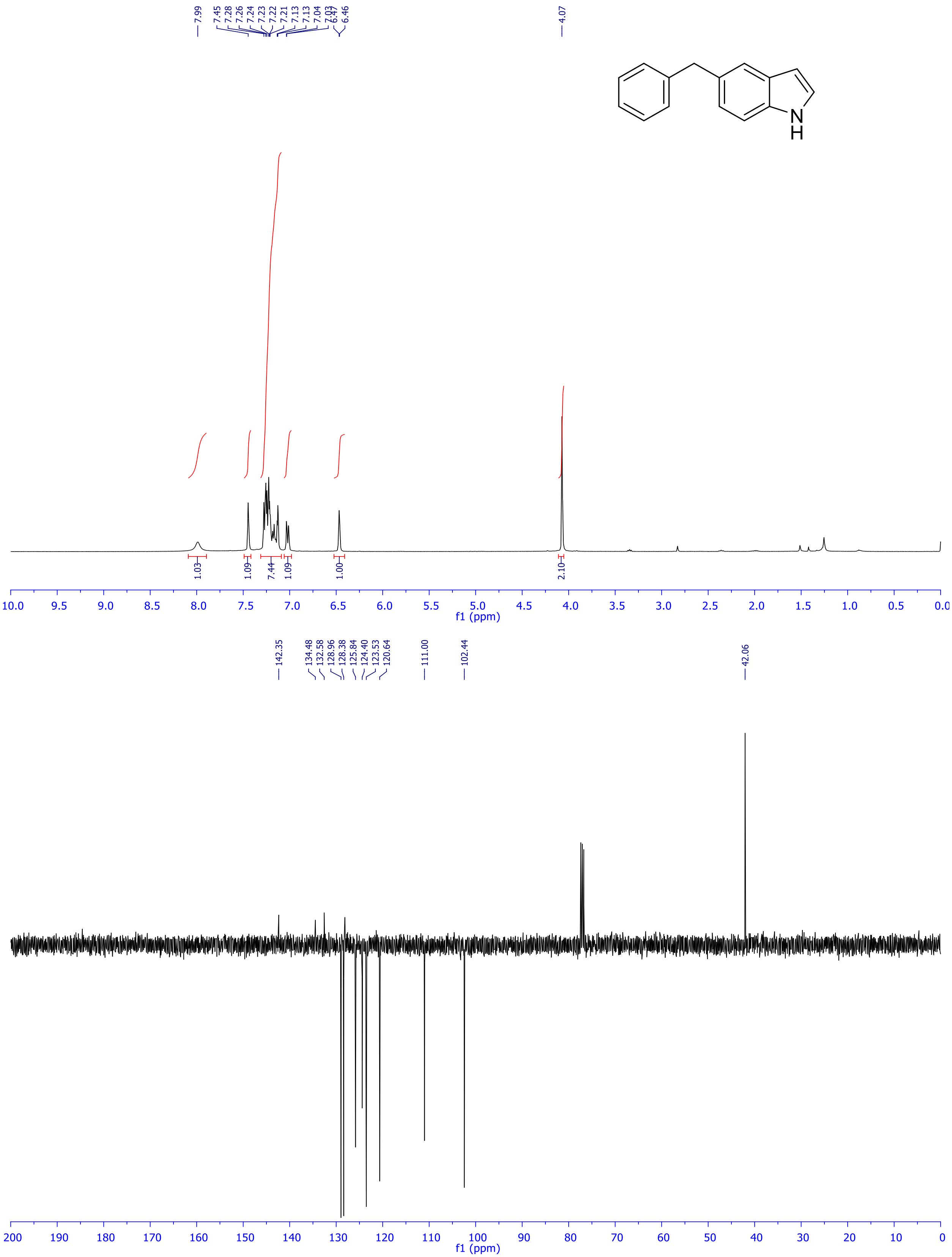

Figure S1. ^{1}H and ^{13}C (APT) NMR of **2**

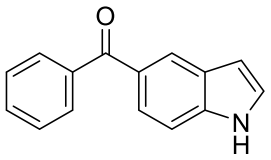

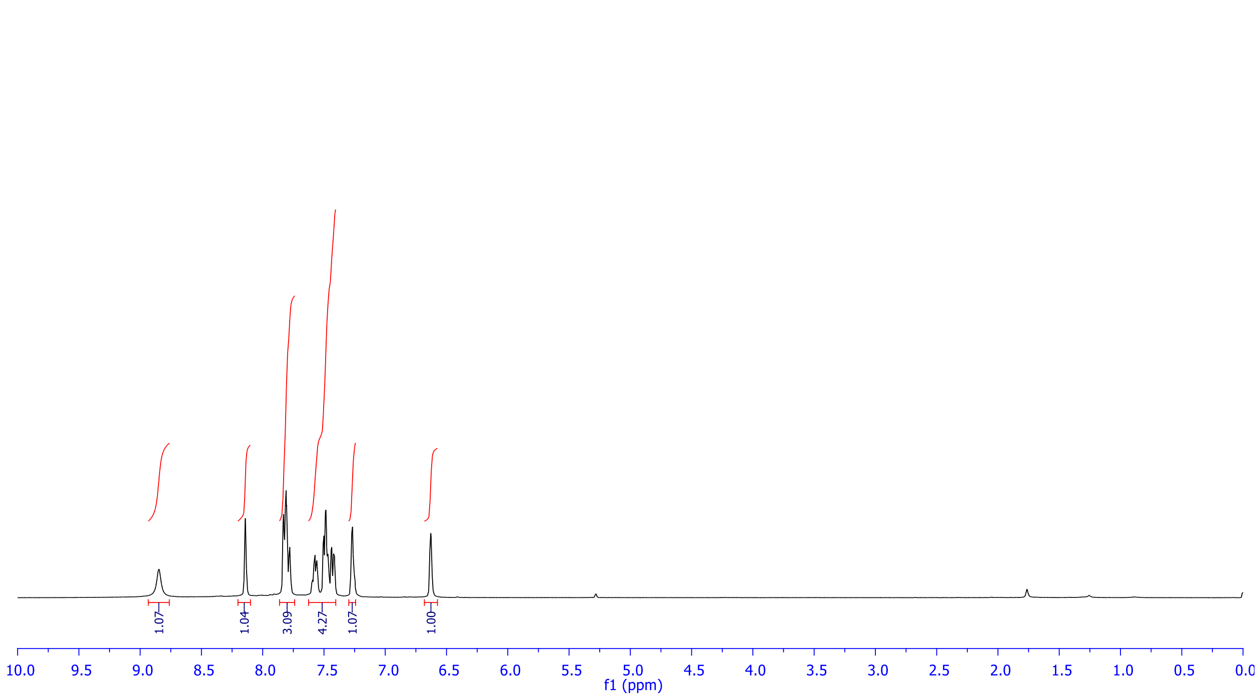

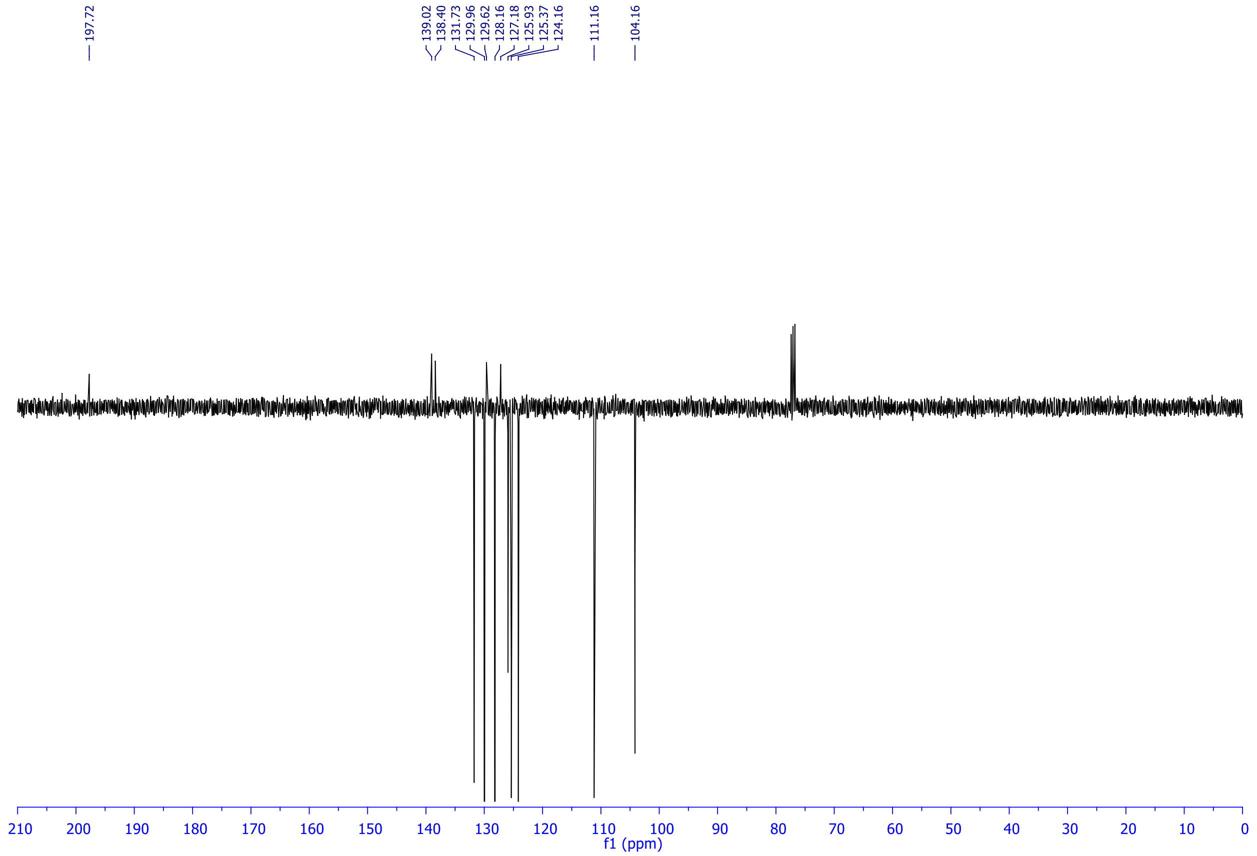

Figure S2. ^{1}H and ^{13}C (APT) NMR of **4**

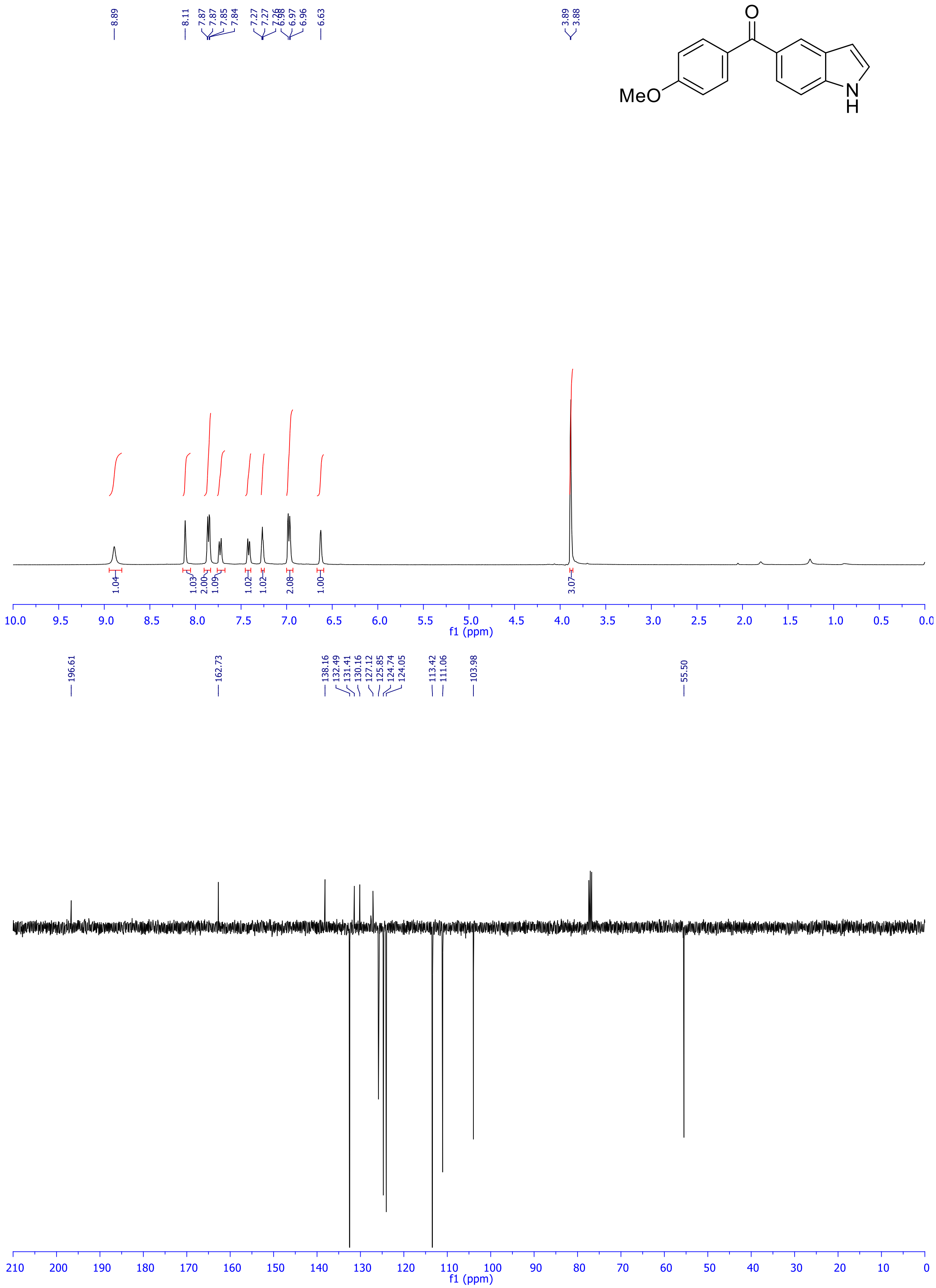

Figure S3. ^{1}H and ^{13}C (APT) NMR of **5**

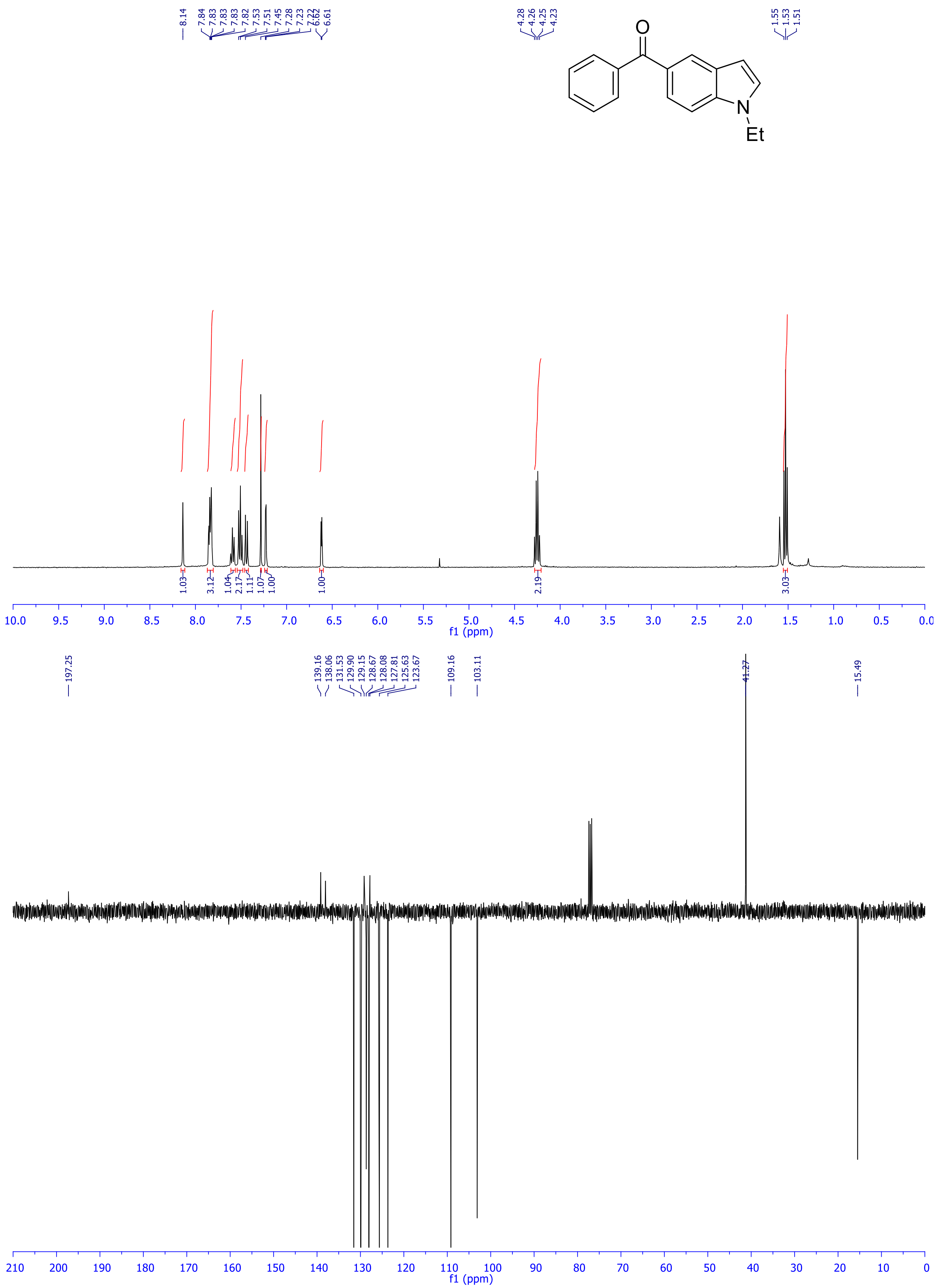

Figure S4. ^{1}H and ^{13}C (APT) NMR of **6**

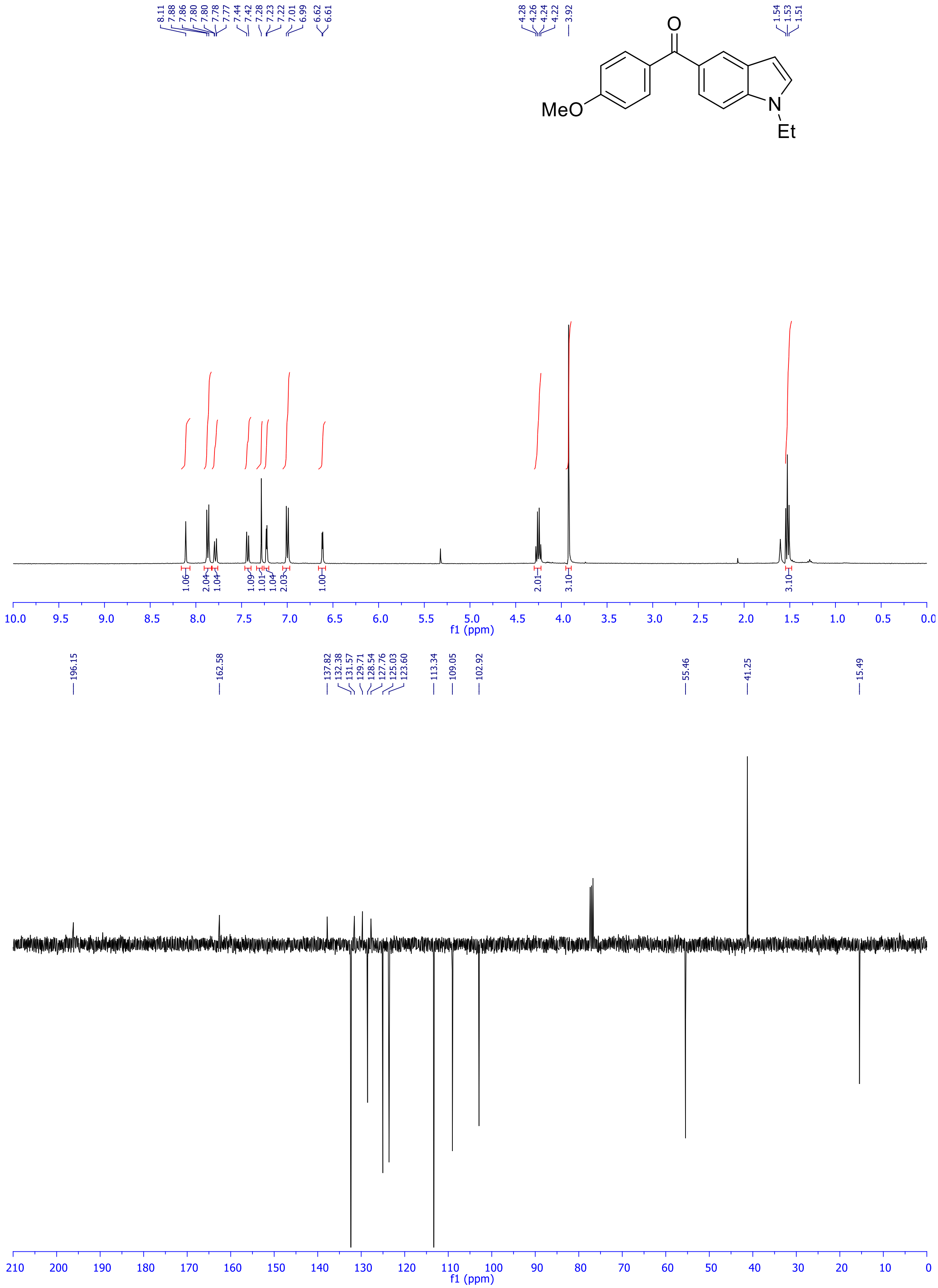

Figure S5. ^{1}H and ^{13}C (APT) NMR of **7**

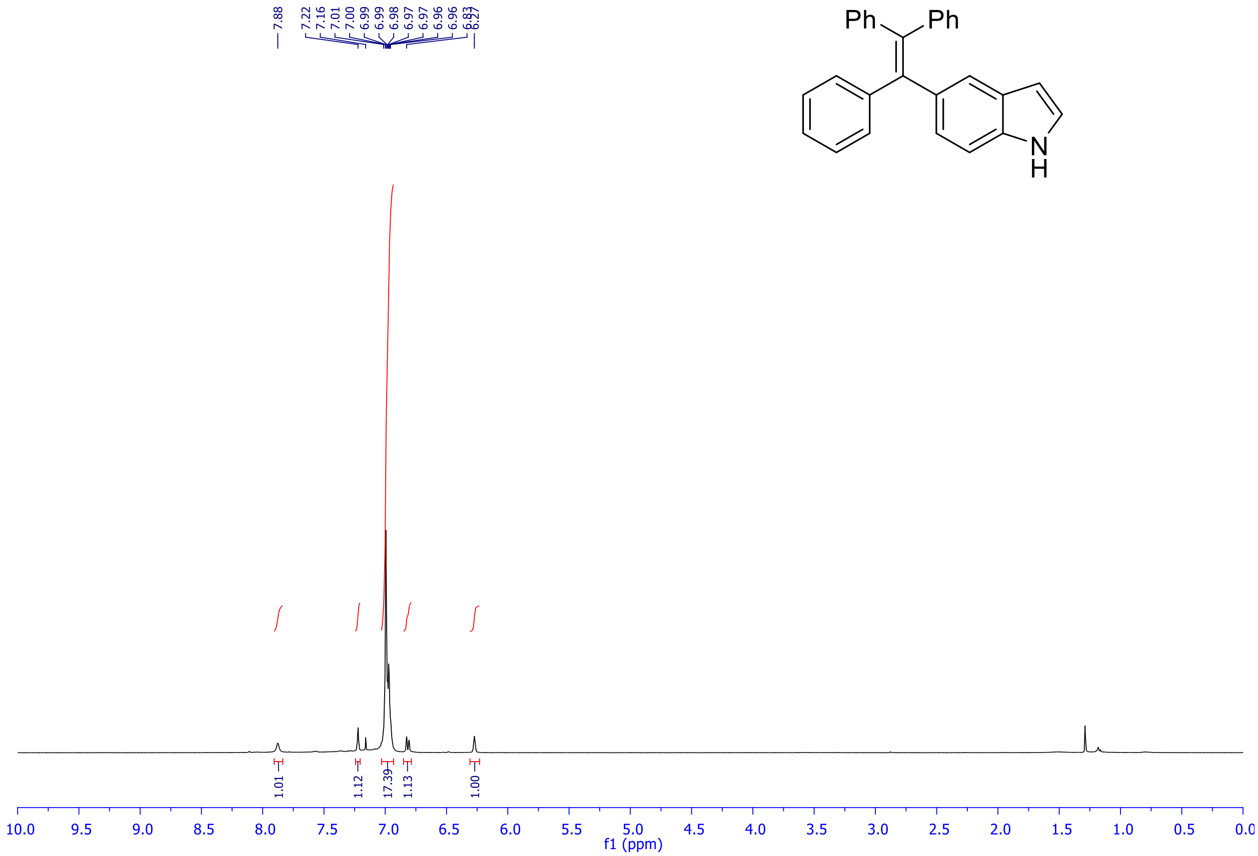

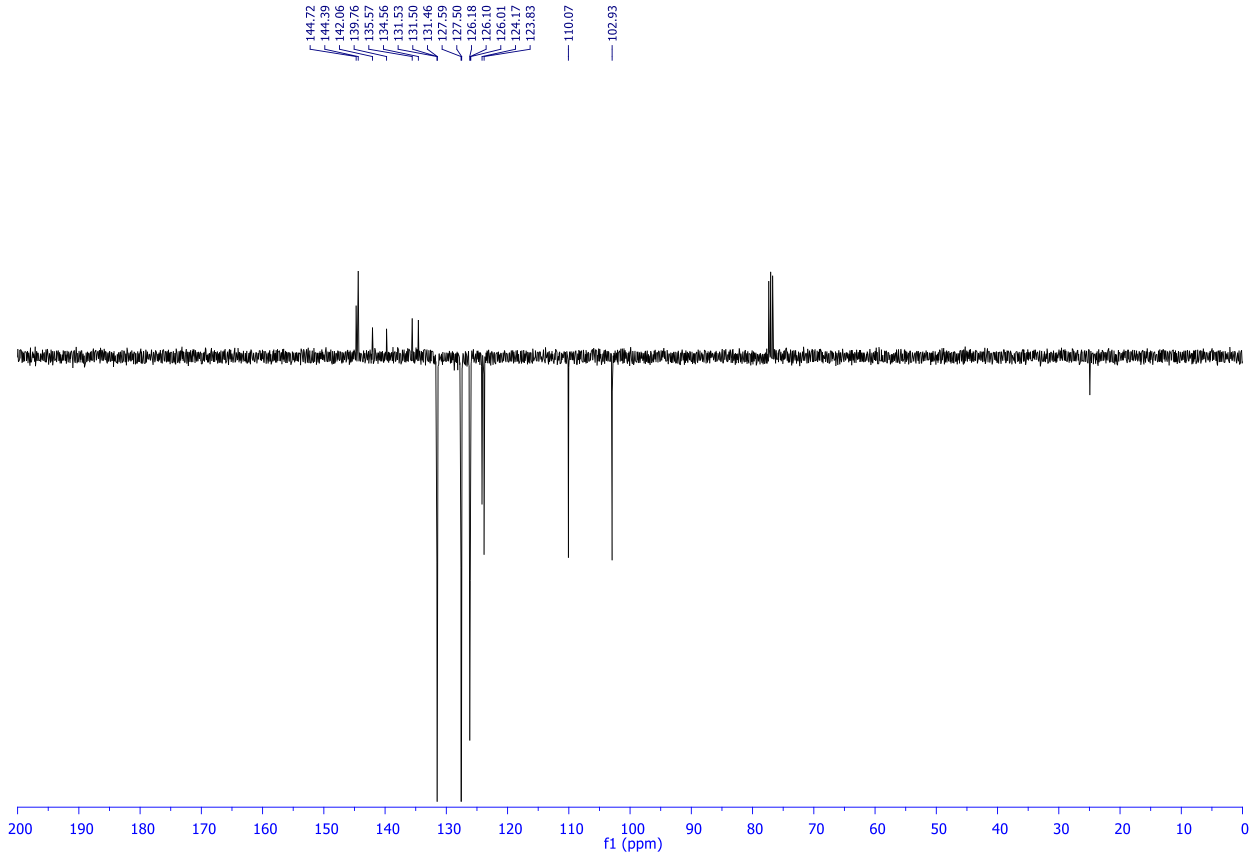

Figure S6. ^{1}H and ^{13}C (APT) NMR of **9**

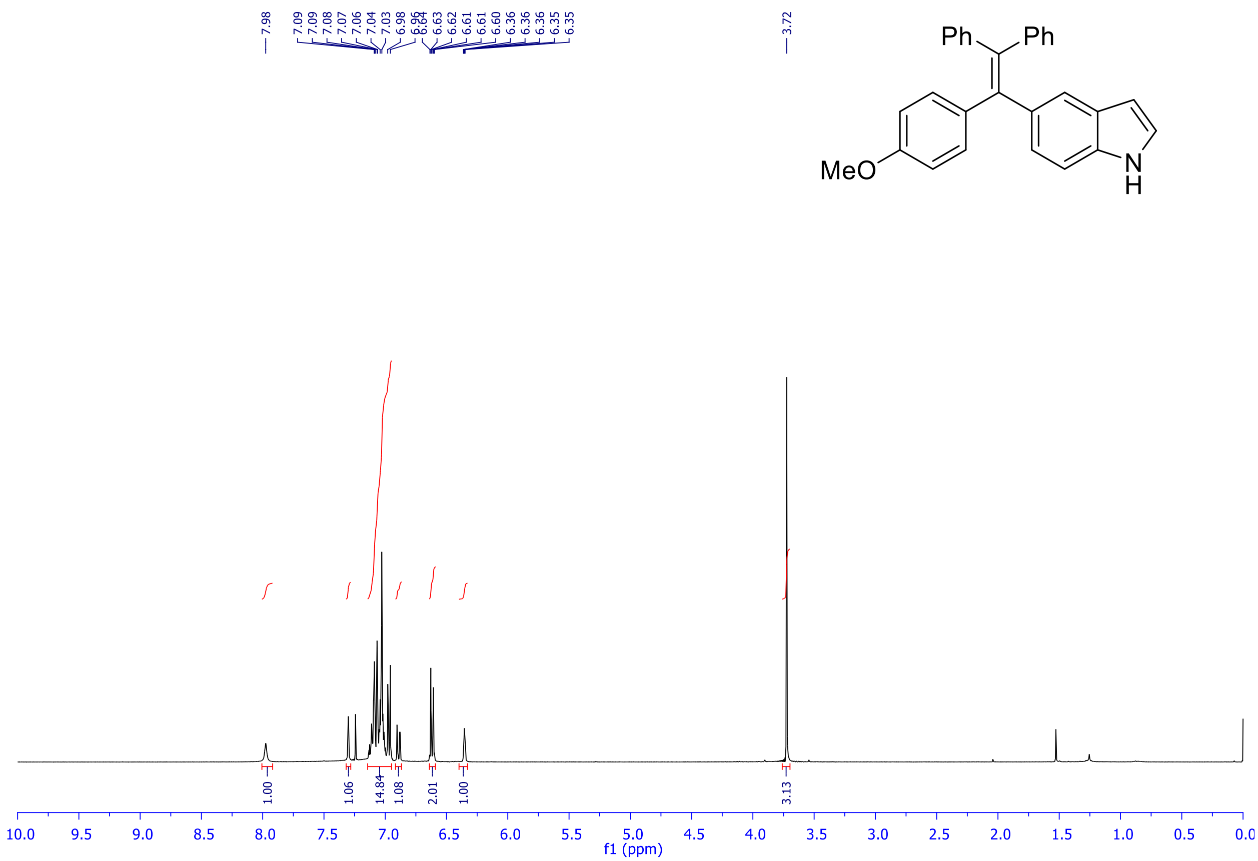

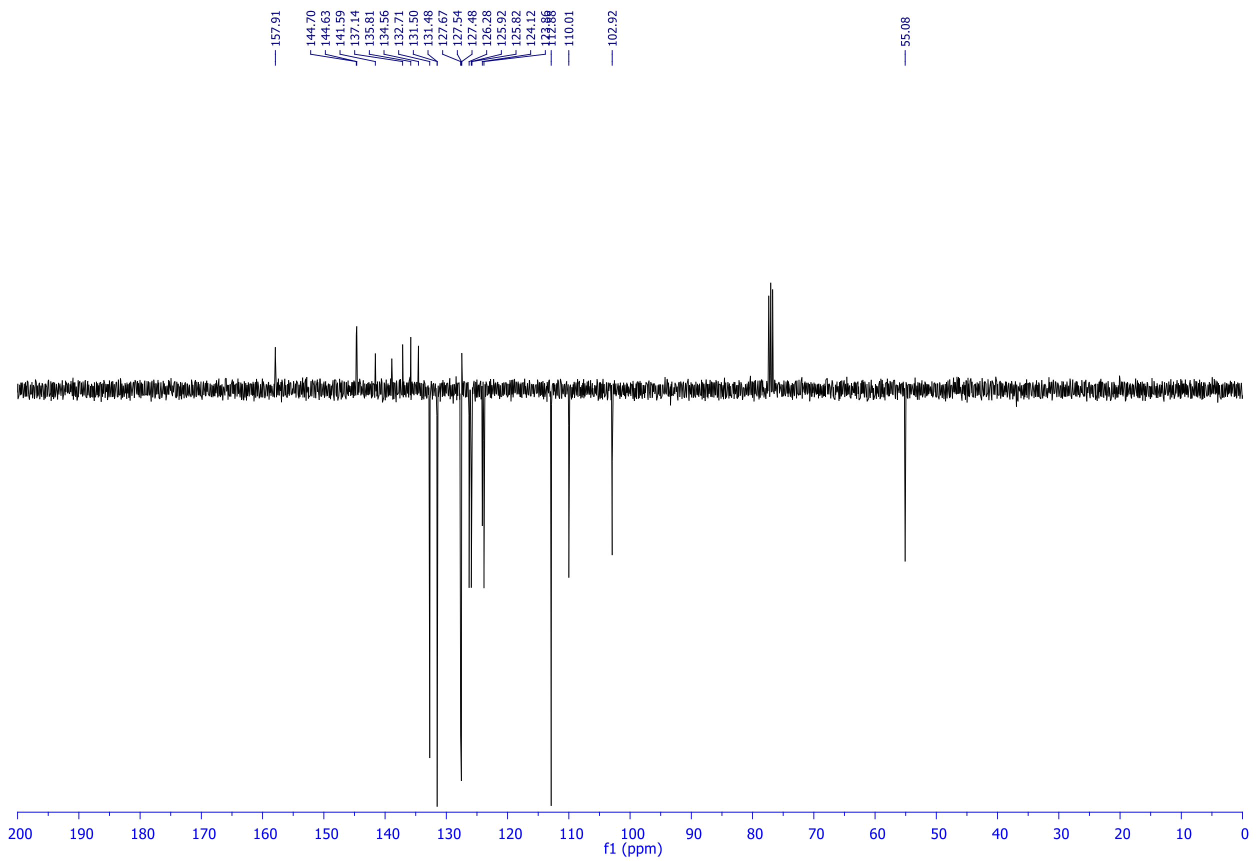

Figure S7. ^{1}H and ^{13}C (APT) NMR of **10**

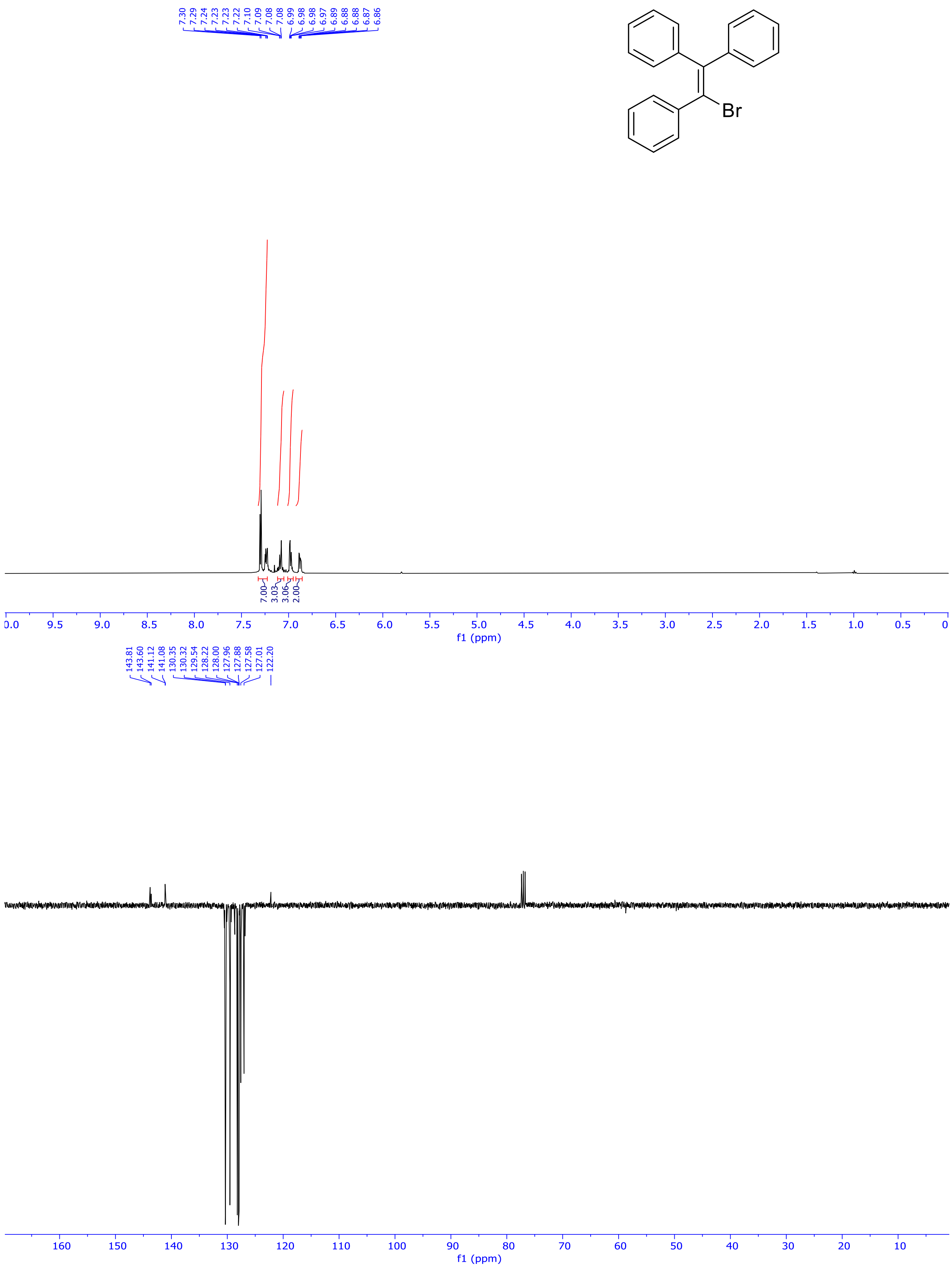

Figure S8. ^{1}H and ^{13}C (APT) NMR of **11**

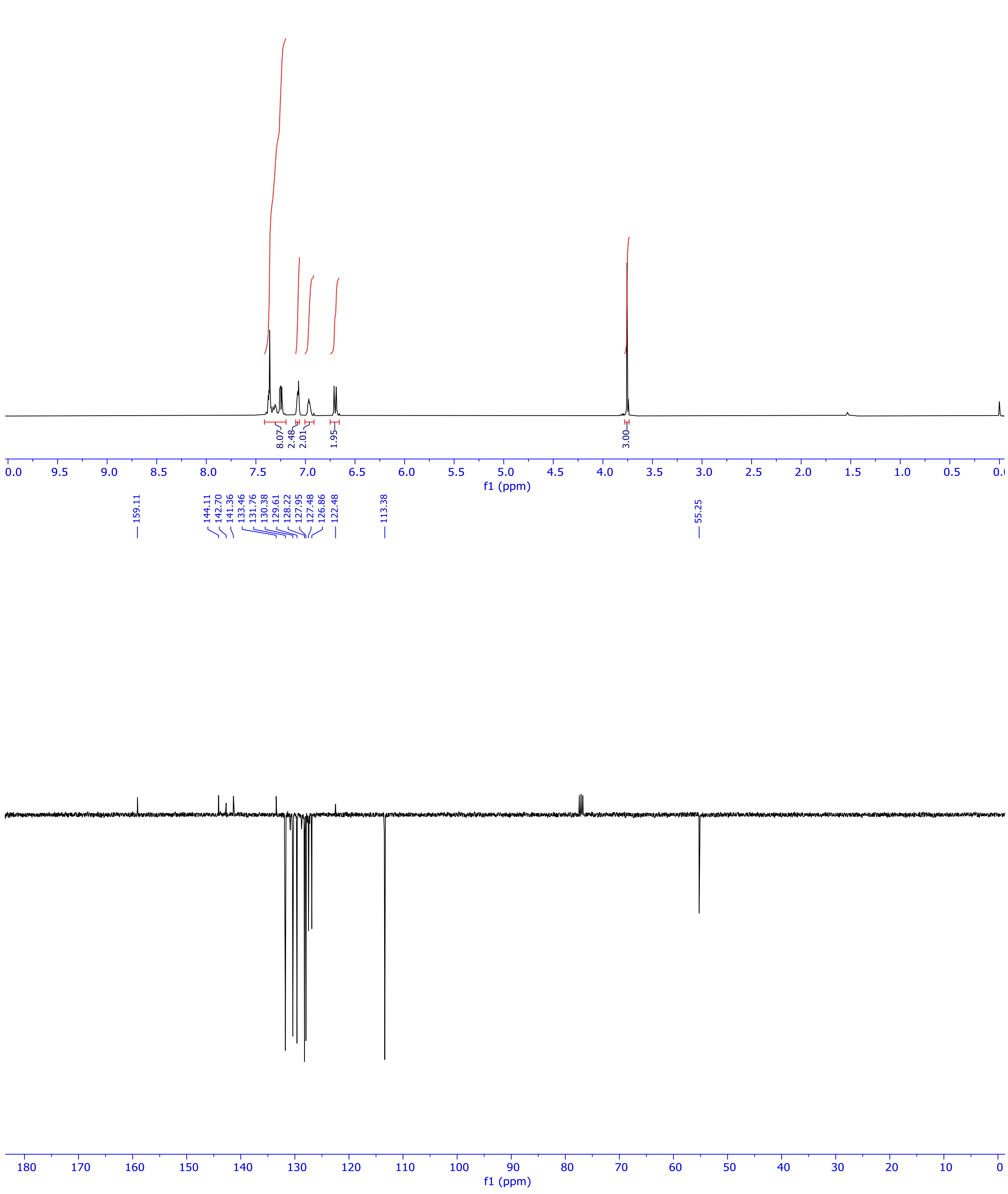

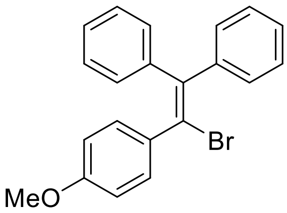

Figure S9. ^{1}H and ^{13}C (APT) NMR of **12**

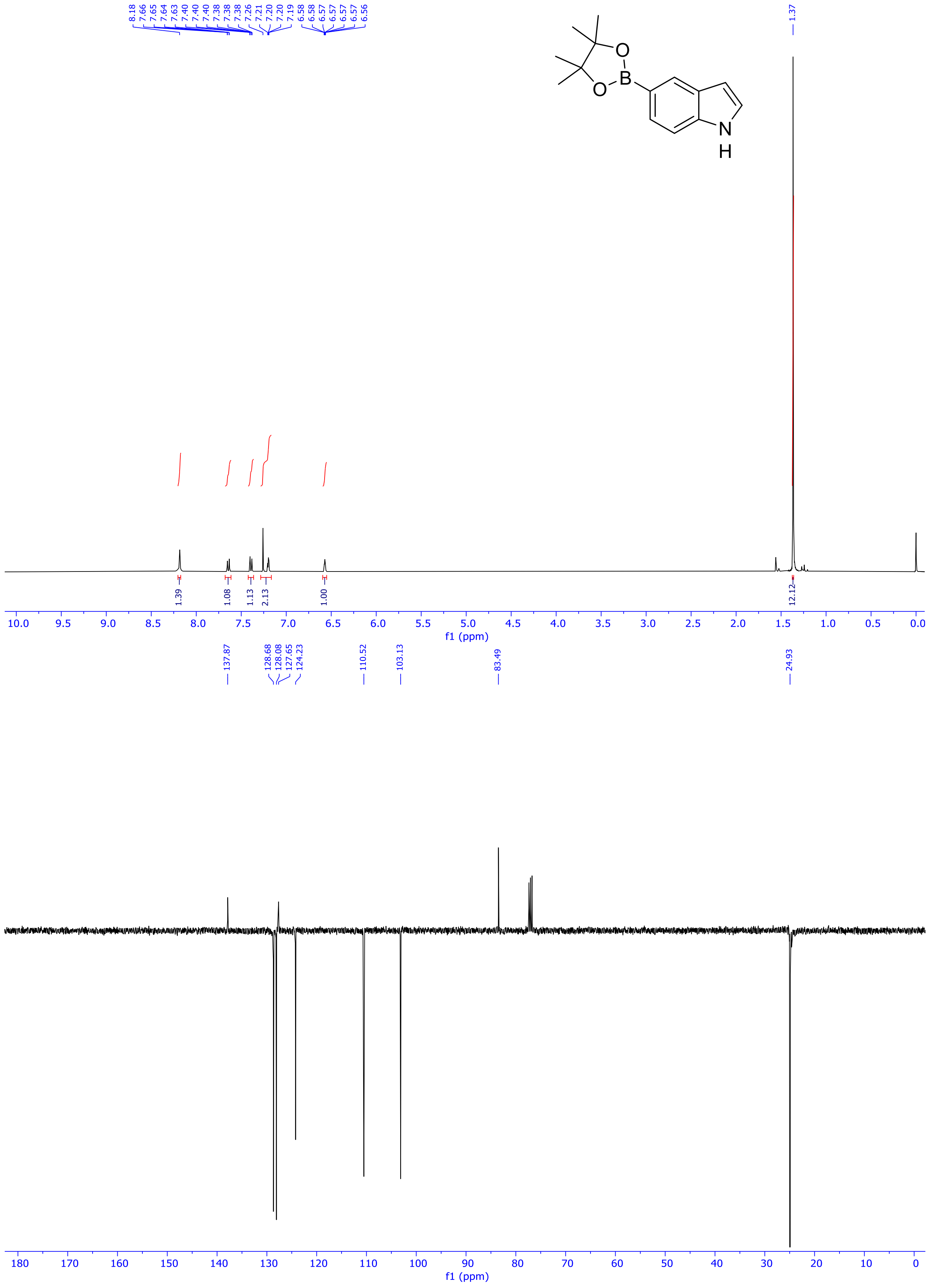

Figure S10. ^{1}H and ^{13}C (APT) NMR of **13**

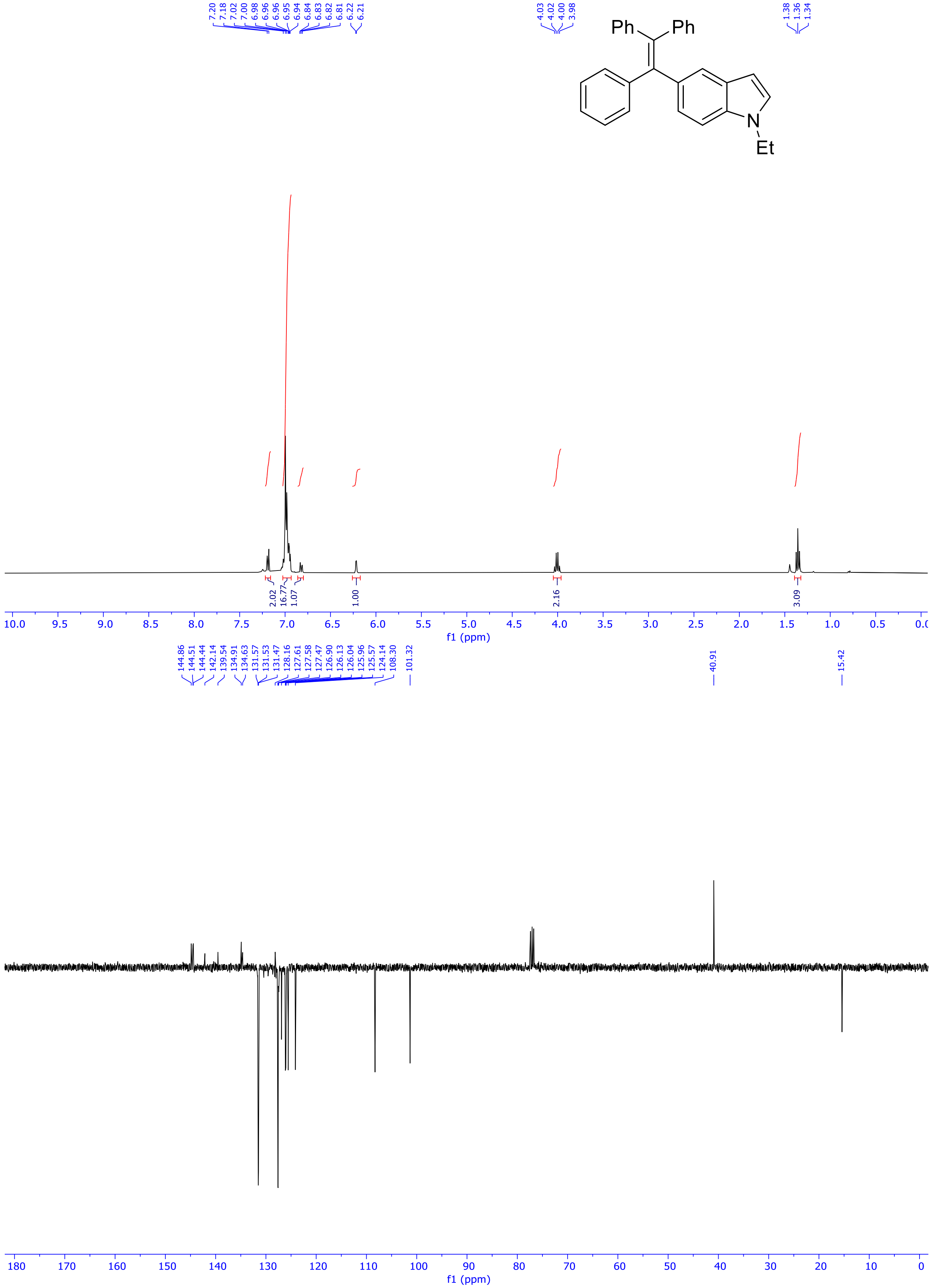

Figure S11. ^{1}H and ^{13}C (APT) NMR of **14**

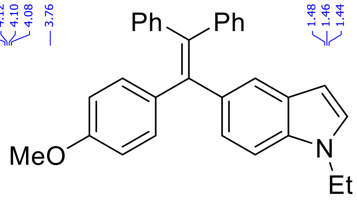

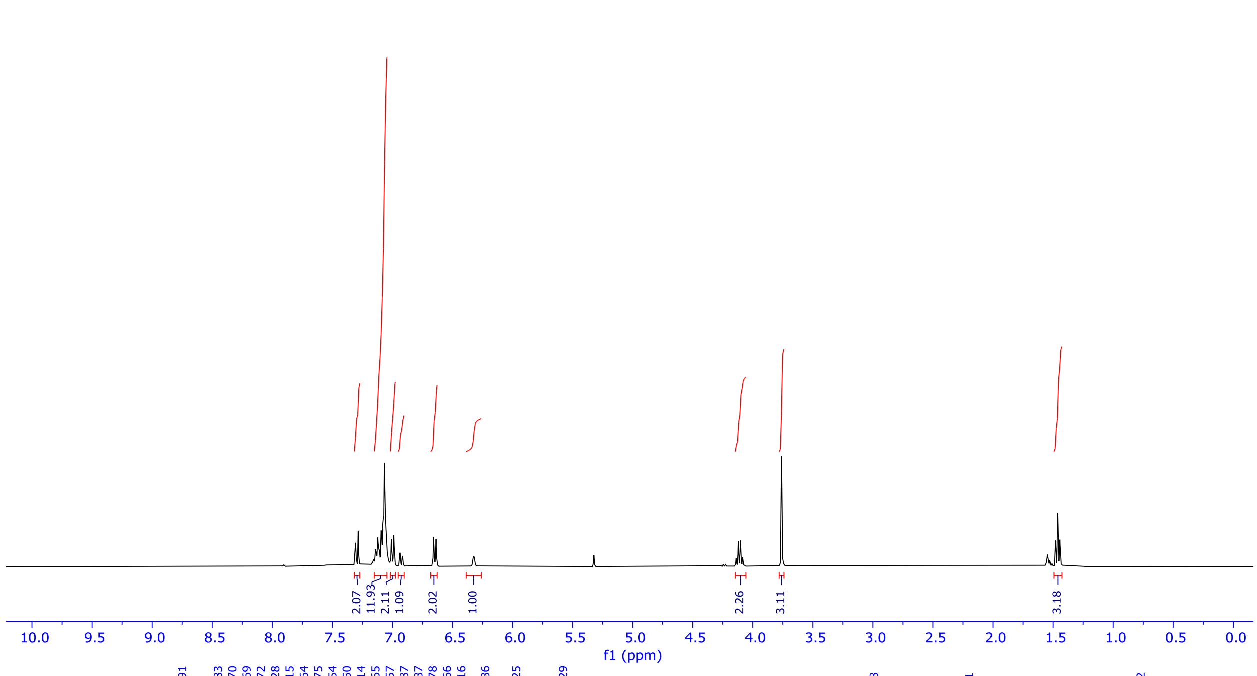

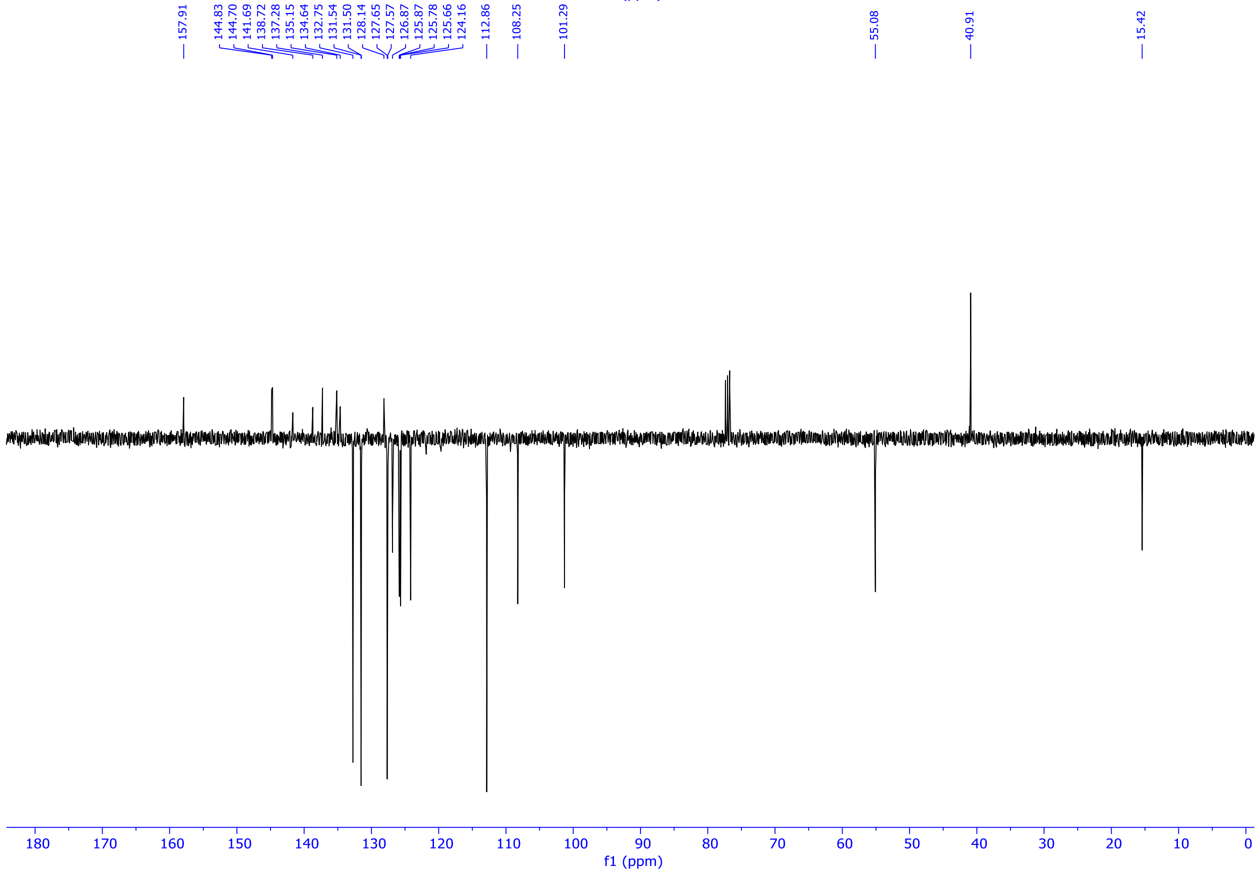

Figure S12. ^{1}H and ^{13}C (APT) NMR of **15**

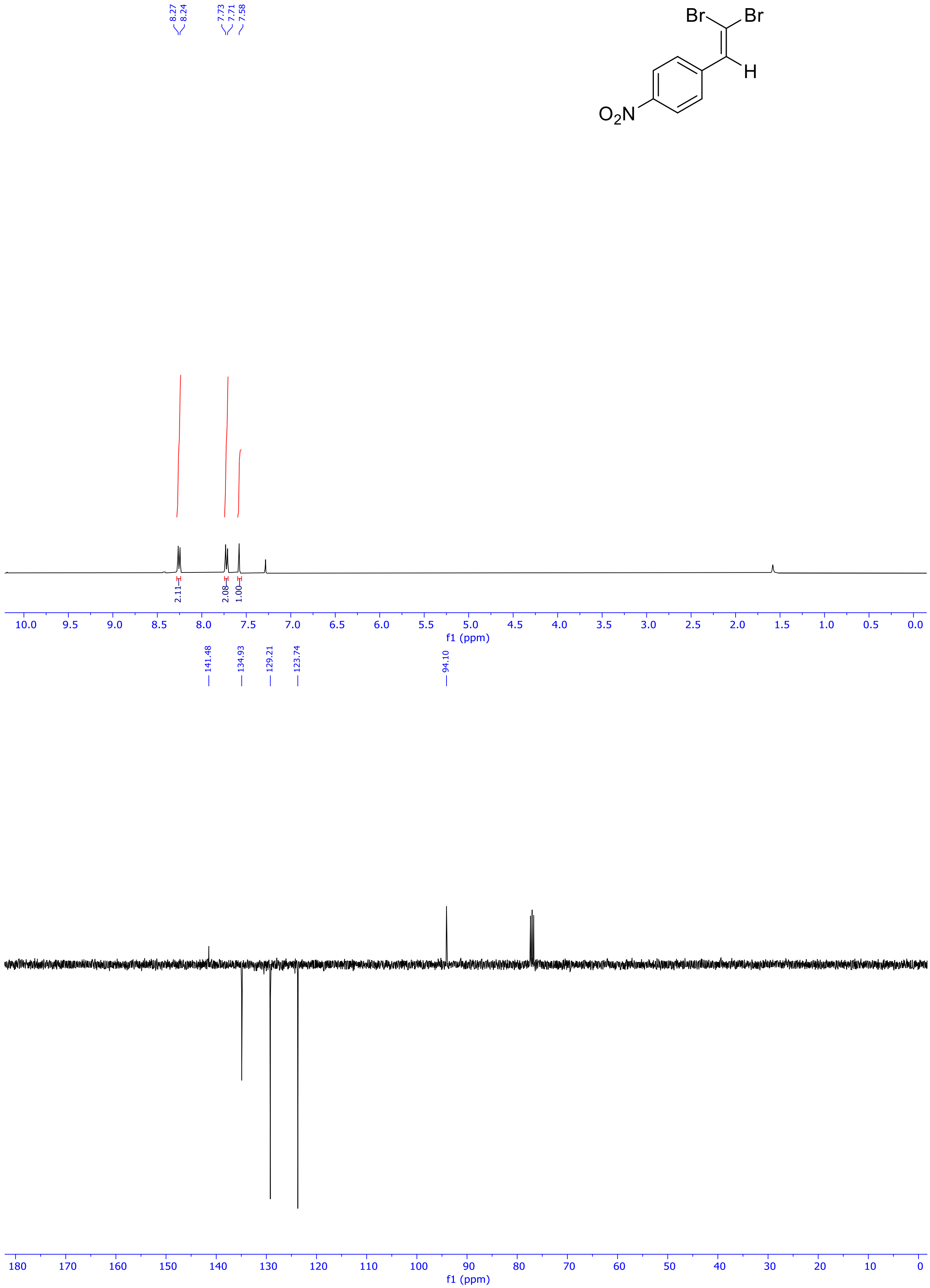

Figure S13. ^{1}H and ^{13}C (APT) NMR of **17**

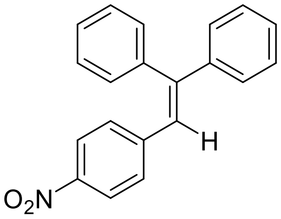

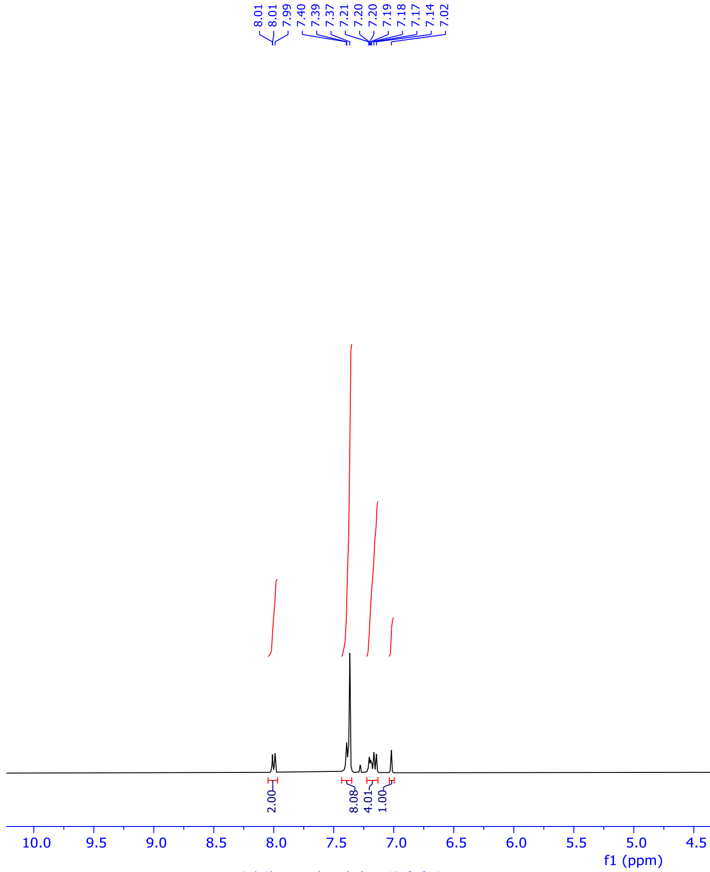

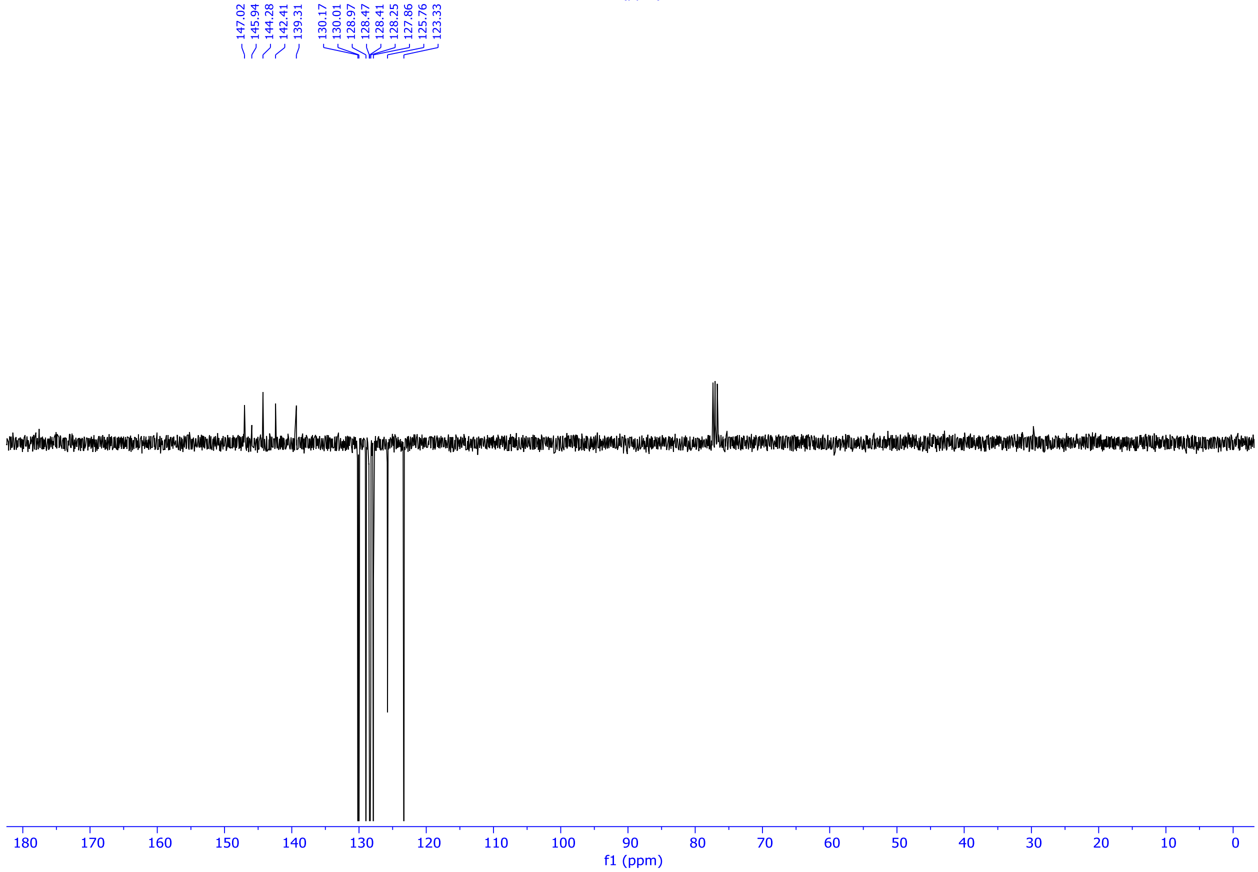

Figure S14. ^{1}H and ^{13}C (APT) NMR of **18**

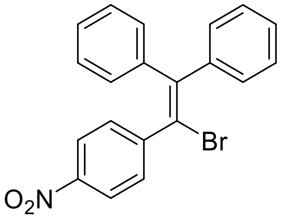

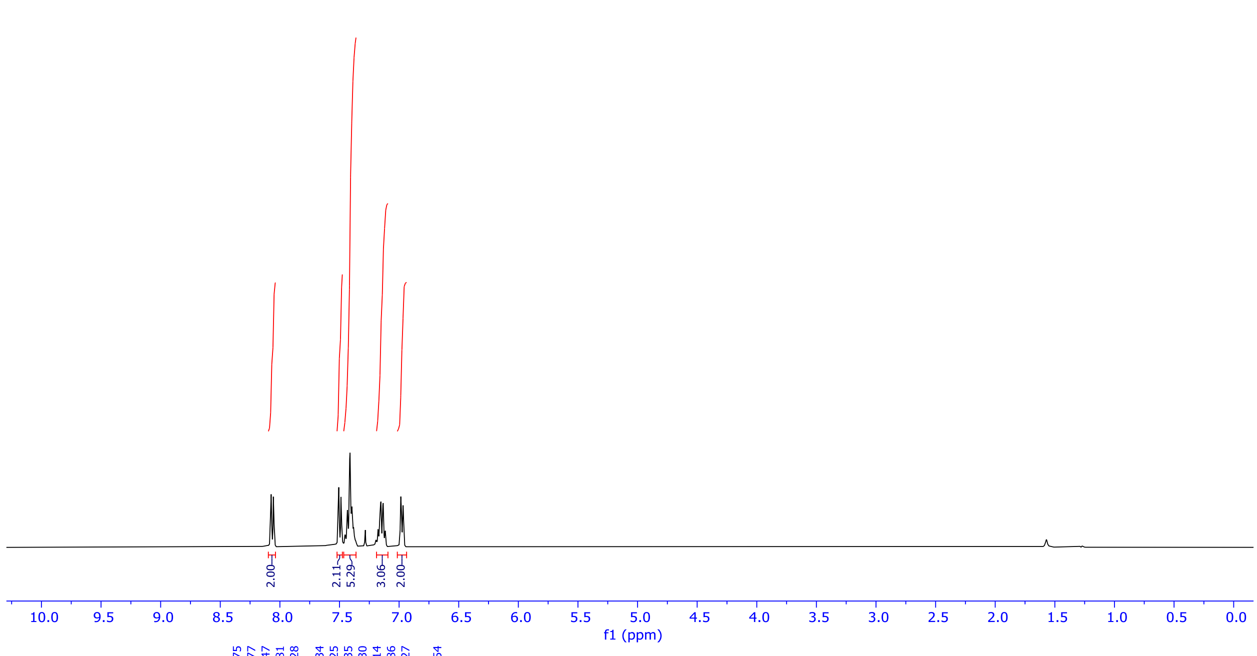

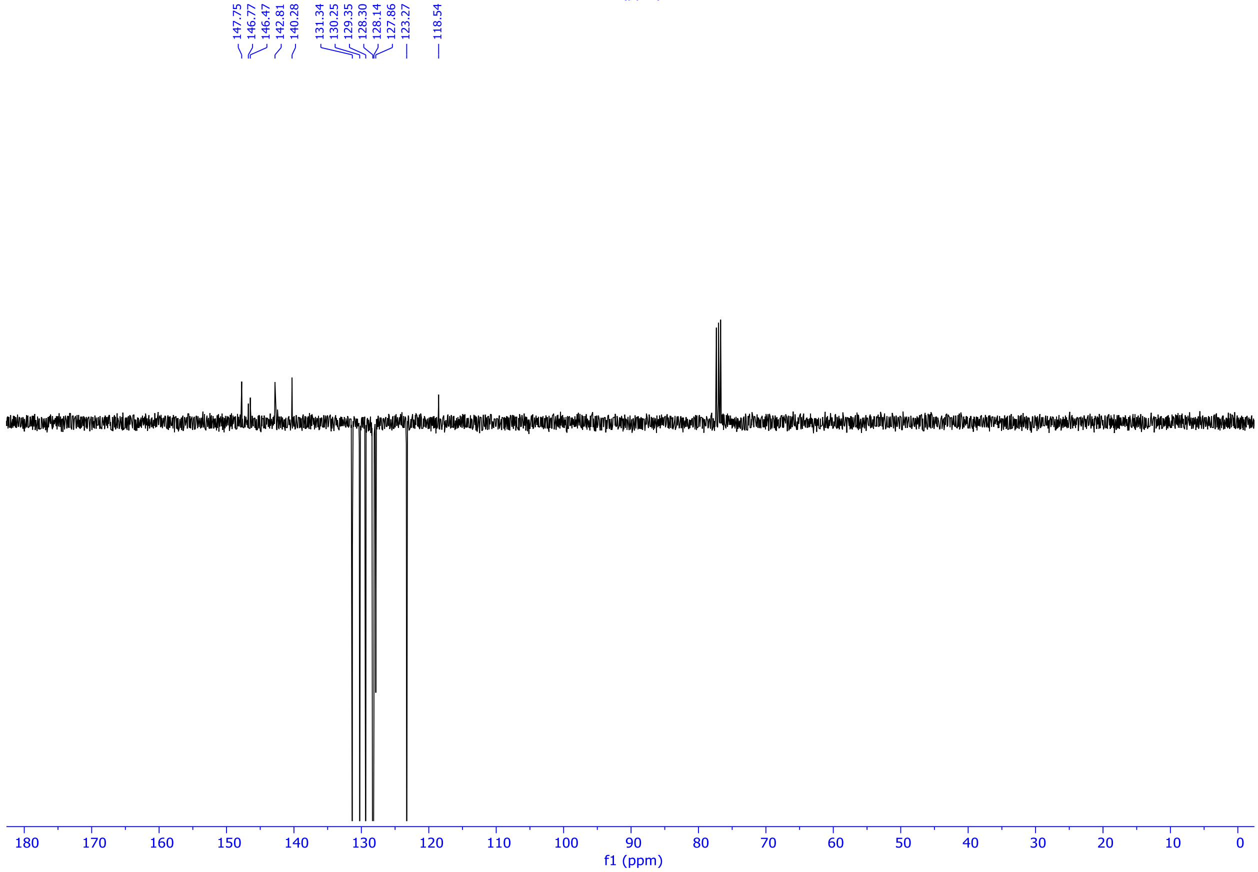

Figure S15. ^{1}H and ^{13}C (APT) NMR of **19**

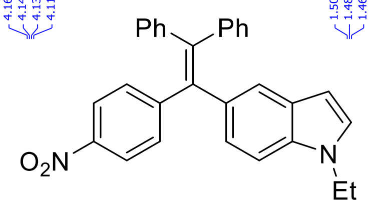

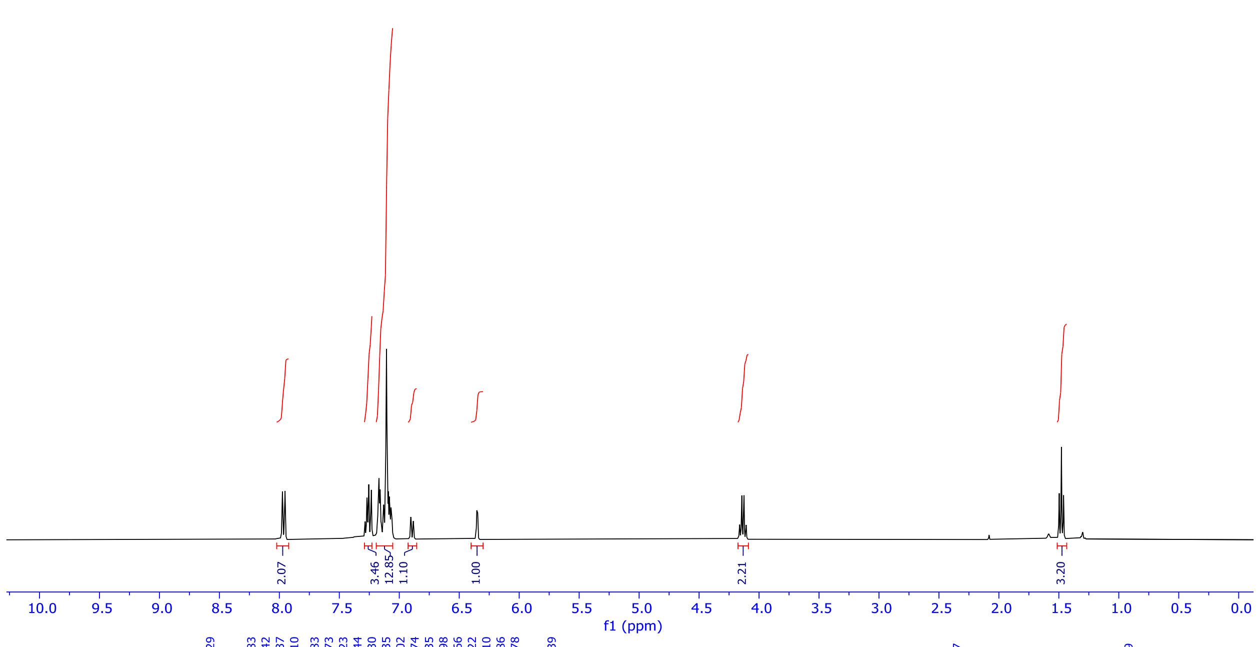

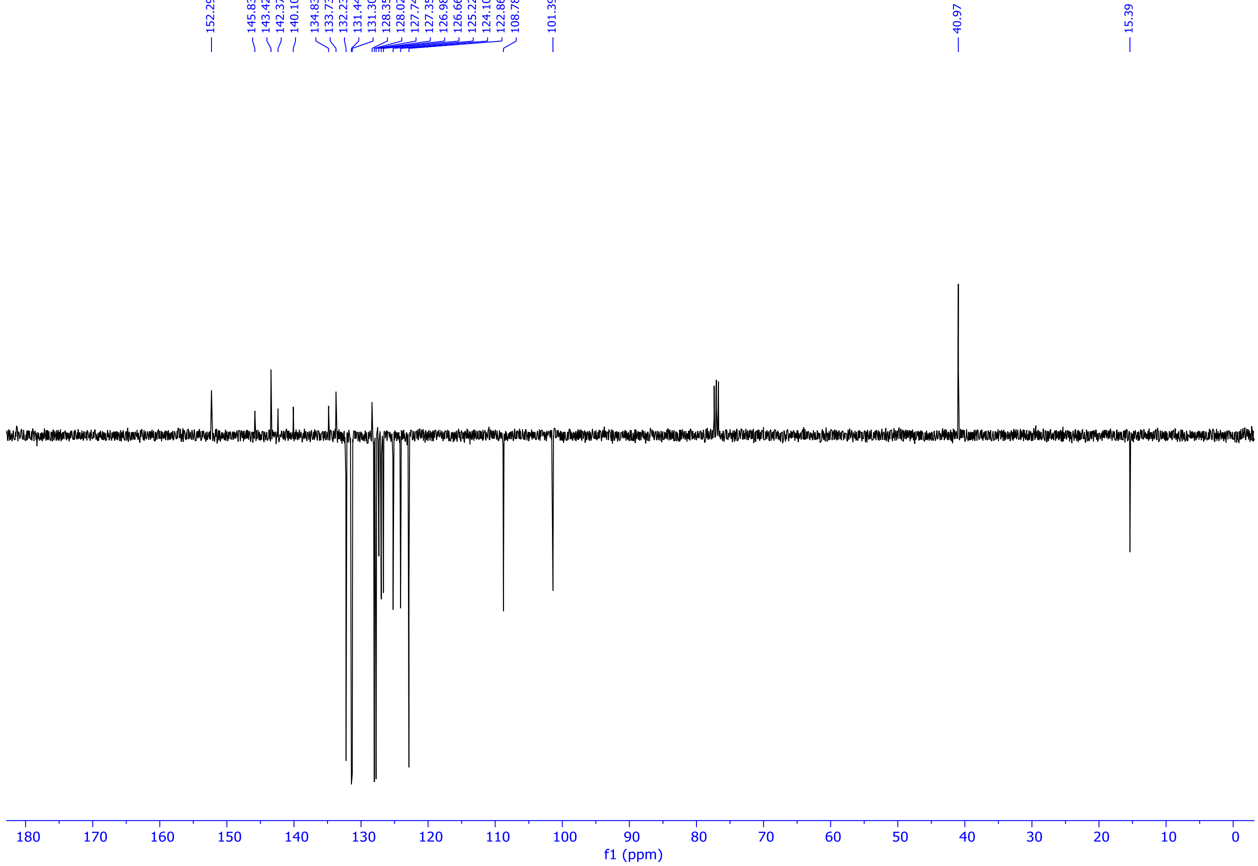

Figure S16. ^{1}H and ^{13}C (APT) NMR of **21**

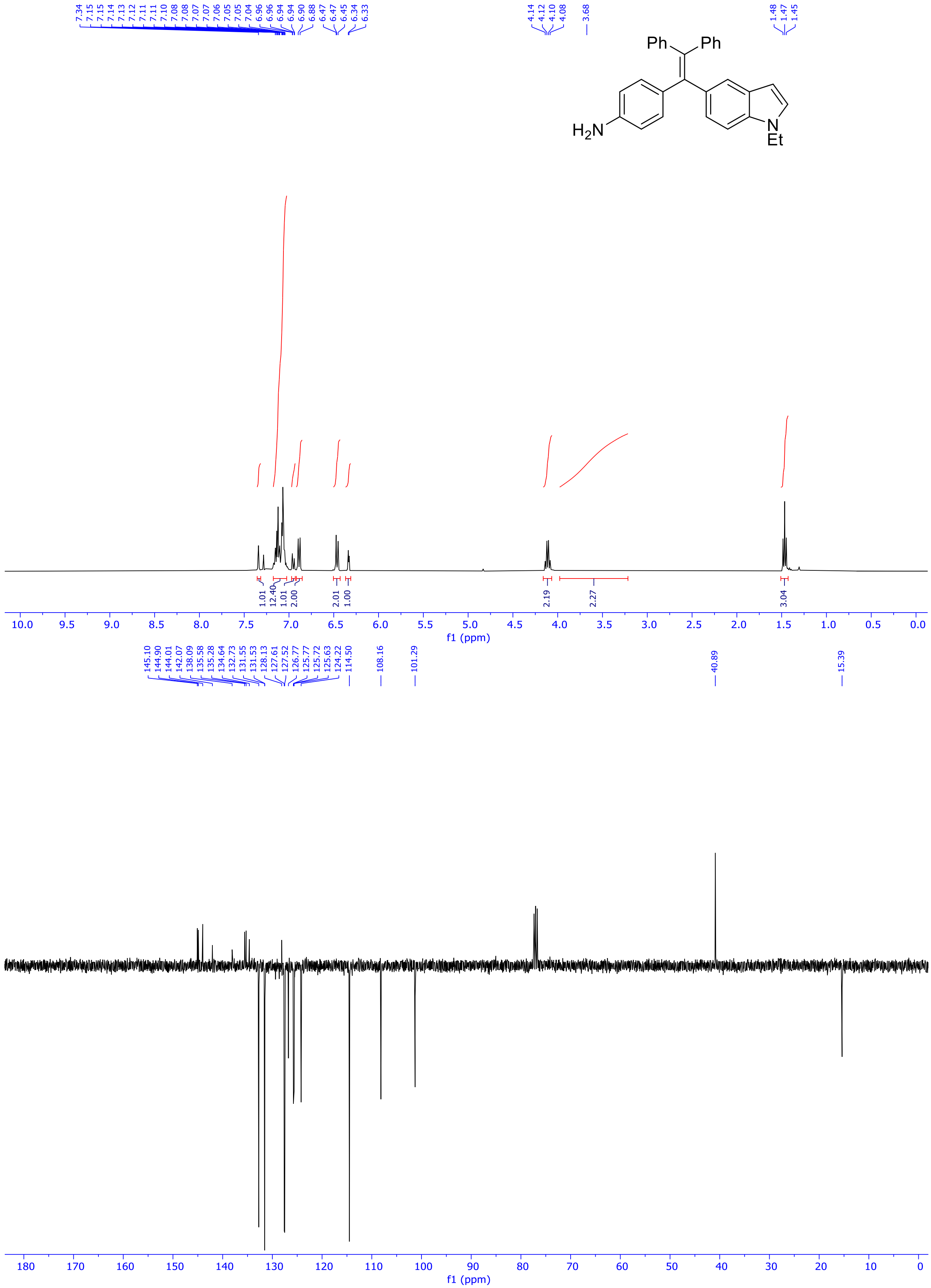

Figure S17. ^{1}H and ^{13}C (APT) NMR of **22**

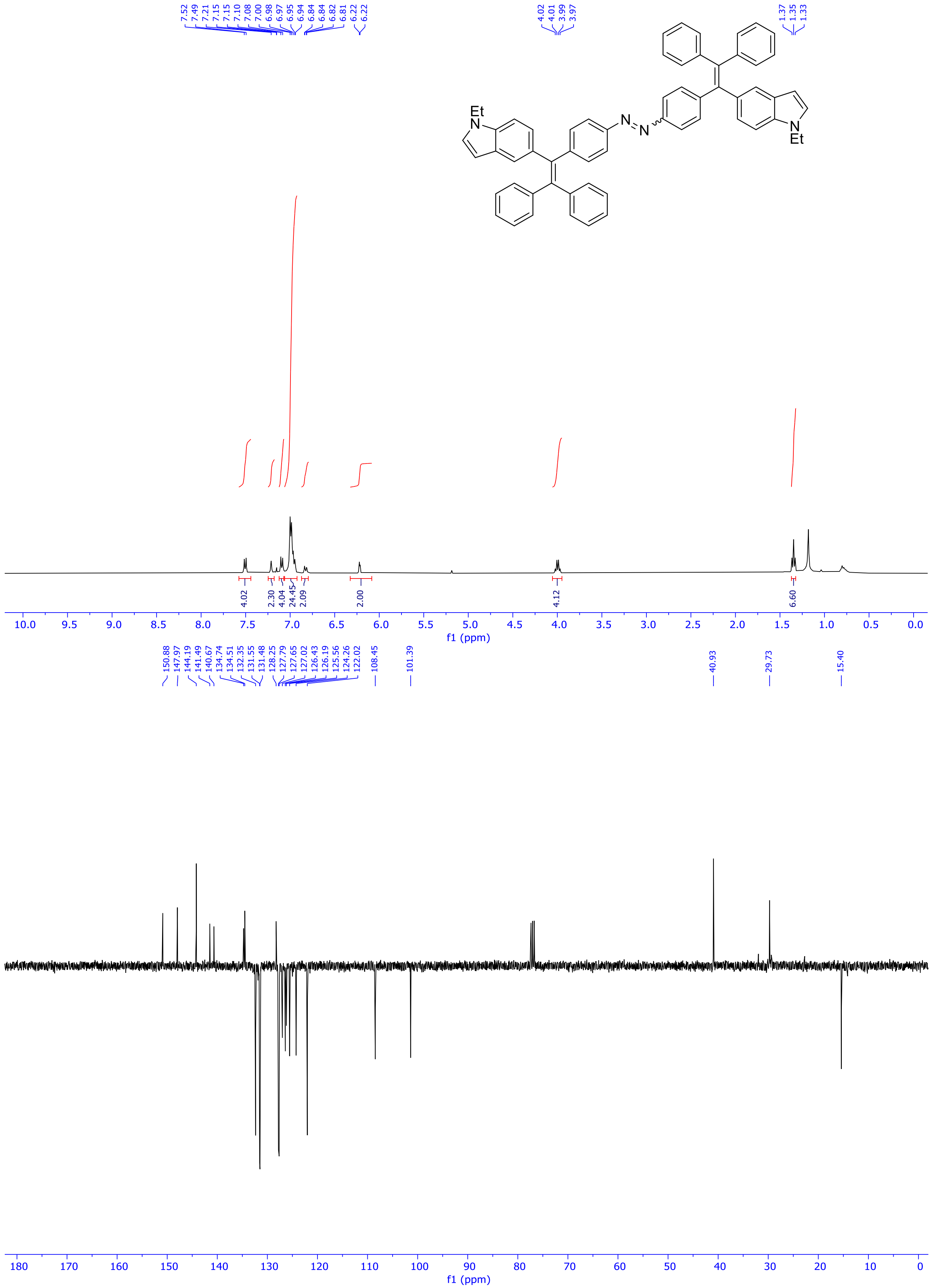

Figure S18. ^{1}H and ^{13}C (APT) NMR of **23**

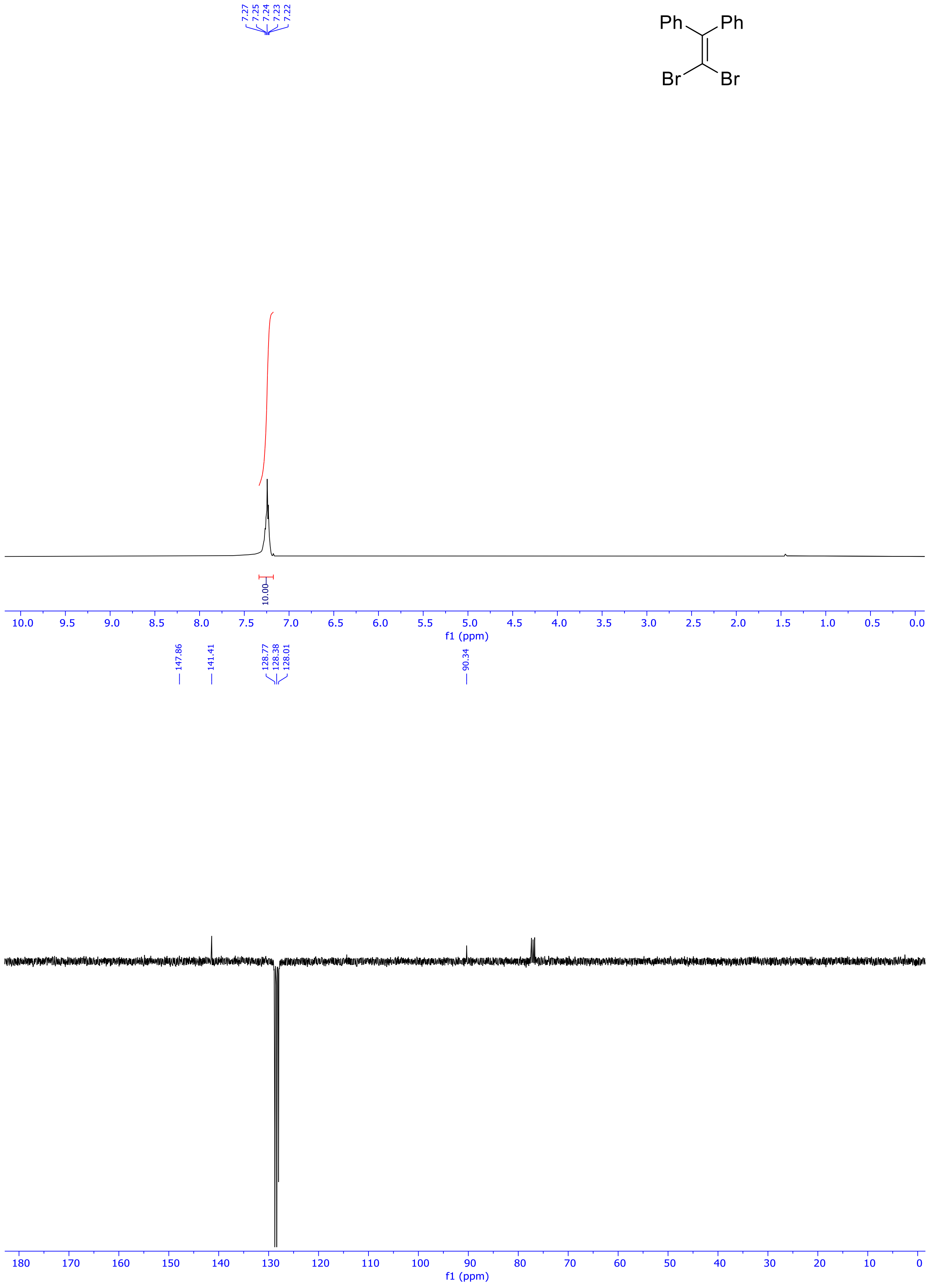

Figure S19. ^{1}H and ^{13}C (APT) NMR of **24**

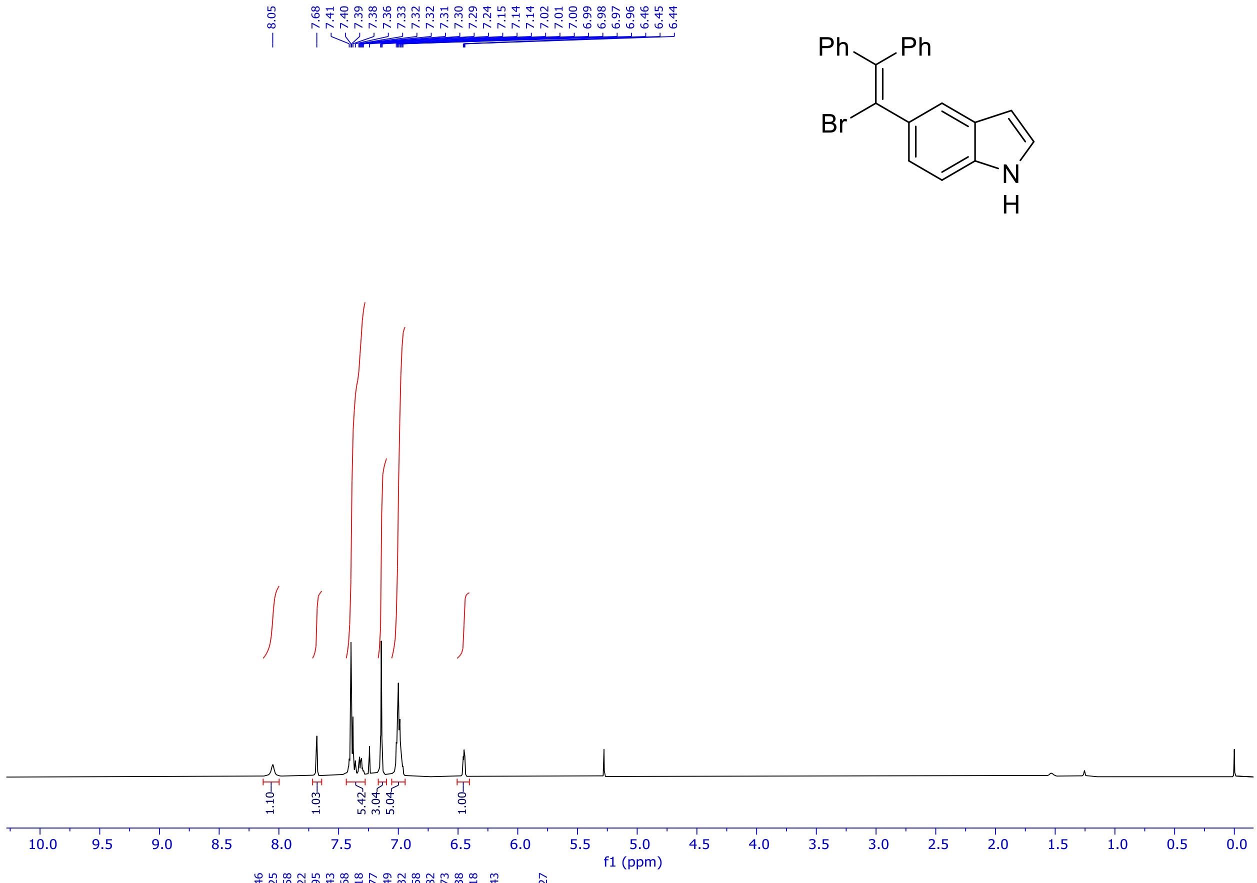

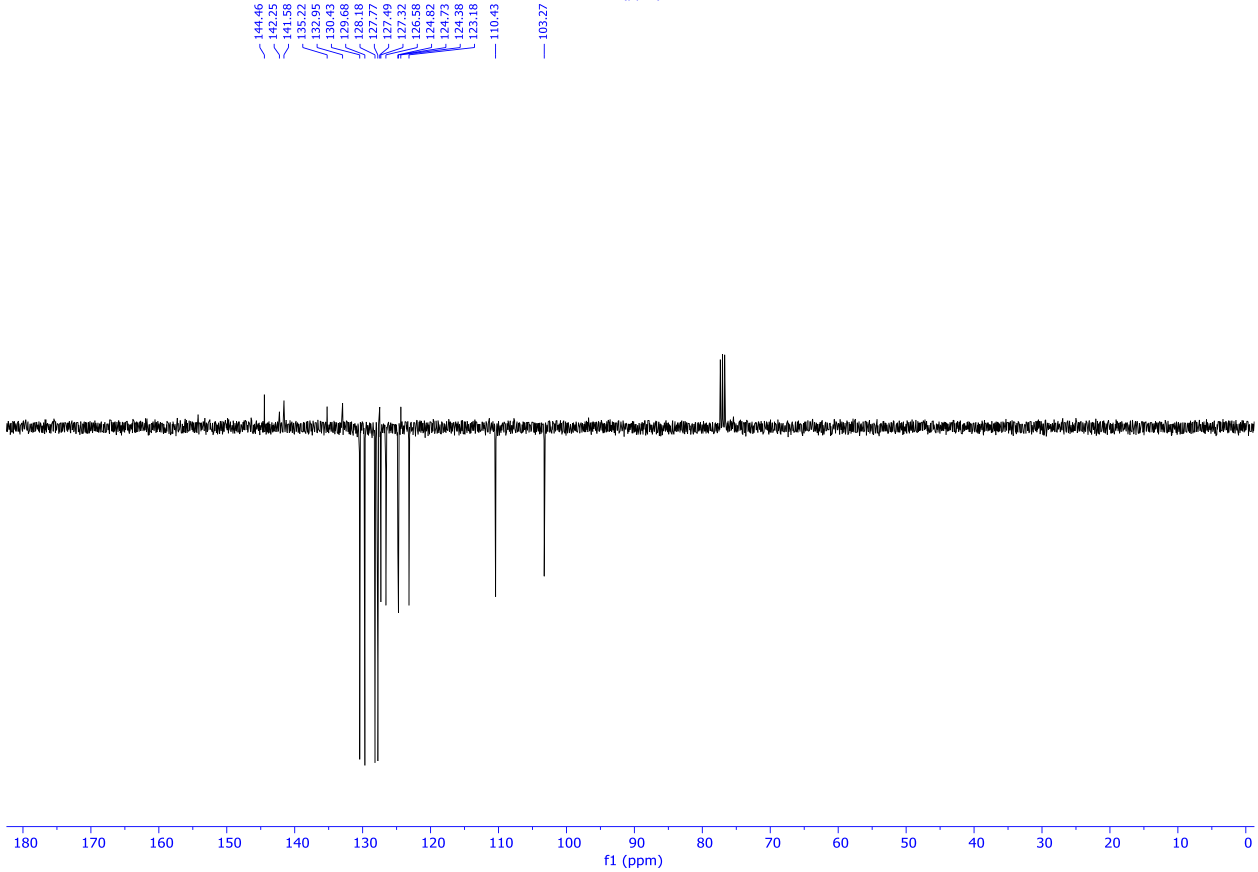

Figure S20. ^{1}H and ^{13}C (APT) NMR of **26**

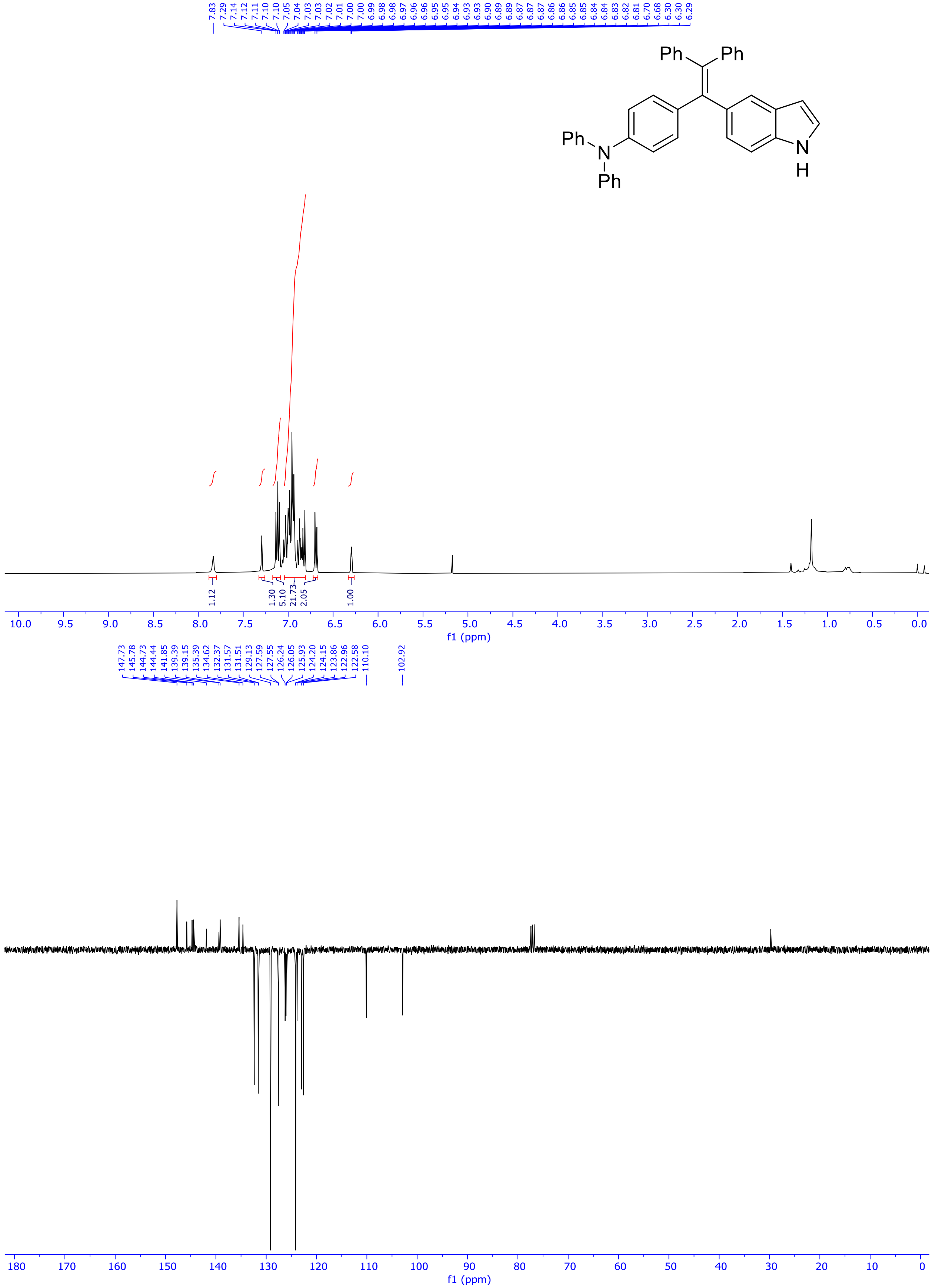

Figure S21. ^{1}H and ^{13}C (APT) NMR of **28**

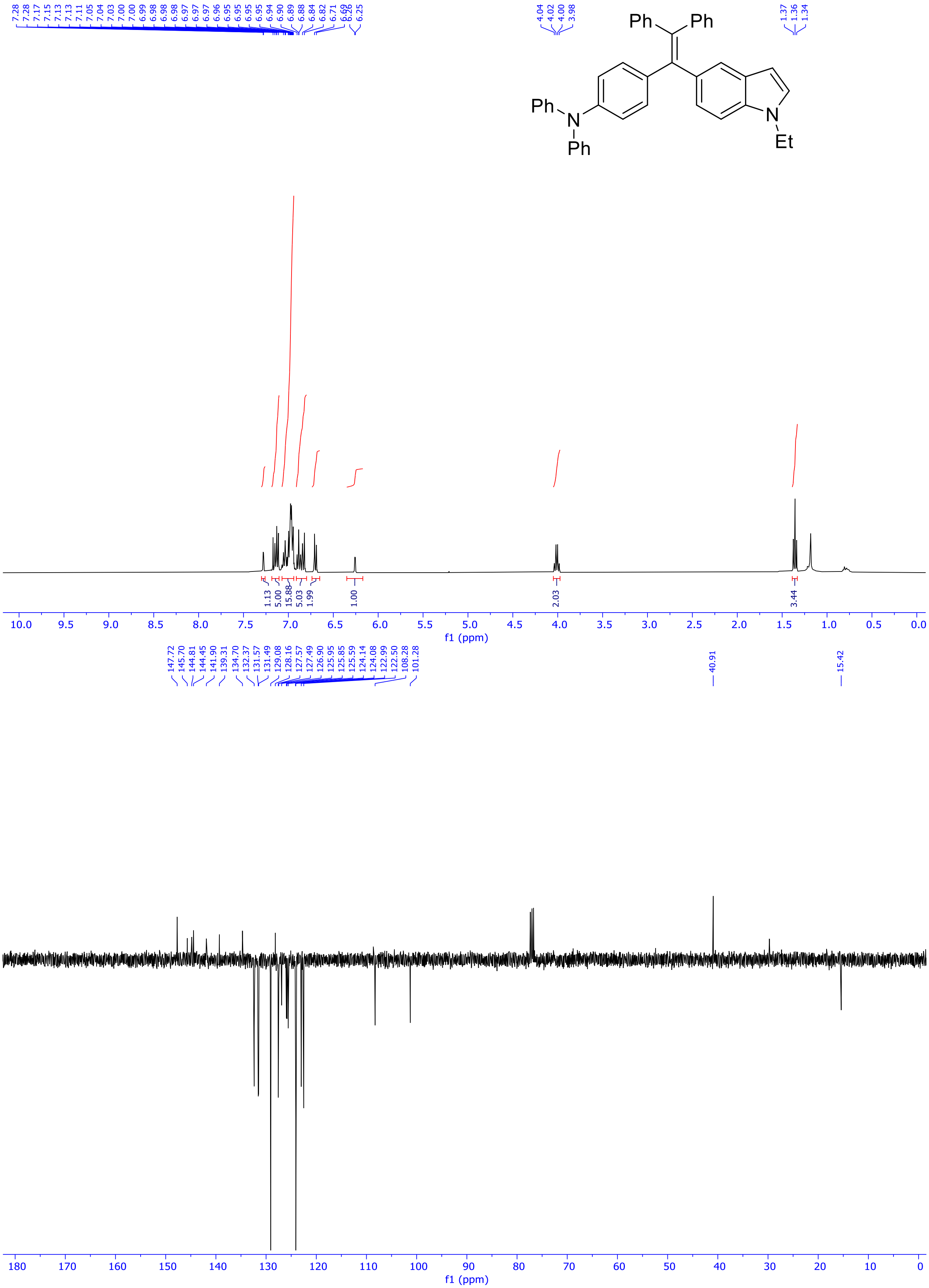

Figure S22. ^{1}H and ^{13}C (APT) NMR of **29**

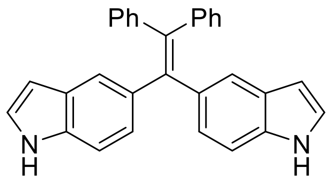

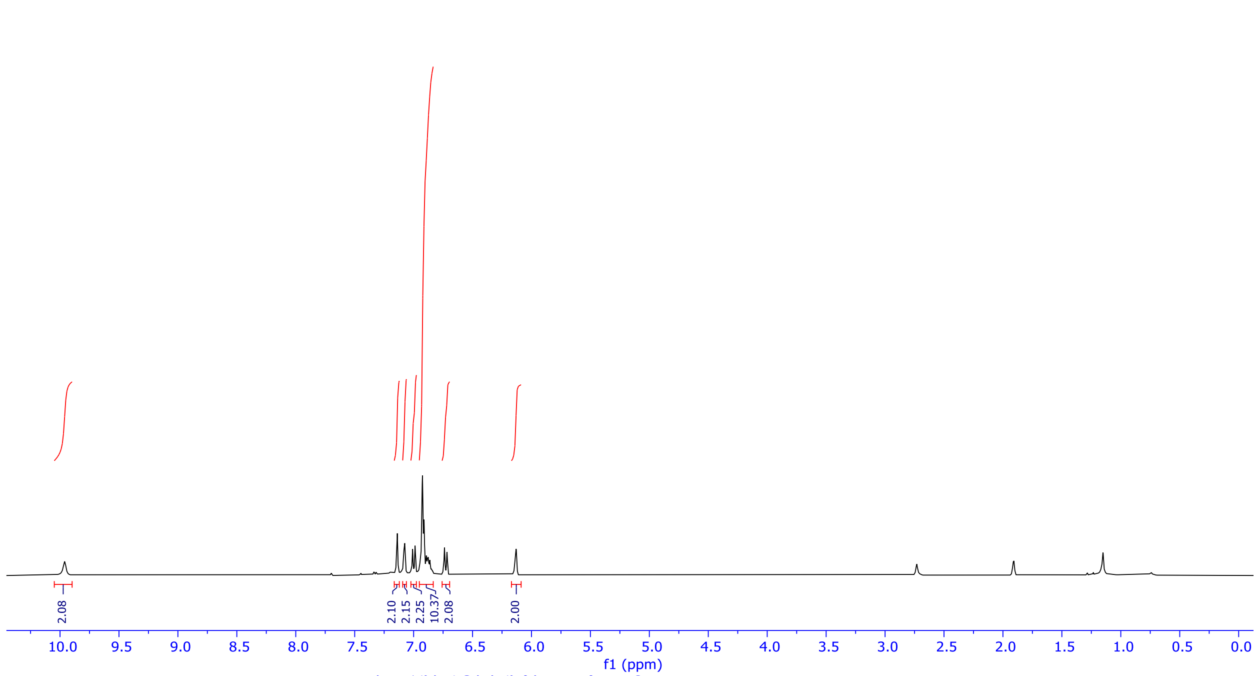

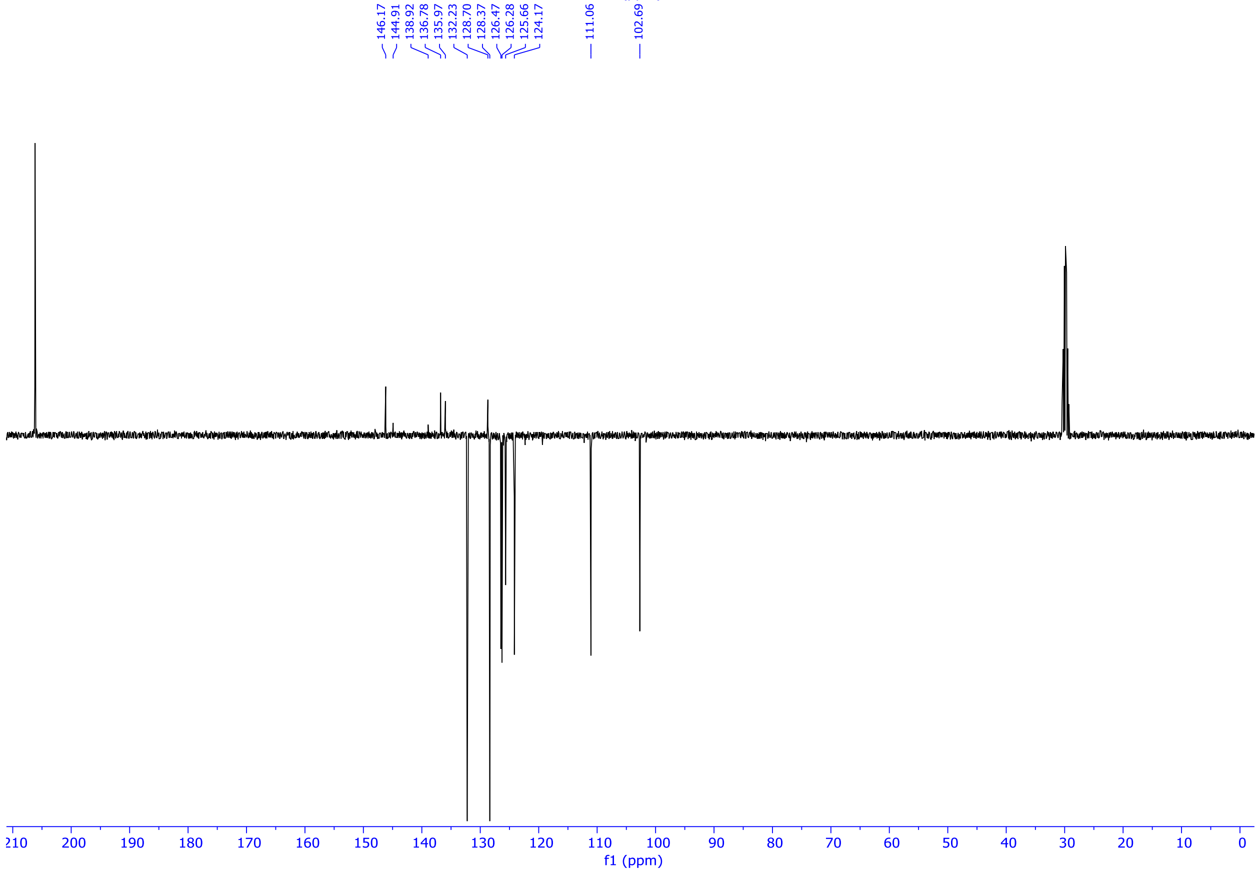

Figure S23. ^{1}H and ^{13}C (APT) NMR of **30**

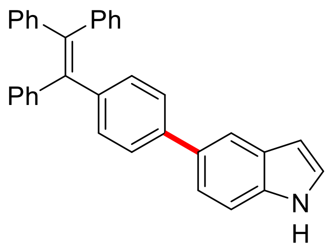

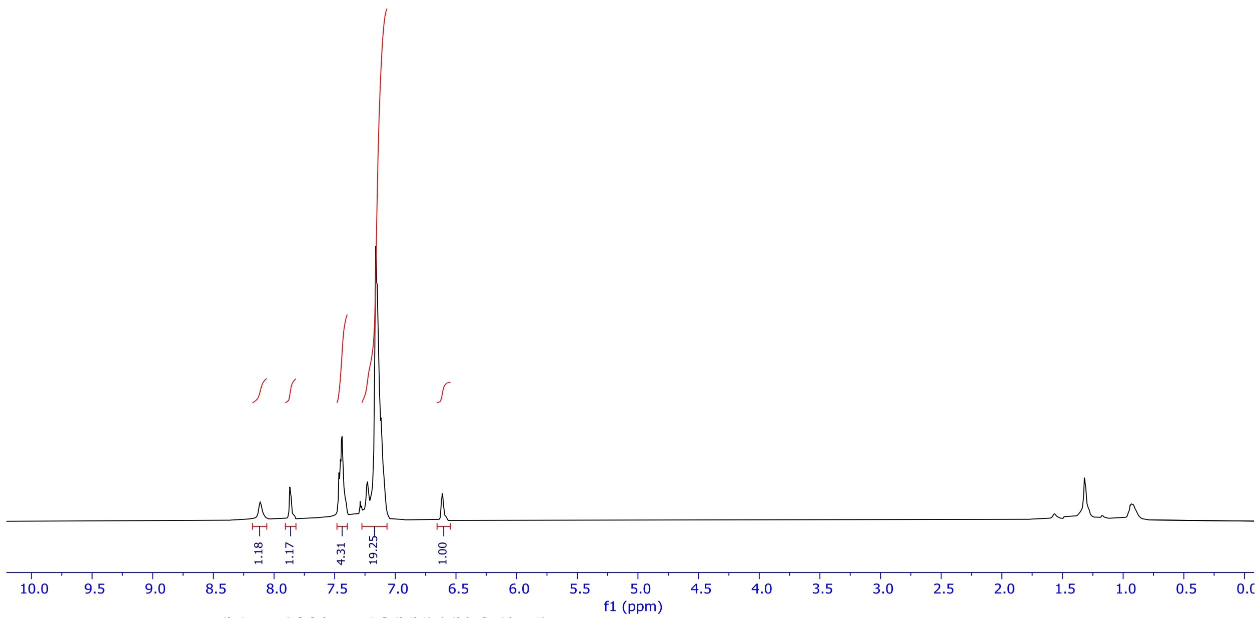

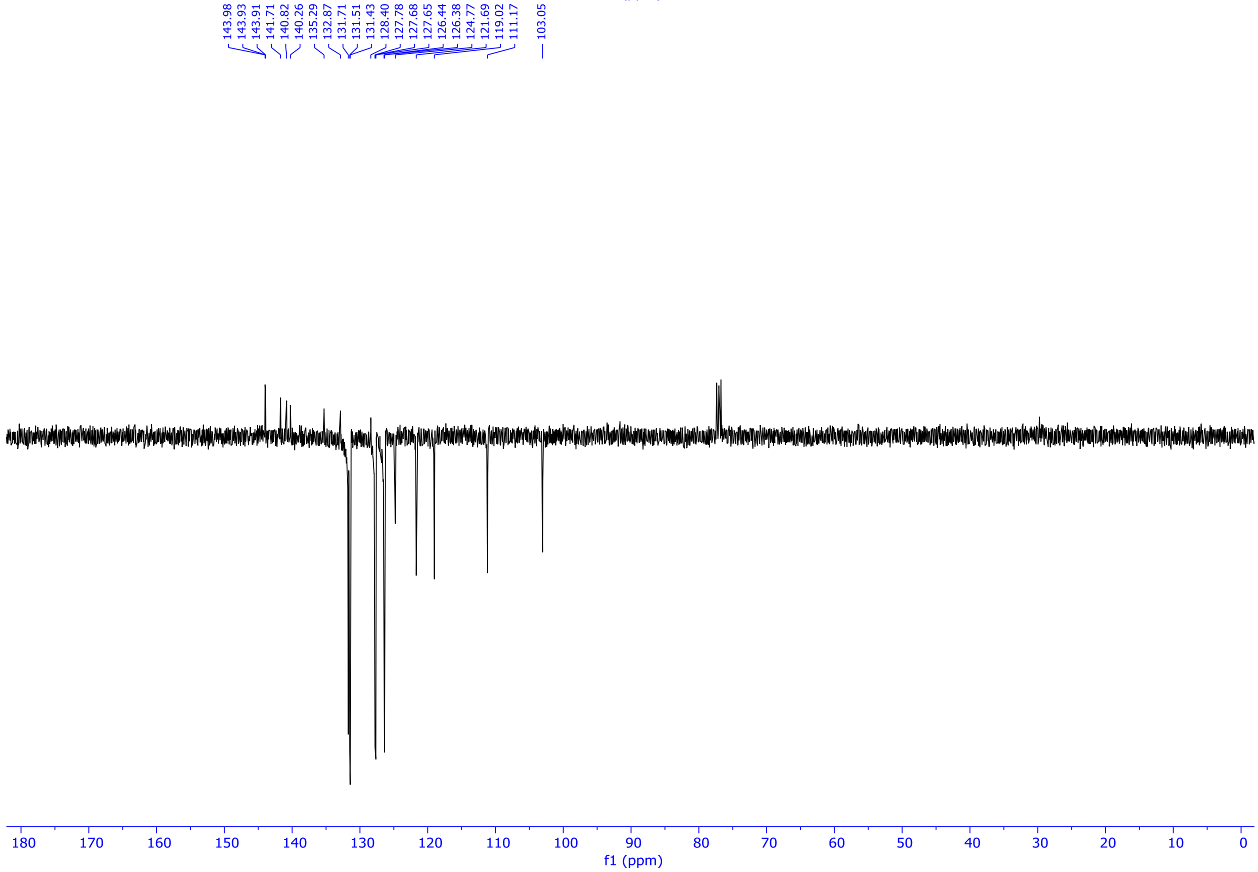

Figure S24. ^{1}H and ^{13}C (APT) NMR of **32**

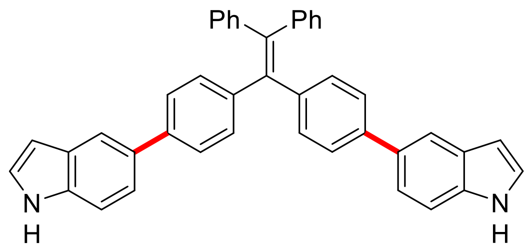

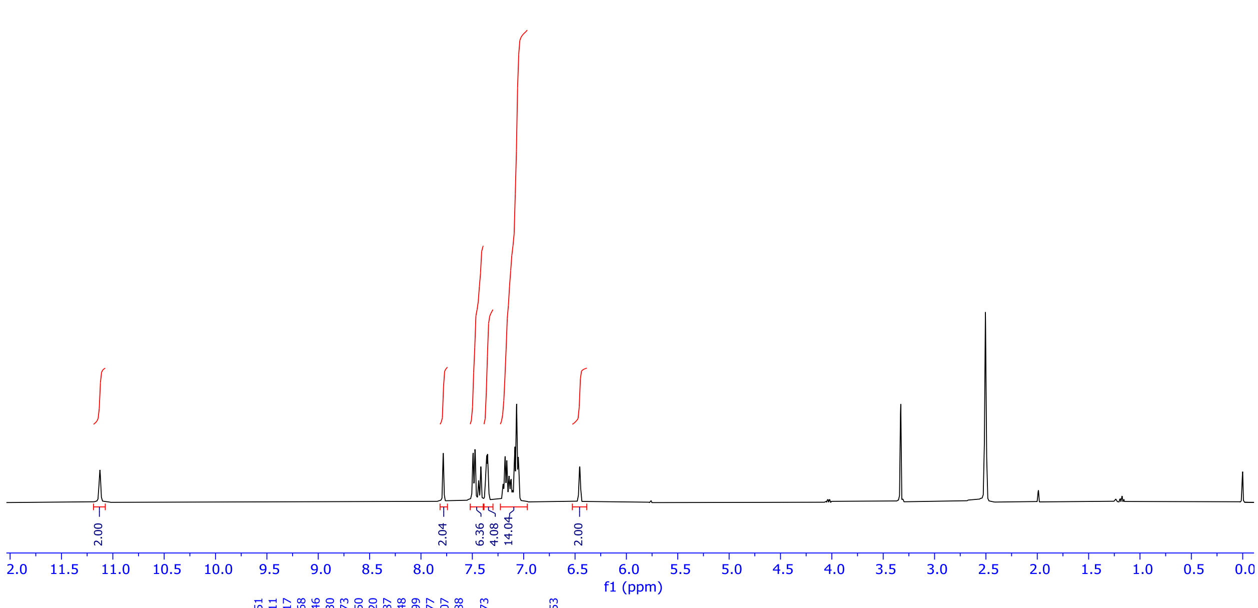

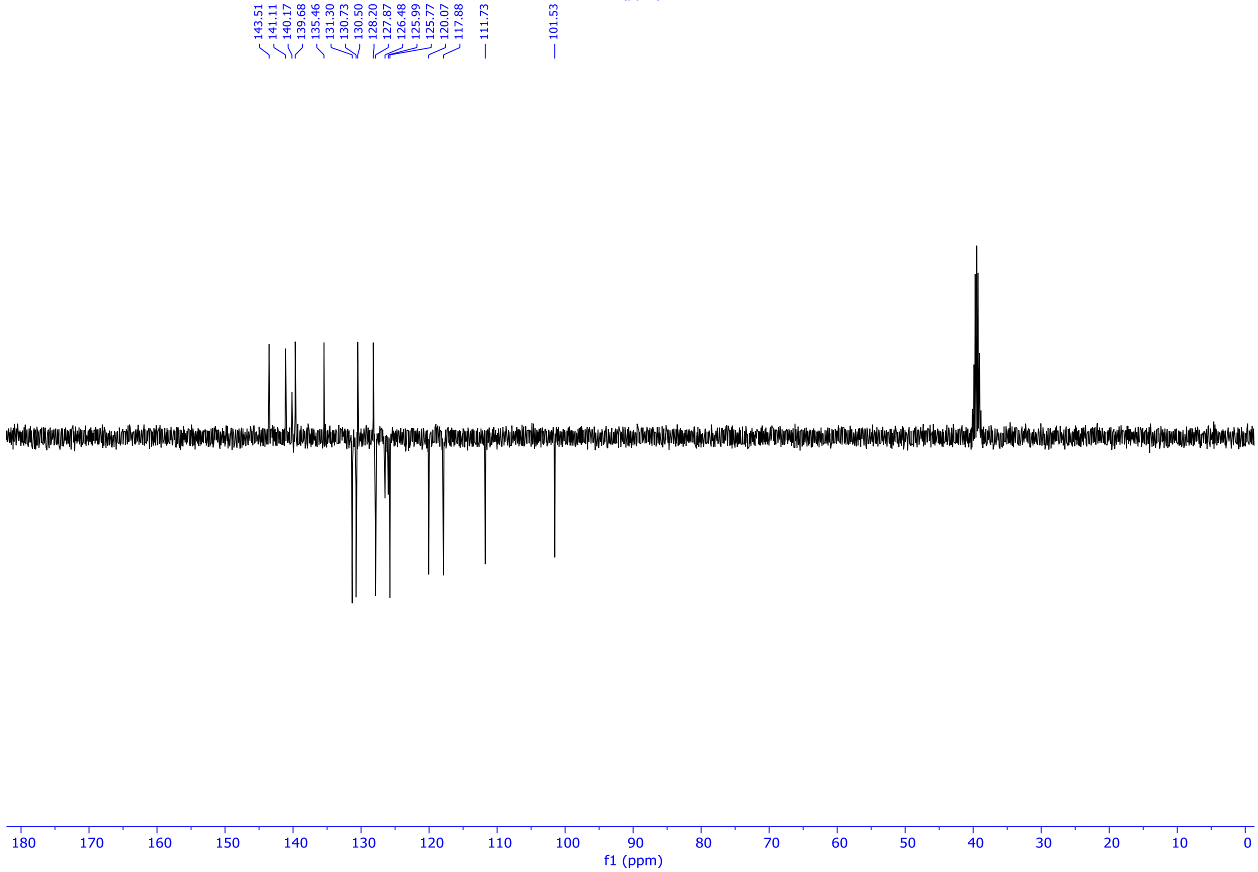

Figure S25. ^{1}H and ^{13}C (APT) NMR of **33**

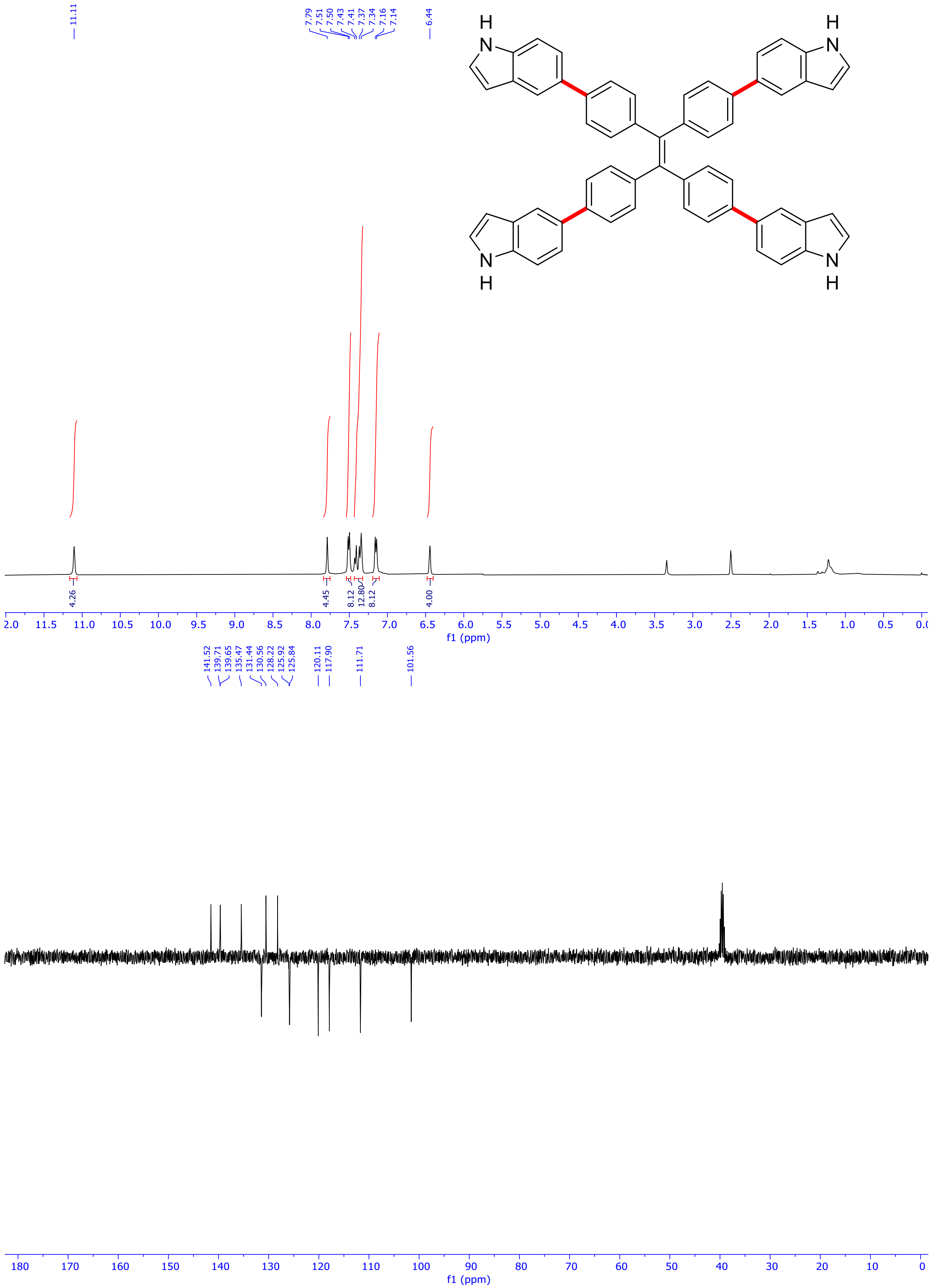

Figure S26. ^{1}H and ^{13}C (APT) NMR of **34**

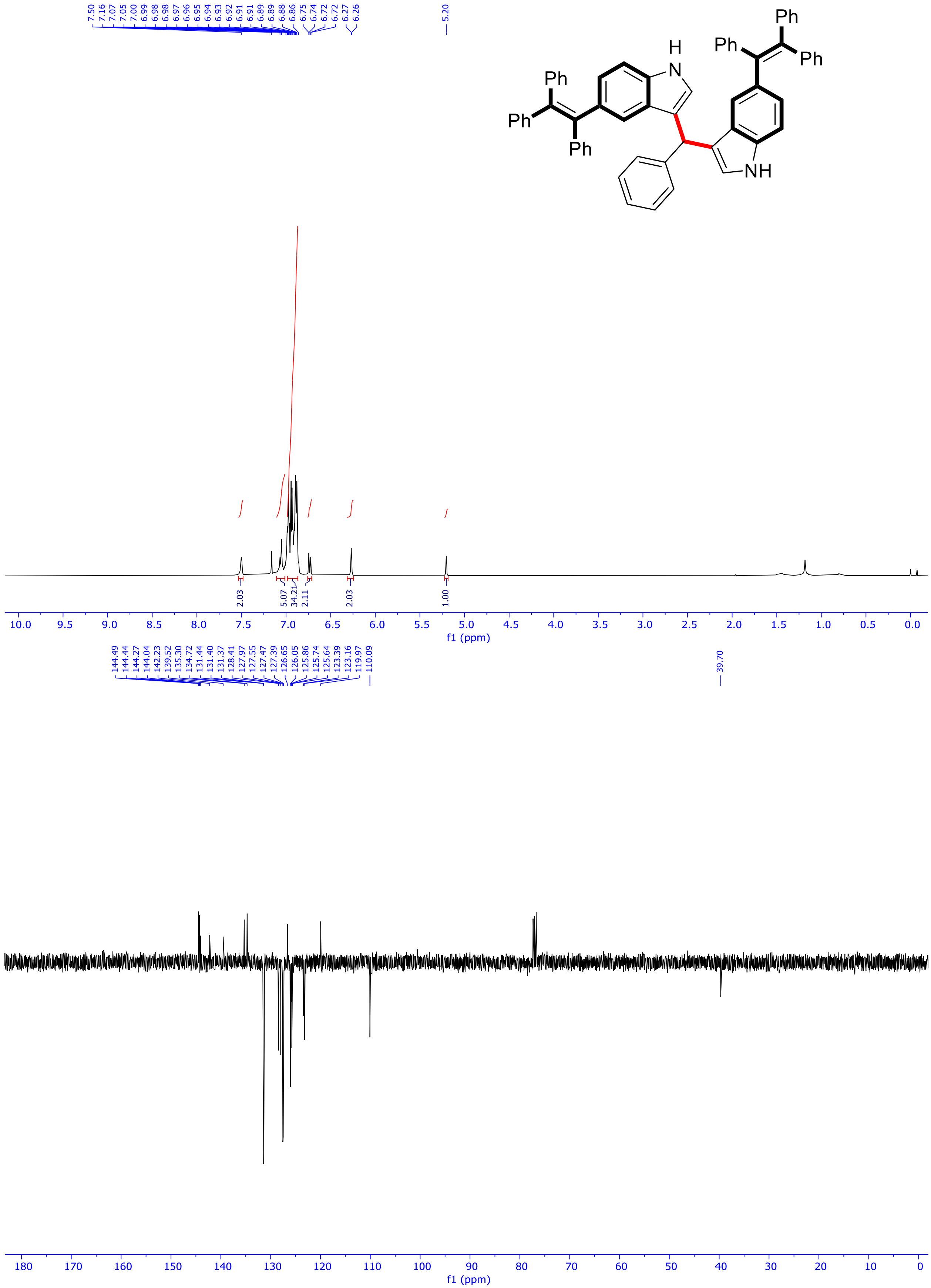

Figure S27. ^{1}H and ^{13}C (APT) NMR of **36a**

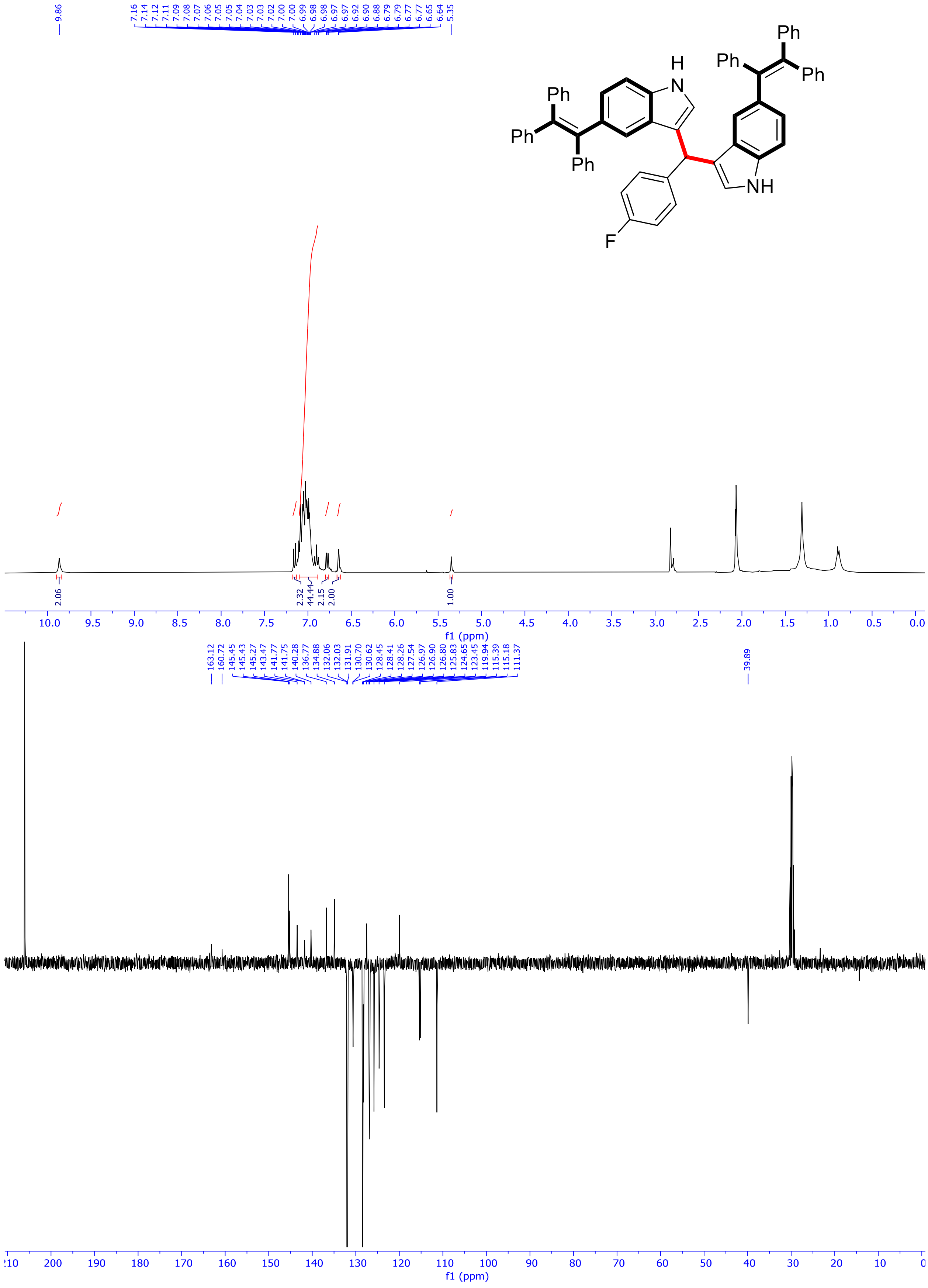

Figure S28. ^{1}H and ^{13}C (APT) NMR of **36b**

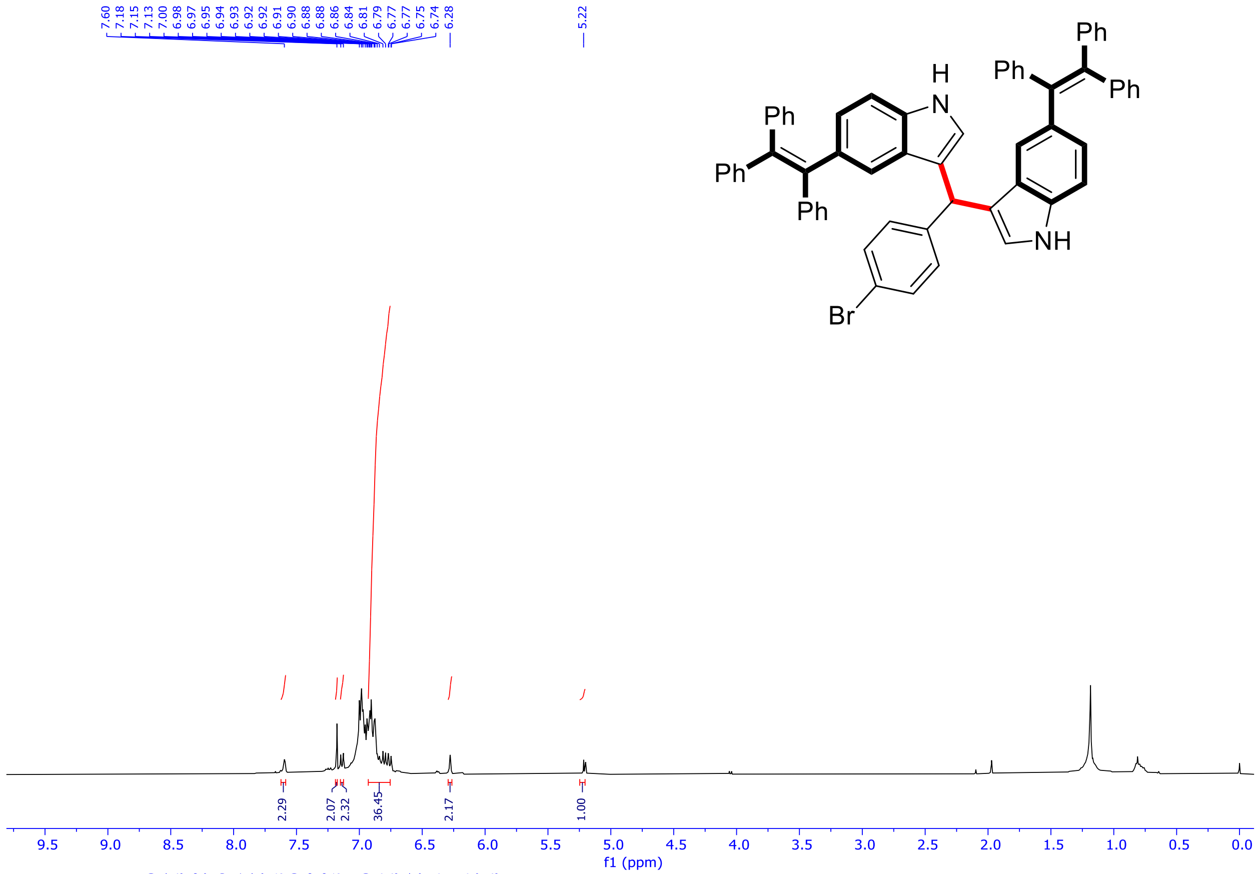

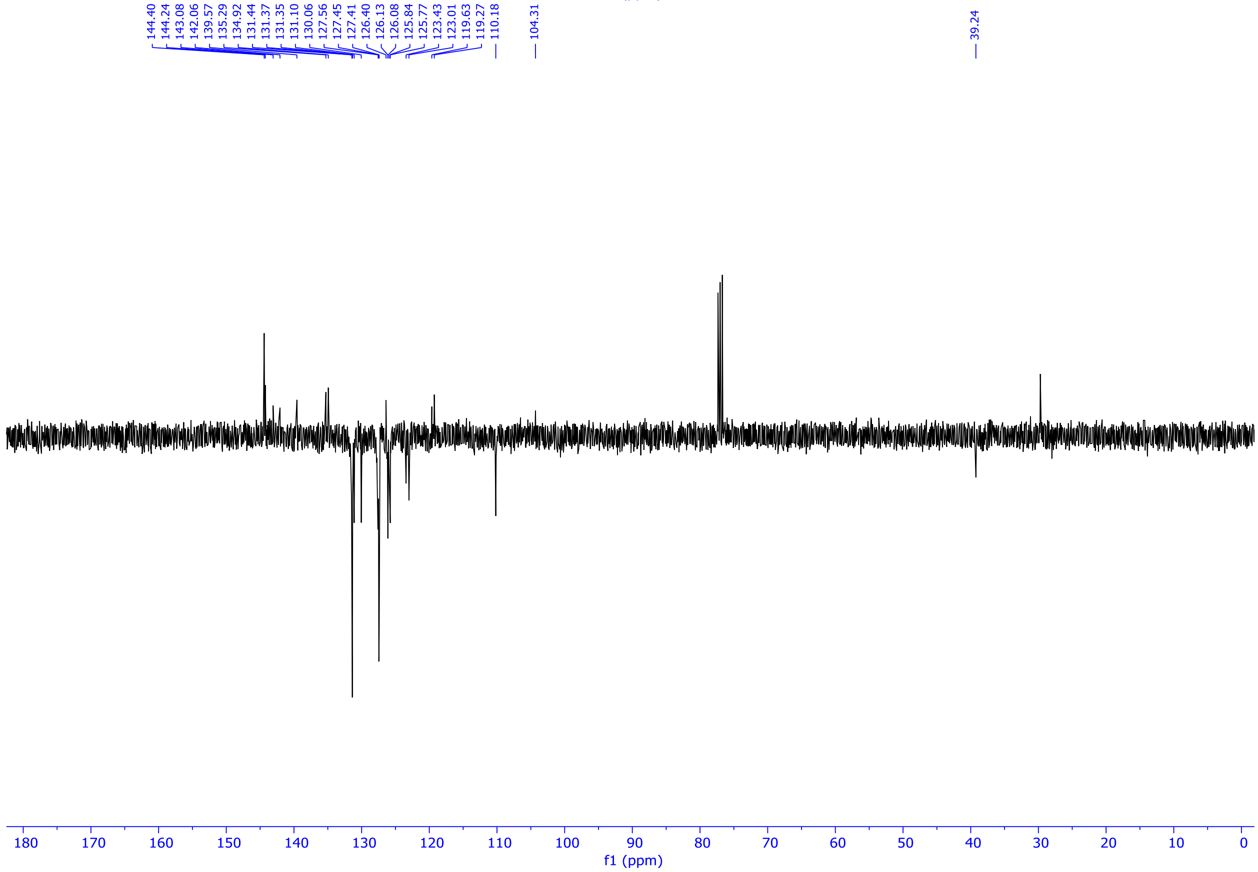

Figure S29. ^{1}H and ^{13}C (APT) NMR of **36c**

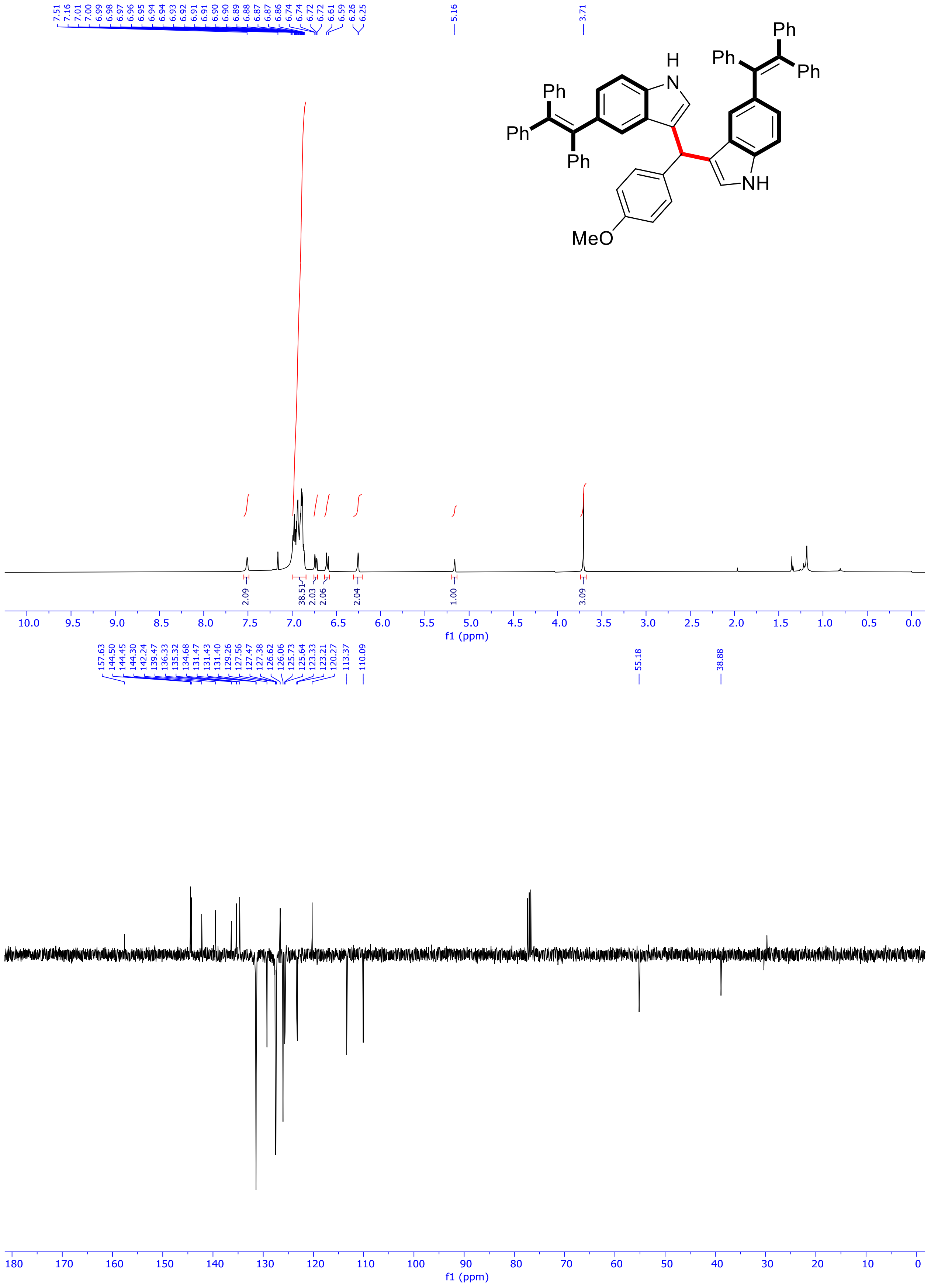

Figure S30. ^{1}H and ^{13}C (APT) NMR of **36d**

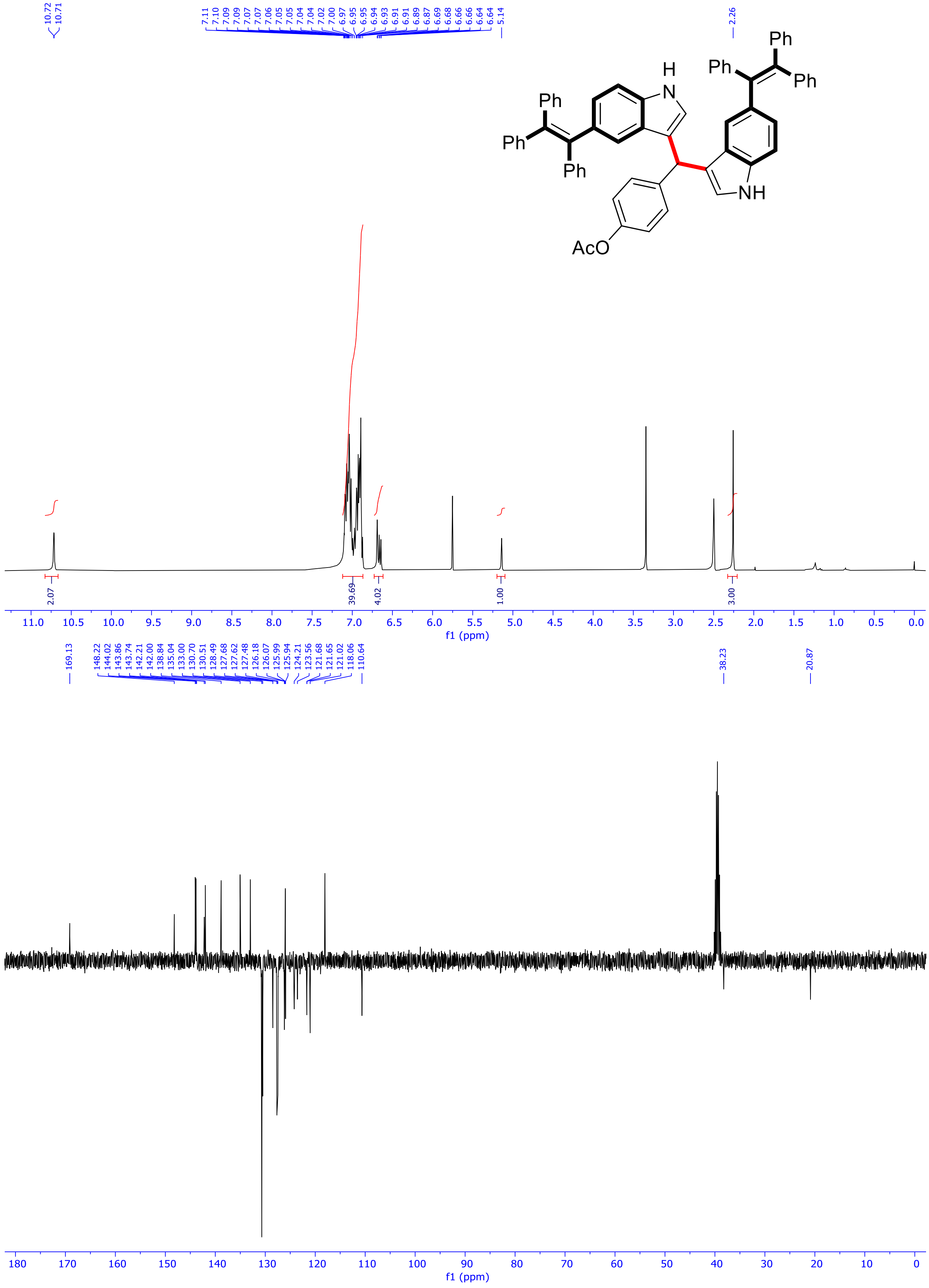

Figure S31. ^{1}H and ^{13}C (APT) NMR of **36e**

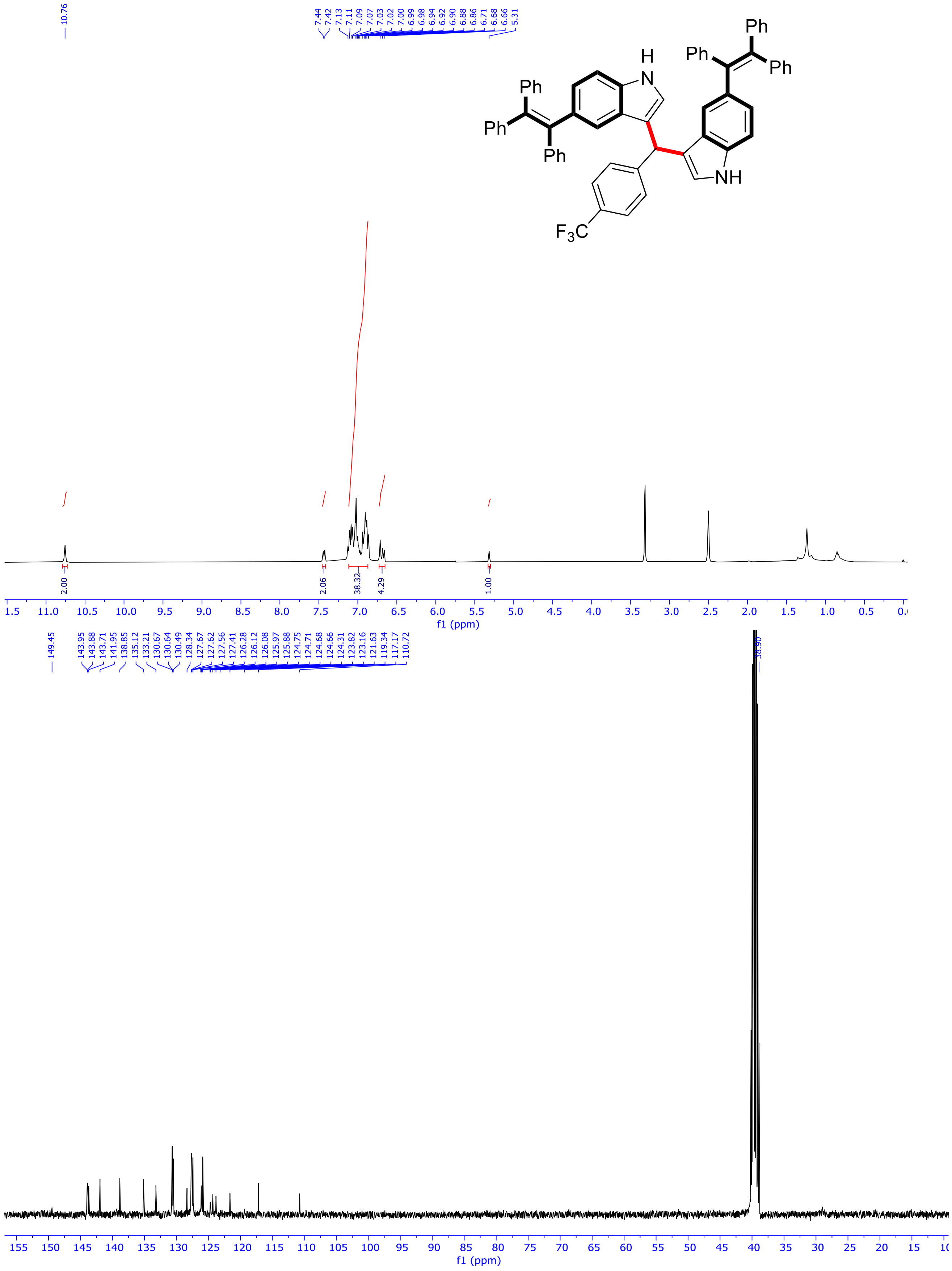

Figure S32. ^{1}H and ^{13}C NMR of **36f**

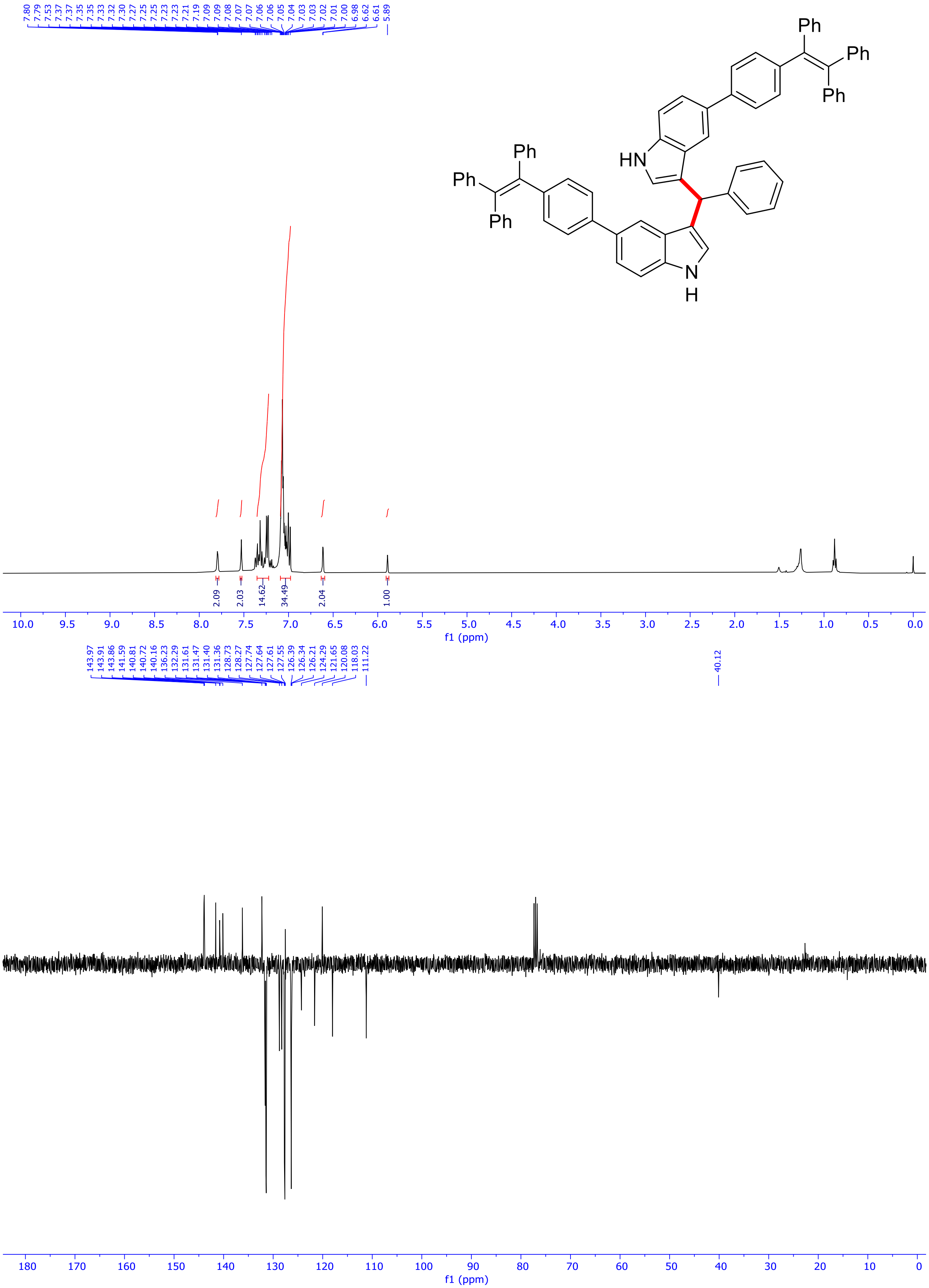

Figure S33. ^{1}H and ^{13}C (APT) NMR of **37a**

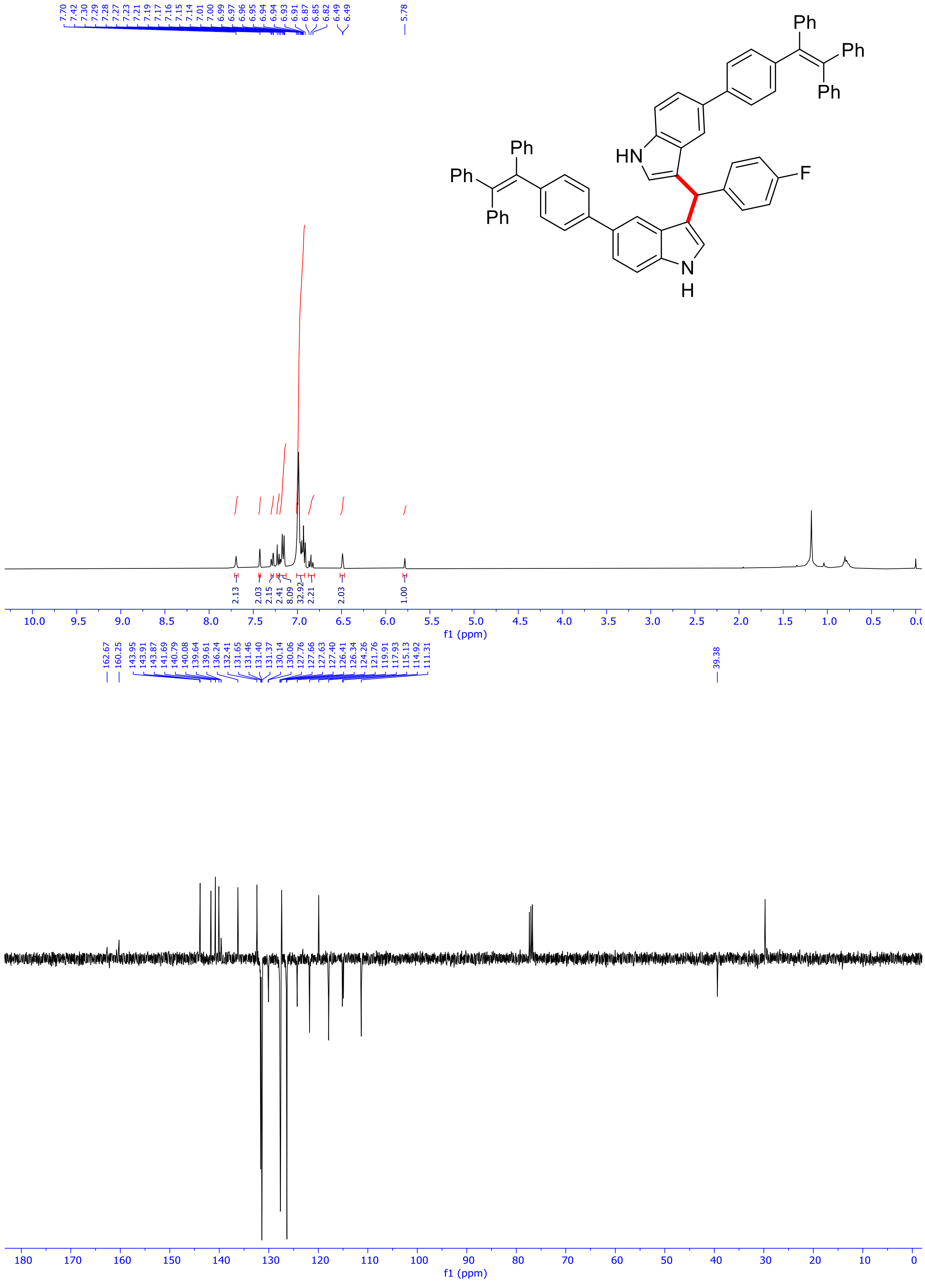

Figure S34. ^{1}H and ^{13}C (APT) NMR of **37b**

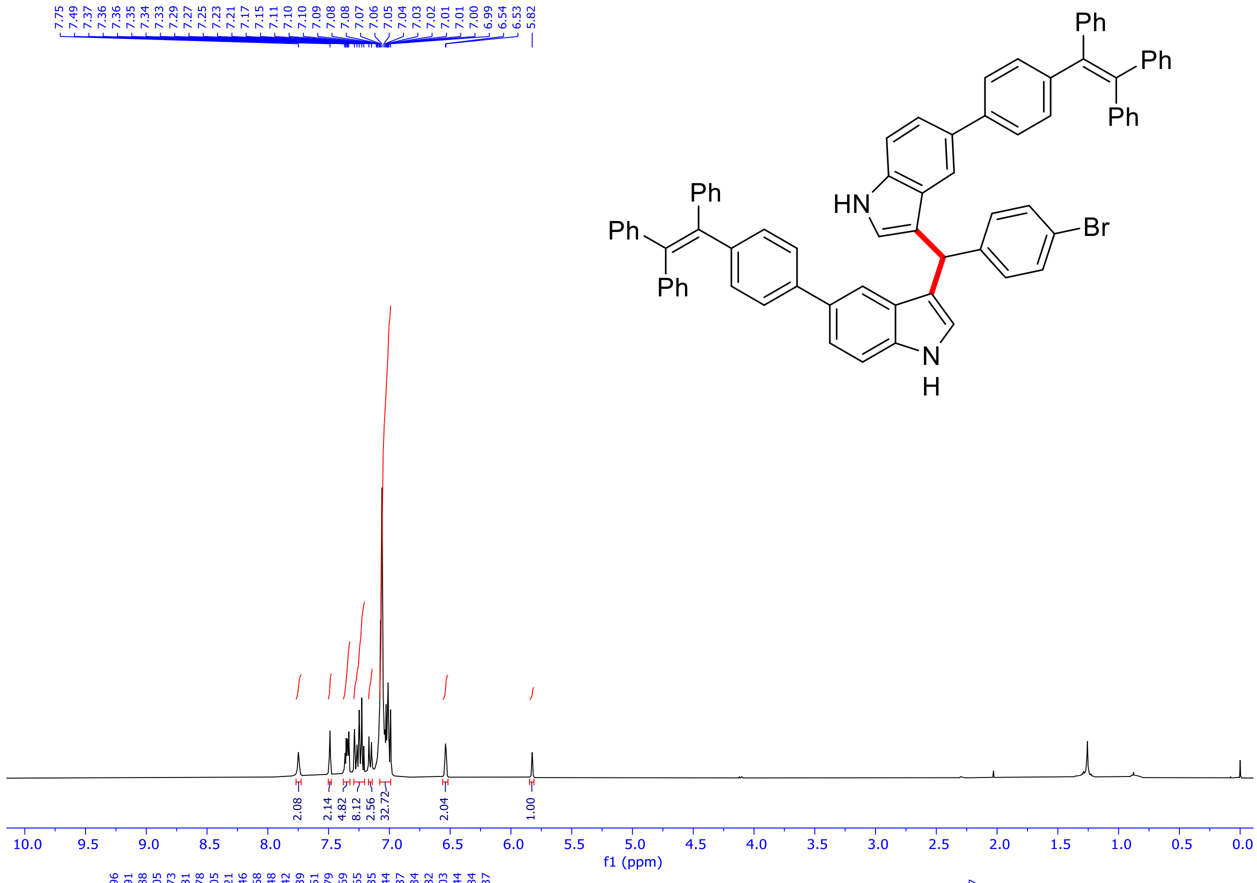

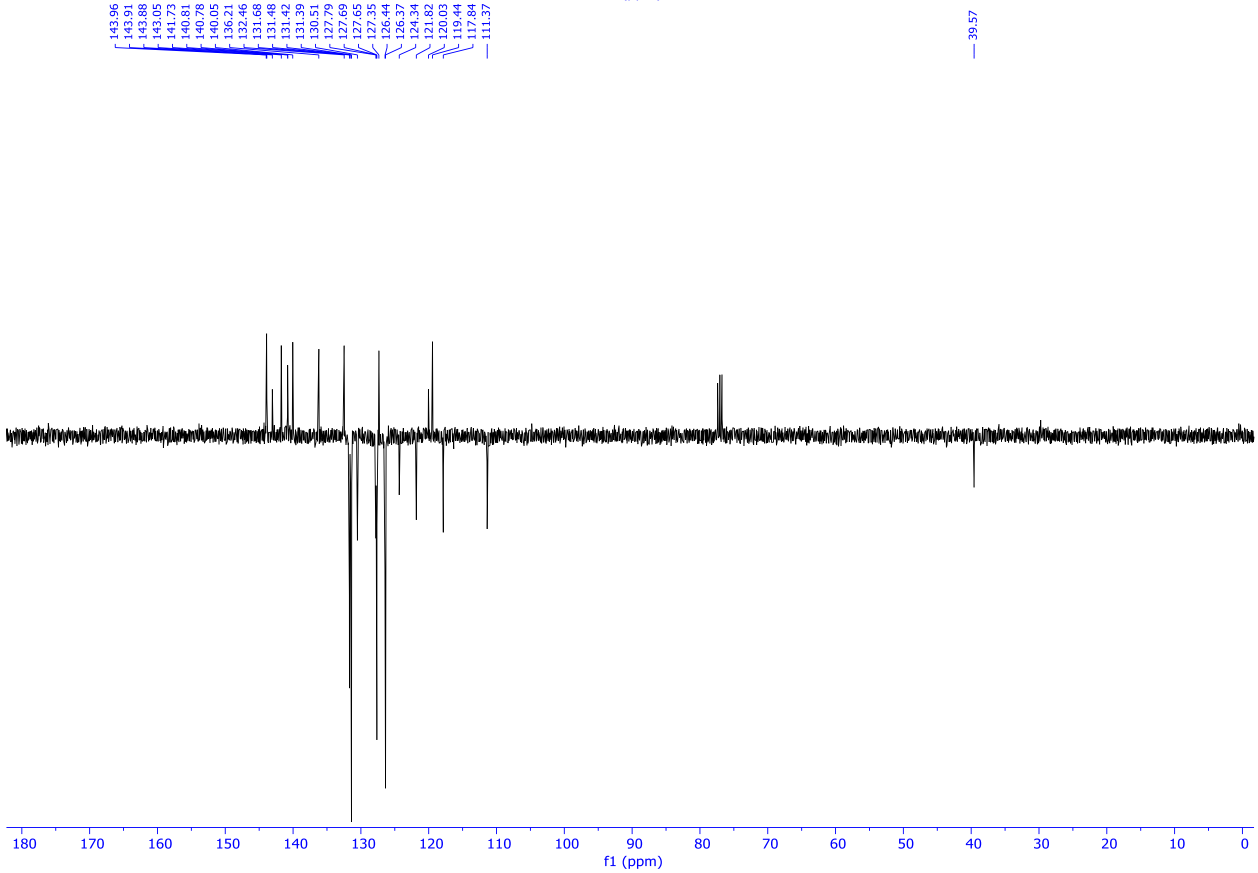

Figure S35. ^{1}H and ^{13}C (APT) NMR of **37c**

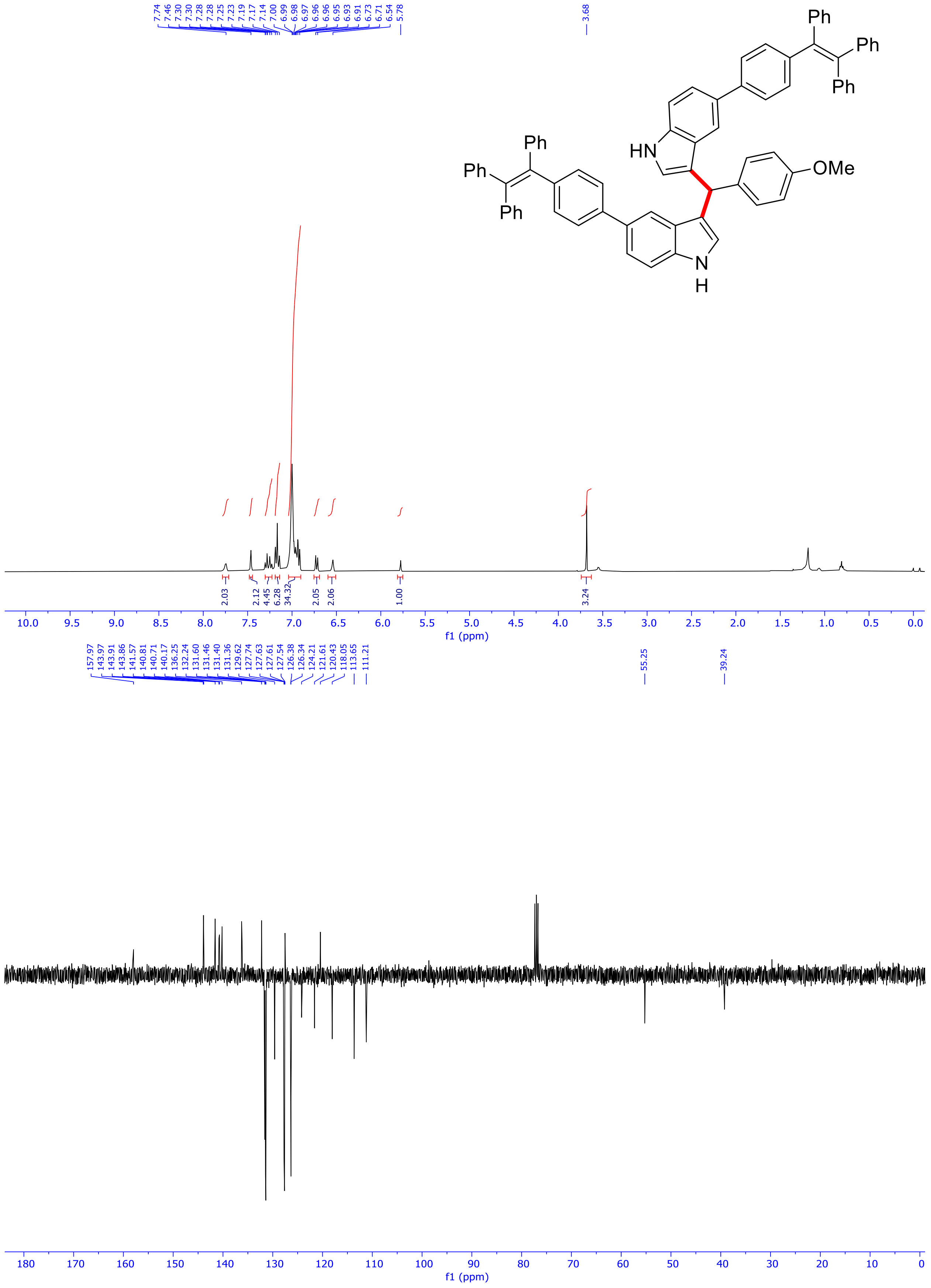

Figure S36. ^{1}H and ^{13}C (APT) NMR of **37d**

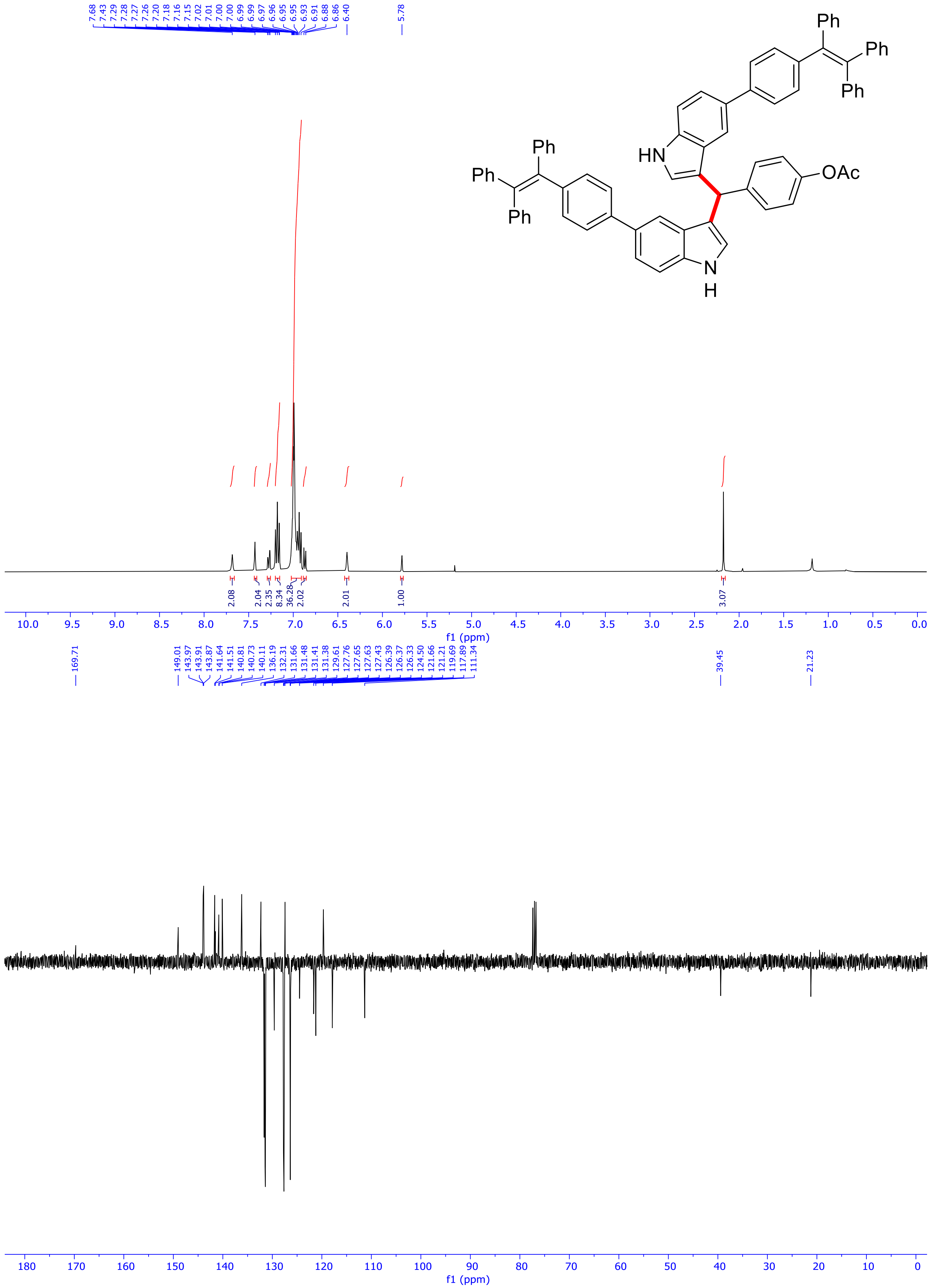

Figure S37. ^{1}H and ^{13}C (APT) NMR of **37e**

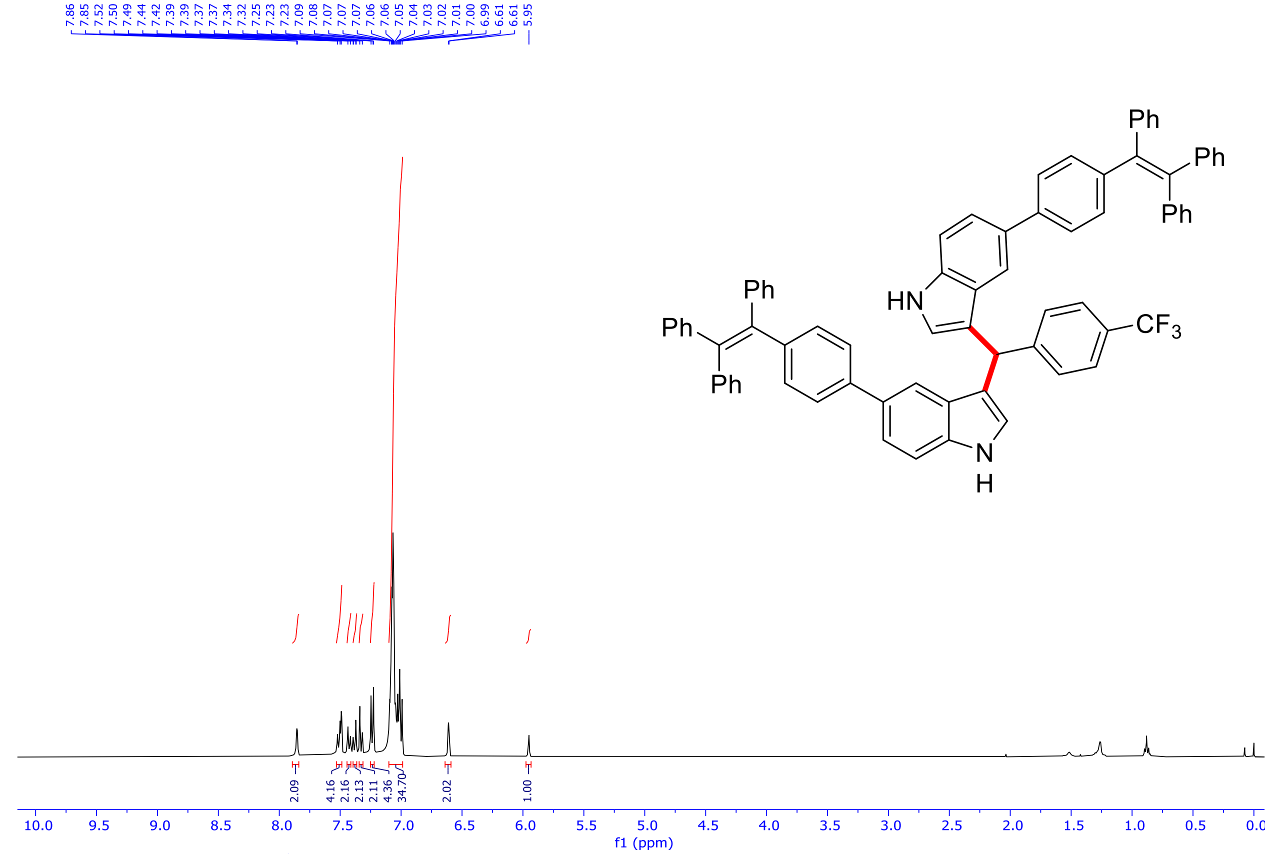

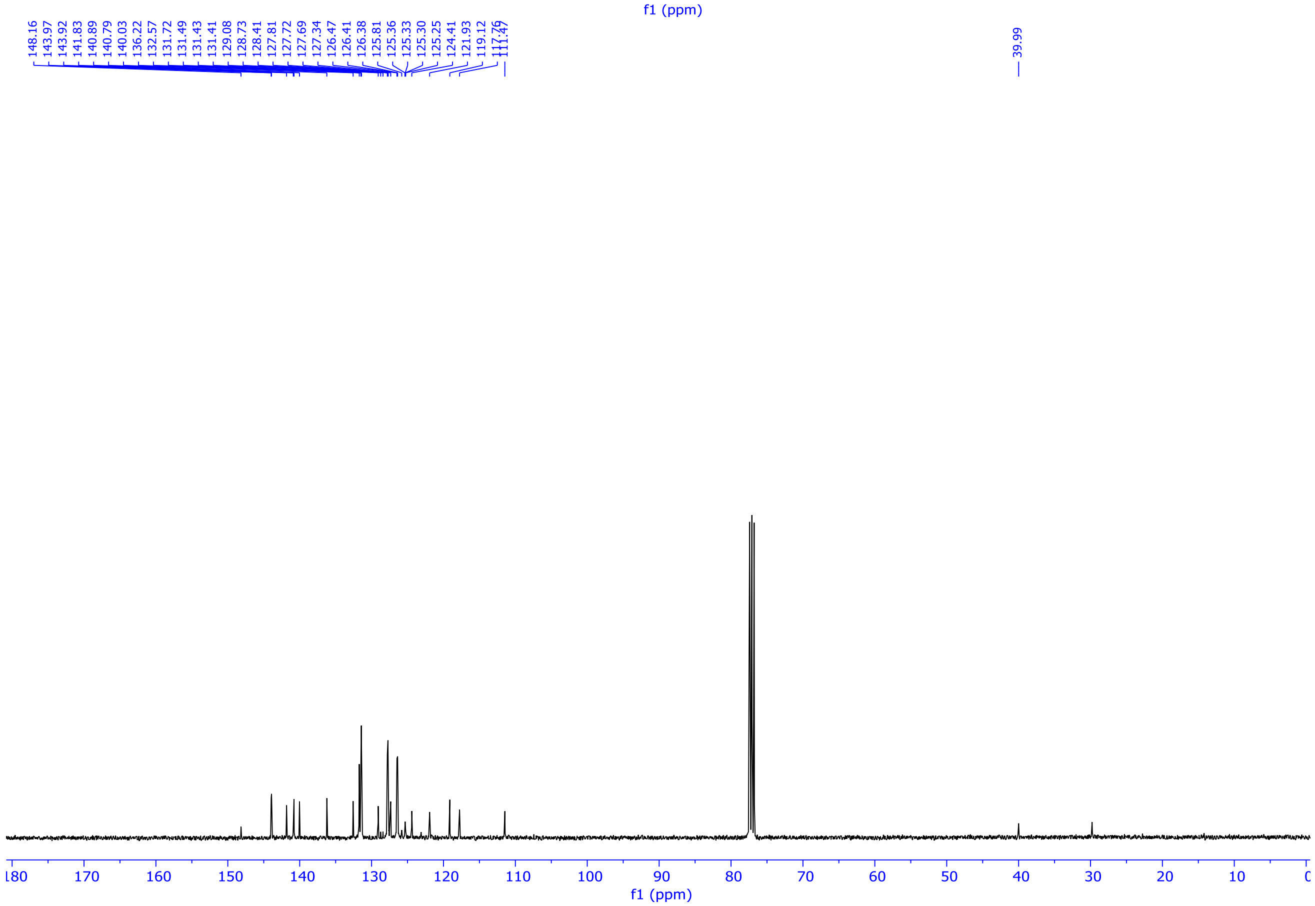

Figure S38. ^{1}H and ^{13}C NMR of **37f**

3. HRMS Spectra

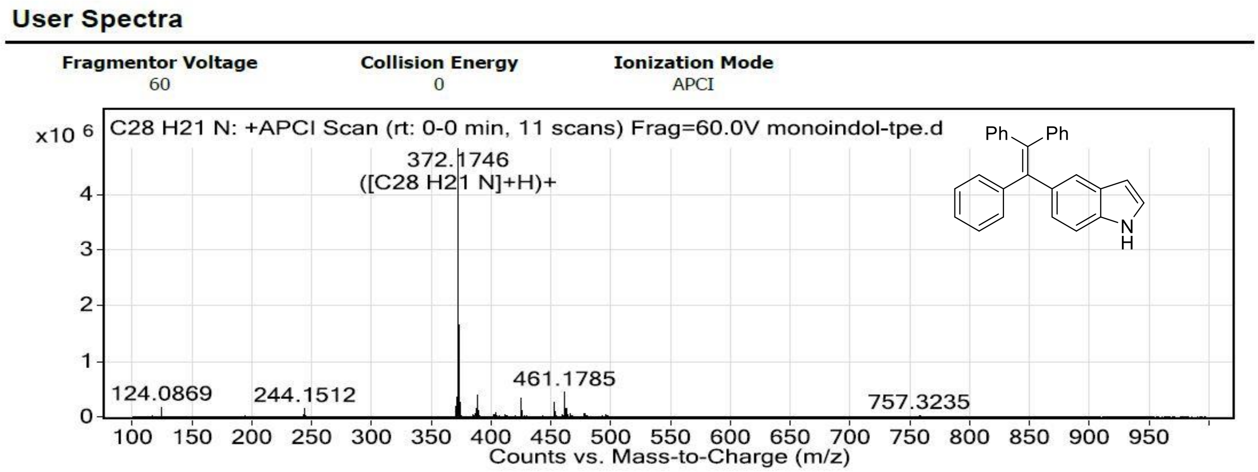

3.1. HRMS spectra of 9

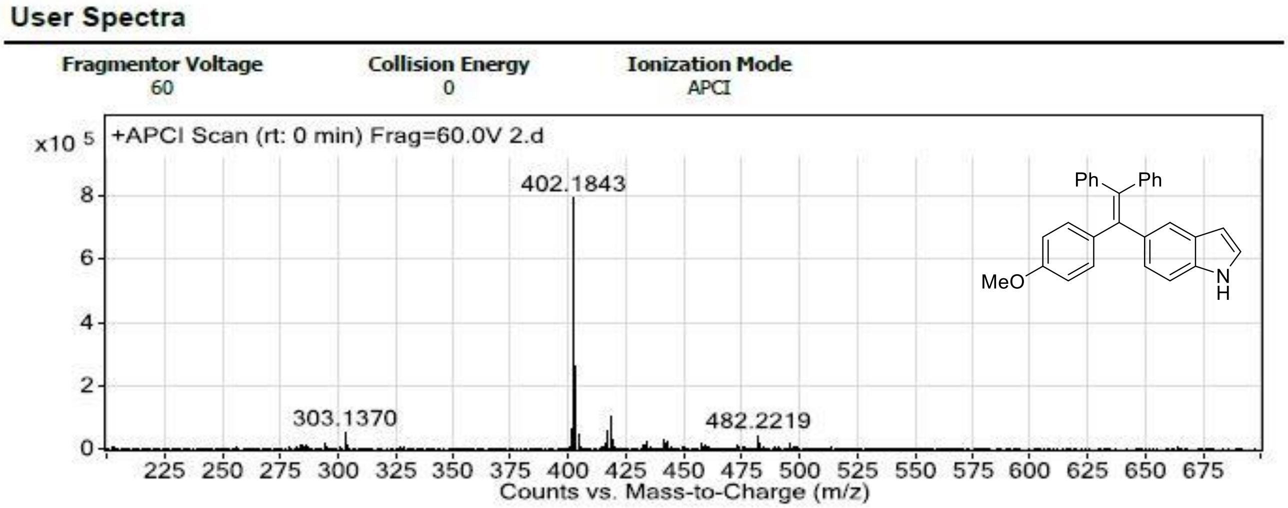

3.2. HRMS spectra of 10

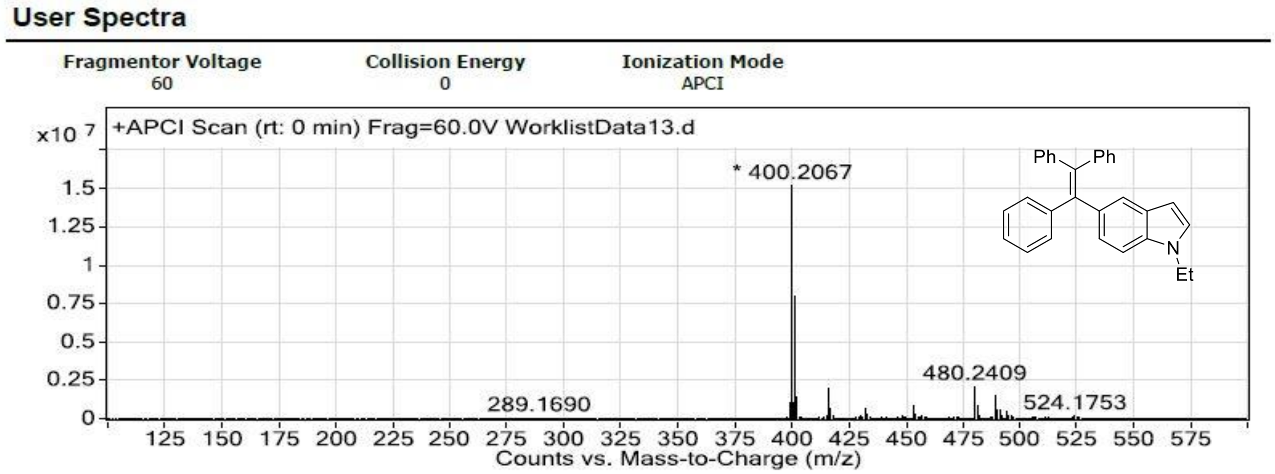

3.3. HRMS spectra of 14

User Spectra

Fragmentor Voltage	Collision Energy	Ionization Mode
60	0	APCI

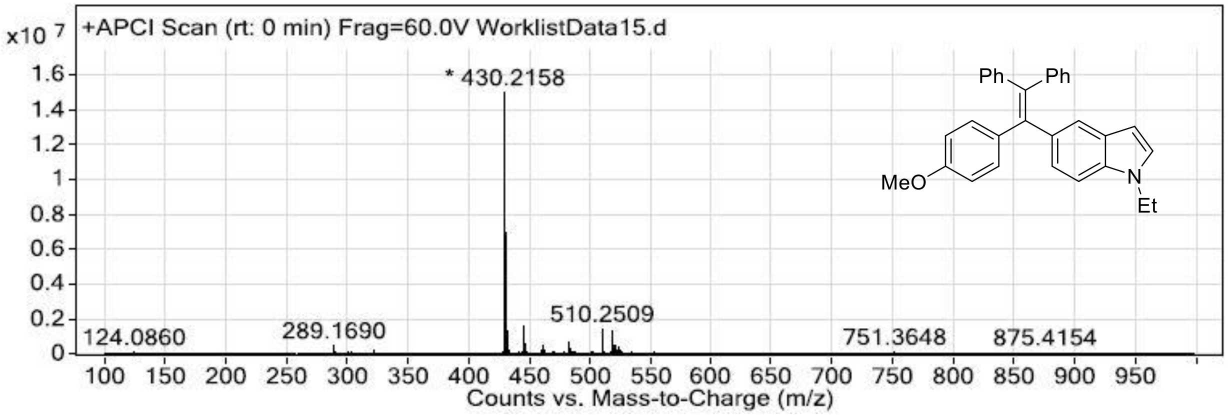

3.4. HRMS spectra of 15

User Spectra

Fragmentor Voltage	Collision Energy	Ionization Mode
90	0	ESI

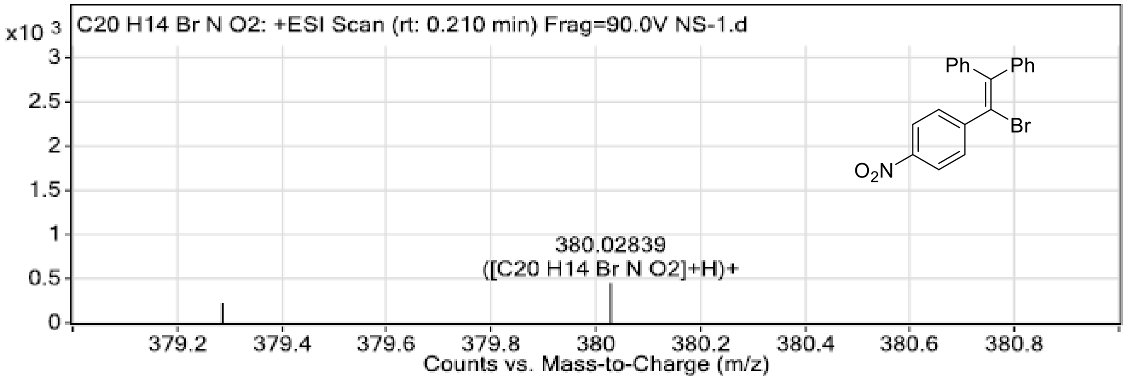

3.5. HRMS spectra of 19

User Spectra

Fragmentor Voltage	Collision Energy	Ionization Mode
60	0	APCI

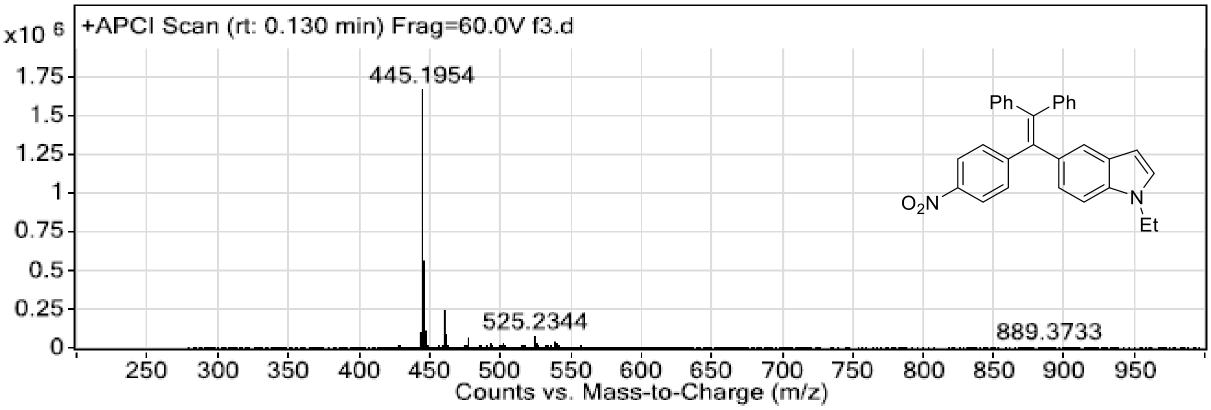

3.6. HRMS spectra of 21

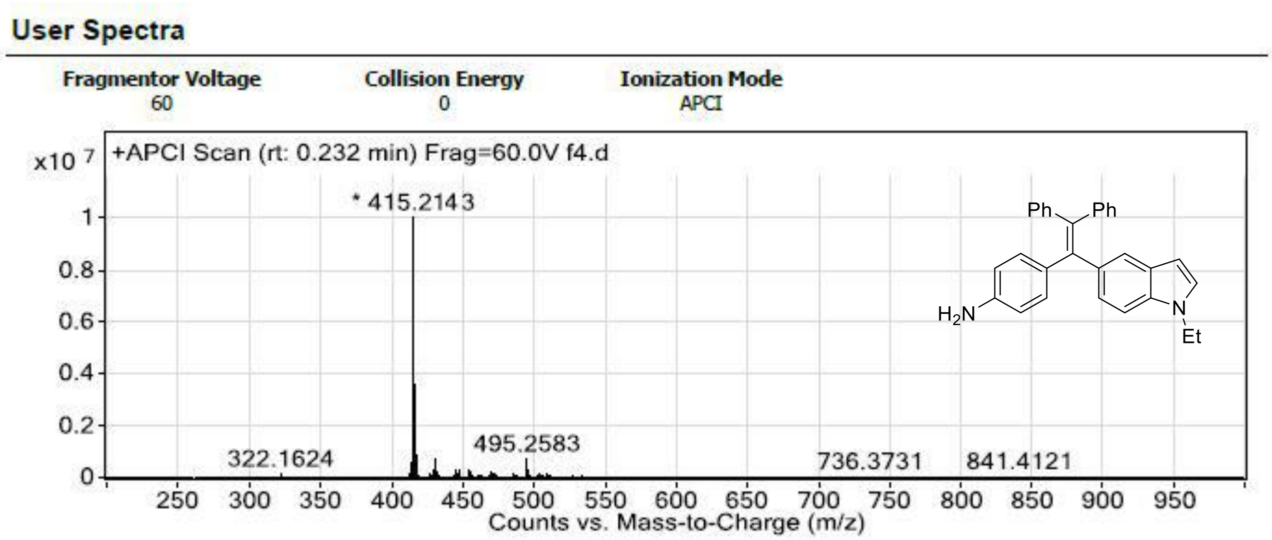

3.7. HRMS spectra of 22

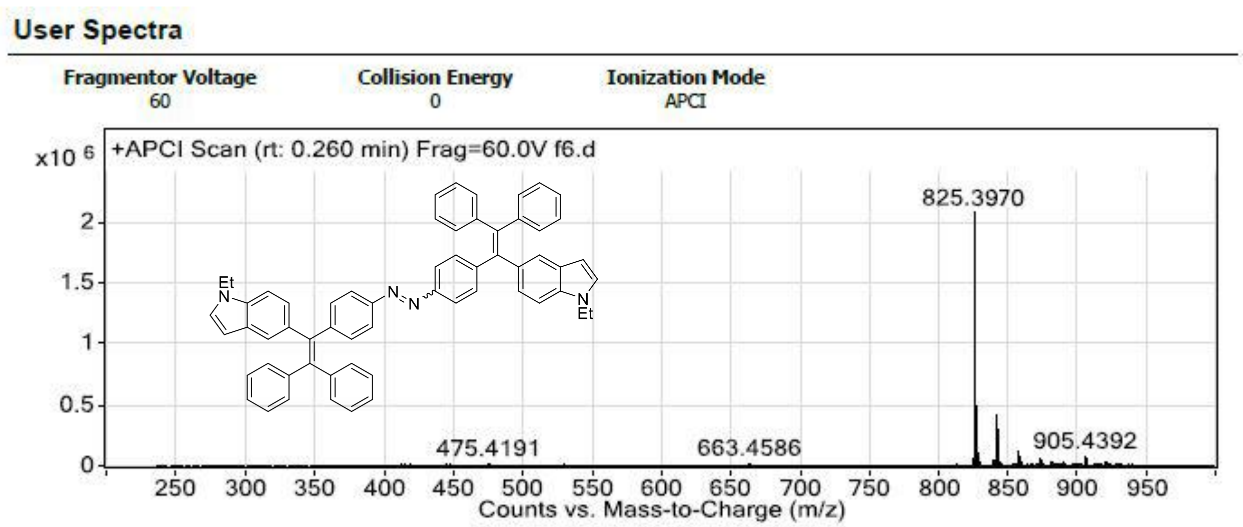

3.8. HRMS spectra of 23

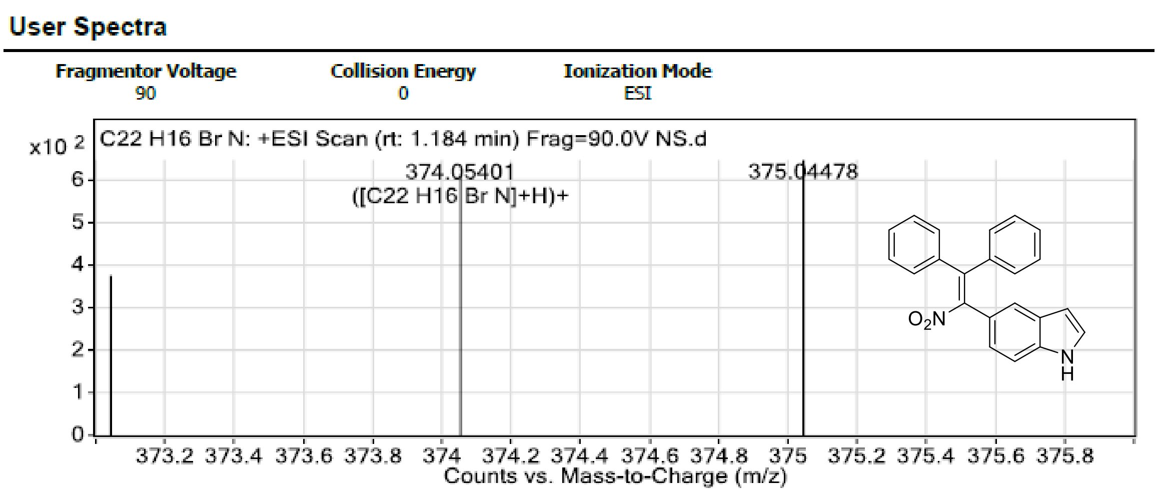

3.9. HRMS spectra of 26

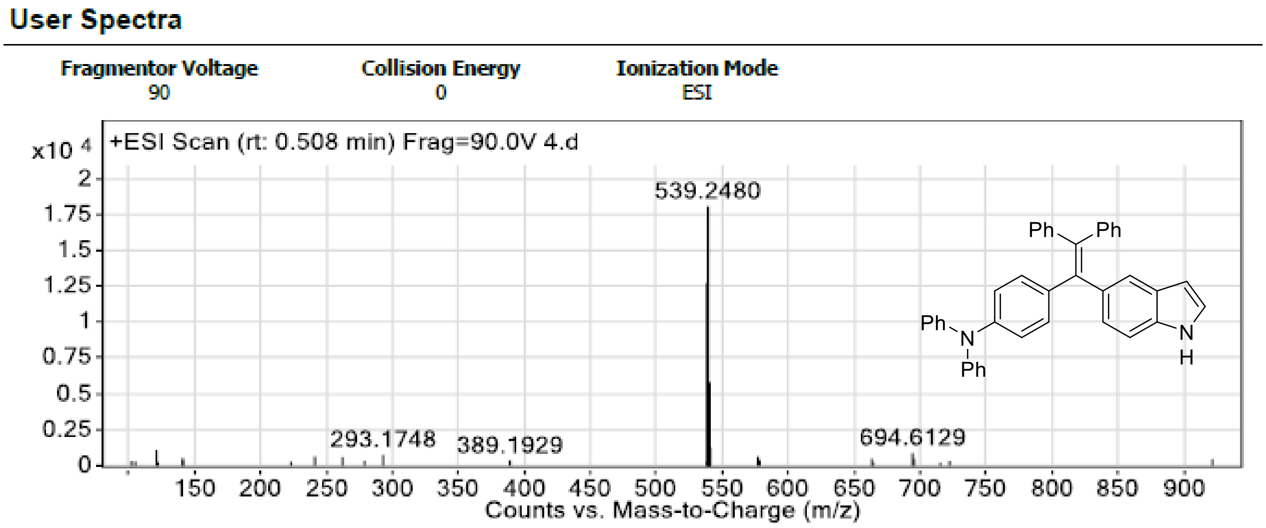

3.10. HRMS spectra of 28

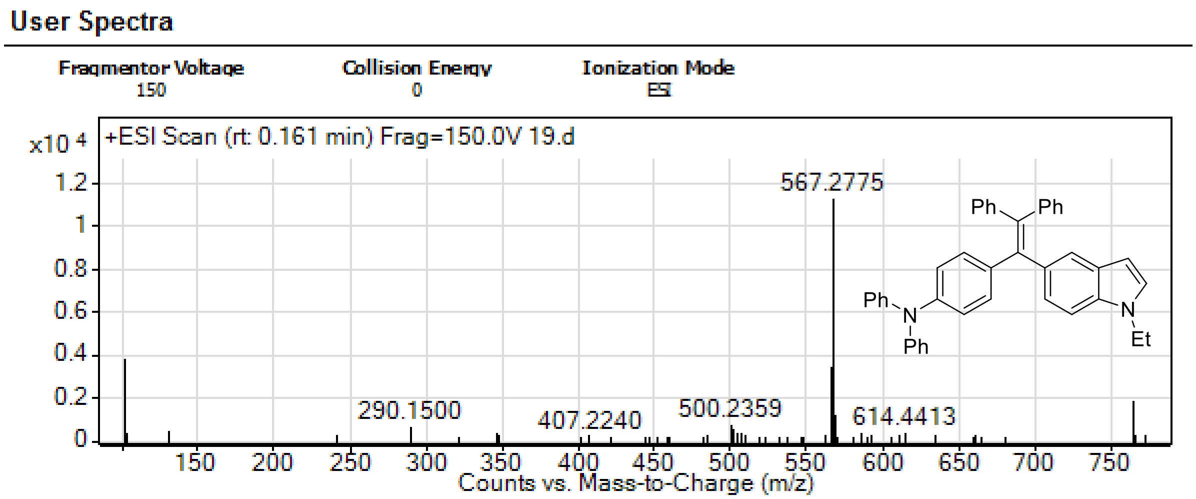

3.11. HRMS spectra of 29

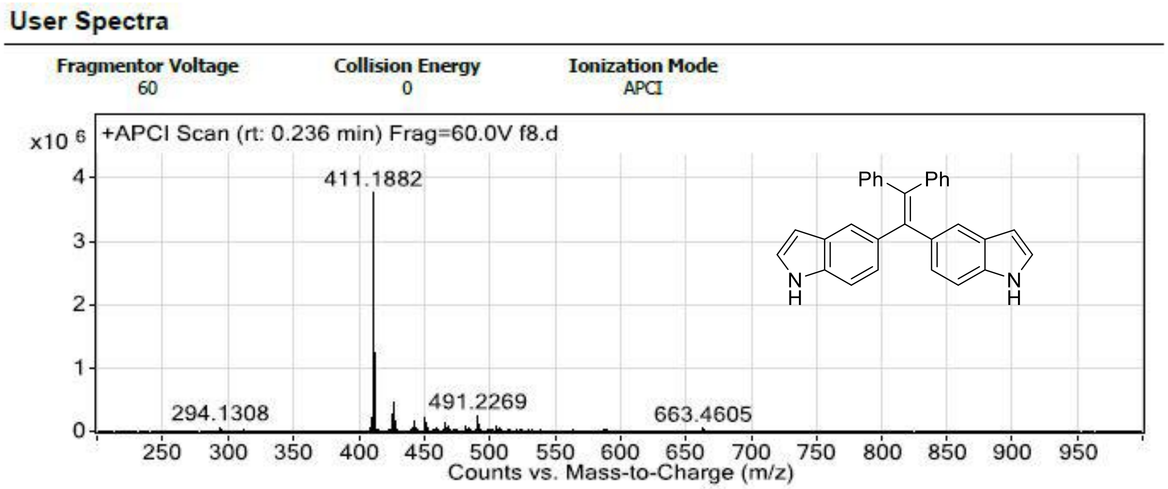

3.12. HRMS spectra of 30

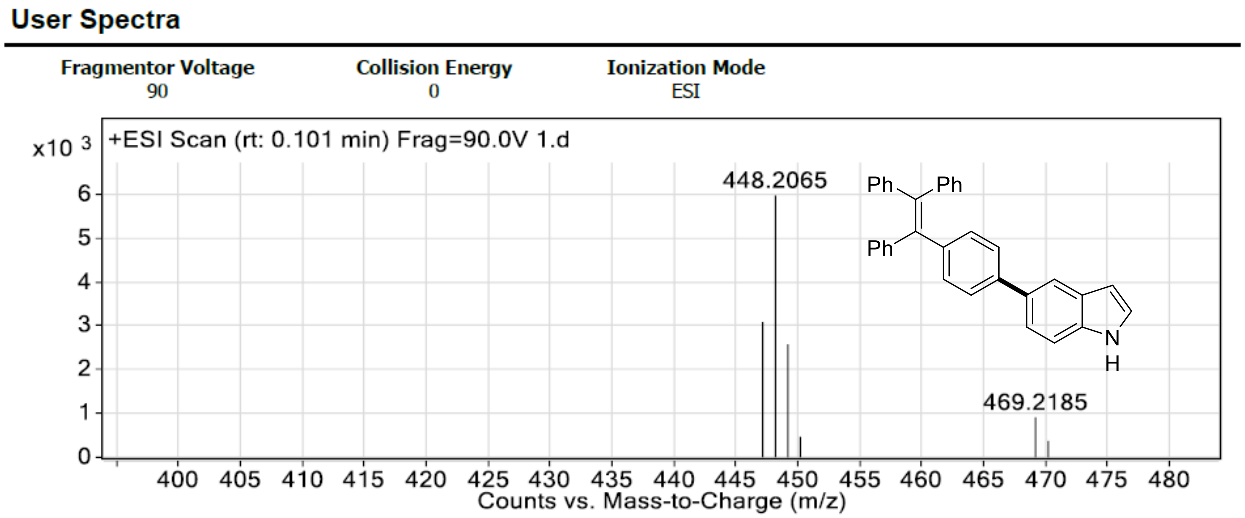

3.13. HRMS spectra of 32

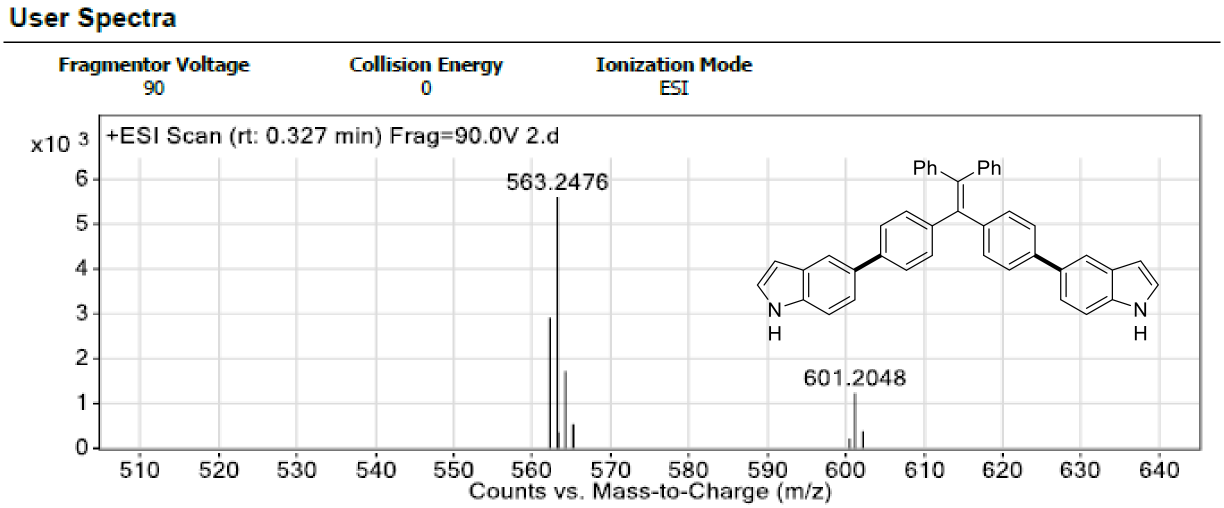

3.14. HRMS spectra of 33

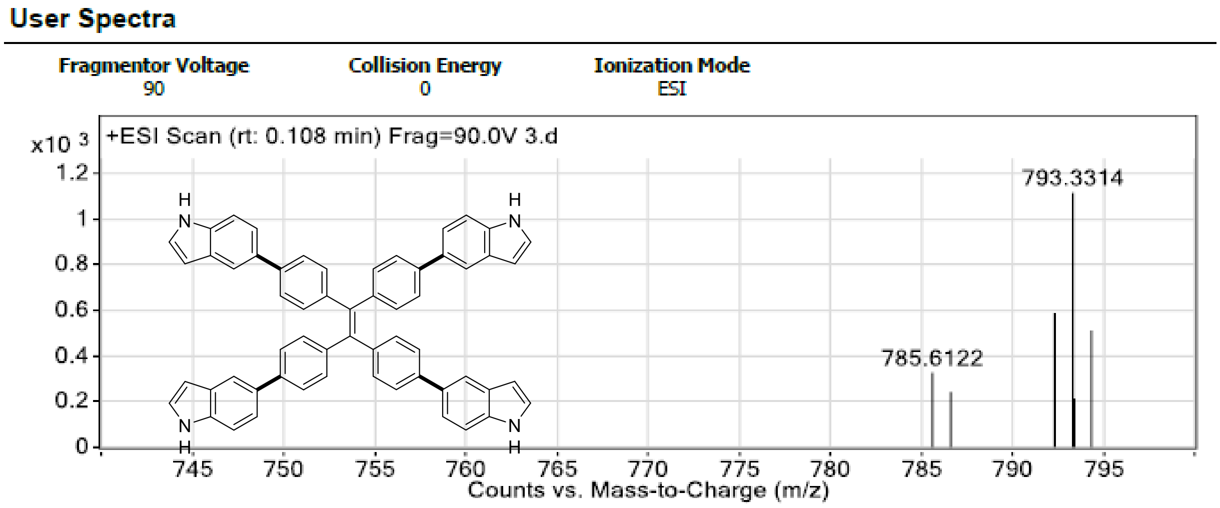

3.15. HRMS spectra of 34

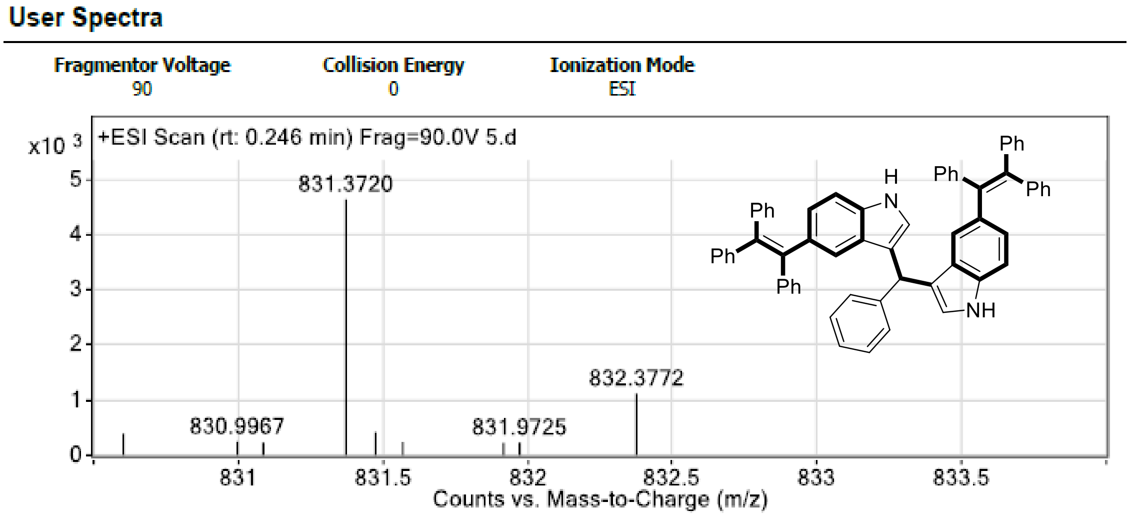

3.16. HRMS spectra of 36a

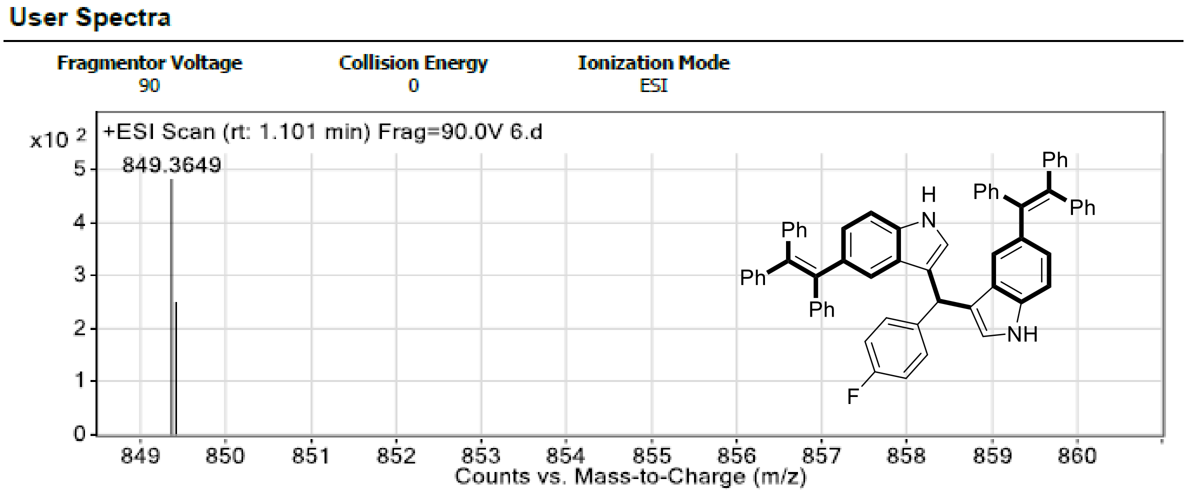

3.17. HRMS spectra of 36b

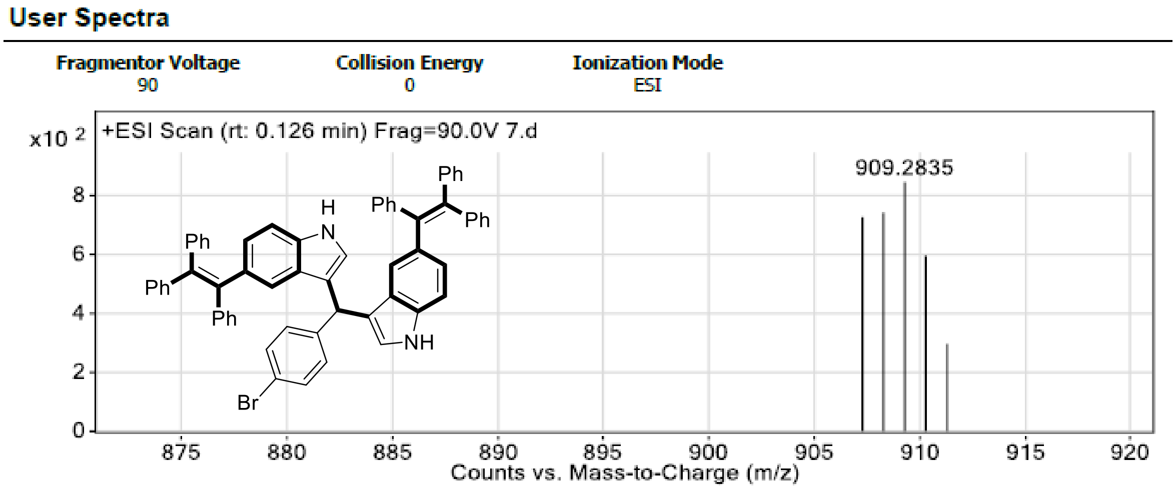

3.18. HRMS spectra of 36c

User Spectra

Fragmentor Voltage	Collision Energy	Ionization Mode
150	0	ESI

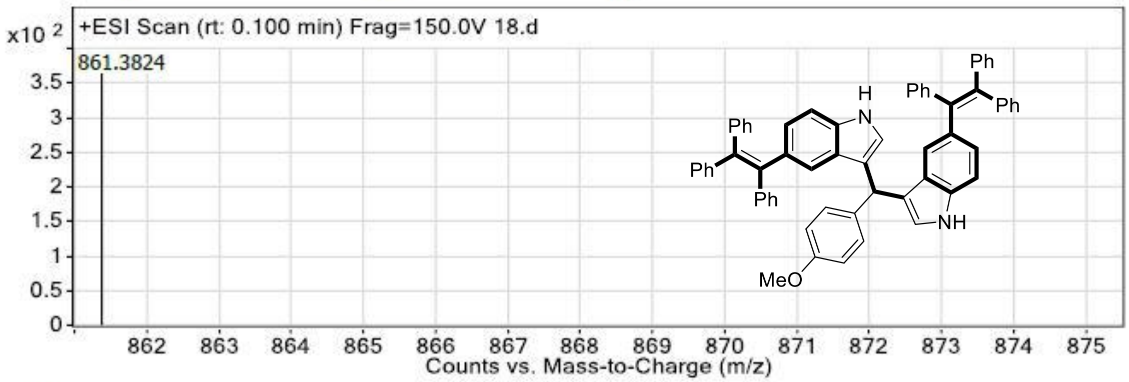

3.19. HRMS spectra of 36d

User Spectra

Fragmentor Voltage	Collision Energy	Ionization Mode
90	0	ESI

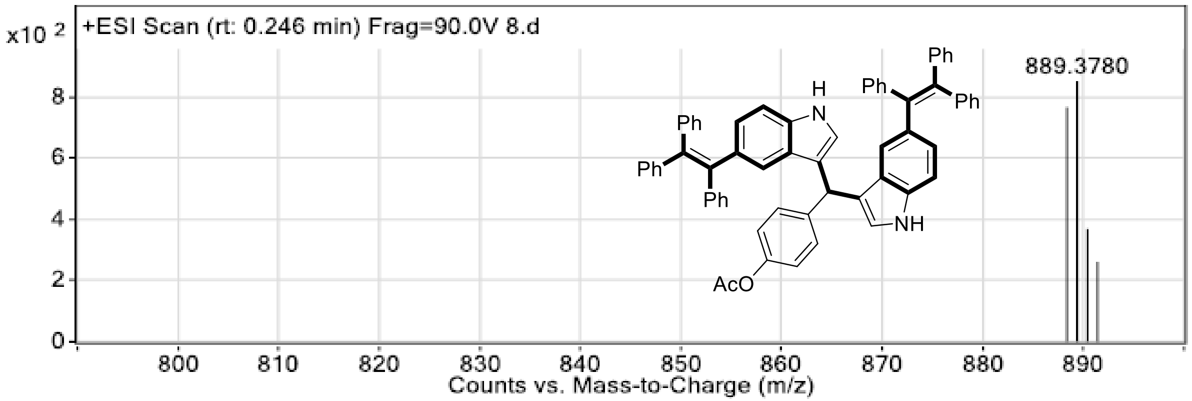

3.20. HRMS spectra of 36e

User Spectra

Fragmentor Voltage	Collision Energy	Ionization Mode
90	0	ESI

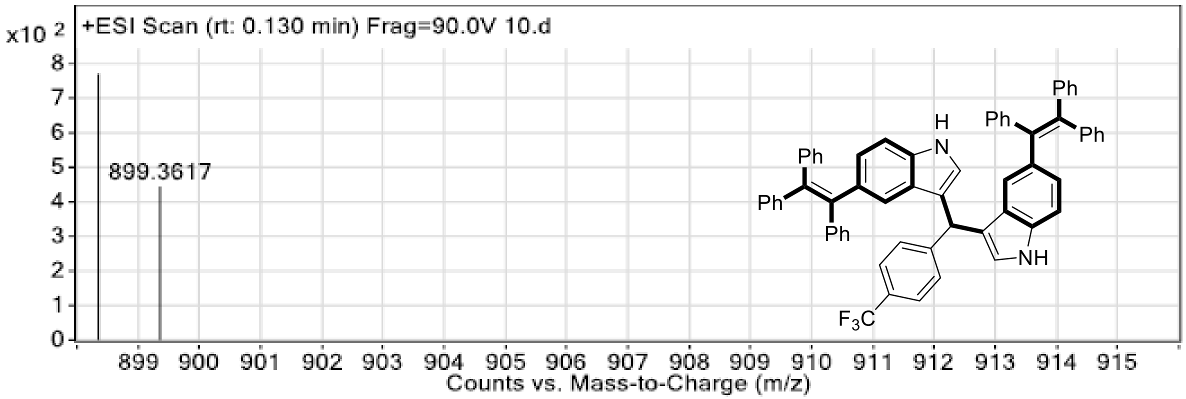

3.21. HRMS spectra of 36f

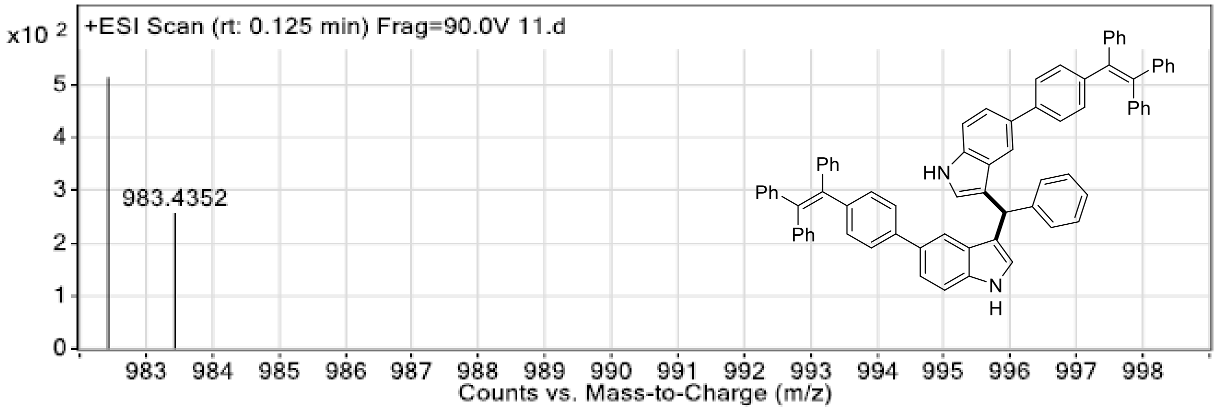

3.22. HRMS spectra of 37a

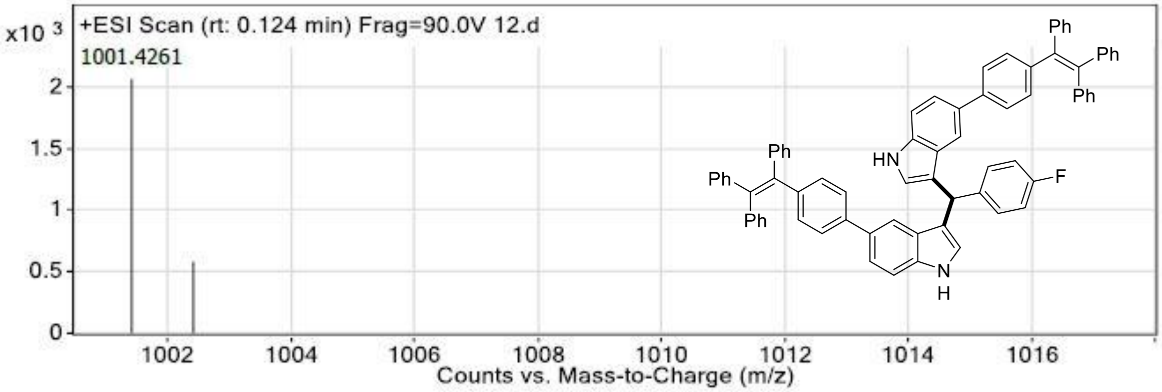

3.23. HRMS spectra of 37b

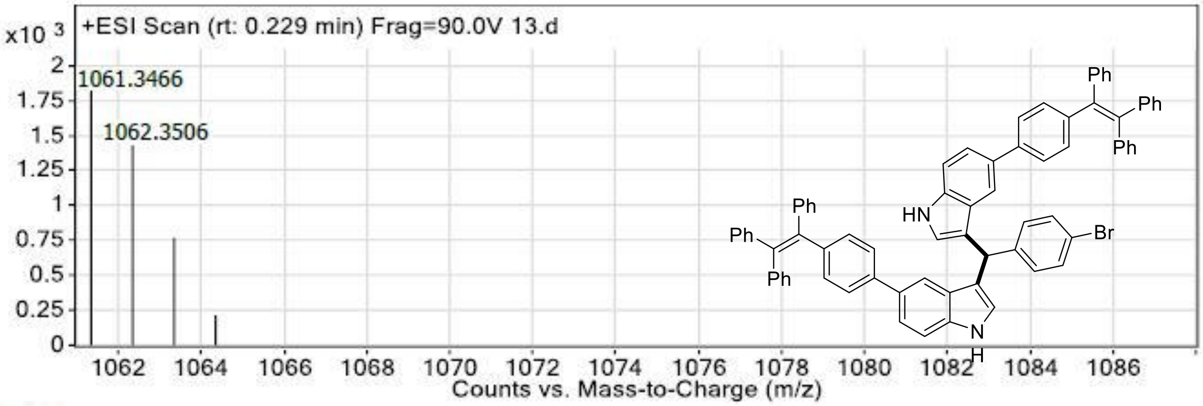

3.24. HRMS spectra of 37c

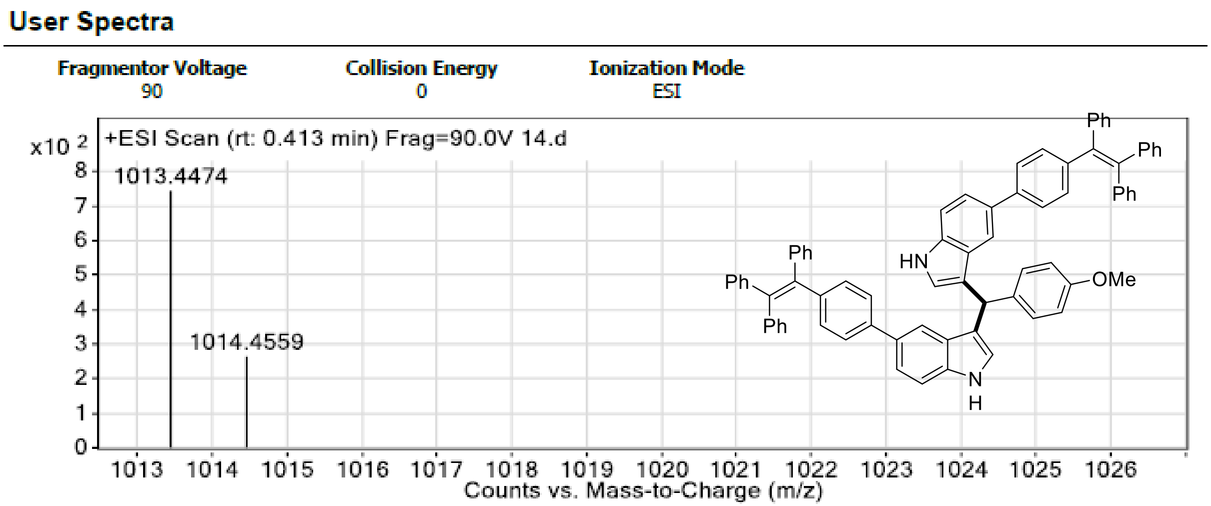

3.25. HRMS spectra of 37d

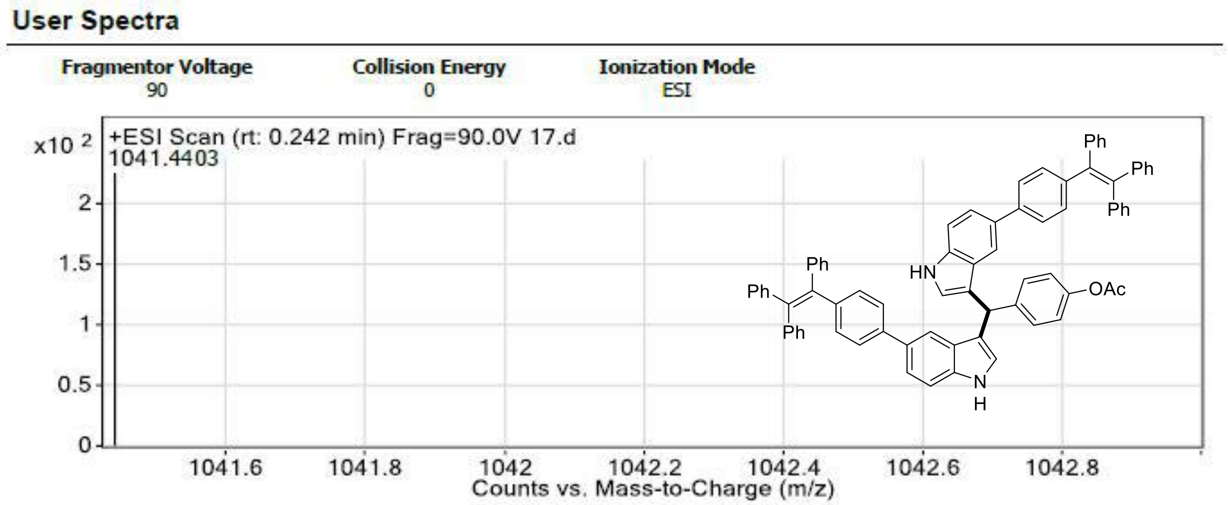

3.26. HRMS spectra of 37e

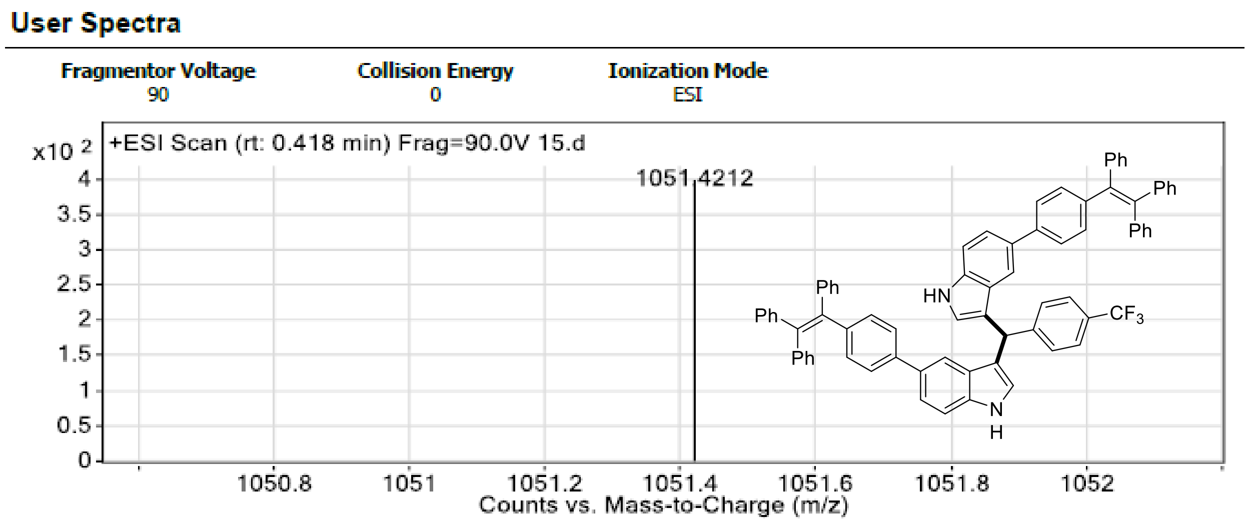

3.27. HRMS spectra of 37f

4. AIE Characteristics

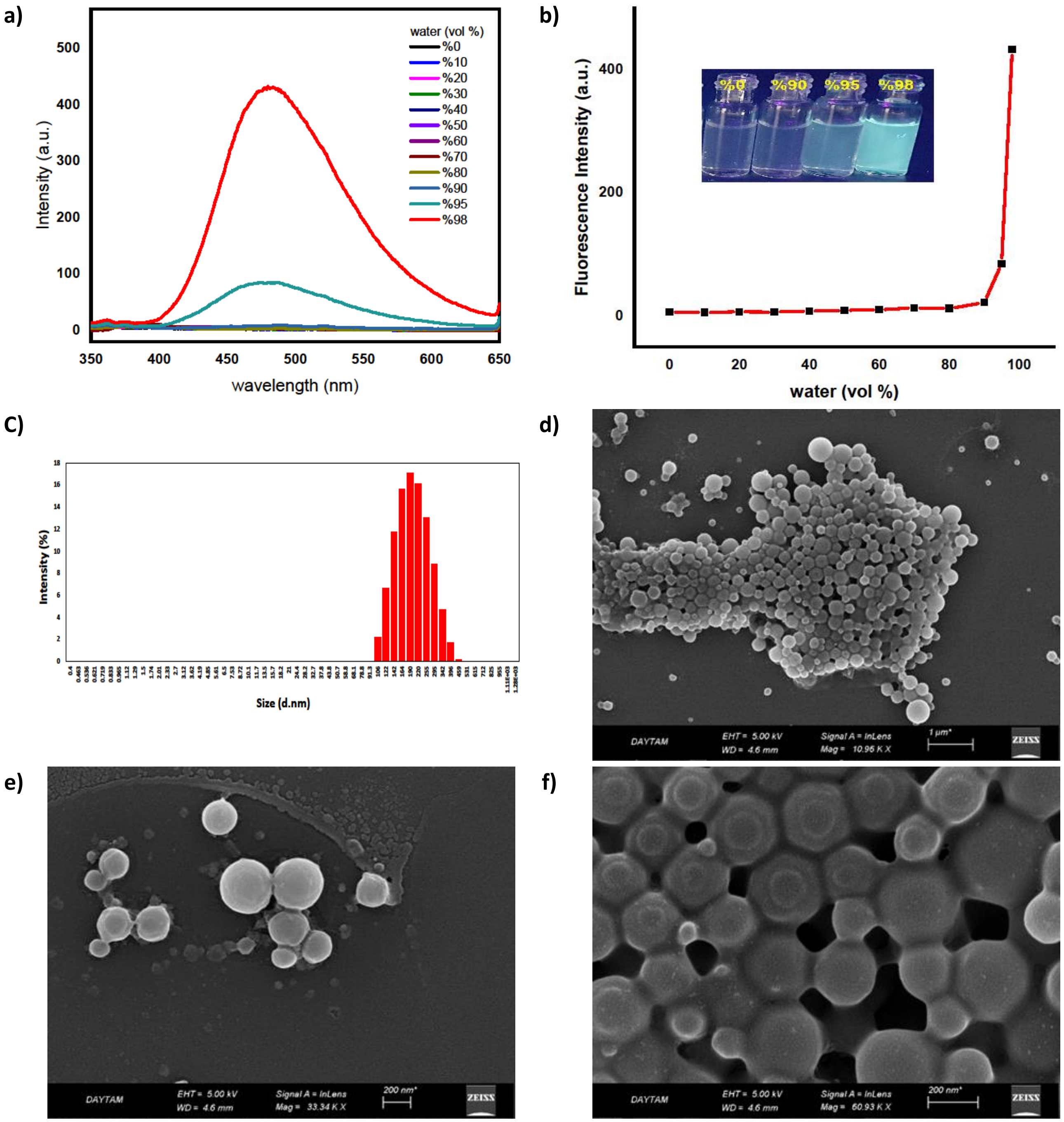

Figure S39. (a) Fluorescence spectra of **10** in THF and THF–water mixtures with different water fractions; (b) Plot of peak fluorescence intensities of **10** in THF–water with different water fractions (f_w). Luminogen concentration: 20 μM. Photograph of **10** in THF–water mixtures (f_w = 0–98%) with different water fractions (20 μM) under 365 nm UV illumination. Photograph of **10** in water–THF mixed solution (f_w = 0–98%) under a UV lamp. (c) DLS particle size-distribution profile of **10** in THF–water mixture (2: 98, v: v); (d-f) SEM image.

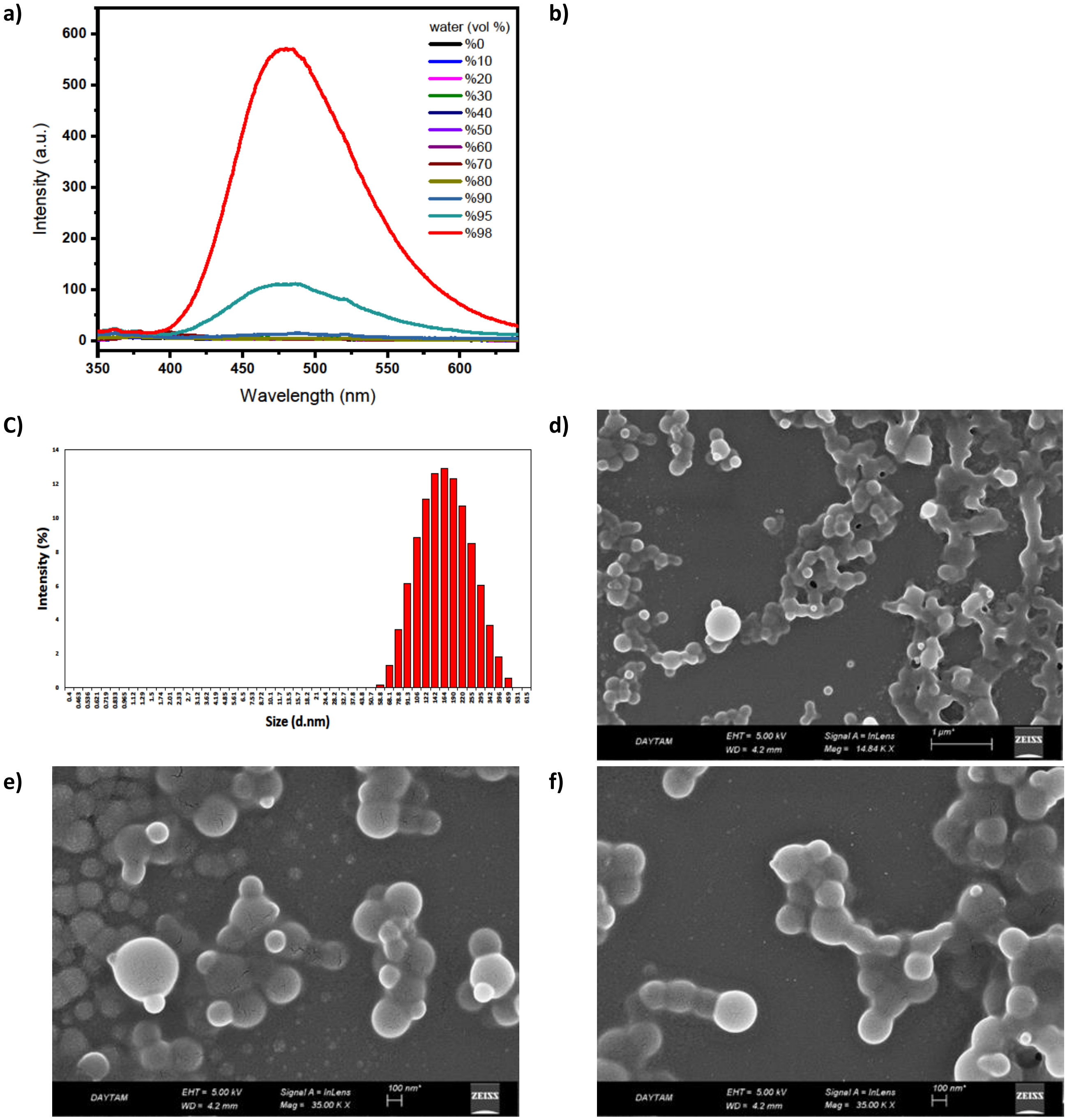

Figure S40. (a) Fluorescence spectra of **14** in THF and THF–water mixtures with different water fractions; (b) Plot of peak fluorescence intensities of **14** in THF–water with different water fractions (f_w). Luminogen concentration: 20 μM. Photograph of **14** in THF–water mixtures (f_w = 0–98%) with different water fractions (20 μM) under 365 nm UV illumination. Photograph of **14** in water–THF mixed solution (f_w = 0–98%) under a UV lamp. (c) DLS particle size-distribution profile of **14** in THF–water mixture (2: 98, v: v); (d-f) SEM image.

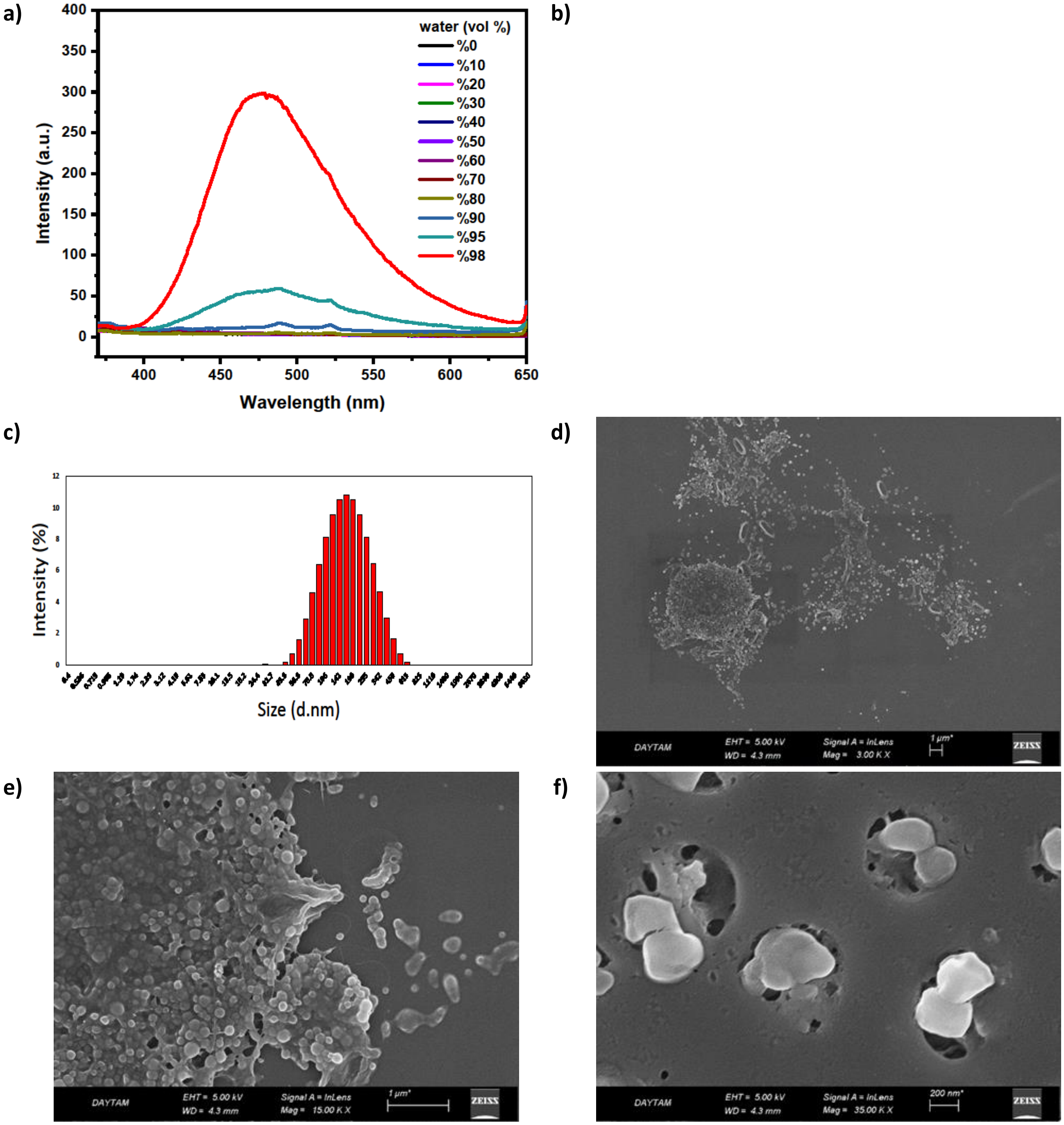

Figure S41. (a) Fluorescence spectra of **15** in THF and THF–water mixtures with different water fractions; (b) Plot of peak fluorescence intensities of **15** in THF–water with different water fractions (f_w). Luminogen concentration: 20 μM. Photograph of **15** in THF–water mixtures (f_w = 0–98%) with different water fractions (20 μM) under 365 nm UV illumination. Photograph of **15** in water–THF mixed solution (f_w = 0–98%) under a UV lamp. (c) DLS particle size-distribution profile of **15** in THF–water mixture (2: 98, v: v); (d-f) SEM image.

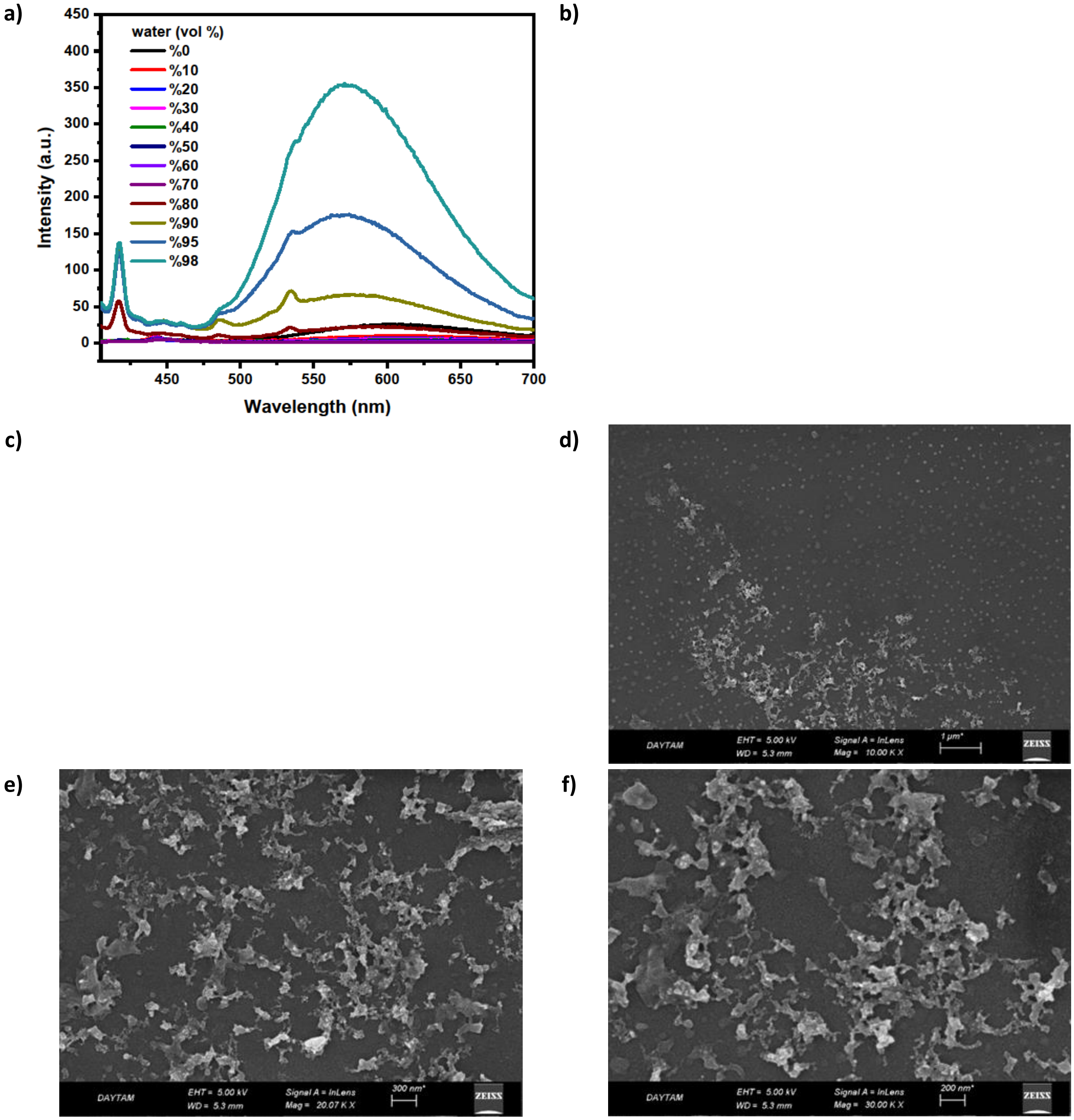

Figure S42. (a) Fluorescence spectra of **21** in THF and THF–water mixtures with different water fractions; (b) Plot of peak fluorescence intensities of **21** in THF–water with different water fractions (f_w). Luminogen concentration: 20 µM. Photograph of **21** in THF–water mixtures (f_w = 0–98%) with different water fractions (20 µM) under 365 nm UV illumination. Photograph of **21** in water–THF mixed solution (f_w = 0–98%) under a UV lamp. (c) DLS particle size-distribution profile of **21** in THF–water mixture (2: 98, v: v); (d-f) SEM image.

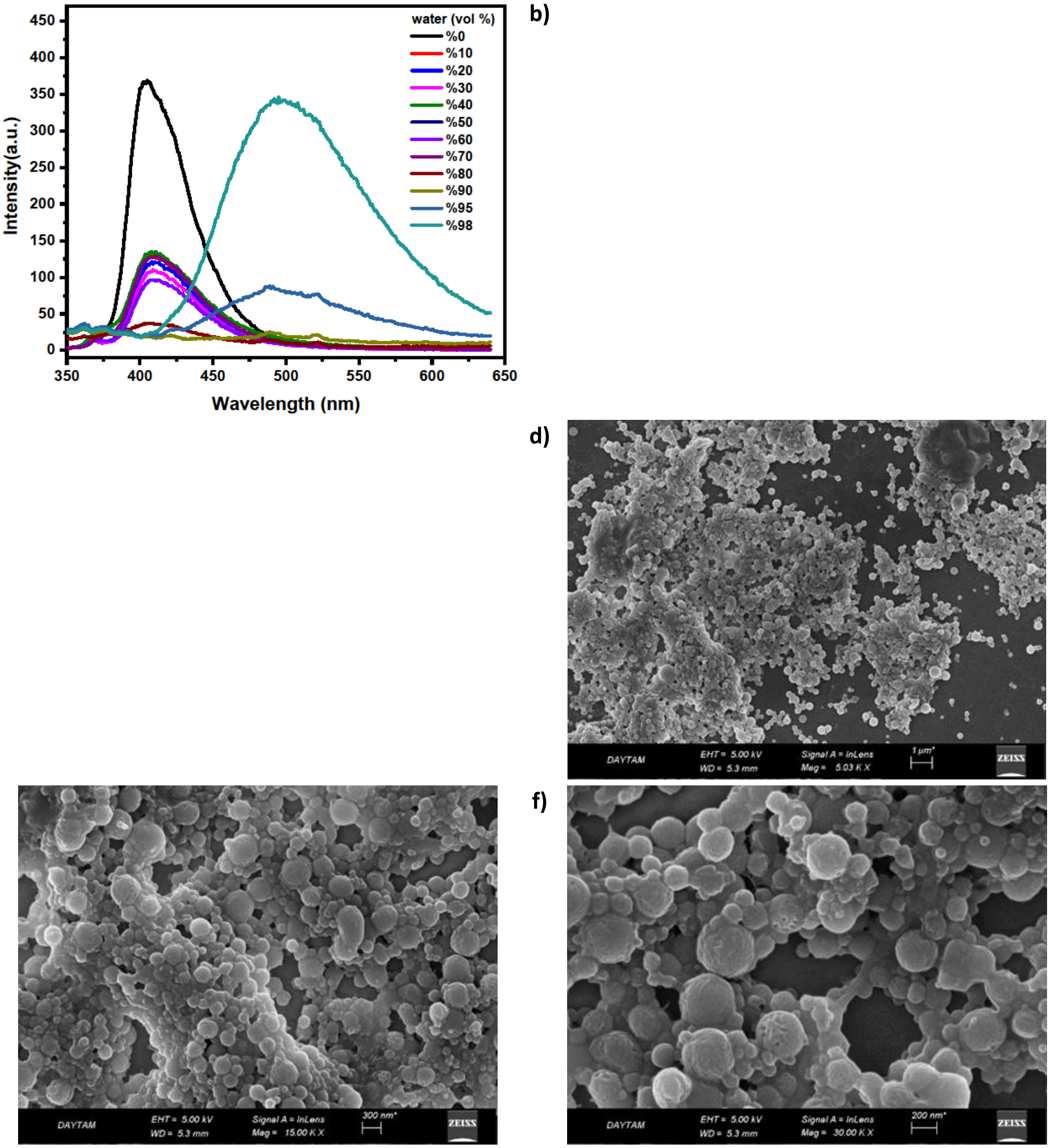

Figure S43. (a) Fluorescence spectra of **22** in THF and THF–water mixtures with different water fractions; (b) Plot of peak fluorescence intensities of **22** in THF–water with different water fractions (f_w). Luminogen concentration: 20 μM. Photograph of **22** in THF–water mixtures (f_w = 0–98%) with different water fractions (20 μM) under 365 nm UV illumination. Photograph of **22** in water–THF mixed solution (f_w = 0–98%) under a UV lamp. (c) DLS particle size-distribution profile of **22** in THF–water mixture (2: 98, v: v); (d-f) SEM image.

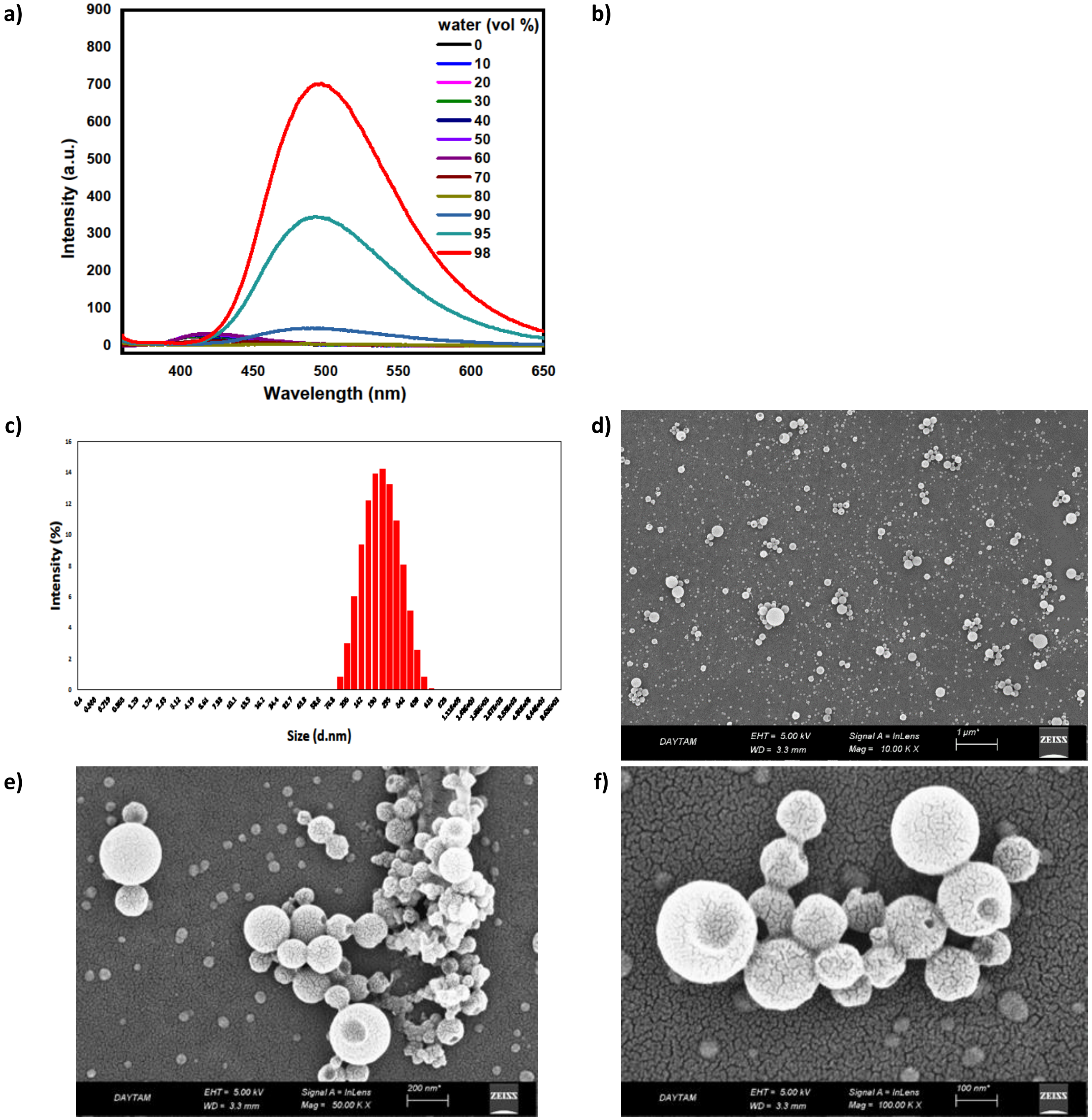

Figure S44. (a) Fluorescence spectra of **28** in THF and THF–water mixtures with different water fractions; (b) Plot of peak fluorescence intensities of **28** in THF–water with different water fractions (f_w). Luminogen concentration: 20 μM. Photograph of **28** in THF–water mixtures (f_w = 0–98%) with different water fractions (20 μM) under 365 nm UV illumination. Photograph of **28** in water–THF mixed solution (f_w = 0–98%) under a UV lamp. (c) DLS particle size-distribution profile of **28** in THF–water mixture (2: 98, v: v); (d-f) SEM image.

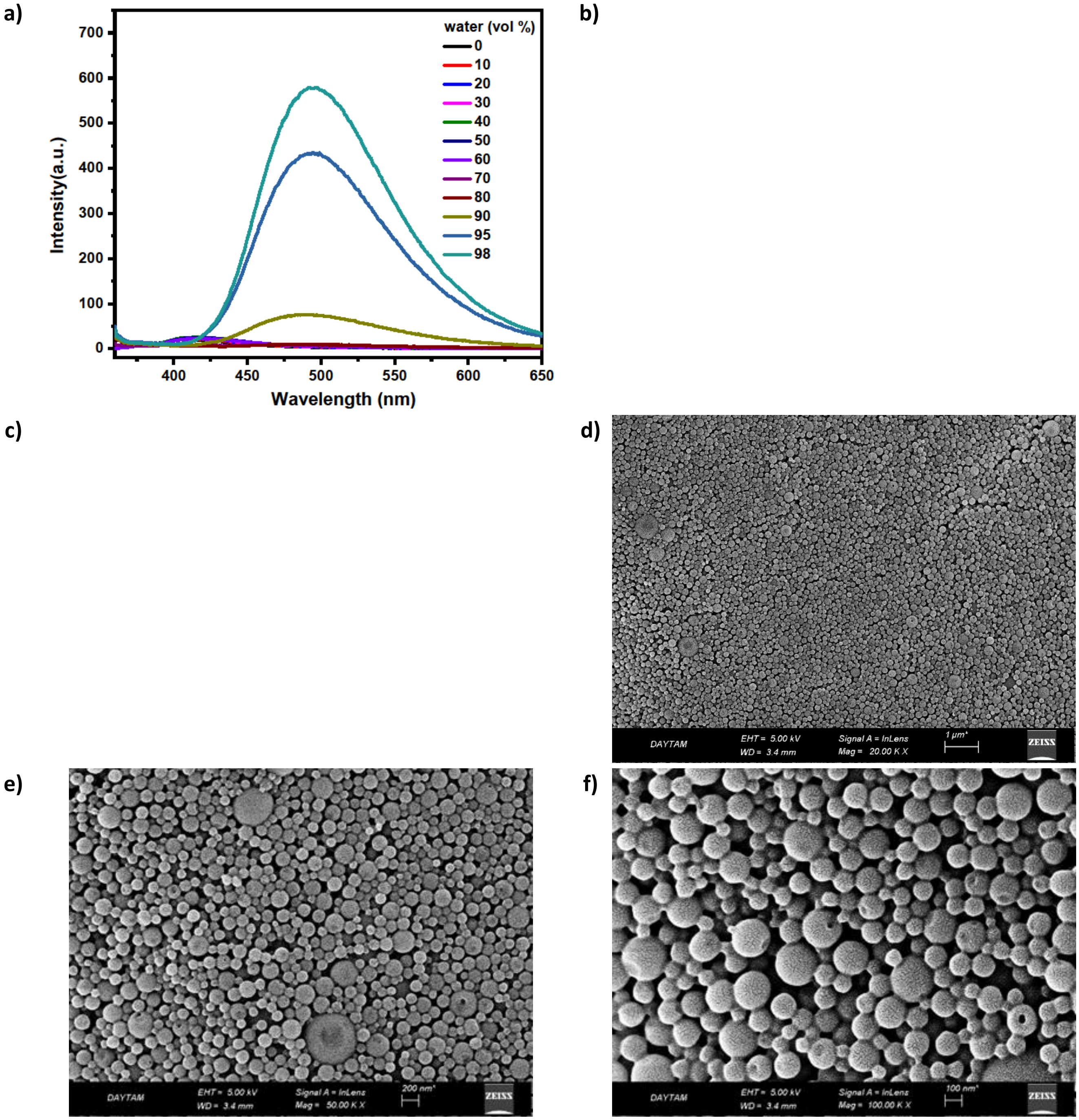

Figure S45. (a) Fluorescence spectra of **29** in THF and THF–water mixtures with different water fractions; (b) Plot of peak fluorescence intensities of **29** in THF–water with different water fractions (f_w). Luminogen concentration: 20 µM. Photograph of **29** in THF–water mixtures (f_w = 0–98%) with different water fractions (20 µM) under 365 nm UV illumination. Photograph of **29** in water–THF mixed solution (f_w = 0–98%) under a UV lamp. (c) DLS particle size-distribution profile of **29** in THF–water mixture (2: 98, v: v); (d-f) SEM image.

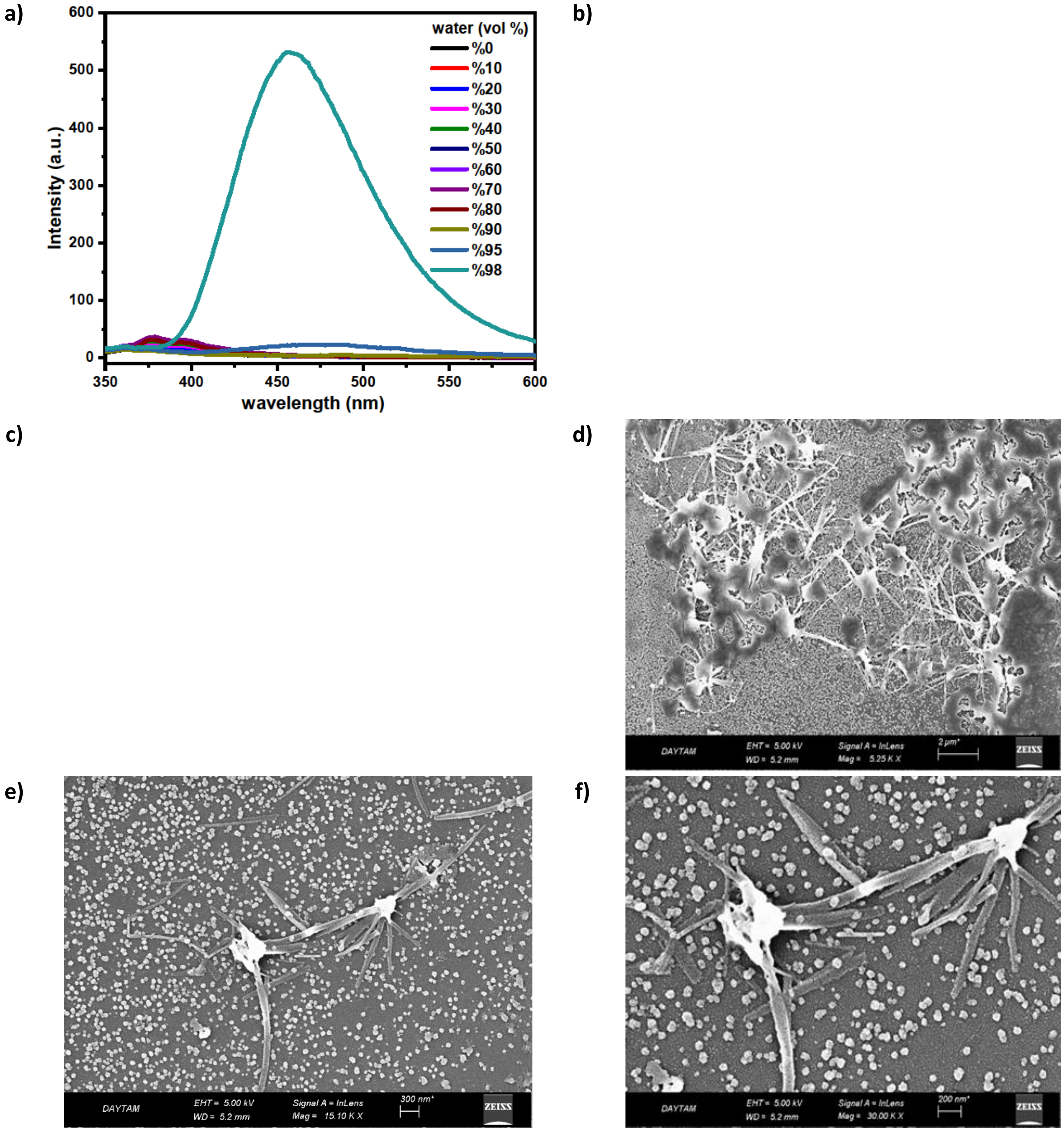

Figure S46. (a) Fluorescence spectra of **30** in THF and THF–water mixtures with different water fractions; (b) Plot of peak fluorescence intensities of **30** in THF–water with different water fractions (f_w). Luminogen concentration: 20 μM. Photograph of **30** in THF–water mixtures (f_w = 0–98%) with different water fractions (20 μM) under 365 nm UV illumination. Photograph of **30** in water–THF mixed solution (f_w = 0–98%) under a UV lamp. (c) DLS particle size-distribution profile of **30** in THF–water mixture (2: 98, v: v); (d-f) SEM image.

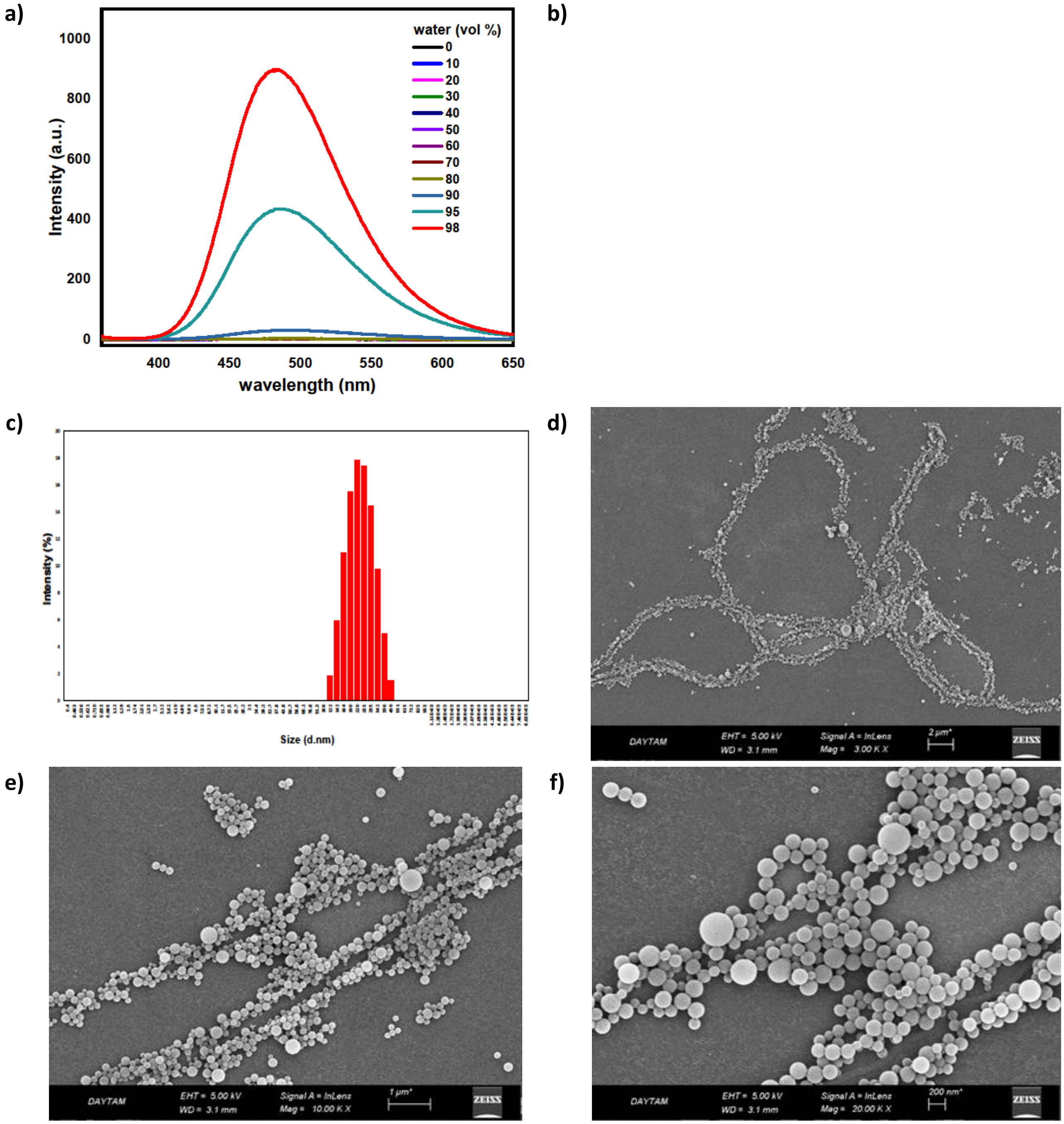

Figure S47. (a) Fluorescence spectra of **32** in THF and THF–water mixtures with different water fractions; (b) Plot of peak fluorescence intensities of **32** in THF–water with different water fractions (f_w). Luminogen concentration: 20 µM. Photograph of **32** in THF–water mixtures (f_w = 0–98%) with different water fractions (20 µM) under 365 nm UV illumination. Photograph of **32** in water–THF mixed solution (f_w = 0–98%) under a UV lamp. (c) DLS particle size-distribution profile of **32** in THF–water mixture (2: 98, v: v); (d-f) SEM image.

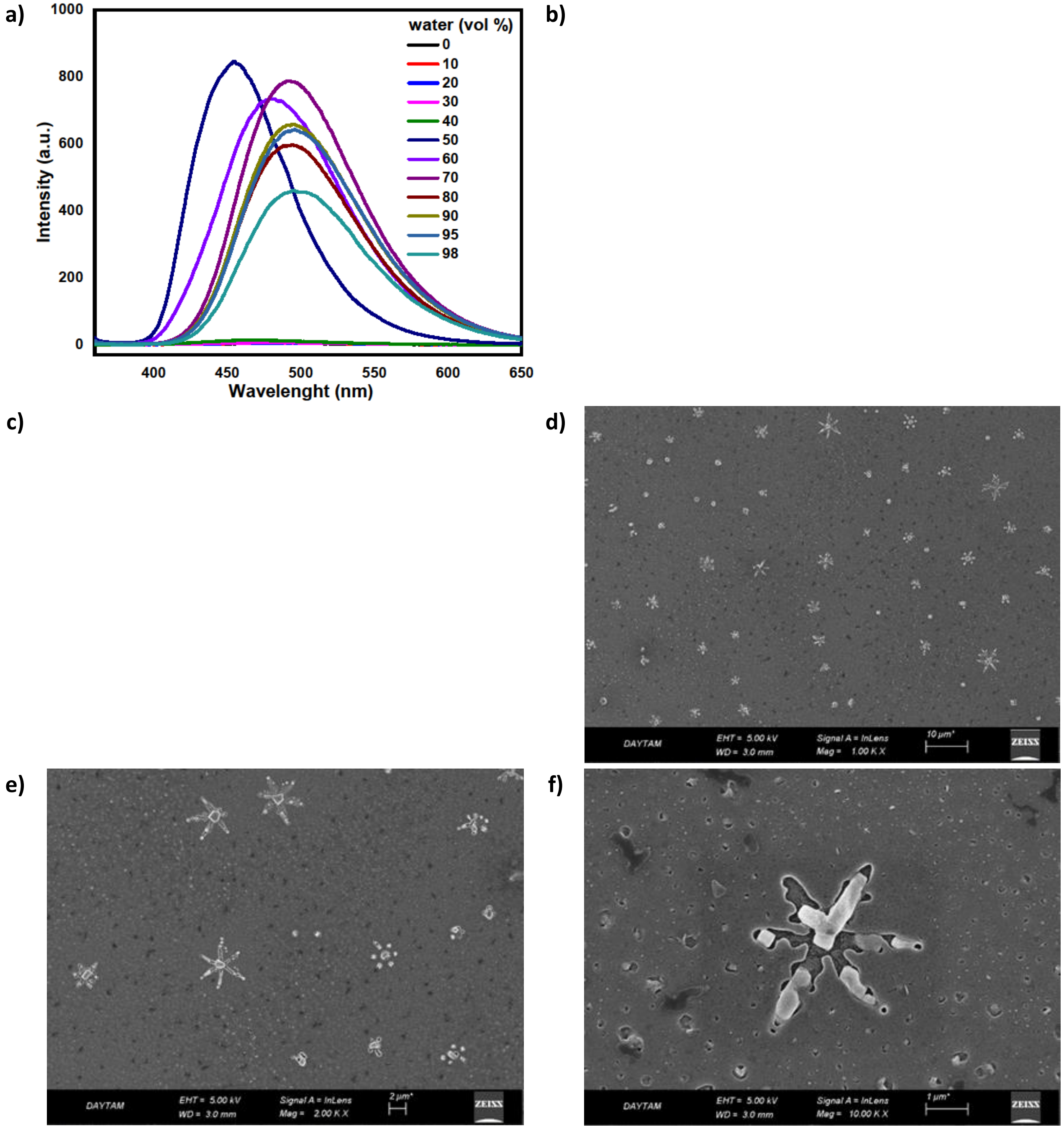

Figure S48. (a) Fluorescence spectra of **33** in DMSO and DMSO–water mixtures with different water fractions; (b) Plot of peak fluorescence intensities of **33** in DMSO–water with different water fractions (f_w). Luminogen concentration: 20 μM. Photograph of **33** in DMSO–water mixtures (f_w = 0–98%) with different water fractions (20 μM) under 365 nm UV illumination. Photograph of **33** in water–DMSO mixed solution (f_w = 0–98%) under a UV lamp. (c) DLS particle size-distribution profile of **33** in DMSO–water mixture (2: 98, v: v); (d-f) SEM image.

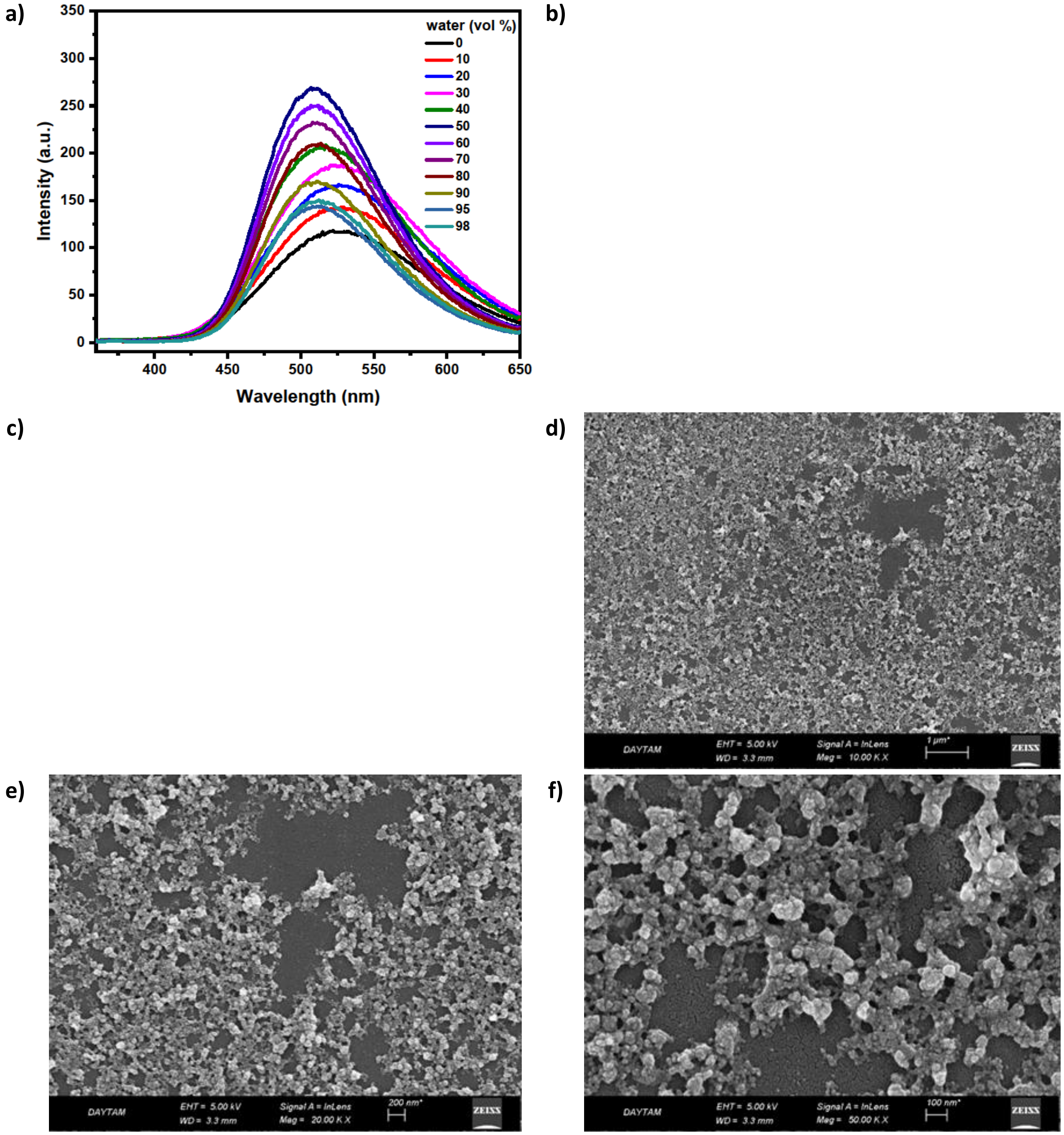

Figure S49. (a) Fluorescence spectra of **34** in DMSO and DMSO–water mixtures with different water fractions; (b) Plot of peak fluorescence intensities of **34** in DMSO–water with different water fractions (f_w). Luminogen concentration: 20 μM. Photograph of **34** in DMSO–water mixtures (f_w = 0–98%) with different water fractions (20 μM) under 365 nm UV illumination. Photograph of **34** in water–DMSO mixed solution (f_w = 0–98%) under a UV lamp. (c) DLS particle size-distribution profile of **34** in DMSO–water mixture (2: 98, v: v); (d-f) SEM image.

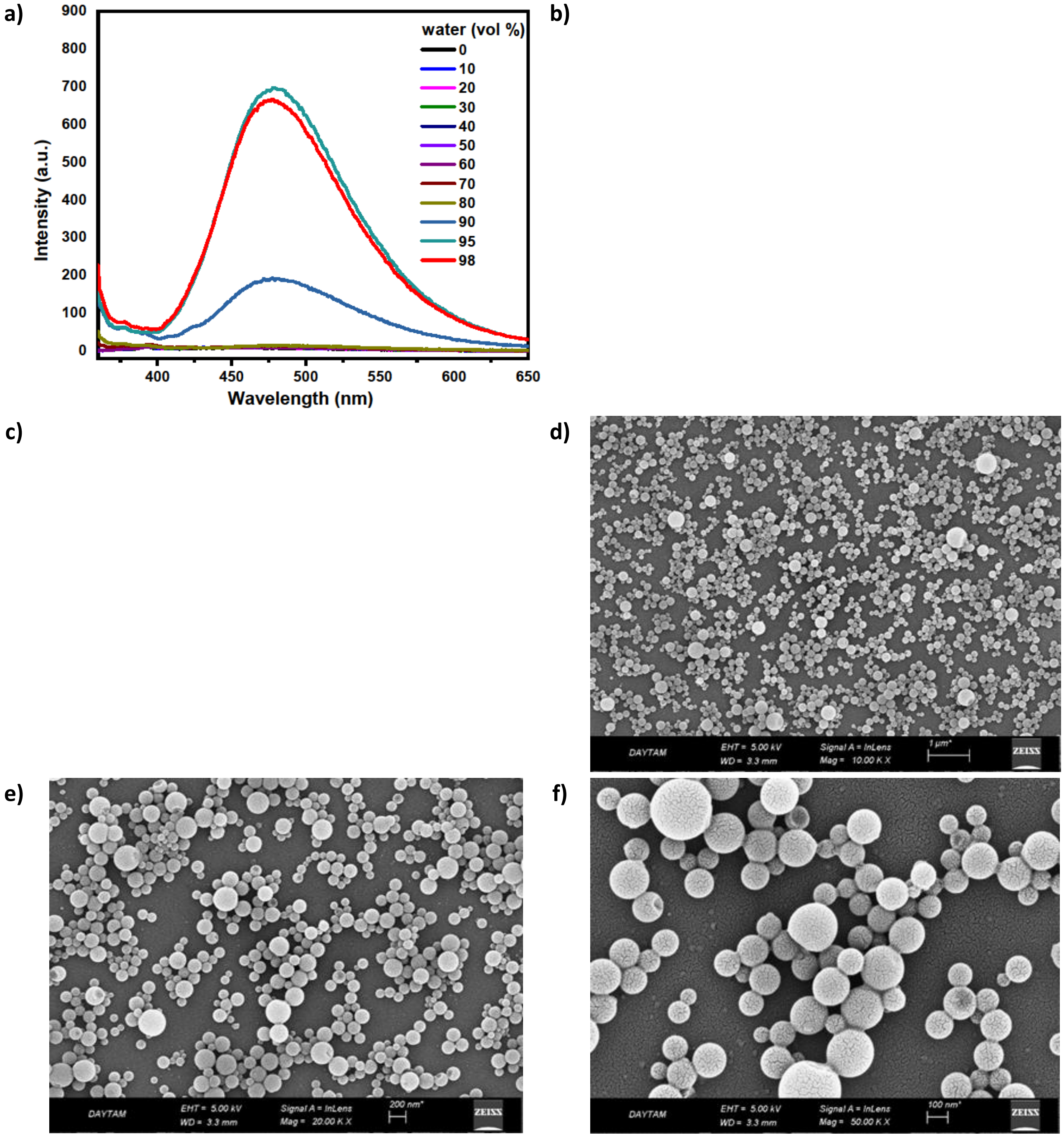

Figure S50. (a) Fluorescence spectra of **36a** in THF and THF–water mixtures with different water fractions; (b) Plot of peak fluorescence intensities of **36a** in THF–water with different water fractions (f_w). Luminogen concentration: 20 μM. Photograph of **36a** in THF–water mixtures (f_w = 0–98%) with different water fractions (20 μM) under 365 nm UV illumination. Photograph of **36a** in water–THF mixed solution (f_w = 0–98%) under a UV lamp. (c) DLS particle size-distribution profile of **36a** in THF–water mixture (5: 95, v: v); (d-f) SEM image.

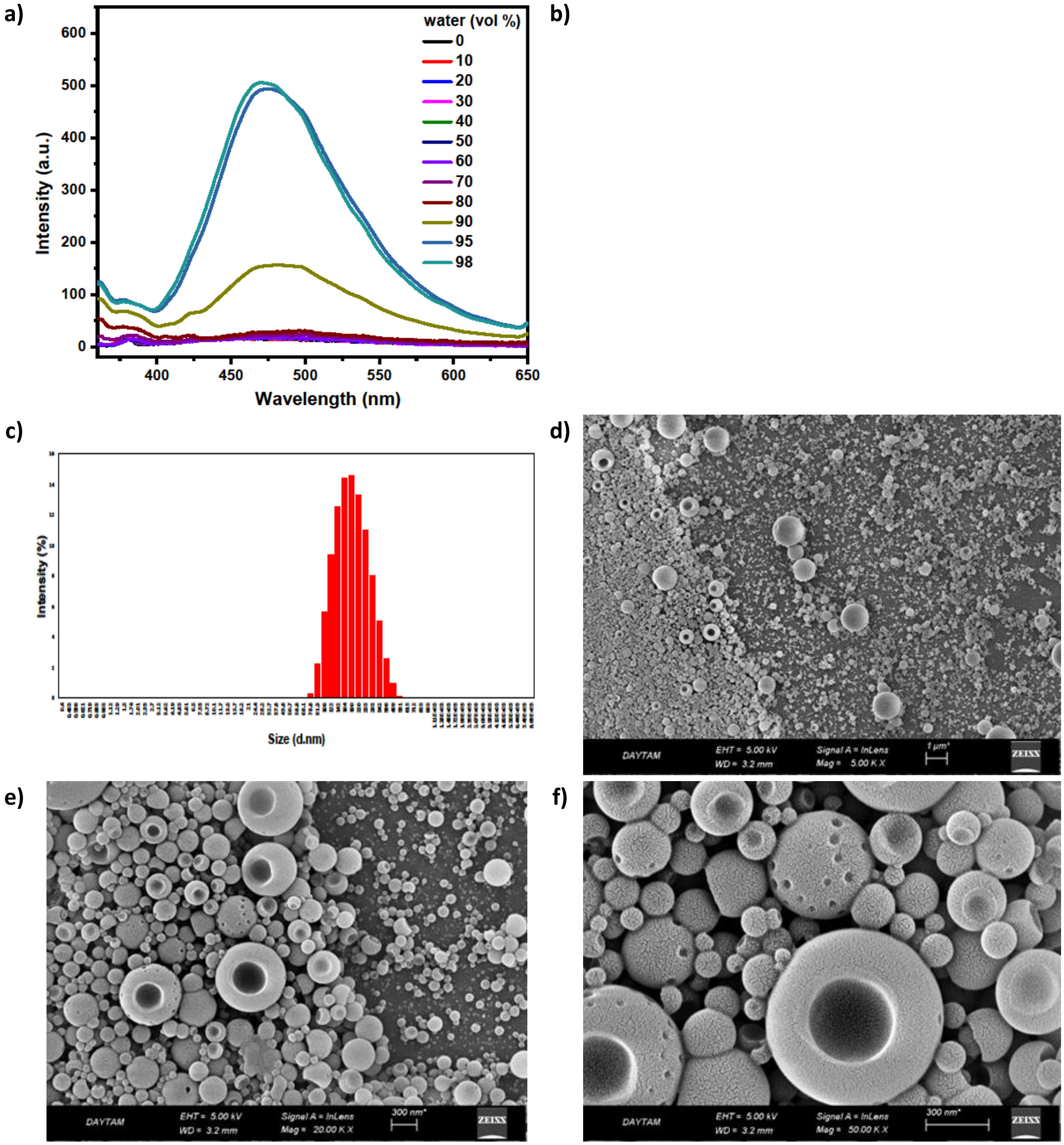

Figure S51. (a) Fluorescence spectra of **36b** in THF and THF–water mixtures with different water fractions; (b) Plot of peak fluorescence intensities of **36b** in THF–water with different water fractions (f_w). Luminogen concentration: 20 μM. Photograph of **36b** in THF–water mixtures (f_w = 0–98%) with different water fractions (20 μM) under 365 nm UV illumination. Photograph of **36b** in water–THF mixed solution (f_w = 0–98%) under a UV lamp. (c) DLS particle size-distribution profile of **36b** in THF–water mixture (5: 95, v: v); (d-f) SEM image.

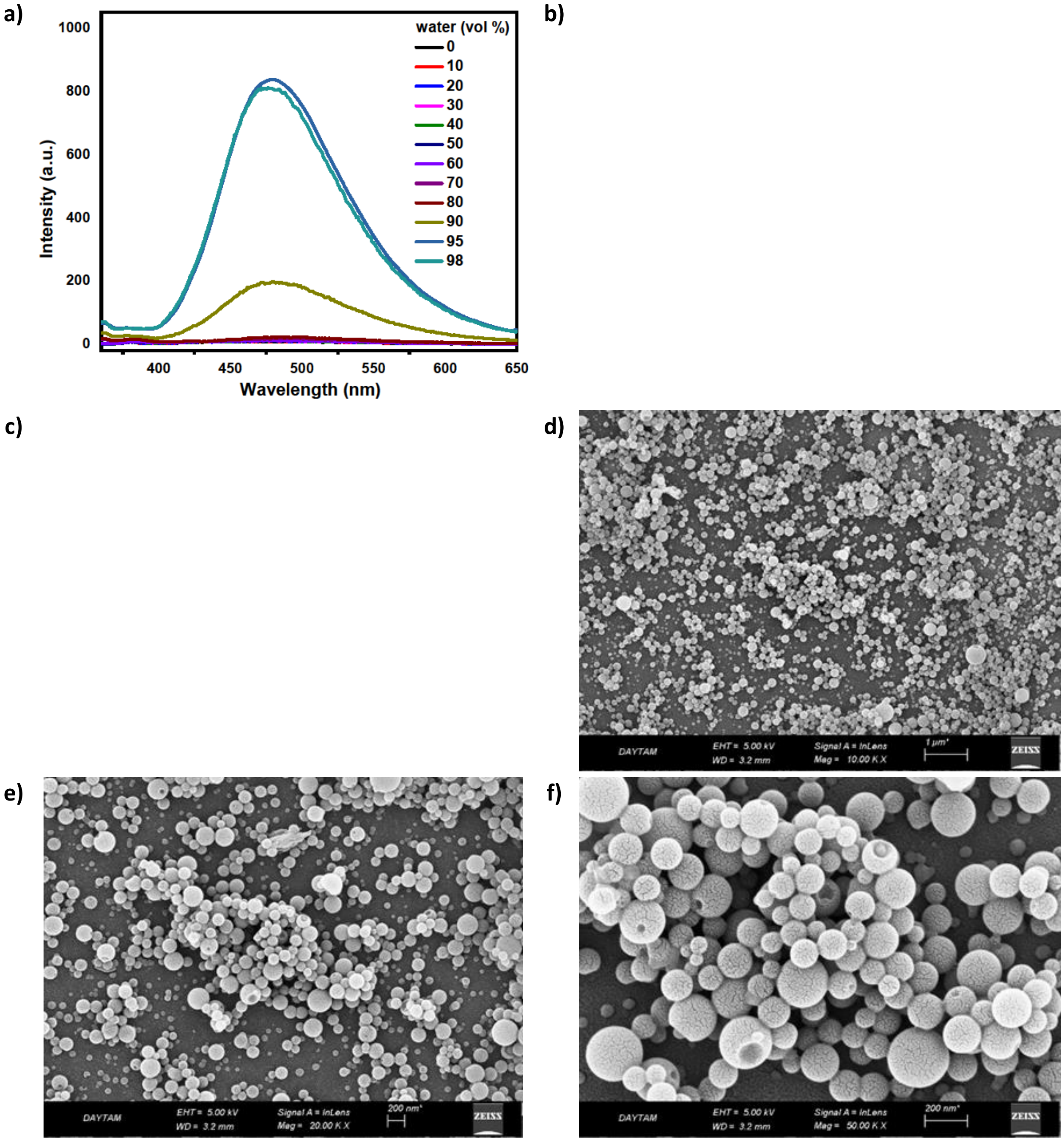

Figure S52. (a) Fluorescence spectra of **36c** in THF and THF–water mixtures with different water fractions; (b) Plot of peak fluorescence intensities of **36c** in THF–water with different water fractions (f_w). Luminogen concentration: 20 μM. Photograph of **36c** in THF–water mixtures (f_w = 0–98%) with different water fractions (20 μM) under 365 nm UV illumination. Photograph of **36c** in water–THF mixed solution (f_w = 0–98%) under a UV lamp. (c) DLS particle size-distribution profile of **36c** in THF–water mixture (5: 95, v: v); (d-f) SEM image.

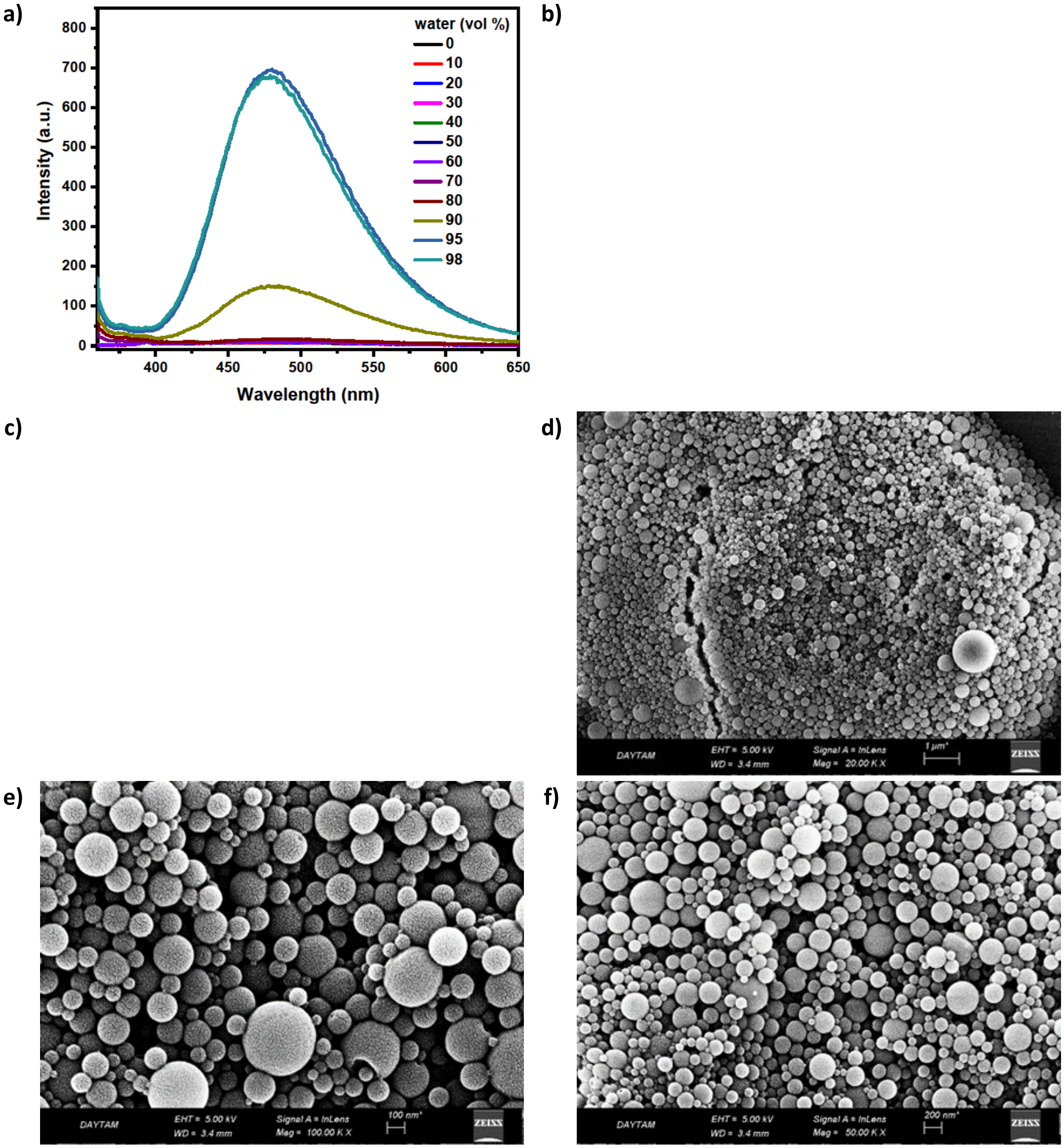

Figure S53. (a) Fluorescence spectra of **36d** in THF and THF–water mixtures with different water fractions; (b) Plot of peak fluorescence intensities of **36d** in THF–water with different water fractions (f_w). Luminogen concentration: 20 μM. Photograph of **36d** in THF–water mixtures (f_w = 0–98%) with different water fractions (20 μM) under 365 nm UV illumination. Photograph of **36d** in water–THF mixed solution (f_w = 0–98%) under a UV lamp. (c) DLS particle size-distribution profile of **36d** in THF–water mixture (5: 95, v: v); (d-f) SEM image.

a)

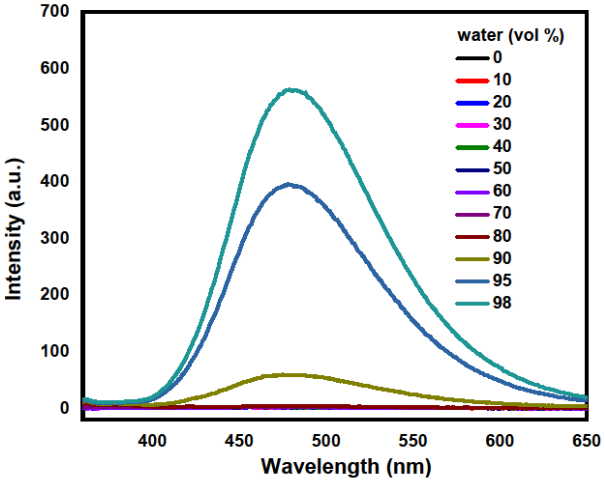

b)

c)

d)

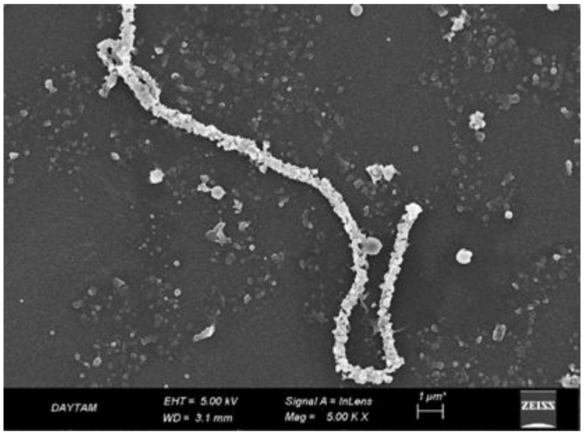

e)

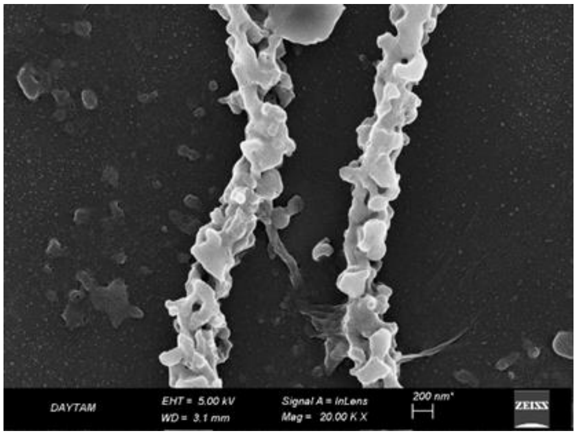

f)

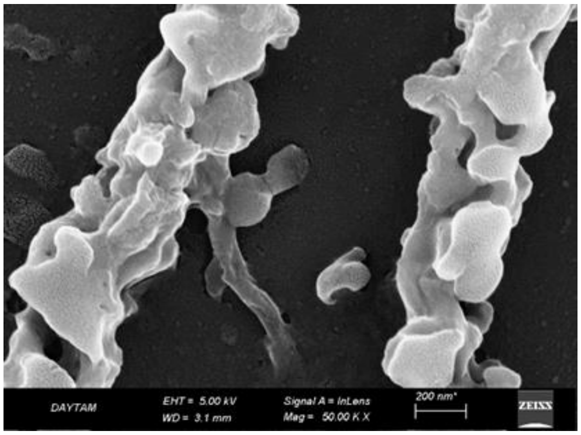

Figure S54. (a) Fluorescence spectra of **36e** in THF and THF–water mixtures with different water fractions; (b) Plot of peak fluorescence intensities of **36e** in THF–water with different water fractions (f_w). Luminogen concentration: 20 μM. Photograph of **36e** in THF–water mixtures (f_w = 0–98%) with different water fractions (20 μM) under 365 nm UV illumination. Photograph of TFTB in water–THF mixed solution (f_w = 0–98%) under a UV lamp. (c) DLS particle size-distribution profile of **36e** in THF–water mixture (2: 98, v: v); (d-f) SEM image.

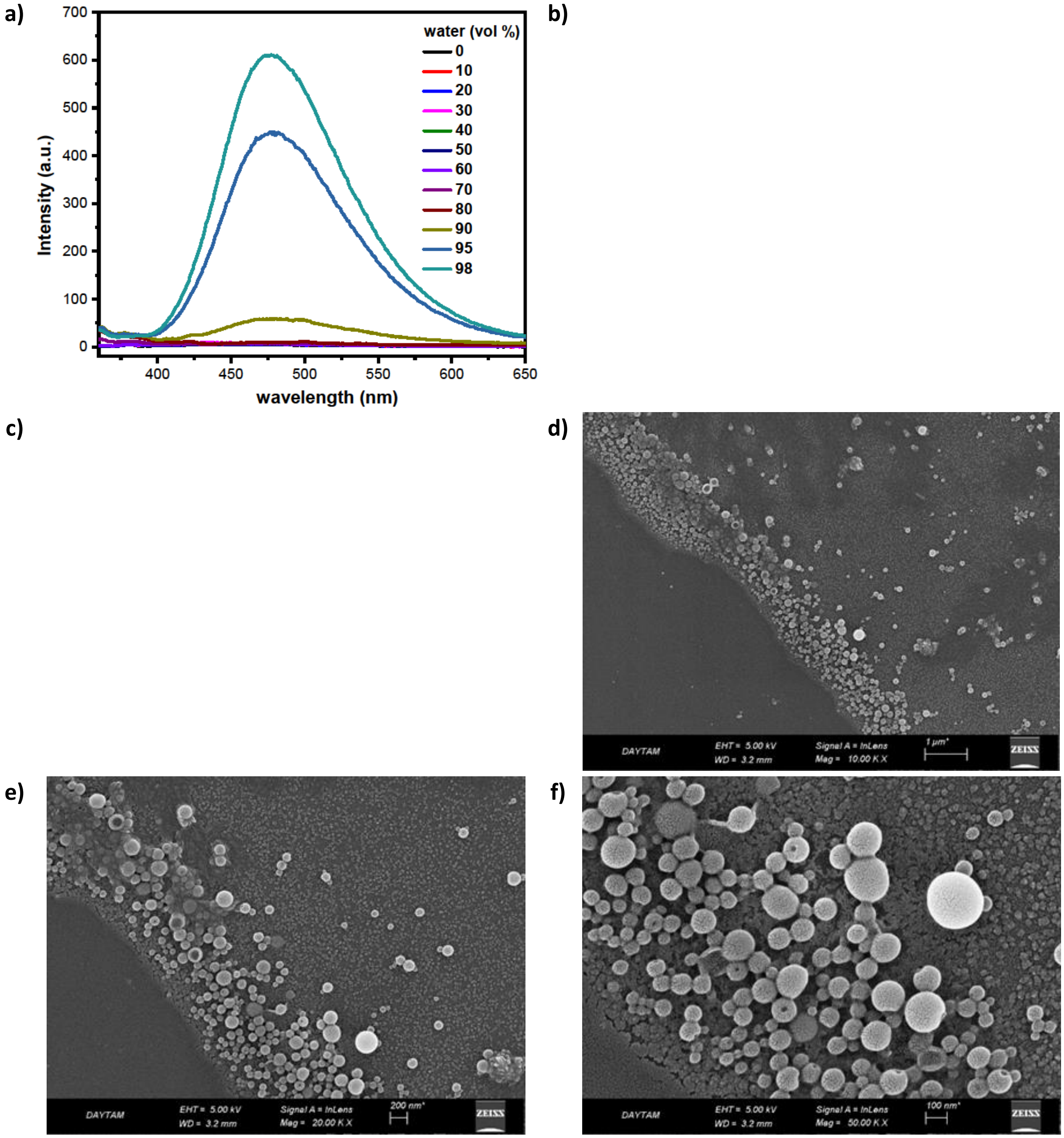

Figure S55. (a) Fluorescence spectra of **36g** in THF and THF–water mixtures with different water fractions; (b) Plot of peak fluorescence intensities of **36g** in THF–water with different water fractions (f_w). Luminogen concentration: 20 μM. Photograph of **36g** in THF–water mixtures (f_w = 0–98%) with different water fractions (20 μM) under 365 nm UV illumination. Photograph of **36g** in water–THF mixed solution (f_w = 0–98%) under a UV lamp. (c) DLS particle size-distribution profile of **36g** in THF–water mixture (2: 98, v: v); (d-f) SEM image.

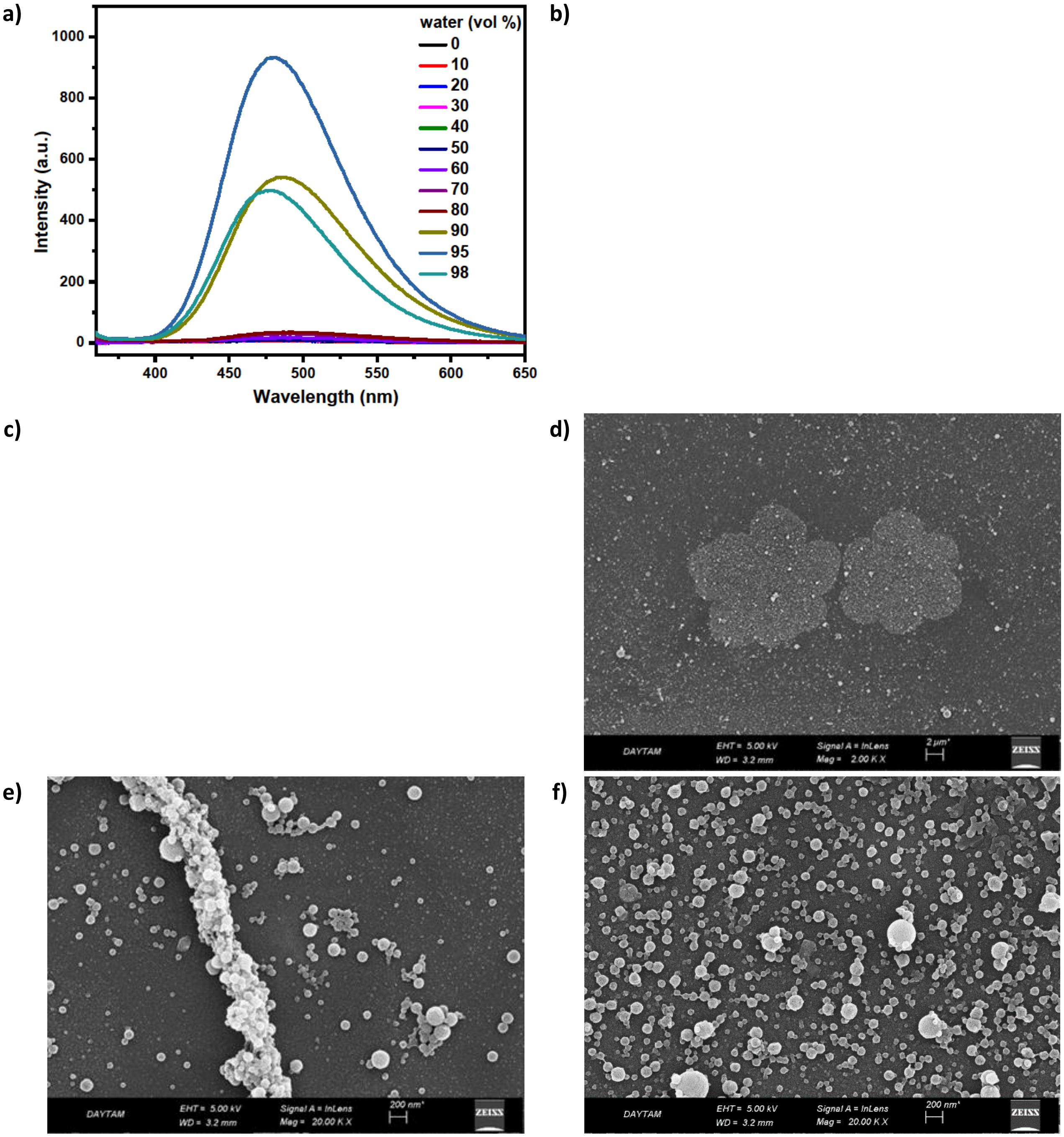

Figure S56. (a) Fluorescence spectra of **37a** in THF and THF–water mixtures with different water fractions; (b) Plot of peak fluorescence intensities of **37a** in THF–water with different water fractions (f_w). Luminogen concentration: 20 μM. Photograph of **37a** in THF–water mixtures (f_w = 0–98%) with different water fractions (20 μM) under 365 nm UV illumination. Photograph of **37a** in water–THF mixed solution (f_w = 0–98%) under a UV lamp. (c) DLS particle size-distribution profile of **37a** in THF–water mixture (5: 95, v: v); (d-f) SEM image.

a)

b)

c)

d)

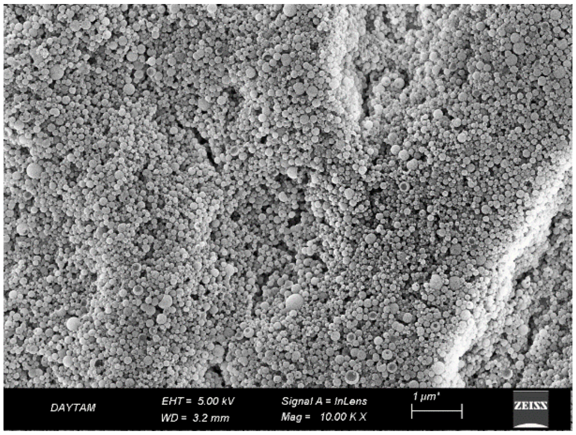

e)

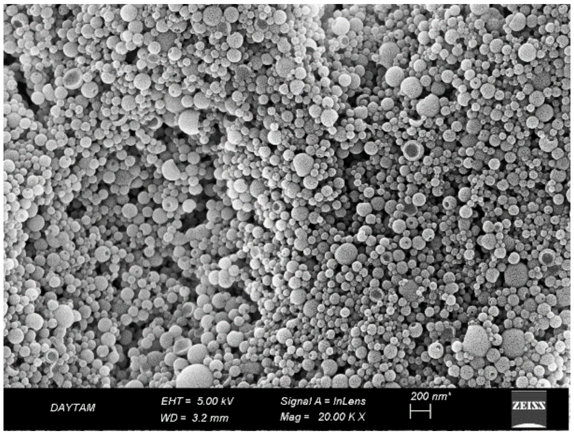

f)

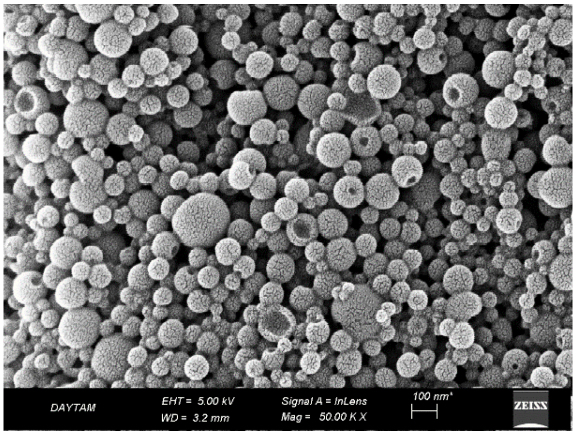

Figure S57. (a) Fluorescence spectra of **37b** in THF and THF–water mixtures with different water fractions; (b) Plot of peak fluorescence intensities of **37b** in THF–water with different water fractions (f_w). Luminogen concentration: 20 μM. Photograph of **37b** in THF–water mixtures (f_w = 0–98%) with different water fractions (20 μM) under 365 nm UV illumination. Photograph of **37b** in water–THF mixed solution (f_w = 0–98%) under a UV lamp. (c) DLS particle size-distribution profile of **37b** in THF–water mixture (5: 95, v: v); (d-f) SEM image.

a) b)

c)

d)

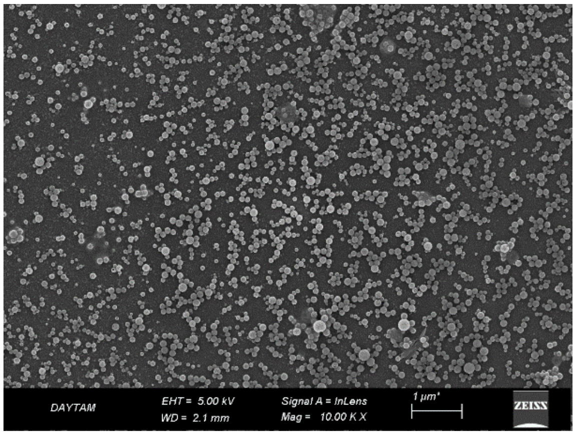

e)

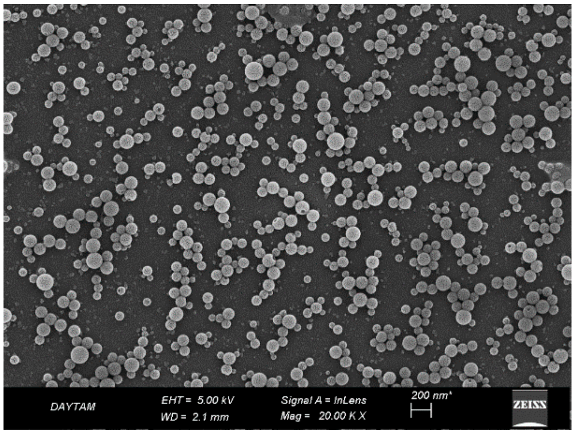

f)

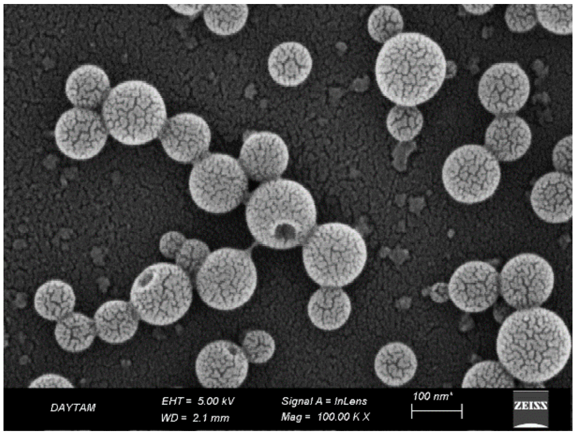

Figure S58. (a) Fluorescence spectra of **37c** in THF and THF–water mixtures with different water fractions; (b) Plot of peak fluorescence intensities of **37c** in THF–water with different water fractions (f_w). Luminogen concentration: 20 μM. Photograph of **37c** in THF–water mixtures (f_w = 0–98%) with different water fractions (20 μM) under 365 nm UV illumination. Photograph of **37c** in water–THF mixed solution (f_w = 0–98%) under a UV lamp. (c) DLS particle size-distribution profile of **37c** in THF–water mixture (10: 90, v: v); (d-f) SEM image.

a) b)

c)

d)

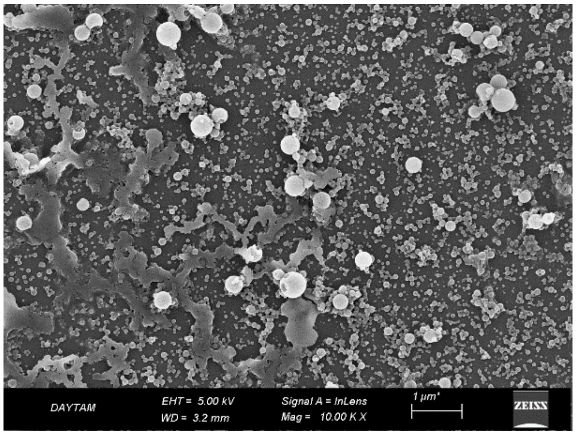

e)

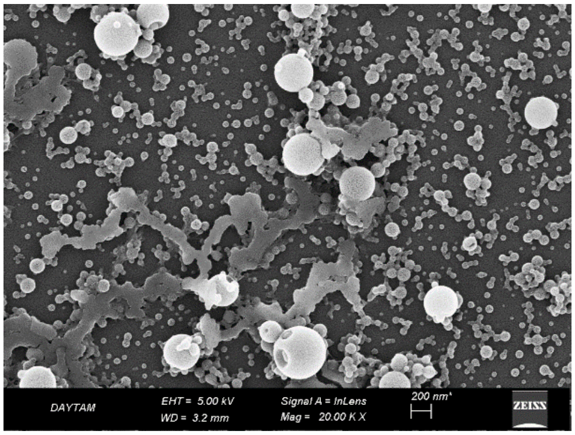

f)

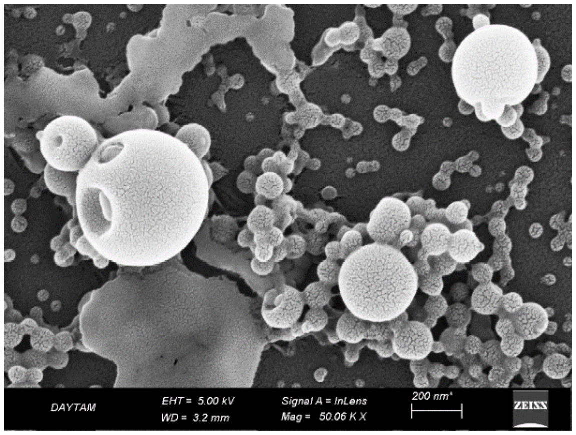

Figure S59. (a) Fluorescence spectra of **37d** in THF and THF–water mixtures with different water fractions; (b) Plot of peak fluorescence intensities of **37d** in THF–water with different water fractions (f_w). Luminogen concentration: 20 μM. Photograph of **37d** in THF–water mixtures (f_w = 0–98%) with different water fractions (20 μM) under 365 nm UV illumination. Photograph of **37d** in water–THF mixed solution (f_w = 0–98%) under a UV lamp. (c) DLS particle size-distribution profile of **37d** in THF–water mixture (5: 95, v: v); (d-f) SEM image.

a)

b)

c)

d)

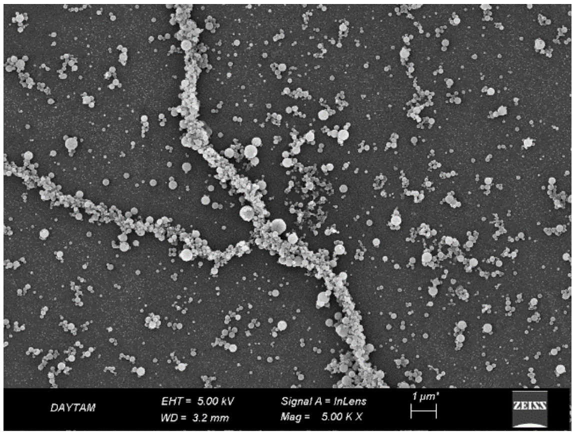

e)

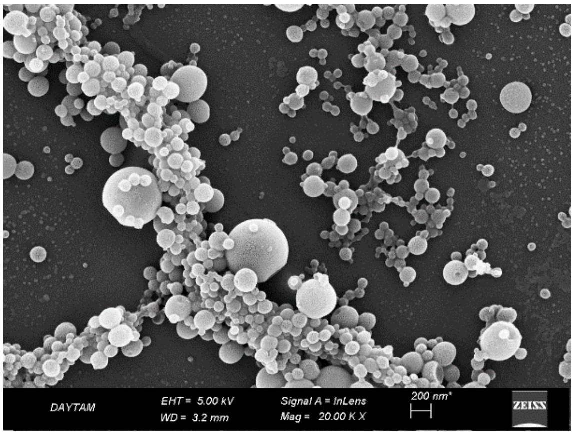

f)

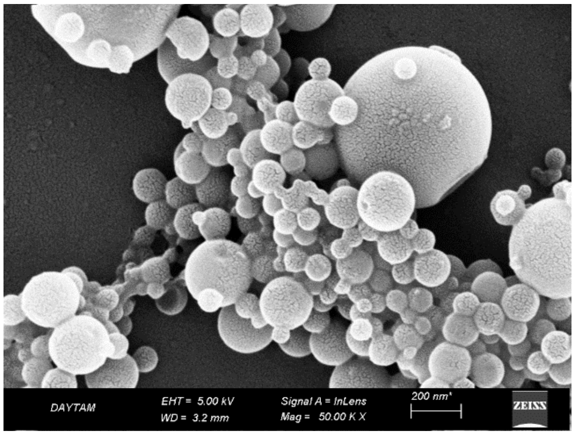

Figure S60. (a) Fluorescence spectra of **37e** in THF and THF–water mixtures with different water fractions; (b) Plot of peak fluorescence intensities of **37e** in THF–water with different water fractions (f_w). Luminogen concentration: 20 μM. Photograph of **37e** in THF–water mixtures (f_w = 0–98%) with different water fractions (20 μM) under 365 nm UV illumination. Photograph of **37e** in water–THF mixed solution (f_w = 0–98%) under a UV lamp. (c) DLS particle size-distribution profile of **37e** in THF–water mixture (5: 95, v: v); (d-f) SEM image.

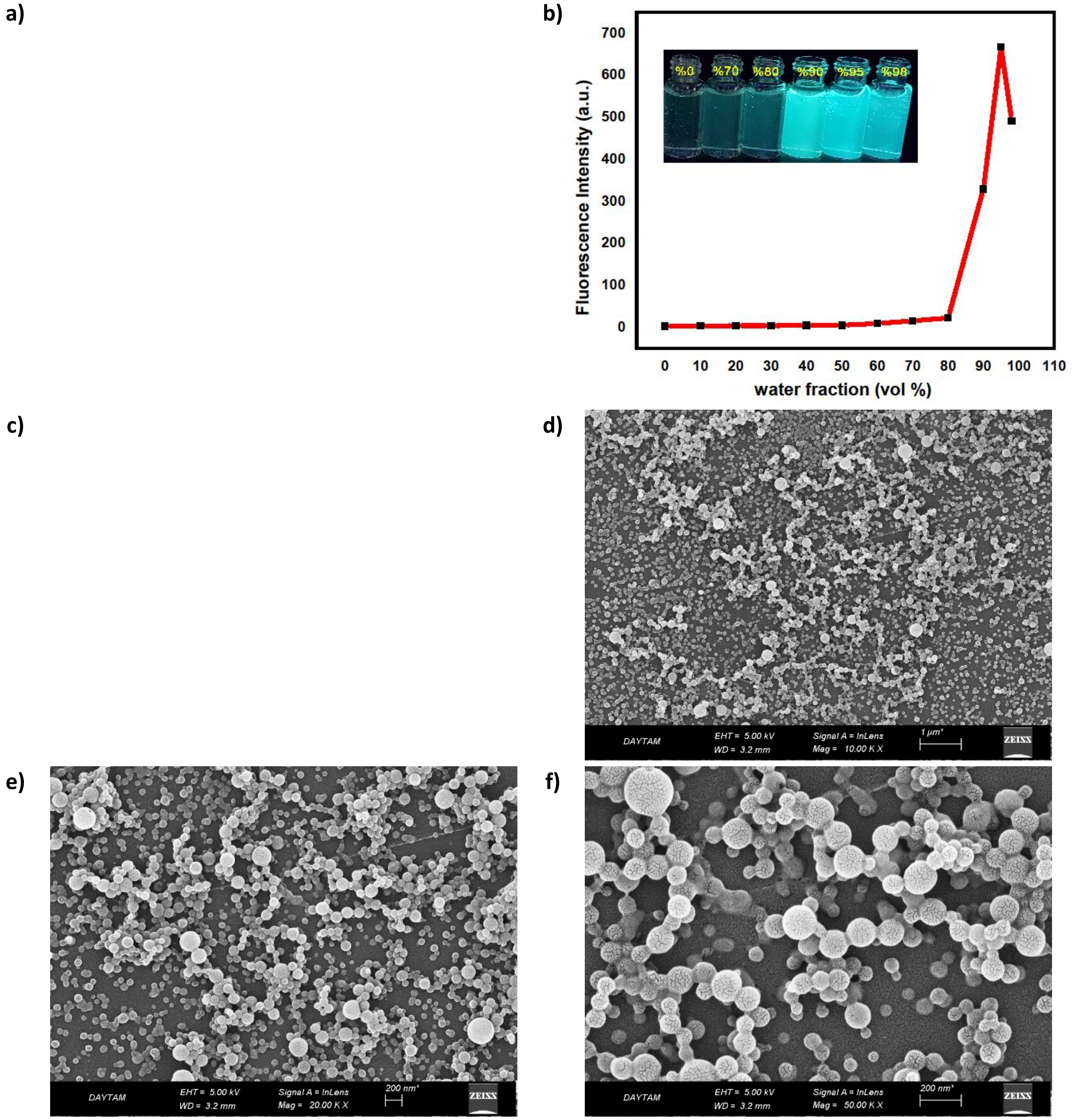

Figure S61. (a) Fluorescence spectra of **37g** in THF and THF–water mixtures with different water fractions; (b) Plot of peak fluorescence intensities of **37g** in THF–water with different water fractions (f_w). Luminogen concentration: 20 μM. Photograph of **37g** in THF–water mixtures (f_w = 0–98%) with different water fractions (20 μM) under 365 nm UV illumination. Photograph of **37g** in water–THF mixed solution (f_w = 0–98%) under a UV lamp. (c) DLS particle size-distribution profile of **37g** in THF–water mixture (2: 98, v: v); (d-f) SEM image.

5. Theoretical Calculations

All calculations were carried out using density functional theory (DFT) at the B3LYP (Becke 3-parameter-Lee Yang-Parr) with the basis set of 6-311G (d,p).

Cartesian cooordinates and total energy of optimized structure

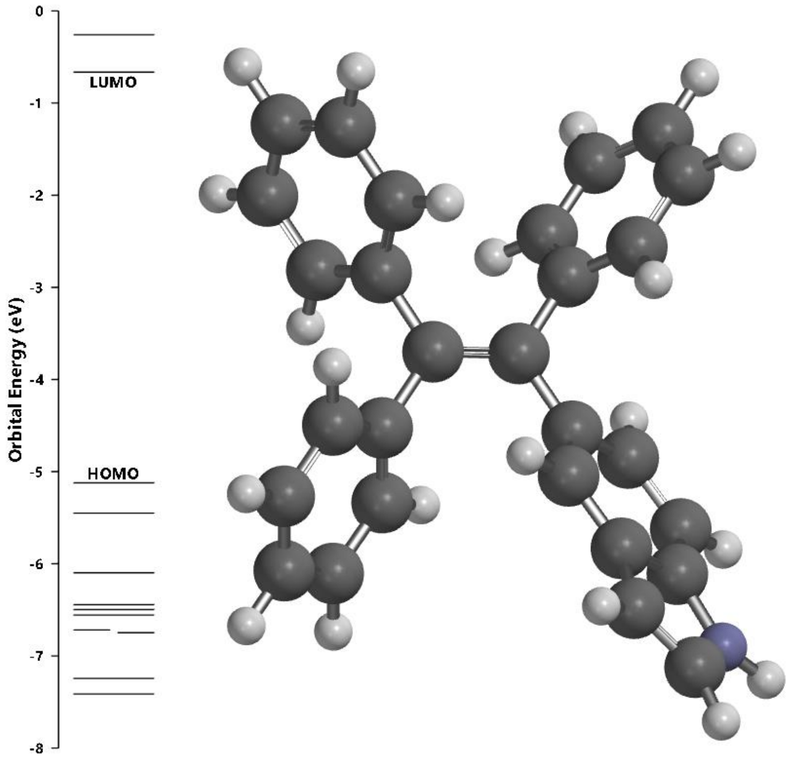

Figure S62. Optimized geometry by semi-emprical calculation using PM6 of compound **9**

Table S1. Atom coordinates and total energy of the optimized geometry of compound **9**

Total Energy: -1134.366945 hartrees zero imaginary frequency

Cartesian Coordinates (Angstroms)

Atom			X	Y	Z
1	C	C1	0.6777816	5.0240045	-1.0658893
2	C	C2	-0.2405919	4.3429463	-1.8715496
3	C	C3	-0.5758337	3.0169453	-1.5884935
4	C	C4	0.0181148	2.3622129	-0.4981403
5	C	C5	0.9386573	3.0466287	0.3106366
6	C	C6	1.2638177	4.3749187	0.0252412
7	C	C7	-0.3457479	0.9559749	-0.1941492
8	C	C8	0.5511372	-0.0529959	-0.2321677
9	C	C9	-1.7740074	0.7658964	0.1552049
10	C	C10	1.9700396	0.1593065	-0.6146205
11	C	C11	0.2136778	-1.4564997	0.1041937
12	C	C12	2.9935133	-0.2240759	0.2670708
13	C	C13	4.3303101	-0.0403291	-0.0930542
14	C	C14	4.6545163	0.5191890	-1.3332870

15 C	C15	3.6368290	0.8979327	-2.2141570
16 C	C16	2.2973919	0.7204121	-1.8589055
17 C	C17	0.5576643	-2.4788351	-0.8302019
18 C	C18	0.2937122	-3.8112735	-0.5787124
19 C	C19	-0.3256986	-4.1277543	0.6472270
20 C	C20	-0.6798739	-3.1141374	1.6020508
21 C	C21	-0.4003185	-1.7675336	1.3116266
22 C	C22	-2.3268331	1.4737740	1.2348477
23 C	C23	-3.6745807	1.3077053	1.5611849
24 C	C24	-4.4785970	0.4428124	0.8116659
25 C	C25	-3.9307274	-0.2590362	-0.2667948
26 C	C26	-2.5824767	-0.1011431	-0.5967778
27 N	N27	-0.7101813	-5.3672736	1.1847623
28 C	C28	-1.2970360	-5.1384264	2.4487910
29 C	C29	-1.2893178	-3.7831989	2.7222854
30 H	H30	0.9357014	6.0572210	-1.2877061
31 H	H31	-0.6973817	4.8472912	-2.7209825
32 H	H32	-1.2997951	2.4896442	-2.2076623
33 H	H33	1.4031229	2.5364690	1.1530271
34 H	H34	1.9783239	4.9042161	0.6535672
35 H	H35	2.7405462	-0.6685476	1.2289644
36 H	H36	5.1215673	-0.3360288	0.5934326
37 H	H37	5.6966135	0.6597848	-1.6118299
38 H	H38	3.8872062	1.3359380	-3.1786263
39 H	H39	1.5050621	1.0257108	-2.5411605
40 H	H40	1.0443858	-2.1869054	-1.7619606
41 H	H41	0.5524733	-4.5872451	-1.2906886
42 H	H42	-0.6717970	-0.9905021	2.0249503
43 H	H43	-1.7027601	2.1537419	1.8129183
44 H	H44	-4.0998136	1.8549291	2.4005550
45 H	H45	-5.5280612	0.3155908	1.0678379
46 H	H46	-4.5547632	-0.9344629	-0.8493773
47 H	H47	-2.1525713	-0.6571905	-1.4286374
48 H	H48	-0.5893653	-6.2585652	0.7483745
49 H	H49	-1.6689632	-5.9525531	3.0456202
50 H	H50	-1.6650719	-3.2966827	3.5994959

Table S2. Atomic charges of compound **9** calculated by B3LYP/6-31G (d,p) method

Atom Label	Natural charge	Mulliken charge	Electrostatic charge
**C1**	-0.240	-0.126	-0.180
**C2**	-0.230	-0.136	-0.037
**C3**	-0.219	-0.158	-0.357
**C4**	-0.056	+0.078	+0.442
**C5**	-0.216	-0.149	-0.256
**C6**	-0.227	-0.135	-0.099
**C7**	-0.020	-0.074	-0.381
**C8**	-0.006	-0.037	-0.176
**C9**	-0.057	+0.084	+0.453
**C10**	-0.059	+0.074	+0.465

**C11**	-0.083	+0.075	+0.239
**C12**	-0.215	-0.158	-0.352
**C13**	-0.231	-0.137	-0.050
**C14**	-0.239	-0.126	-0.187
**C15**	-0.229	-0.136	-0.081
**C16**	-0.214	-0.152	-0.305
**C17**	-0.221	-0.186	-0.240
**C18**	-0.262	-0.166	-0.268
**C19**	+0.159	+0.305	+0.161
**C20**	-0.095	+0.116	+0.224
**C21**	-0.192	-0.235	-0.385
**C22**	-0.220	-0.160	-0.368
**C23**	-0.231	-0.137	-0.032
**C24**	-0.240	-0.126	-0.186
**C25**	-0.228	-0.135	-0.090
**C26**	-0.213	-0.149	-0.264
**C28**	-0.036	+0.043	-0.095
**C29**	-0.304	-0.219	-0.369
**H30**	+0.235	+0.125	+0.125
**H31**	+0.235	+0.127	+0.111
**H32**	+0.240	+0.133	+0.149
**H33**	+0.243	+0.134	+0.135
**H34**	+0.236	+0.127	+0.121
**H35**	+0.242	+0.136	+0.149
**H36**	+0.235	+0.126	+0.114
**H37**	+0.234	+0.125	+0.128
**H38**	+0.235	+0.127	+0.119
**H39**	+0.243	+0.136	+0.150
**H40**	+0.243	+0.133	+0.132

**H41**	+0.235	+0.123	+0.164
**H42**	+0.245	+0.137	+0.191
**H43**	+0.241	+0.134	+0.152
**H44**	+0.235	+0.126	+0.110
**H45**	+0.234	+0.125	+0.126
**H46**	+0.235	+0.127	+0.118
**H47**	+0.243	+0.136	+0.147
**H48**	+0.430	+0.328	+0.343
**H49**	+0.232	+0.151	+0.168
**H50**	+0.243	+0.131	+0.188
**N27**	-0.572	-0.687	-0.365

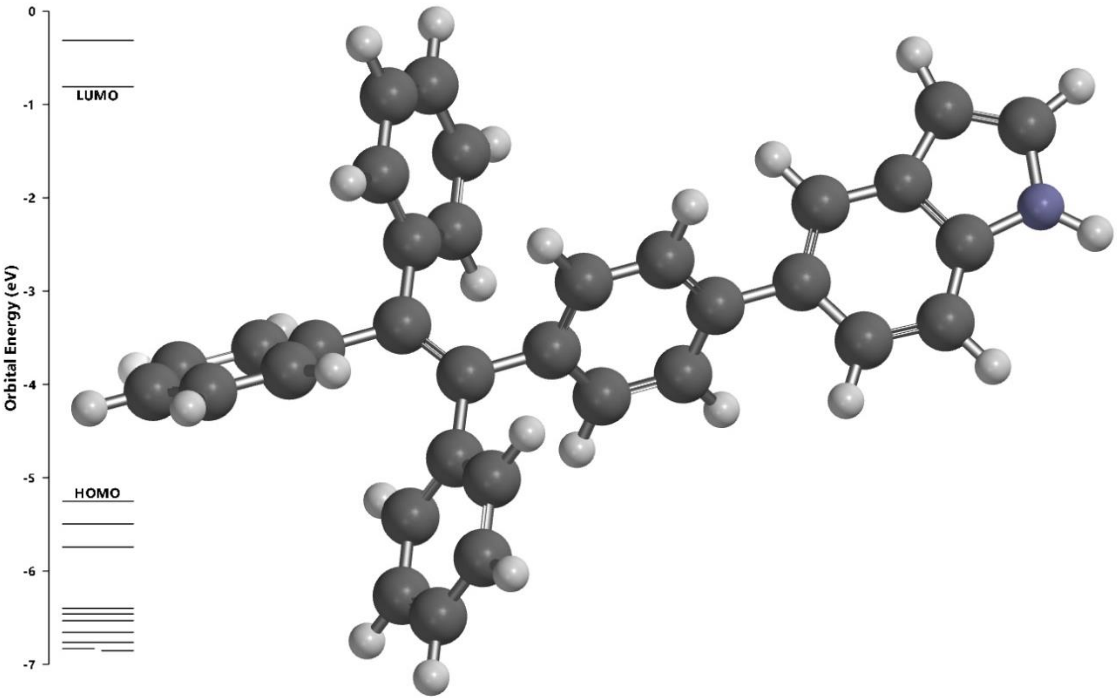

Figure S63. Optimized geometry by semi-emprical calculation using PM6 of compound **32**

Table S3. Atom coordinates and total energy of the optimized geometry of compound **32**

Total Energy: -1365.423022 hartrees zero imaginary frequency

Cartesian Coordinates (Angstroms)

```
   Atom          X             Y             Z
 ---------    ------------- ------------- -------------
```

1	C	C1	0.8814618	6.4703347	-1.5904399
2	C	C2	0.5157586	6.1826560	-0.2714090
3	C	C3	0.2239661	4.8701473	0.1064726
4	C	C4	0.3046678	3.8340826	-0.8380097
5	C	C5	0.6710689	4.1243504	-2.1615342
6	C	C6	0.9575126	5.4402691	-2.5331987
7	C	C7	-0.0188495	2.4437710	-0.4347585
8	C	C8	-1.3833211	2.2733120	0.1213379
9	C	C9	0.8537592	1.4217018	-0.5633688
10	C	C10	0.5203782	0.0265522	-0.1878677
11	C	C11	2.2281437	1.5969836	-1.0931775
12	C	C12	-1.5663460	1.7547025	1.4128978
13	C	C13	-2.8556975	1.6150417	1.9323095
14	C	C14	-3.9661587	1.9888736	1.1690459
15	C	C15	-3.7854424	2.5057498	-0.1180556
16	C	C16	-2.4992570	2.6519090	-0.6424500
17	C	C17	1.2930092	-0.6410361	0.7759142
18	C	C18	0.9924554	-1.9573711	1.1261890
19	C	C19	-0.0812723	-2.6245964	0.5144041
20	C	C20	-0.8502195	-1.9571965	-0.4524641
21	C	C21	-0.5516105	-0.6397909	-0.8017832
22	C	C22	2.6354760	0.8724632	-2.2250754
23	C	C23	3.9321054	1.0247709	-2.7212869
24	C	C24	4.8297613	1.8919195	-2.0896938
25	C	C25	4.4268433	2.6105729	-0.9597453
26	C	C26	3.1298553	2.4669863	-0.4605912
27	C	C27	-0.4026109	-4.0194891	0.8875632
28	C	C28	0.6141410	-5.0129721	0.7754174
29	C	C29	0.3774419	-6.3334998	1.1066166
30	C	C30	-0.9134971	-6.6640250	1.5650697
31	C	C31	-1.9520506	-5.6781959	1.6857026
32	C	C32	-1.6767281	-4.3444441	1.3373338
33	N	N33	-1.4520031	-7.8962916	1.9727069
34	C	C34	-2.7990571	-7.6902887	2.3413568
35	C	C35	-3.1239947	-6.3564200	2.1763057
36	H	H36	1.1066353	7.4938444	-1.8823393
37	H	H37	0.4558533	6.9837975	0.4631832
38	H	H38	-0.0703699	4.6471864	1.1311035
39	H	H39	0.7403575	3.3207176	-2.8935565
40	H	H40	1.2435689	5.6622968	-3.5599273
41	H	H41	-0.7023389	1.4527409	2.0035782
42	H	H42	-2.9945931	1.2110044	2.9338128
43	H	H43	-4.9691032	1.8777063	1.5755966
44	H	H44	-4.6493151	2.7967935	-0.7132124
45	H	H45	-2.3580501	3.0622205	-1.6415032
46	H	H46	2.1296877	-0.1274709	1.2486729
47	H	H47	1.5877227	-2.4686860	1.8814923
48	H	H48	-1.6808265	-2.4723143	-0.9336497
49	H	H49	-1.1555484	-0.1225028	-1.5468714
50	H	H50	1.9390524	0.1900856	-2.7107095
51	H	H51	4.2447443	0.4646115	-3.6008426
52	H	H52	5.8398651	2.0068597	-2.4768059
53	H	H53	5.1238765	3.2870751	-0.4680950
54	H	H54	2.8128139	3.0351442	0.4129985
55	H	H55	1.5963965	-4.7110967	0.4112710
56	H	H56	1.1497774	-7.0901360	1.0181726
57	H	H57	-2.4537700	-3.5878733	1.4284187
58	H	H58	-0.9650576	-8.7690034	1.9973929
59	H	H59	-3.4122027	-8.5033551	2.6888915
60	H	H60	-4.0688642	-5.8911790	2.3711939

Table S4. Atomic charges of compound **32** calculated by B3LYP/6-31G (d,p) method

Atom Label	Natural charge	Mulliken charge	Electrostatic charge
C1	-0.239	-0.126	-0.181
C2	-0.230	-0.136	-0.039
C3	-0.217	-0.159	-0.357
C4	-0.059	+0.078	+0.450
C5	-0.214	-0.150	-0.267
C6	-0.228	-0.136	-0.094
C7	-0.014	-0.054	-0.333
C8	-0.059	+0.080	+0.477
C9	-0.010	-0.060	-0.266
C10	-0.066	+0.085	+0.339
C11	-0.059	+0.080	+0.418
C12	-0.213	-0.151	-0.289
C13	-0.229	-0.137	-0.086
C14	-0.239	-0.126	-0.187
C15	-0.231	-0.137	-0.033
C16	-0.217	-0.158	-0.373
C17	-0.211	-0.167	-0.308
C18	-0.219	-0.172	-0.151
C19	-0.047	+0.069	+0.122
C20	-0.215	-0.171	-0.243
C21	-0.208	-0.159	-0.187
C22	-0.217	-0.159	-0.345
C23	-0.230	-0.136	-0.042
C24	-0.239	-0.126	-0.181
C25	-0.228	-0.136	-0.095
C26	-0.214	-0.151	-0.264
C27	-0.071	+0.060	+0.110

**C28**	-0.227	-0.188	-0.185
**C29**	-0.260	-0.164	-0.290
**C30**	+0.157	+0.304	+0.191
**C31**	-0.094	+0.120	+0.259
**C32**	-0.204	-0.246	-0.393
**C34**	-0.034	+0.045	-0.058
**C35**	-0.307	-0.221	-0.418
**H36**	+0.235	+0.126	+0.126
**H37**	+0.236	+0.128	+0.113
**H38**	+0.241	+0.135	+0.145
**H39**	+0.244	+0.137	+0.143
**H40**	+0.236	+0.128	+0.121
**H41**	+0.244	+0.138	+0.151
**H42**	+0.236	+0.129	+0.119
**H43**	+0.235	+0.126	+0.127
**H44**	+0.236	+0.128	+0.112
**H45**	+0.241	+0.135	+0.147
**H46**	+0.241	+0.134	+0.147
**H47**	+0.240	+0.132	+0.128
**H48**	+0.241	+0.132	+0.148
**H49**	+0.243	+0.135	+0.134
**H50**	+0.242	+0.136	+0.146
**H51**	+0.236	+0.128	+0.113
**H52**	+0.235	+0.126	+0.127
**H53**	+0.236	+0.128	+0.121
**H54**	+0.243	+0.136	+0.143
**H55**	+0.241	+0.130	+0.130
**H56**	+0.235	+0.123	+0.164
**H57**	+0.241	+0.133	+0.183

**H58**	+0.430	+0.328	+0.352
**H59**	+0.233	+0.152	+0.162
**H60**	+0.243	+0.130	+0.200
**N33**	-0.572	-0.687	-0.400

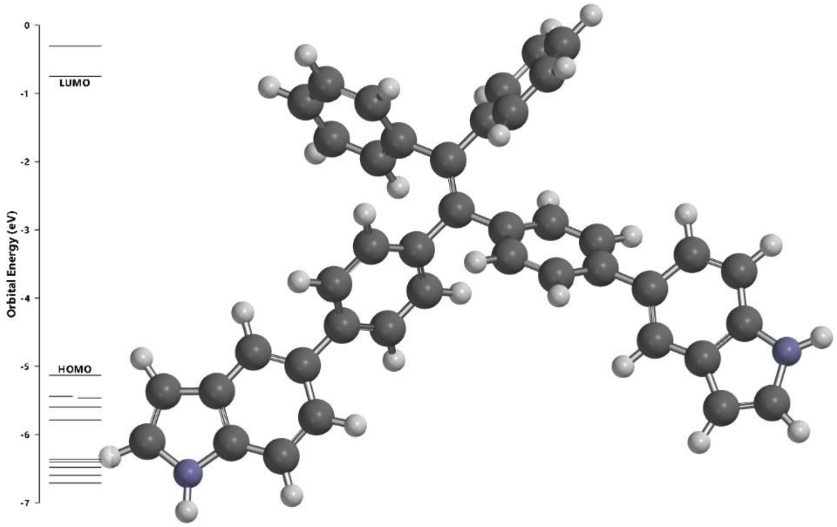

Figure S64. Optimized geometry by semi-emprical calculation using PM6 of compound **33**

Table S5. Atom coordinates and total energy of the optimized geometry of compound **33**

Total Energy -1728.044409 hartrees zero imaginary frequency

Cartesian Coordinates (Angstroms)

Atom		X	Y	Z
1 C	C1	-1.3073512	3.4849104	0.2300982
2 C	C2	-1.5960414	2.0723415	0.5783037
3 C	C3	-2.8791515	1.8726350	1.2947749
4 C	C4	-0.7626883	1.0565680	0.2681826
5 C	C5	-1.0388566	-0.3529492	0.6366763
6 C	C6	0.5063039	1.2542624	-0.4728420
7 C	C7	-1.1928935	-0.7230376	1.9816060
8 C	C8	-1.4375006	-2.0549500	2.3183705
9 C	C9	-1.5316721	-3.0327340	1.3153416
10 C	C10	-1.3755909	-2.6612957	-0.0301719
11 C	C11	-1.1287370	-1.3305549	-0.3673311
12 C	C12	1.7204522	0.8399182	0.0976385
13 C	C13	2.9170726	1.0116259	-0.5986221
14 C	C14	2.9147024	1.5933900	-1.8767914
15 C	C15	1.6991581	2.0028391	-2.4482406
16 C	C16	0.5026977	1.8357851	-1.7504379
17 C	C17	4.1832273	1.7718867	-2.6166322
18 C	C18	-1.7940656	-4.4439367	1.6730001
19 C	C19	-0.8772986	-5.4451580	1.2366273
20 C	C20	-1.0647680	-6.7808401	1.5370547
21 C	C21	-2.2050135	-7.1199173	2.2926184

22 C	C22	-3.1409523	-6.1266803	2.7421125
23 C	C23	-2.9168824	-4.7764542	2.4211201
24 C	C24	5.0077297	0.6805280	-2.8615915
25 C	C25	6.2050524	0.8819522	-3.5701852
26 C	C26	6.5418653	2.2051764	-4.0177786
27 C	C27	5.7059003	3.3114796	-3.7666649
28 C	C28	4.5362062	3.0772213	-3.0693003
29 N	N29	-2.6638319	-8.3682195	2.7464527
30 C	C30	-3.8617483	-8.1648045	3.4649451
31 C	C31	-4.1702262	-6.8169432	3.4757895
32 C	C32	7.2689350	0.0086105	-3.9940056
33 C	C33	8.2012020	0.7777208	-4.6658889
34 N	N34	7.7729573	2.1226093	-4.6903275
35 C	C35	-3.1039677	2.5259640	2.5171778
36 C	C36	-4.3152037	2.3491185	3.1898590
37 C	C37	-5.3086512	1.5281556	2.6463349
38 C	C38	-5.0878835	0.8808867	1.4264792
39 C	C39	-3.8772837	1.0496450	0.7499666
40 C	C40	-2.2078589	4.2024595	-0.5734962
41 C	C41	-1.9442724	5.5353588	-0.8968349
42 C	C42	-0.7889732	6.1607971	-0.4165634
43 C	C43	0.1058391	5.4488743	0.3883298
44 C	C44	-0.1483375	4.1136298	0.7114079
45 H	H45	-1.1288029	0.0336700	2.7630095
46 H	H46	-1.5535172	-2.3387552	3.3637231
47 H	H47	-1.4533046	-3.4151590	-0.8124450
48 H	H48	-1.0033755	-1.0463598	-1.4116550
49 H	H49	1.7252752	0.3808681	1.0859551
50 H	H50	3.8581017	0.6973641	-0.1489107
51 H	H51	1.6906524	2.4481715	-3.4422310
52 H	H52	-0.4375337	2.1630730	-2.1936259
53 H	H53	-0.0071876	-5.1344807	0.6579220
54 H	H54	-0.3655858	-7.5420907	1.2079315
55 H	H55	-3.6205272	-4.0155092	2.7527993
56 H	H56	4.7392475	-0.3179370	-2.5225172
57 H	H57	5.9755246	4.3048249	-4.1093805
58 H	H58	3.8603851	3.9033491	-2.8470617
59 H	H59	-2.2185971	-9.2489945	2.5878846
60 H	H60	-4.3933780	-8.9893887	3.9068084
61 H	H61	-5.0156137	-6.3497927	3.9386756
62 H	H62	7.3102657	-1.0458119	-3.8109881
63 H	H63	9.1274613	0.4794836	-5.1251465
64 H	H64	8.2616613	2.8850731	-5.1134820
65 H	H65	-2.3337352	3.1720891	2.9356947
66 H	H66	-4.4862445	2.8537990	4.1391078
67 H	H67	-6.2514198	1.3930144	3.1721157
68 H	H68	-5.8595476	0.2403203	1.0027761
69 H	H69	-3.7006540	0.5359734	-0.1940820
70 H	H70	-3.1117472	3.7174953	-0.9394529
71 H	H71	-2.6417572	6.0887917	-1.5230123
72 H	H72	-0.5874202	7.1996707	-0.6683684
73 H	H73	1.0053031	5.9341754	0.7632339
74 H	H74	0.5544712	3.5551983	1.3281607

Table S6. Atomic charges of compound **33** calculated by B3LYP/6-31G (d,p) method

Atom Label	Natural charge	Mulliken charge	Electrostatic charge
**C1**	-0.058	+0.080	+0.506
**C2**	-0.016	-0.061	-0.368

**C3**	-0.058	+0.081	+0.450
**C4**	-0.008	-0.058	-0.240
**C5**	-0.065	+0.087	+0.387
**C6**	-0.066	+0.088	+0.359
**C7**	-0.208	-0.160	-0.230
**C8**	-0.215	-0.170	-0.219
**C9**	-0.048	+0.068	+0.112
**C10**	-0.219	-0.172	-0.135
**C11**	-0.210	-0.168	-0.339
**C12**	-0.210	-0.169	-0.298
**C13**	-0.218	-0.172	-0.168
**C14**	-0.048	+0.069	+0.119
**C15**	-0.217	-0.172	-0.201
**C16**	-0.207	-0.160	-0.236
**C17**	-0.071	+0.060	+0.096
**C18**	-0.071	+0.060	+0.088
**C19**	-0.227	-0.188	-0.178
**C20**	-0.260	-0.164	-0.283
**C21**	+0.157	+0.305	+0.186
**C22**	-0.094	+0.119	+0.240
**C23**	-0.204	-0.245	-0.367
**C24**	-0.204	-0.245	-0.385
**C25**	-0.094	+0.120	+0.253
**C26**	+0.157	+0.305	+0.199
**C27**	-0.261	-0.164	-0.299
**C28**	-0.227	-0.189	-0.176
**C30**	-0.034	+0.044	-0.065
**C31**	-0.307	-0.221	-0.405
**C32**	-0.307	-0.221	-0.403

**C33**	-0.034	+0.045	-0.074
**C35**	-0.217	-0.158	-0.364
**C36**	-0.230	-0.136	-0.034
**C37**	-0.239	-0.126	-0.185
**C38**	-0.228	-0.136	-0.093
**C39**	-0.214	-0.150	-0.269
**C40**	-0.218	-0.158	-0.391
**C41**	-0.230	-0.136	-0.027
**C42**	-0.239	-0.126	-0.184
**C43**	-0.228	-0.136	-0.097
**C44**	-0.214	-0.150	-0.285
**H45**	+0.243	+0.135	+0.144
**H46**	+0.241	+0.132	+0.145
**H47**	+0.240	+0.131	+0.127
**H48**	+0.241	+0.134	+0.151
**H49**	+0.241	+0.134	+0.140
**H50**	+0.240	+0.131	+0.137
**H51**	+0.240	+0.131	+0.137
**H52**	+0.243	+0.135	+0.144
**H53**	+0.241	+0.130	+0.129
**H54**	+0.235	+0.123	+0.161
**H55**	+0.241	+0.132	+0.177
**H56**	+0.241	+0.131	+0.176
**H57**	+0.235	+0.123	+0.164
**H58**	+0.241	+0.131	+0.133
**H59**	+0.430	+0.328	+0.352
**H60**	+0.233	+0.151	+0.163
**H61**	+0.242	+0.130	+0.197
**H62**	+0.242	+0.130	+0.196

**H63**	+0.233	+0.151	+0.166
**H64**	+0.430	+0.328	+0.355
**H65**	+0.241	+0.134	+0.148
**H66**	+0.235	+0.127	+0.111
**H67**	+0.235	+0.126	+0.126
**H68**	+0.236	+0.128	+0.119
**H69**	+0.244	+0.137	+0.147
**H70**	+0.241	+0.135	+0.153
**H71**	+0.235	+0.127	+0.111
**H72**	+0.234	+0.125	+0.126
**H73**	+0.236	+0.128	+0.122
**H74**	+0.244	+0.137	+0.145
**N29**	-0.572	-0.686	-0.396
**N34**	-0.572	-0.687	-0.404

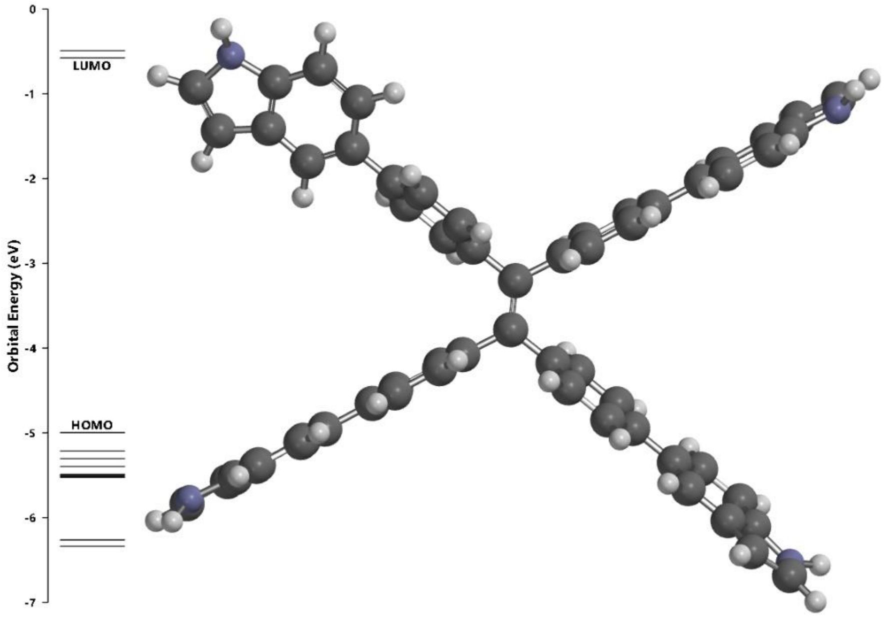

Figure S65. Optimized geometry by semi-emprical calculation using PM6 of compound **34**

Table S7. Atom coordinates and total energy of the optimized geometry of compound **34**

Total Energy -2453.286868 hartrees; zero imaginary frequency

Cartesian Coordinates (Angstroms)

Atom		X	Y	Z
1 C	C1	-0.0147141	1.8426653	-0.4240910
2 C	C2	-0.3599736	0.4790826	0.0197733
3 C	C3	-1.6225481	0.3929331	0.7768836
4 C	C4	0.4101999	-0.5905077	-0.2453572
5 C	C5	0.0663977	-1.9561468	0.1925972
6 C	C6	1.6724243	-0.4980444	-1.0029479
7 C	C7	0.6308348	-2.4808482	1.3558773
8 C	C8	0.3222005	-3.7747886	1.7564355
9 C	C9	-0.5474492	-4.5596594	0.9947094
10 C	C10	-1.1080964	-4.0318869	-0.1713839
11 C	C11	-0.8048590	-2.7360463	-0.5694275
12 C	C12	2.8847926	-0.3512500	-0.3275173
13 C	C13	4.0747251	-0.2827037	-1.0410385
14 C	C14	4.0655934	-0.3659076	-2.4359177
15 C	C15	2.8498162	-0.5160381	-3.1083559
16 C	C16	1.6588580	-0.5805233	-2.3960606
17 C	C17	5.3255573	-0.2934673	-3.1886774
18 C	C18	-0.8716755	-5.9288117	1.4186767
19 C	C19	-0.7969401	-6.9772703	0.4726684
20 C	C20	-1.0928750	-8.2814292	0.8128746
21 C	C21	-1.4727644	-8.5318337	2.1374510
22 C	C22	-1.5466836	-7.4911578	3.0999742
23 C	C23	-1.2450116	-6.1767745	2.7321955
24 C	C24	6.2455163	0.7058589	-2.9039338
25 C	C25	7.4340771	0.7435737	-3.6386579
26 C	C26	7.6726152	-0.2216507	-4.6518291
27 C	C27	6.7448908	-1.2314347	-4.9371550
28 C	C28	5.5802236	-1.2514548	-4.1974644
29 N	N29	-1.9007421	-9.7247033	2.7692353
30 C	C30	-2.1126475	-9.4337665	4.1421186

31 C	C31	-1.9517755	-8.0846330	4.3493471
32 C	C32	8.5775218	1.6197707	-3.5972494
33 C	C33	9.4704400	1.1922030	-4.5507984
34 N	N34	8.9039326	0.1071353	-5.2687209
35 C	C35	-1.6393966	0.6833300	2.1416463
36 C	C36	-2.8326530	0.6243876	2.8505234
37 C	C37	-4.0217787	0.2810110	2.2017455
38 C	C38	-4.0009501	-0.0066068	0.8343937
39 C	C39	-2.8075299	0.0474992	0.1255735
40 C	C40	-0.4686730	2.3096972	-1.6583700
41 C	C41	-0.1637697	3.6016464	-2.0678714
42 C	C42	0.6005876	4.4377180	-1.2497022
43 C	C43	1.0527067	3.9665563	-0.0144219
44 C	C44	0.7438456	2.6765479	0.3984707
45 C	C45	-5.2833566	0.2225978	2.9530380
46 C	C46	0.9241439	5.8031922	-1.6859179
47 C	C47	2.2628805	6.2514680	-1.6044771
48 C	C48	2.6264867	7.5204722	-2.0063314
49 C	C49	1.6142610	8.3577896	-2.4920875
50 C	C50	0.2645457	7.9253985	-2.5711758
51 C	C51	-0.0788085	6.6310323	-2.1704069
52 C	C52	-5.3355972	-0.4457989	4.1683595
53 C	C53	-6.5513829	-0.4774302	4.8573875
54 C	C54	-7.6954454	0.1576049	4.3071424
55 C	C55	-7.6420695	0.8327935	3.0811451
56 C	C56	-6.4307308	0.8554198	2.4206837
57 C	C57	-6.9428120	-1.0602496	6.1160607
58 C	C58	-8.2743296	-0.7819817	6.3130109
59 N	N59	-8.7828513	-0.1009300	5.1764570
60 N	N60	1.6725873	9.6652969	-3.0325398
61 C	C61	0.3442590	10.0728568	-3.3209018
62 C	C62	-0.5112450	9.0209692	-3.0953854
63 H	H63	1.3209184	-1.8699022	1.9485518
64 H	H64	0.7633721	-4.1848189	2.6719802
65 H	H65	-1.7932795	-4.6423479	-0.7705245

66	H	H66	-1.2524872	-2.3186378	-1.4783850
67	H	H67	2.8937836	-0.2929216	0.7667696
68	H	H68	5.0259796	-0.1643105	-0.5097963
69	H	H69	2.8372187	-0.5785008	-4.2024939
70	H	H70	0.7055595	-0.6931477	-2.9242031
71	H	H71	-0.4930063	-6.7390148	-0.5533966
72	H	H72	-1.0310448	-9.0894985	0.0779053
73	H	H73	-1.3068390	-5.3681263	3.4682195
74	H	H74	6.0523860	1.4518519	-2.1257040
75	H	H75	6.9383585	-1.9763643	-5.7147180
76	H	H76	4.8291380	-2.0271565	-4.3876805
77	H	H77	-1.6351568	-10.6203157	2.4477862
78	H	H78	-2.4069045	-10.2036184	4.8556723
79	H	H79	-2.0996861	-7.5415263	5.2790803
80	H	H80	8.6915969	2.4654216	-2.9239974
81	H	H81	10.4468898	1.6047403	-4.8052468
82	H	H82	9.4511184	-0.5587887	-5.7515017
83	H	H83	-0.7091203	0.9625820	2.6489194
84	H	H84	-2.8443281	0.8505572	3.9228367
85	H	H85	-4.9299011	-0.2799312	0.3211779
86	H	H86	-2.7897705	-0.1854766	-0.9450986
87	H	H87	-1.0647593	1.6528907	-2.3015708
88	H	H88	-0.5210068	3.9685712	-3.0368152
89	H	H89	1.6507310	4.6195503	0.6312662
90	H	H90	1.0920815	2.3099615	1.3706192
91	H	H91	3.0241900	5.5653770	-1.2151052
92	H	H92	3.6645583	7.8607196	-1.9469601
93	H	H93	-1.1164474	6.2873357	-2.2386058
94	H	H94	-4.4516156	-0.9415352	4.5835227
95	H	H95	-8.5261660	1.3254736	2.6657446
96	H	H96	-6.3458240	1.3773275	1.4602701
97	H	H97	-6.2847150	-1.6192058	6.7763889
98	H	H98	-8.9117184	-1.0559729	7.1539036
99	H	H99	-9.5824186	0.4780611	5.2137669
100	H	H100	2.3894249	10.3043595	-2.8009514

101 H H101 0.1172195 11.0663175 -3.7078952

102 H H102 -1.5830866 8.9972493 -3.2740141

Table S8. Atomic charges of compound **34** calculated by the B3LYP/6-31G (d,p) method

Atom Label	Natural charge	Mulliken charge	Electrostatic charge
**C1**	-0.064	+0.086	+0.356
**C2**	-0.012	-0.065	-0.275
**C3**	-0.064	+0.090	+0.319
**C4**	-0.013	-0.054	-0.244
**C5**	-0.065	+0.086	+0.348
**C6**	-0.064	+0.089	+0.323
**C7**	-0.207	-0.159	-0.193
**C8**	-0.216	-0.170	-0.238
**C9**	-0.048	+0.067	+0.116
**C10**	-0.219	-0.171	-0.157
**C11**	-0.210	-0.168	-0.306
**C12**	-0.210	-0.168	-0.267
**C13**	-0.218	-0.172	-0.191
**C14**	-0.049	+0.067	+0.134
**C15**	-0.218	-0.173	-0.221
**C16**	-0.206	-0.160	-0.211
**C17**	-0.069	+0.059	+0.112
**C18**	-0.071	+0.057	+0.120
**C19**	-0.227	-0.187	-0.197
**C20**	-0.261	-0.164	-0.288
**C21**	+0.156	+0.304	+0.195
**C22**	-0.094	+0.119	+0.247
**C23**	-0.204	-0.245	-0.389
**C24**	-0.204	-0.244	-0.389

**C25**	-0.094	+0.120	+0.252
**C26**	+0.156	+0.303	+0.183
**C27**	-0.261	-0.164	-0.274
**C28**	-0.226	-0.187	-0.204
**C30**	-0.035	+0.044	-0.063
**C31**	-0.307	-0.221	-0.409
**C32**	-0.308	-0.222	-0.402
**C33**	-0.035	+0.044	-0.071
**C35**	-0.211	-0.168	-0.268
**C36**	-0.218	-0.171	-0.196
**C37**	-0.049	+0.065	+0.141
**C38**	-0.217	-0.171	-0.222
**C39**	-0.207	-0.159	-0.206
**C40**	-0.211	-0.167	-0.291
**C41**	-0.217	-0.171	-0.177
**C42**	-0.049	+0.070	+0.121
**C43**	-0.216	-0.171	-0.217
**C44**	-0.208	-0.158	-0.208
**C45**	-0.070	+0.058	+0.112
**C46**	-0.070	+0.059	+0.111
**C47**	-0.226	-0.189	-0.192
**C48**	-0.261	-0.164	-0.288
**C49**	+0.156	+0.303	+0.192
**C50**	-0.094	+0.120	+0.258
**C51**	-0.204	-0.245	-0.392
**C52**	-0.204	-0.244	-0.396
**C53**	-0.094	+0.120	+0.269
**C54**	+0.156	+0.304	+0.160
**C55**	-0.261	-0.164	-0.265

**C56**	-0.226	-0.186	-0.201
**C57**	-0.307	-0.222	-0.413
**C58**	-0.035	+0.044	-0.060
**C61**	-0.035	+0.044	-0.065
**C62**	-0.307	-0.221	-0.411
**H63**	+0.243	+0.135	+0.137
**H64**	+0.240	+0.131	+0.146
**H65**	+0.239	+0.130	+0.130
**H66**	+0.241	+0.133	+0.145
**H67**	+0.241	+0.134	+0.135
**H68**	+0.240	+0.131	+0.139
**H69**	+0.240	+0.131	+0.139
**H70**	+0.244	+0.136	+0.143
**H71**	+0.241	+0.130	+0.134
**H72**	+0.235	+0.122	+0.163
**H73**	+0.242	+0.135	+0.184
**H74**	+0.240	+0.130	+0.178
**H75**	+0.235	+0.122	+0.161
**H76**	+0.241	+0.129	+0.136
**H77**	+0.430	+0.327	+0.351
**H78**	+0.232	+0.150	+0.162
**H79**	+0.243	+0.131	+0.198
**H80**	+0.242	+0.130	+0.195
**H81**	+0.232	+0.150	+0.163
**H82**	+0.430	+0.327	+0.351
**H83**	+0.241	+0.134	+0.140
**H84**	+0.240	+0.131	+0.140
**H85**	+0.240	+0.131	+0.139
**H86**	+0.243	+0.135	+0.143

**H87**	+0.240	+0.132	+0.143
**H88**	+0.240	+0.130	+0.137
**H89**	+0.240	+0.130	+0.138
**H90**	+0.243	+0.134	+0.137
**H91**	+0.242	+0.134	+0.139
**H92**	+0.235	+0.123	+0.163
**H93**	+0.241	+0.131	+0.178
**H94**	+0.240	+0.130	+0.179
**H95**	+0.235	+0.122	+0.159
**H96**	+0.241	+0.129	+0.134
**H97**	+0.242	+0.130	+0.197
**H98**	+0.232	+0.150	+0.160
**H99**	+0.430	+0.327	+0.349
**H100**	+0.430	+0.327	+0.354
**H101**	+0.232	+0.150	+0.163
**H102**	+0.242	+0.130	+0.198
**N29**	-0.573	-0.687	-0.402
**N34**	-0.572	-0.686	-0.395
**N59**	-0.572	-0.687	-0.388
**N60**	-0.572	-0.686	-0.406

6. Electrochemical Measurements

The cyclic voltammetry (CV) technique was realized by CH Instruments 617D electrochemical workstation for electrochemical characterization. All CV measurements were performed under an argon (Ar) atmosphere and the electrochemical cell included an Ag wire as a reference electrode (RE), Pt wire as a counter electrode, and glassy carbon as a working electrode (WE) in a supporting electrolyte solution containing 0.1 M $TBAPF_6$ in acetonitrile. Molecules were drop-casted on the glassy carbon working carbon-working electrode before the potential was scanned in the monomer-free electrolyte solution.

Thermogravimetric analyses were realized by utilizing a Perkin Elmer Thermogravimetric Analyzer Pyris 1 TGA under a nitrogen (N_2) atmosphere at a heating rate of 10 °C/min. Thermal transitions were done *via* differential scanning calorimetry by using a Perkin Elmer, Jade DSC under a nitrogen atmosphere at a heating rate of 10 °C/min.

The dielectric properties of ITO/**TPE 33**/Al] devices were analyzed using HP 4194A Impedance Analyzer within the frequency range of 100 Hz–15 MHz at room temperature.

7. OLED Application

Device Fabrication and Characterization. The patterned ITO-coated glass substrates (120 nm, 5-10Ω/sq.) were supplied from Kintec Company. PEDOT: PSS was obtained from Heraeus Clevios GmbH. 0.45 µm PTFE and PVDF membrane filter (Millipore) was used to filter solution form molecules and PEDOT: PSS, respectively. For the fabrication of OLEDs, patterned ITO-coated glass substrates were cleaned ultrasonically in acetone, deionized water, and alcohol, in turn. Then, ozone treatments were applied for cleaning the substrates. PEDOT: PSS (~70 nm) as the hole injection layer (HIL) was spin-coated onto the pre-cleaned ITO-coated glass at 3000 rpm for 40 s and then baked at 120°C for 20 min. The **TPEs 9**, **32**, **33**, and **34** were prepared by dissolving in 1,2-dichlorobenzene (20 mg/mL) and growing on top of the HIL by spin casting at 1000 rpm for 40 s then baked at 50°C for 10 min in the glove box. TPBi (~40 nm) as the electron transport layer (ETL) was grown in a thermal vacuum chamber before Lithiumfloride (LiF) as an EIL (~0.5 nm) and cathode layer of aluminum (~110 nm) was deposited by vacuum evaporation (5x10-6 mbar) method. The devices with ITO/PEDOT: PSS/**TPE 9**-**32**-**33**-**34** (~ 40 nm)/TPBi/LiF/Al structure were fabricated. For the characterization of OLEDs, the Hamamatsu PMA-12 C10027 Photonic Multichannel analyzer and a digital multimeter (2427-C 3A Keithley) were used in a dark sample chamber after UV epoxy encapsulation of the devices. A stylus profiler (KLA Tencor P-6) was utilized to measure the thickness of organic layers.

Photophysical Measurements: The optical transmission spectra were measured and recorded using an FS5 spectrofluorometer (Edinburg Inst).
